# Endohedrally Functionalized Heteroleptic Coordination Cages for Phosphate Ester Binding

**DOI:** 10.1002/anie.202209305

**Published:** 2022-10-19

**Authors:** André Platzek, Selina Juber, Cem Yurtseven, Shota Hasegawa, Laura Schneider, Christoph Drechsler, Kristina E. Ebbert, Robin Rudolf, Qian‐Qian Yan, Julian J. Holstein, Lars V. Schäfer, Guido H. Clever

**Affiliations:** ^1^ Department of Chemistry and Chemical Biology TU Dortmund University Otto-Hahn-Straße 6 44227 Dortmund Germany; ^2^ Theoretical Chemistry Ruhr University Bochum 44780 Bochum Germany

**Keywords:** Coordination Cages, Host–Guest Chemistry, MD Simulation, Self-Assembly, Supramolecular Chemistry

## Abstract

Metallosupramolecular hosts of nanoscopic dimensions, which are able to serve as selective receptors and catalysts, are usually composed of only one type of organic ligand, restricting diversity in terms of cavity shape and functional group decoration. We report a series of heteroleptic [Pd_2_A_2_B_2_] coordination cages that self‐assemble from a library of shape complementary bis‐monodentate ligands in a non‐statistical fashion. Ligands A feature an inward pointing NH function, able to engage in hydrogen bonding and amenable to being functionalized with amide and alkyl substituents. Ligands B comprise tricyclic aromatic backbones of different shape and electronic situation. The obtained heteroleptic coordination cages were investigated for their ability to bind phosphate diesters as guests. All‐atom molecular dynamics (MD) simulations in explicit solvent were conducted to understand the mechanistic relationships behind the experimentally determined guest affinities.

## Graphical abstract

A series of heteroleptic [Pd_2_A_2_B_2_] coordination cages was prepared by shape‐complementary assembly (SCA) from a modular set of ligands and screened for binding phosphate guests. All‐atom molecular dynamics simulations reveal that an endohedral hydrogen bond donor site is the decisive but not exclusive factor for binding phosphate diesters inside the cavity in a competitive solvent.

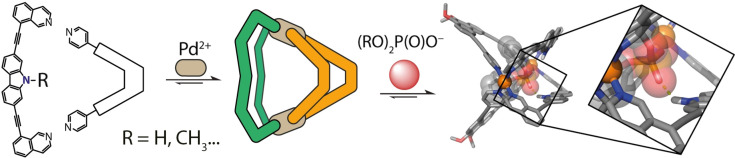

Anionic phosphate esters play major roles in biological systems as parts of structure‐giving biominerals, nucleic acids, phosphorylated proteins, lipids, sugars and metabolites. Phosphodiesters, in focus here, are found in key biological structures such as oligonucleotides, phosphatidylcholines and small molecules such as 3′,5′‐cyclic adenosine monophosphate. Furthermore, they are produced on large industrial scale, e.g. as fertilizer components, pesticides, flame retardants and softeners and their spread and bioaccumulation present severe environmental hazards.[^1^, ^2^, ^3^, ^4^] In the past decades, numerous synthetic receptor molecules for phosphate binding have been described.[^5^, ^6^, ^7^, ^8^, ^9^, ^10^] Many of these receptors consist of pyridinium, imidazolium and macrocyclic ammonium salts as well as organic molecules containing urea and thiourea moieties to recognize phosphate anions by charge and hydrogen bonding interactions.[^9^, ^11^, ^12^, ^13^, ^14^, ^15^] Besides organic receptor molecules, a variety of metal complexes^[16]^ and self‐assembled coordination cages^[17]^ have been developed for recognizing anions, including phosphates, as well as neutral organophosphates and phosphonates.[^18^, ^19^, ^20^, ^21^] For example, a variety of Zn^2+^ and Cu^2+^ complexes were introduced as sensors for nucleotides.[^22^, ^23^]

A large number of coordination‐based rings and cages, capable of binding guests inside their nanoscale cavities, have been described in the recent years based on various metal‐ligand combinations.[^24^, ^25^, ^26^, ^27^, ^28^, ^29^, ^30^, ^31^] A prominent subcategory are metallo‐supramolecular cages built from square‐planar coordinated Pd^II^ cations and nitrogen donor‐based bridging ligands.[^32^, ^33^, ^34^, ^35^, ^36^, ^37^, ^38^] Among these, dinuclear lantern‐shaped cages of [Pd_2_L_4_] stoichiometry represent the host structures of lowest nuclearity, a compound class we have mainly in focused on in recent years.^[39]^

So far, the vast majority of reported coordination cages have been assembled using only one kind of ligand per structure, owing to the stochastic nature of thermodynamically driven self‐assembly under dynamic ligand exchange conditions, following simple geometric rules. However, while corresponding assembly products often exhibit intriguing beauty concerning their highly symmetric shapes, e.g. following the Platonic or Archimedean solids, this can also restrict the versatility of the created voids and their equipment with chemical functionality.

To gain more structural and functional diversity, we foster the development of assembly strategies aimed at the high‐yielding, non‐statistical synthesis of heteroleptic coordination cages composed of two or even more different ligands within one metallosupramolecular architecture.[^40^, ^41^, ^42^, ^43^, ^44^, ^45^, ^46^, ^47^, ^48^, ^49^, ^50^, ^51^, ^52^] Recently, two robust approaches towards heteroleptic cage formation were established for which we coined the terms “Coordination Sphere Engineering” (CSE) and “Shape Complementary Assembly” (SCA). The former method is based on ligand interactions (such as steric bulk^[53]^ or hydrogen bonding[^54^, ^55^]) in direct vicinity of the structure's metal nodes, the latter, used herein, brings together pairs of ligands that match in terms of their overall geometry to integratively self‐sort into a discrete assembly that outcompetes homoleptic assembly products in terms of enthalpic and/or entropic drive.^[56]^ These strategies not only allow us to create structural diversity of self‐assembled hosts from a modular set of ligands^[57]^ but set the foundation to combine different exo‐ and/or endohedral functionalities and exploit their interplay.

Here, we report a series of heteroleptic [Pd_2_A_2_B_2_] coordination cages that self‐assemble from a library of shape complementary bis‐monodentate ligands A=**L^1^** ^–**4**^ and B=**L^A–D^** in a non‐statistical fashion (Figure 1). Banana‐shaped ligands **L^1^** ^–**4**^ were synthesized via sonogashira cross coupling of 2,7‐dibromo‐carbazole derivatives with 7‐alkinyl isoquinoline donor groups. Ligand **L^1^** is based on *N*‐unsubstituted carbazole, whereas methylation of the secondary amine lead to ligand **L^2^**. The reaction of 2,7‐dibromo‐carbazole with carbamates and isocyanates, respectively, produced the backbones for the urea‐type ligands **L^3^** and **L^4^**. The syntheses of the shape complementary ligands **L^A–B^** were already reported in previous studies.[^40^, ^57^] Ligands **L^C–D^** were synthesized via suzuki cross coupling reactions of 3,6‐dibromo *N*‐hexylcarbazole and 3,6‐dibromo fluorenone, respectively, with 4‐pyridineboronic acid pinacol ester.

**Figure 1:**
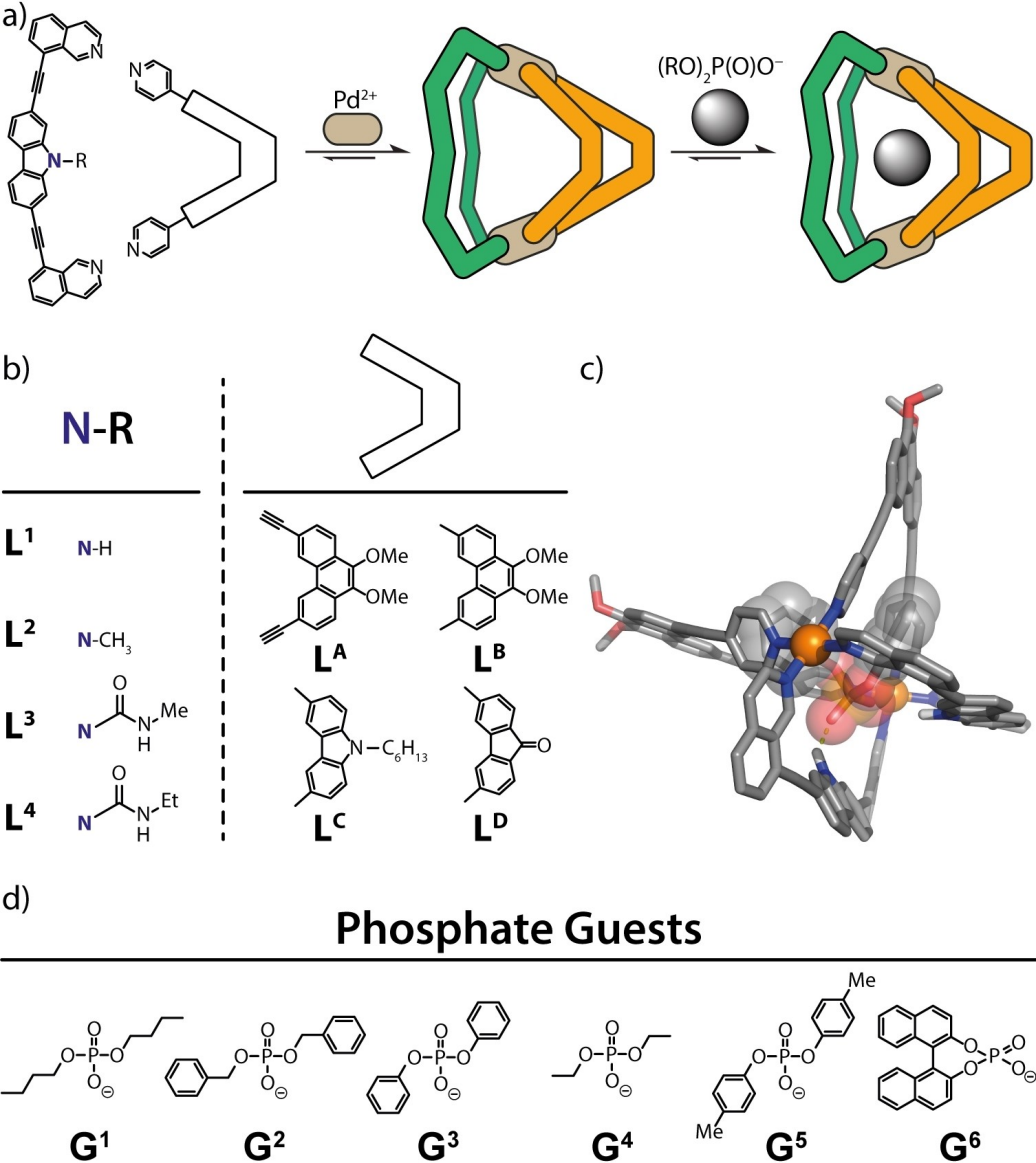
a) Heteroleptic cage formation and guest uptake, b) shape complementary ligands **L^1^** ^–**4**^ and **L^A–D^**, c) MD‐simulated structure of host–guest‐complex [**G^3^** @Pd_2_ **L^1^**_2_ **L^A^**_2_] and d) examined phosphate ester guests **G^1^** ^–**6**^.

The addition of 0.55 eq Pd^II^ cations as solution of the [Pd(MeCN)_4_](BF_4_)_2_ salt to a 1 : 1 mixture of solutions of one of ligands **L^1^** ^–**4**^ with one of ligands **L^A–D^** in DMSO‐d_6_ and DMF‐d_7_ leads to the quantitative formation of the corresponding heteroleptic coordination cages of the type [Pd_2_ **L^1^** ^–**4**^_2_ **L^A–D^**_2_] after a few minutes at room temperature (Supporting Information, Figures S28–76).

As an example, the self‐assembly of cage [Pd_2_ **L^1^**_2_ **L^A^**_2_] was confirmed by ^1^H NMR measurements, as downfield shifting of the signals of protons in close proximity to the nitrogen donors of both ligands was observed (Figure 2b). The signals of proton c′ and the secondary amine were also shifted downfield as these are pointing into the newly formed cavity. Furthermore, the formation of a single discrete complex composed of both ligands was confirmed by ^1^H DOSY NMR measurement, showing that only one species is formed in solution (Figure 2c). The hydrodynamic radius *r*_H_=11.97 Å was calculated by using the stokes–einstein equation and is in good accordance to other reported heteroleptic coordination cages.[^42^, ^54^] ESI‐MS measurements support the NMR data. Peaks for differently charged variants of the same species, namely [Pd_2_ **L^1^**_2_ **L^A^**_2_]^4+^, [Pd_2_ **L^1^**_2_ **L^A^**_2_+BF_4_]^3+^ and [Pd_2_ **L^1^**_2_ **L^A^**_2_+2BF_4_]^2+^ dominate the respective mass spectrum.

**Figure 2:**
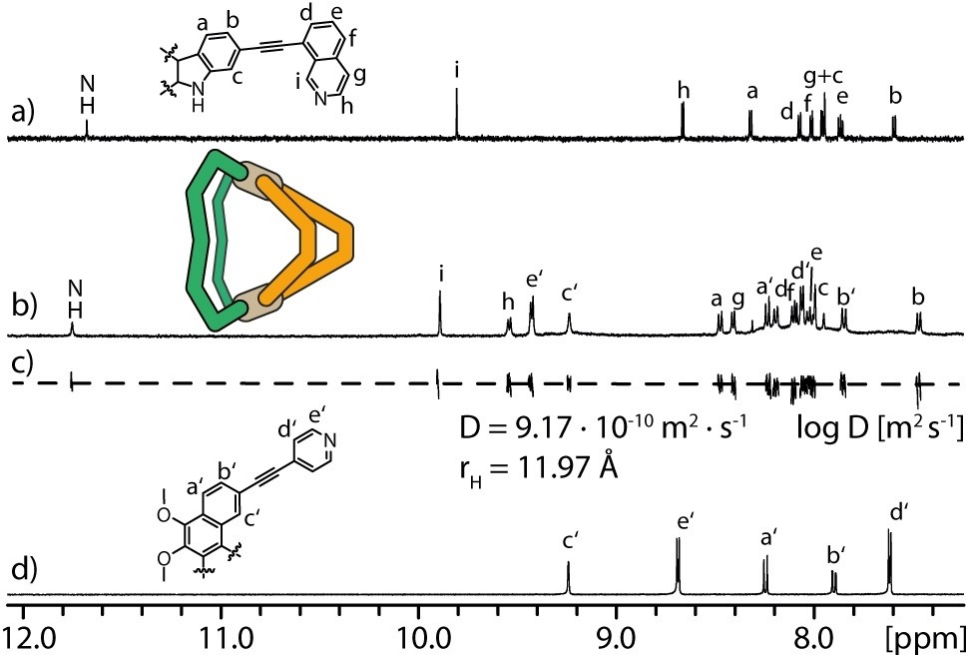
Partial ^1^H NMR spectra of ligands a) **L^1^** and d) **L^A^**, b) heteroleptic coordination cage [Pd_2_ **L^1^**_2_ **L^A^**_2_] and c) partial ^1^H DOSY NMR spectrum of coordination cage, diffusion coefficient *D* and hydrodynamic radius *r*_H_.

For compound [Pd_2_ **L^1^**_2_ **L^A^**_2_] we were able to obtain single crystals via slow vapor diffusion of toluene into solution of cage in DMF (Figure 3a). The obtained structure confirms the *cis*‐arrangement of pairs of ligands **L^1^** and **L^A^** around the two square‐planar Pd^II^ centers, sitting in a distance of 14.403(5) Å. The carbazole planes of ligands **L^1^** are almost coplanar with a N−N distance of 6.727(7) Å. Inspection of the structure reveals that the inner cavity can essentially be accessed from three directions, i.e. through the apertures between the neighboring unlike ligands (Figure 3a, left) and through a wide channel between ligands **L^A^** (Figure 3a, right). The X‐ray analysis further showed that the coordination cage contains two BF_4_^−^ anions in the cavity that are in close proximity to the secondary amines of **L^1^** to which they form hydrogen bonds (closest H−F distance=2.109 Å). Furthermore, two encapsulated DMF molecules with their oxygen atom pointing towards the Pd centers were observed to be sandwiched between the carbazole moieties of ligands **L^1^** in the crystal structure.

**Figure 3:**
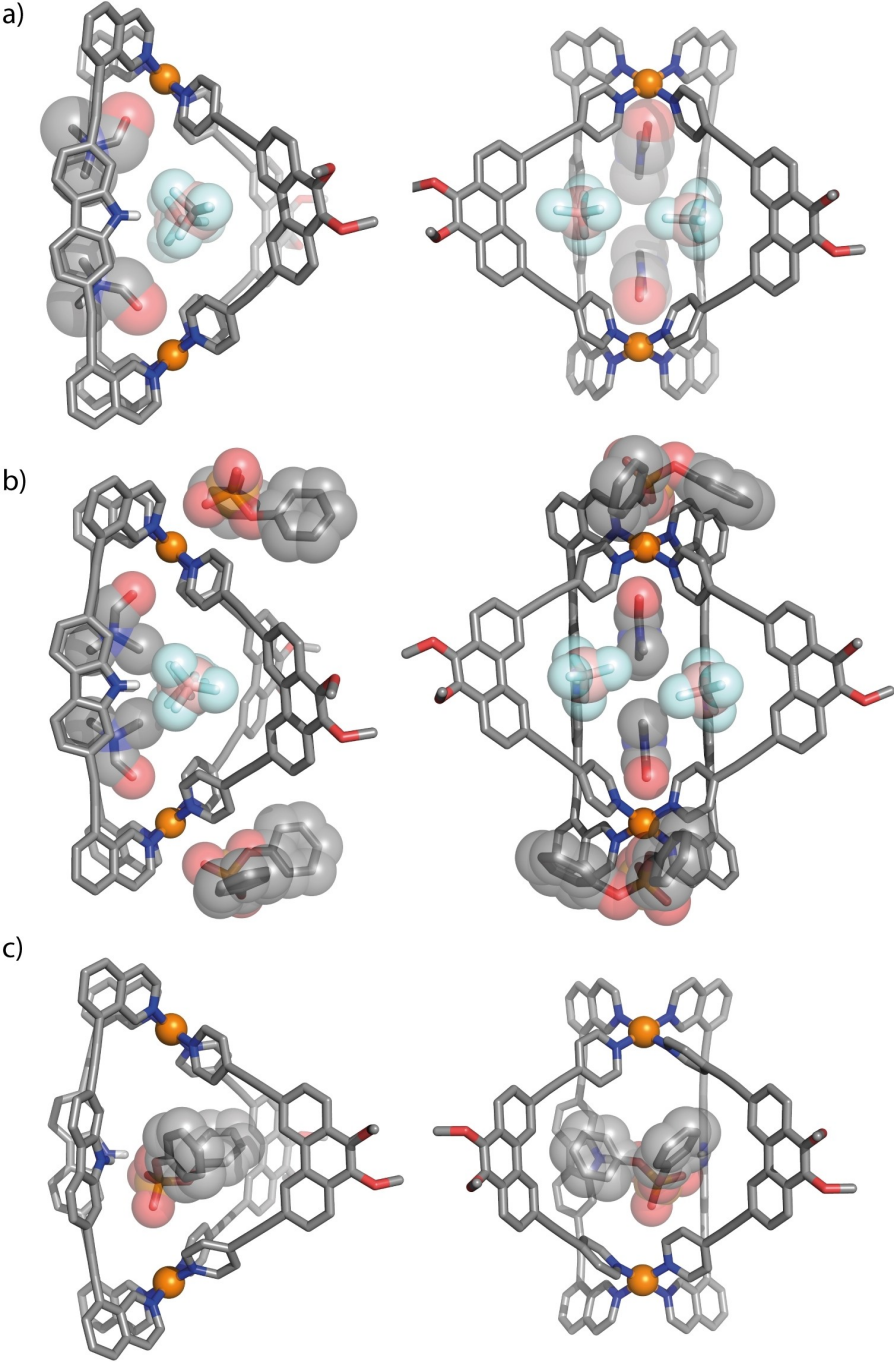
X‐ray structures of a) [Pd_2_ **L^1^**_2_ **L^A^**_2_], b) [Pd_2_ **L^1^**_2_ **L^A^**_2_+2**G^3^**] (both containing two molecules of DMF and BF_4_^−^, each) and c) MD simulation of [**G^3^** @Pd_2_ **L^1^**_2_ **L^A^**_2_].

Next, we tested the uptake of phosphate diesters as guests. Therefore, phosphate esters were purchased as free acids and neutralized with tetra‐*n*‐butyl ammonium hydroxide to form the corresponding salts of the type (RO)_2_P(O)O^−^(NBu_4_)^+^. Different aliphatic and aromatic groups were used as R (Figure 1d). While all examined phosphates showed signs of interaction with the cationic host, NMR signal broadening hampered deeper characterization of some of the formed host–guest complexes, in particular for the aliphatic esters. Aromatic guest **G^3^** was found to behave best in terms of NMR spectroscopic investigation and was therefore chosen for the following experiments. The ^1^H NMR titration of cage [Pd_2_ **L^1^**_2_ **L^A^**_2_] with diphenyl phosphate **G^3^** revealed a prominent downfield shift for the signal assigned to the cage's inward pointing NH proton (ligand **L^1^**), indicating guest uptake concomitant with hydrogen bonding, most probably to a phosphate oxygen (Figure 4c). Further signals of the cage's inward pointing protons were found to shift downfield (e.g. signals of protons c, i, c′, e′). Furthermore, 1H–1H‐NOESY analysis revealed crosspeaks between phenyl proton signals of **G^3^** and inward pointing proton signals c′ and d′ of ligand **L^A^**, representing another strong indication for inside binding and indicating that the guest's phenyl groups are oriented towards this rather hydrophobic ligand (Supporting Information, Figure S101). Since only one set of gradually shifting signals was observed in the NMR data, it is clear that the binding of the phosphate is in fast exchange compared to the NMR time scale. From the plot of the Δ*δ* values of selected proton signals over the equivalents of added guest, the software Bindfit yielded a binding constant of K=2062±31 M^−1^.[^58^, ^59^, ^60^] Owing to solubility issues, experiments had to be conducted in DMSO, although this solvent is known to be a strong competitor in terms of hydrogen bonding. In addition, some titrations were performed in DMF‐d_7_ and similar binding constants were obtained (Supporting Information, Table S1).

**Figure 4:**
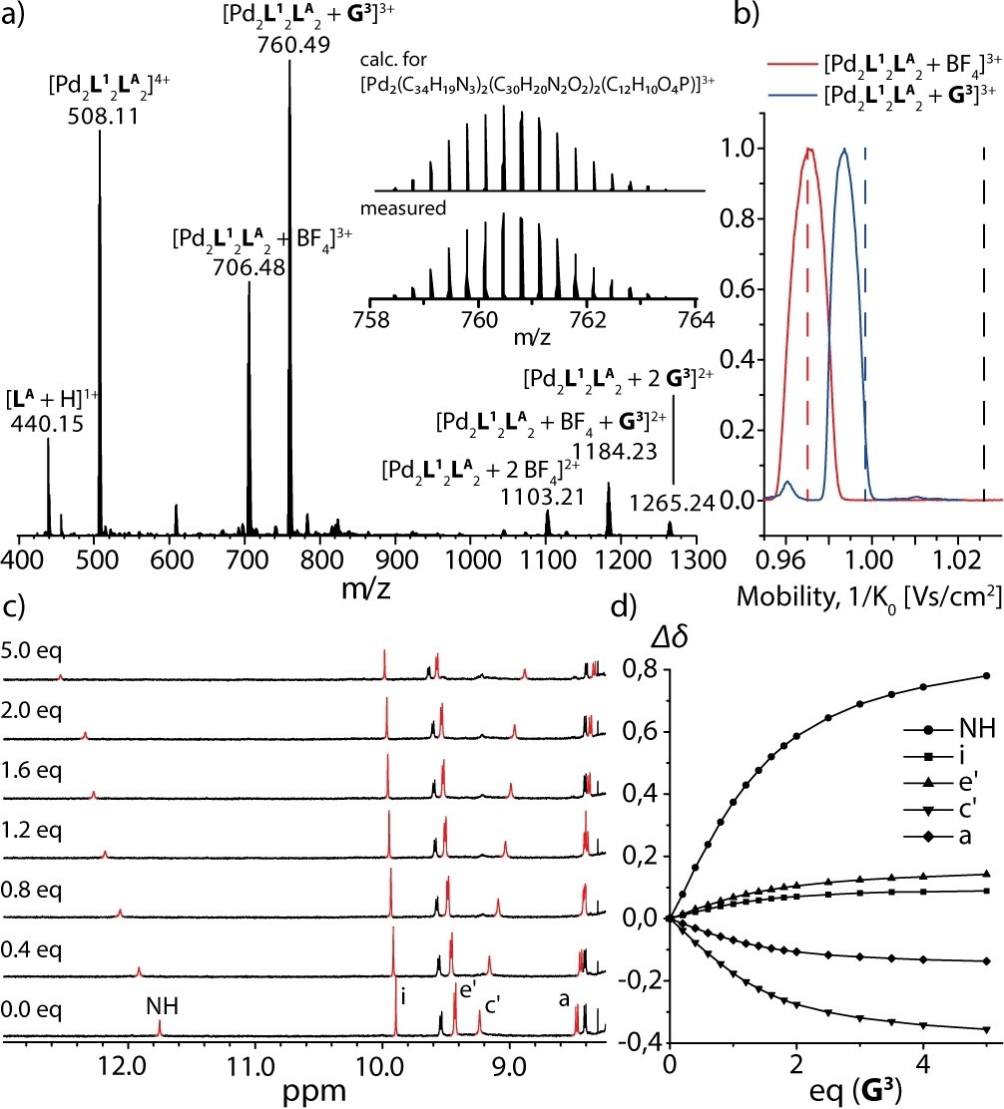
a) ESI‐MS spectrum of [**G^3^** @Pd_2_ **L^1^**_2_ **L^A^**_2_], b) experimental ion mobilities of [BF_4_@Pd_2_ **L^1^**_2_ **L^A^**_2_]^3+^ (solid red line) and [**G^3^** @Pd_2_ **L^1^**_2_ **L^A^**_2_]^3+^ (solid blue line) and theoretically determined ion mobilities of [BF_4_@Pd_2_ **L^1^**_2_ **L^A^**_2_]^3+^ (dashed red line) and [**G^3^** @Pd_2_ **L^1^**_2_ **L^A^**_2_]^3+^ (dashed blue line) compared to the mobilitiy of **G^3^** placed outside of [Pd_2_ **L^1^**_2_ **L^A^**_2_]^4+^ (dashed black line). All theoretical values were scaled by a factor of 0.934 (for exp. and theor. collisional cross sections e/tCCS, see Supporting Information), c) partial ^1^H NMR spectra of the titration of **G^3^** to [Pd_2_ **L^1^**_2_ **L^A^**_2_] and d) plot of Δ*δ* of selected proton signals against guest concentration.

To prove that guest binding is mainly driven by the formation of hydrogen bonds, the titration experiment was repeated with cage [Pd_2_ **L^2^**_2_ **L^A^**_2_], containing ligand **L^2^** instead of **L^1^**, in which the carbazoles’ hydrogen bond donor functions are blocked by methyl substituents. Upon addition of **G^3^**, a downfield shift of signals of the inner protons was still observed, though being much smaller compared to the situation with [Pd_2_ **L^1^**_2_ **L^A^**_2_]. A much lower binding constant of K=77±3 M^−1^ was obtained accordingly, as the association should now be limited to overall weaker non‐covalent interactions between the cationic host and the phosphate, moreover in a very polar solvent environment. Titration of the other aromatic guests to cage [Pd_2_ **L^1^**_2_ **L^A^**_2_] also showed binding to the host with comparable binding constants (Table S1). Remarkably, chiral guest **G^6^** was also found to bind inside the host, giving rise to a slight guest‐to‐host chiral induction effect, as evidenced by CD spectral examination (Supporting Information). Furthermore, titration of aliphatic guests **G^1^** and **G^4^** indicated binding by shifting of the corresponding signals. Unfortunately, binding constants could not be determined due to a strong signal broadening after addition of more than 3 eq of guest. However, a guest competition experiment with cage [Pd_2_ **L^1^**_2_ **L^A^**_2_] and 1 eq of diphenyl phosphate guest **G^3^** as well as 1 eq of diethyl phosphate **G^4^** clearly revealed that the aromatic guest is binding stronger than the aliphatic (Supporting Information). Clear indication for guest uptake was further obtained by trapped ion mobility spectrometry (TIMS), coupled to high‐resolution ESI‐TOF mass spectrometry, as the gas‐phase collisional cross sections (CCS) of the host–guest complexes did not change significantly compared to the free host. This is a strong indication for inside binding.^[61]^ The aromatic guest molecules show the same behavior (see Figure 4a for the ESI mass spectrum and 4b for comparison of ion mobilities of free and guest‐bound host). These results were also supported by comparing experimental results with theoretically calculated CCS values, that show the same trend (Supporting Information, Table S2). In contrast, the simulated tCCS value for a tentative structure representing an outside binding mode (with the guest sitting close to one of the outer Pd‐faces) would give rise to a much larger increase of the aggregate size, in disagreement with the experimental observation (Figure 4b).

To gain atomic‐level insights into the molecular interactions underlying the experimentally observed differences in guest binding by cages [Pd_2_ **L^1^**_2_ **L^A^**_2_], carrying endohedral NH hydrogen bond donors, and [Pd_2_ **L^2^**_2_ **L^A^**_2_], with these nitrogens methylated, all‐atom MD simulations in explicit DMSO solvent were performed (see Supporting Information). Multiple spontaneous binding and unbinding events of the **G^3^** guest were observed during the extended MD simulation timescale of 50 μs, allowing to estimate the free energies of binding.^[62]^ The standard state binding free energies are −13.3±1.4 and −6.6±0.2 kJ mol^−1^ for cages [Pd_2_ **L^1^**_2_ **L^A^**_2_] and [Pd_2_ **L^2^**_2_ **L^A^**_2_], respectively. Thus, in line with the experimentally measured differences in the association constants (see above), the lack of the H‐bonding capability in **L^2^** reduces binding affinity. To explain the surprisingly small free energy difference, the molecular interactions that govern the host–guest binding were characterized more closely. First, a contact analysis was performed, considering the likelihood of interactions between individual host features (e.g. the NH or NCH_3_ groups or the aromatic moieties of the ligands) and functionalities of the guest (i.e. the anionic phosphate and the phenyl groups). To that end, the chunks of the MD simulation trajectories were analyzed during which the guest was bound inside the cage cavity. Figure 5a shows that in [Pd_2_ **L^1^**_2_ **L^A^**_2_], binding of the **G^3^** guest is mediated by both, H‐bonds between the phosphate and the NH groups of the cage and, in addition, by nonpolar contacts between the **G^3^** phenyl groups and the different chemical moieties of the cage (note that these types of interactions are not mutually exclusive). In the methylated [Pd_2_ **L^2^**_2_ **L^A^**_2_] cage, the lack of the H‐bonding capability is partially compensated by an increase of nonpolar contacts, especially with **L^A^** (Figure 5b). This compensation mechanism can explain the relatively small differences in the binding affinities. This notion is supported by additional analyses of the interaction energies in the simulations (Table S4). While the above mentioned contact analysis suggests only relatively small differences between the two cages, these come along with a strong loss of interaction energy by 38 kJ mol^−1^. Interestingly, the large difference in interaction energy between **L^1^** and **L^2^** due to the loss of the H‐bonding capability is (again, comparable to the contact analysis interpretation) partially compensated by more favorable interactions between the guest and hydrophobic ligand **L^A^** in the methylated cage (Table S4). In addition, to investigate the selectivity of the cage towards guests of different size, we repeated the MD simulations also for guest **G^4^** binding to cage [Pd_2_ **L^2^**_2_ **L^A^**_2_] (Supporting Information; unfortunately, simulations for [**G^4^** @Pd_2_ **L^1^**_2_ **L^A^**_2_] suffered from a large kinetic barrier for guest unbinding, prohibiting the extraction of statistically reliable results). As expected, binding of the smaller diethyl phosphate guest is much weaker than for **G^3^**, with a binding free energy of about −1±1 kJ mol^−1^. Closer analyses of the interaction energies between [Pd_2_ **L^2^**_2_ **L^A^**_2_] and **G^4^** (Table S5) show that this smaller affinity can be attributed to a loss of favorable interactions of the guest with the extended aromatic surfaces of ligands **L^2^** and **L^A^**. This result suggests that instead of the mere size of the guest, the nature of the phosphate substituents (aliphatic vs. aromatic) plays an important role as well.

**Figure 5:**
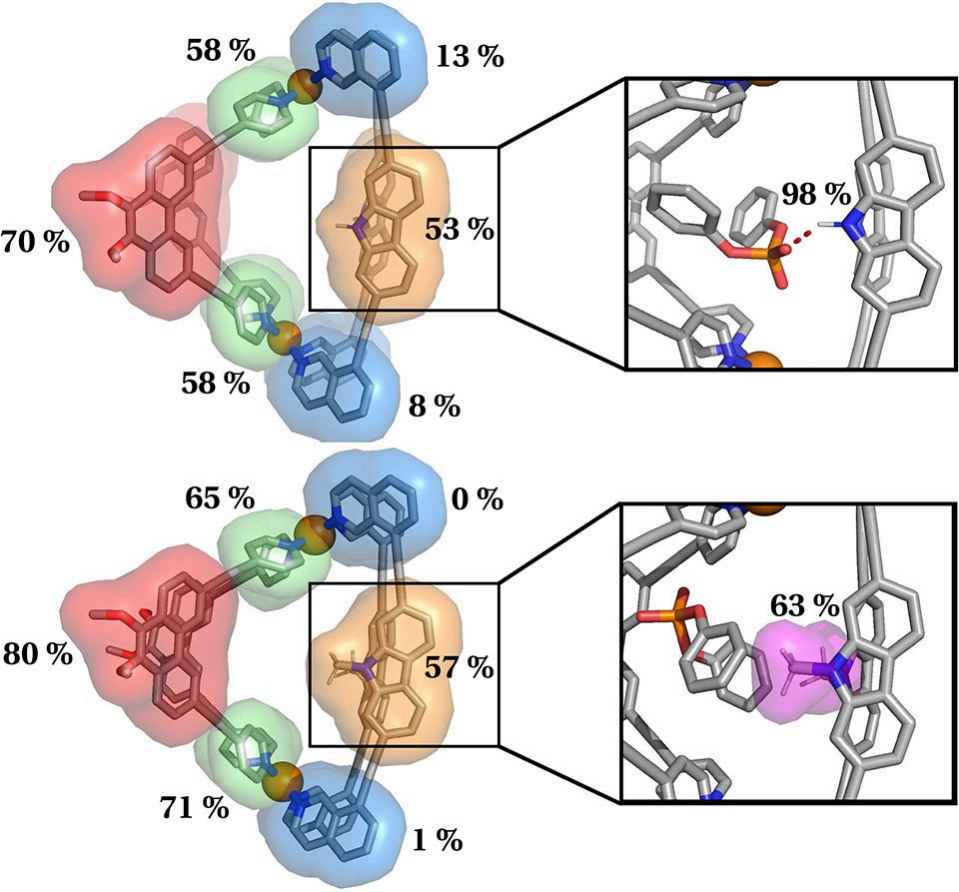
Characterization of cage‐guest contacts during MD simulations of guest **G^3^** bound to a) [Pd_2_ **L^1^**_2_ **L^A^**_2_] and b) methylated cage [Pd_2_ **L^2^**_2_ **L^A^**_2_]. The percentages indicate the likelihood that the bound guest forms the respective interactions with different cage features. These interactions include contacts between the guest and the different aromatic surfaces of the ligands (colored surfaces) and H‐bonds with the NH groups of cage [Pd_2_ **L^1^**_2_ **L^A^**_2_] or nonpolar contacts between the guest and the N‐methyl groups of [Pd_2_ **L^2^**_2_ **L^A^**_2_] (insets at the right).

In addition, we tried to obtain further insight from a solid‐state structure of the host–guest complex. We were able to obtain single crystals of [**G^3^** @Pd_2_ **L^1^**_2_ **L^A^**_2_] and [**G^5^** @Pd_2_ **L^1^**_2_ **L^A^**_2_] via slow vapor diffusion of Et_2_O into solutions of the host–guest compounds in DMF. Surprisingly, the phosphate esters were found to be not located inside the pocket of the host but interact with the Pd cations from the outer faces of the coordination cages in the densely packed solid state structures (Figure 3b), while the cavity contains two BF_4_^−^ anions (hydrogen‐bonding to the ligands’ NH functions) and two DMF molecules (pointing with their oxygen substituents to the axial positions of the inner Pd complexes), positioned in exactly the same locations as in the structure of the parental host shown in Figure 3a.

Next, the modular assembly approach was employed to examine the combination of **L^1^** with shape complementary, but significantly shorter ligands **L^B–D^**, giving rise to cages with decreased cavity volumes. Again, we investigated binding of the phosphate esters. In all cases, titration of **G^3^** lead to a downfield shift of the NH proton signal of **L^1^**, indicating guest encapsulation via hydrogen bonding interactions. At the same time, further signals of the inward pointing protons of the ligands were shifting as well. In comparison to the titration experiments with [Pd_2_ **L^1^**_2_ **L^A^**_2_], however, the binding constants decreased significantly. For example, a binding constant of K=98±1 M^−1^ was calculated for [**G^3^** @Pd_2_ **L^1^**_2_ **L^B^**_2_] and constants for titration of **G^3^** to the other cage derivatives containing ligands **L^C^** and **L^D^** are in a comparable, low range (Table 1). Thus, we can assume that the decrease of the cavity size also correlates with the binding affinity to the guest molecule.

**Table 1:** Overview of binding constants K for encapsulation of **G^3^**.

	[Pd_2_ **L^1^**_2_ **L^A^**_2_]	[Pd_2_ **L^1^**_2_ **L^B^**_2_]	[Pd_2_ **L^1^**_2_ **L^C^**_2_]	[Pd_2_ **L^1^**_2_ **L^D^**_2_]
K [M^−1^]	2062±31	98±1	33±1	149±1
	[Pd_2_ **L^2^**_2_ **L^A^**_2_]	[Pd_2_ **L^2^**_2_ **L^B^**_2_]	[Pd_2_ **L^3^**_2_ **L^A^**_2_]	[Pd_2_ **L^4^**_2_ **L^A^**_2_]
K [M^−1^]	77±3	no binding	46±2	128±3

Interestingly, **G^3^** shows no binding to [Pd_2_ **L^2^**_2_ **L^B^**_2_] at all, where the carbazole NH sites are again blocked by methyl substituents and therefore exclude hydrogen bonding to the guest. In addition, N‐methylation also diminishes the cavity volume, which, together with the shorter linkers of ligand **L^B^** most likely renders the cage's pocket too small for effective guest uptake in this case. Changing the counter ligand to non‐phenanthrene‐based systems **L^C^** and **L^D^** also leads to weak binding. For example, a binding constant of K=33±1 M^−1^ was obtained from a titration of **G^3^** to [Pd_2_ **L^1^**_2_ **L^C^**_2_] (Table 1 and Supporting Information).

Titration of **G^3^** to cage derivatives [Pd_2_ **L^3^**_2_ **L^A^**_2_] and [Pd_2_ **L^4^**_2_ **L^A^**_2_], containing alkylaminoacyl‐substituted carbazole nitrogen positions, also lead to much lower binding constants (Table 1) as compared to formation of the host–guest‐complex [**G^3^** @Pd_2_ **L^1^**_2_ **L^A^**_2_]. While the herein used ligands **L^3^** and **L^4^** should also be able to offer endohedral NH functionalities for hydrogen bonding to the guests, these are not pointing directly into the center of the cavity and the inner space available for guest binding is also lower.

In conclusion, eight members of a new family of heteroleptic coordination cages with endohedral functionalities were synthesized by shape complementary assembly (SCA) in a modular, non‐statistical fashion. The uptake of different phosphate esters was quantified by NMR titrations and it was revealed by all‐atom MD simulations that strong binding is mainly driven by hydrogen bonding to the endohedral NH functions of ligands **L^1^** of the best performing host [Pd_2_ **L^1^**_2_ **L^A^**_2_], despite the use of DMSO or DMF as competitive solvents. However, guest binding was also found inside cages with blocked amine function, albeit with lower binding constants, showing that other structural host features, such as π‐surfaces of the ligands opposite the amines, play important roles as well. Indeed, NOESY analysis and MD simulations revealed that counter ligand **L^A^** serves as hydrophobic binding partner to diphenyl phosphate guest **G^3^** but not to diethyl phosphate guest **G^4^**.

We herein show that the recently growing class of heteroleptic metallosupramolecular cages^[56]^ is amenable to selective endohedral functionalization and modular combination of distinctive functionalities. Hence, they promise to serve as a platform to create cage libraries for screening guest selectivity, e.g. of biologically relevant phosphates and for catalytic conversions under nanoscopic confinement. Their anisotropic shape, together with additional exohedral derivatization, further allows for engineering solubility and promote higher‐order aggregation, e.g. into vesicles as previously shown by us for comparable assemblies.^[63]^ Together, the combination of metal‐mediated architecture, systematic guest binding and MD simulation yielded deep insights into individual host–guest interaction features across a family of cages, forming the basis for a rational design strategy towards tailormade hosts.

## Conflict of interest

The authors declare no conflict of interest.

## Supporting information

As a service to our authors and readers, this journal provides supporting information supplied by the authors. Such materials are peer reviewed and may be re‐organized for online delivery, but are not copy‐edited or typeset. Technical support issues arising from supporting information (other than missing files) should be addressed to the authors.

Supporting Information

Supporting Information

Supporting Information

Supporting Information

## Data Availability

The data that support the findings of this study are available in the supplementary material of this article.
