# Supplementary material for: Endohedrally Functionalized Heteroleptic Coordination Cages for Phosphate Ester Binding
Source: Angew Chem Int Ed Engl. 2022 Oct 19;61(47):e202209305. doi: 10.1002/anie.202209305 (PMC9828229; doi:10.1002/anie.202209305)
Supplement: Supplementary file 4 — Supporting Information [file ANIE-61-0-s003.pdf]

Angewandte Chemie
Eine Zeitschrift der Gesellschaft Deutscher Chemiker

Supporting Information

Endohedrally Functionalized Heteroleptic Coordination Cages for Phosphate Ester Binding

*A. Platzek, S. Juber, C. Yurtseven, S. Hasegawa, L. Schneider, C. Drechsler, K. E. Ebbert, R. Rudolf, Q.-Q. Yan, J. J. Holstein, L. V. Schäfer, G. H. Clever**

Table of contents

1 General Methods 3

1.1 NMR Spectroscopy 3

1.2 X-Ray single crystal structure determination 3

1.3 Mass spectrometry and ion mobility measurements 3

1.4 Gaschromatography electron ionization mass spectrometry (GC/EI-MS) 3

1.5 GPC 4

1.6 UV/Vis spectroscopy 4

1.7 CD spectroscopy 4

2 Experimental Procedures 4

2.1 Synthesis of 8-ethinylisoquinoline 1 5

2.2 Synthesis of 2,7-bis(isoquinolin-8-ylethinyl)-9*H*-carbazole L^1 6

2.3 Synthesis of 2,7-dibromo-9-methyl-9*H*-carbazole 2 7

2.4 Synthesis of 2,7-bis(isoquinolin-8-ylethinyl)-9-methyl-9*H*-carbazole L^2 9

2.5 Synthesis of 2,7-dibromo-*N*-methyl-9*H*-carbazole-9-carboxamide 3 10

2.6 Synthesis of 2,7-bis(isoquinolin-8-ylethinyl)-*N*-methyl-9*H*-carbazole-9-carboxamide 11

2.7 Synthesis of 2,7-dibromo-*N*-ethyl-9*H*-carbazole-9-carboxamide 4 13

2.8 Synthesis of 2,7-bis(isoquinolin-8-ylethinyl)-*N*-ethyl-9*H*-carbazole-9-carboxamide L^4 14

2.9 Synthesis of 3,6-dibromo-9-hexyl-9*H*-carbazole 5 15

2.10 Synthesis of 9-hexyl-3,6-di(pyridine-4-yl)-9H-carbazole L^C 16

2.11 Synthesis of 3,6-di(pyridine-4-yl)-9*H*-fluoren-9-one L^D 18

2.12 General synthesis of heteroleptic coordination cages 19

2.12.1 $Pd_2L^1_2L^A_2$ 19

2.12.2 $Pd_2L^1_2L^B_2$ 24

2.12.3 $Pd_2L^1_2L^C_2$ 27

2.12.4 $Pd_2L^1_2L^D_2$ 30

2.12.5 $Pd_2L^2_2L^A_2$ 33

2.12.6 $Pd_2L^2_2L^B_2$ 36

2.12.7 $Pd_2L^3_2L^A_2$ 39

2.12.8 $Pd_2L^4_2L^A_2$ 42

2.13 General Synthesis of phosphate ester salts G^1-G^6 45

2.13.1 Dibutylphosphate G^1 45

2.13.2 Dibenzylphosphate G^2 46

2.13.3 Diphenylphosphate G^3 47

2.13.4 Diethylphosphate G^4 48

2.13.5 Di-*p*-toluylphosphate G^5 49
2.13.6 (*S*)-Binaphtylphosphate G^6 50
3 Guest Titration Experiments 51
3.1 Titration of G^1 to $Pd_2L^1{}_2L^A{}_2$ 52
3.2 Titration of G^2 to $Pd_2L^1{}_2L^A{}_2$ 54
3.3 Titration of G^3 to $Pd_2L^1{}_2L^A{}_2$ 55
3.4 Titration of G^4 to $Pd_2L^1{}_2L^A{}_2$ 57
3.5 Titration of G^5 to $Pd_2L^1{}_2L^A{}_2$ 59
3.6 Titration of G^6 to $Pd_2L^1{}_2L^A{}_2$ 60
3.7 Titration of G^3 to $Pd_2L^1{}_2L^B{}_2$ 61
3.8 Titration of G^3 to $Pd_2L^1{}_2L^C{}_2$ 62
3.9 Titration of G^3 to $Pd_2L^1{}_2L^D{}_2$ 63
3.10 Titration of G^3 to $Pd_2L^2{}_2L^A{}_2$ 64
3.11 Titration of G^3 to $Pd_2L^2{}_2L^B{}_2$ 65
3.12 Titration of G^3 to $Pd_2L^3{}_2L^A{}_2$ 66
3.13 Titration of G^3 to $Pd_2L^4{}_2L^A{}_2$ 67
3.14 Titration of G^3 to $Pd_2L^1{}_2L^A{}_2$ in DMF-d_7 68
3.15 Titration of G^3 to $Pd_2L^2{}_2L^A{}_2$ in DMF-d_7 69
4 Guest competition experiments 70
5 Single-crystal X-ray structure analysis 71
5.1 Data collection and refinement details of [$Pd_2L^1{}_2L^A{}_2$], ap136d 73
5.2 Data collection and refinement details of [G^3@$Pd_2L^1{}_2L^A{}_2$], ap214 74
5.3 Data collection and refinement details of [G^5@$Pd_2L^1{}_2L^A{}_2$], ap217_sq 75
6 Modelling of coordination cage structures 76
7 Ion Mobility Measurements 77
7.1 Modeling and Theoretical Collisional Cross Sections calculations (CCS) 82
8 UV/Vis and CD spectroscopy 83
9 Molecular Dynamics Simulations 84
10 Literature 87

1 General Methods

1.1 NMR Spectroscopy

NMR spectroscopic data was measured on the spectrometers Bruker AV 500 Avance NEO, Bruker AV 400 Avance III HD NanoBay, AV 500 Avance III HD, AV 600 Avance III HD, AV 700 Avance III HD and Agilent Technologies DD2 500 MHz. For ^{1}H and ^{13}C NMR spectra, chemical shifts were calibrated to the solvent lock signal. For ^{31}P NMR spectra, 85% H_3PO_4 in H_2O (^{31}P, 0 ppm) was used as external standard. Chemical shifts δ are given in ppm, coupling constants J in Hz. All spectra were recorded in standard 5 mm NMR tubes at 25 °C, if not mentioned otherwise. ^{13}C{^{1}H} NMR spectra were processed using automatic BLP (backwards linear prediction, "cryoproc1d") to optimize the baseline. Due to overlapping signals and low signal-to-noise ratio in the aromatic regions not every ^{13}C signal of the coordination cages could be observed. ^{1}H DOSY NMR spectra were recorded with a *dstebpgp3s* pulse sequence with diffusion delays D20 of 0.08 s and gradient powers P30 of 2500 to 3000 µs.[1,2] T1 analyses of the corresponding signals in the 1D spectra were performed to obtain the diffusion coefficients D using the STEJSKAL-TANNER-Equation.[3,4] Hydrodynamic radii r_H were calculated using the STOKES-EINSTEIN-Equation.[5]

1.2 X-Ray single crystal structure determination

Synchrotron beamline P11@DESY: Single crystal X-ray diffraction data was collected at macromolecular beamline P11, Petra III, DESY (a member of the Helmholtz Association, HGF), Hamburg, Germany. Samples were mounted using the Stäubli TX60L robotic arm. A wavelength of λ = 0.6889 Å was chosen using a liquid N_2 cooled double crystal monochromator. Single crystal X-ray diffraction data was collected at 100(2) K on a single axis goniometer, equipped with an Oxford Cryostream 800 device and an Eiger 2 12M detector.

1.3 Mass spectrometry and ion mobility measurements

Mass spectrometry and trapped ion mobility data were measured on Bruker ESI-timsTOF (electrospray ionization-trapped ion mobility-time of flight) and Bruker compact high-resolution LC mass spectrometers (positive/negative mode). For calibration of the TIMS and TOF devices, Agilent ESI-Low Concentration Tuning Mix was used. All measured and calculated values are given in *m/z*.

1.4 Gaschromatography electron ionization mass spectrometry (GC/EI-MS)

GC/EI-MS analysis was obtained using an Agilent 7890B GC system (column: Agilent HP-5MS, 30 m, 0.25 mm, 0.25 µm) with an Agilent 5977A Mass Selective Detector.

1.5 GPC

Recycling gel permeation chromatography was performed on Japan Analytical Industry NEXT and LaboACE instruments using JAIGEL 1-HH and 2-HH columns, 20 mm x 600 mm, flowrate 7 mL/min.

1.6 UV/Vis spectroscopy

UV vis spectra were recorded on a DAD HP-8453 UV-Vis spectrometer.

1.7 CD spectroscopy

Circular dichroism spectra were recorded on an Applied Photophysis qCD Chirascan CD spectrometer with a temperature-controlled cuvette holder.

2 Experimental Procedures

Where necessary, experiments were performed under argon atmosphere using standard Schlenk techniques. Chemicals and standard solvents were purchased from Sigma Aldrich, Acros Organics, Carl Roth, TCI Europe, VWR, ABCR or other suppliers and used as received, if not mentioned differently. Dry solvents were purchased or purified and dried over absorbent-filled columns on a GS-Systems solvent purification system (SPS). Reactions were monitored with thin layer chromatography (TLC) using silica coated aluminium plates (Merck, silica 60, fluorescence indicator F254, thickness 0.25 mm). For column chromatography, silica (Merck, silica 60, 0.02–0.063 mesh ASTM) was used as the stationary phase, if not mentioned otherwise. The syntheses of the compounds $\mathbf{L}^{\mathbf{A}}$ and $\mathbf{L}^{\mathbf{B}}$ were reported in previous studies.[6,7]

2.1 Synthesis of 8-ethinylisoquinoline 1

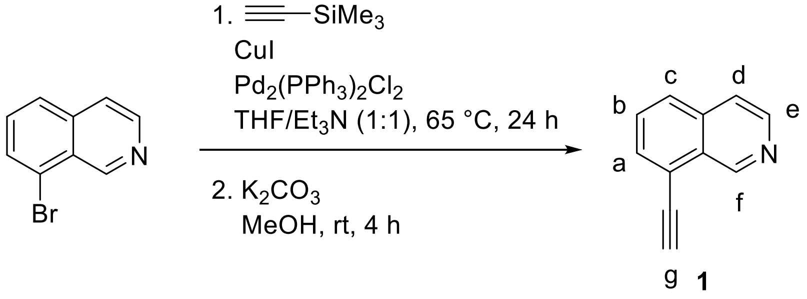

Scheme S1: Synthesis of 8-ethinylisoquinoline **1**.

To a degassed mixture of THF and Et_3N (1:1, 40 mL) 8-bromoisoquinoline (2.00 g, 9.61 mmol, 1 eq), ethinyltrimethylsilane (1.23 g, 12.50 mmol, 1.3 eq), copper(I)-iodide (0.37 g, 1.92 mmol, 0.2 eq) and dichloridobis(triphenylphosphine) palladium(II) (0.67 g, 0.96 mmol, 0.1 eq) were added under argon and heated to 65 °C for 24 h. The reaction mixture was cooled to rt and the solvent was removed. The crude product was purified via column chromatography (pentane/diethyl ether 1:1) to obtain 8-((trimethylsilyl)ethinyl)isoquinoline **1** as an orange oil. The obtained oil was dissolved in MeOH (100 mL) and K_2CO_3 as an excess was added. The reaction mixture was stirred at rt for 4 h. The reaction mixture was dissolved in $CHCl_3$ and carefully washed with water and brine and dried over $MgSO_4$. The solvent was removed in vacuo to obtain 8-ethinylisoquinoline **1** as a dark brown solid (1.39 g, 9.05 mmol, 95%).

^{1}H NMR (700 MHz, 298 K, $CDCl_3$): δ = 9.73 (s, 1H, f), 8.60 (d, ^3J = 5.7, 1H, e), 7.82 (m, 2H, a, c), 7.64 (m, 2H, b, d), 3.55 (s, 1H, g).

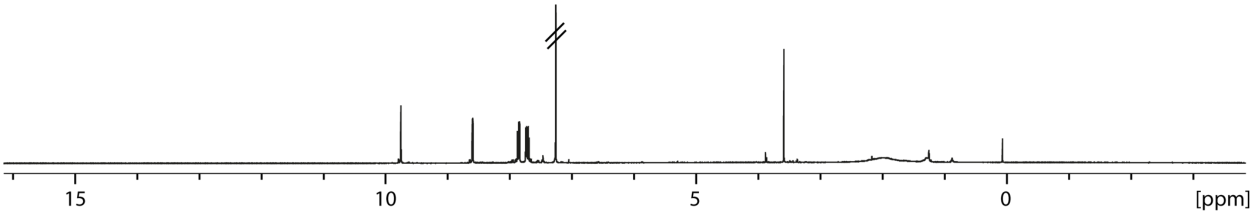

Figure S1: ^{1}H NMR (700 MHz, 298 K, $CDCl_3$) of **1**.

2.2 Synthesis of 2,7-bis(isoquinolin-8-ylethinyl)-9*H*-carbazole L[1]

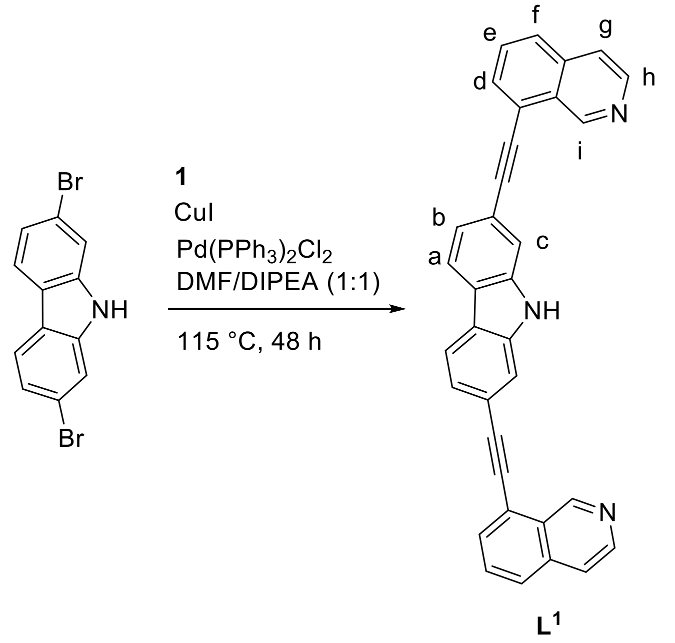

Scheme S2: Synthesis of **L**[1].

To a degassed mixture of DMF and DIPEA (1:1, 15 mL) 2,7-dibromo-9*H*-carbazole (100.00 mg, 0.31 mmol, 1.0 eq), 8-ethinylisoquinoline **1** (141.40 mg, 0.93 mmol, 3.0 eq), copper(I)-iodide (11.27 mg, 0.06 mmol, 0.2 eq) and dichloridobis(triphenylphosphine)palladium(II) (21.60 mg, 0.03 mmol, 0.1 eq) were added under argon in a pressure flask and heated to 115 °C for 48 h. The reaction mixture was allowed to cool down to rt, dissolved in ethyl acetate and was washed with water and brine and dried over $MgSO_4$. The solvent was removed *in vacuo*. The crude product was dissolved in DMF and purified via GPC to obtain **L**[1] as a yellowish-brown solid (35.00 mg, 0.07 mmol, 24%).

^{1}H NMR (500 MHz, 298 K, DMSO-d_6): δ = 11.67 (s, 1H, N*H*), 9.80 (s, 2H, i), 8.65 (d, ^3J = 5.5, 2H, h), 8.31 (d, ^3J = 8.0, 2H, a), 8.06 (d, ^3J = 8.2, 2H, d), 8.01 (d, ^3J = 6.5, 2H, f), 7.95 (d, ^3J = 5.4, 2H, g), 7.94 (s, 2H, c), 7.86 (t, ^3J = 7.4, 2H, e), 7.58 (dd, ^3J = 8.1, ^4J = 1.2, 2H, b).

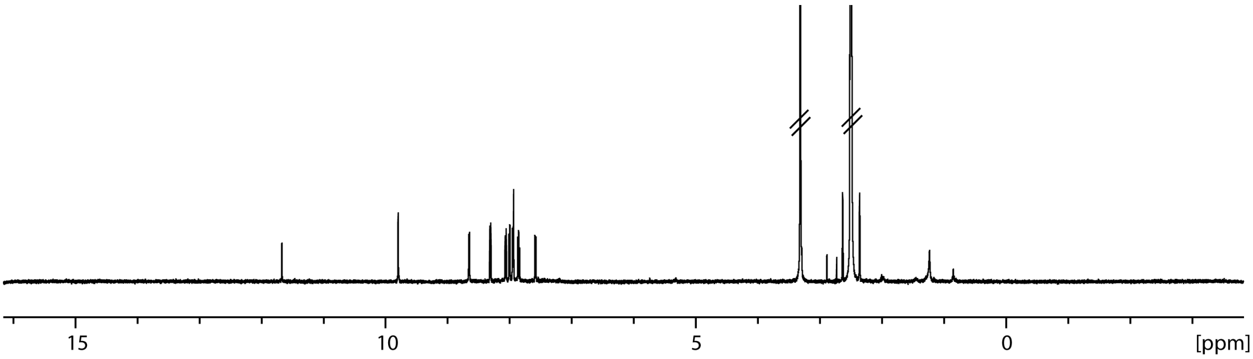

Figure S2: ^{1}H NMR (500 MHz, 298 K, DMSO-d6) of **L**[1].

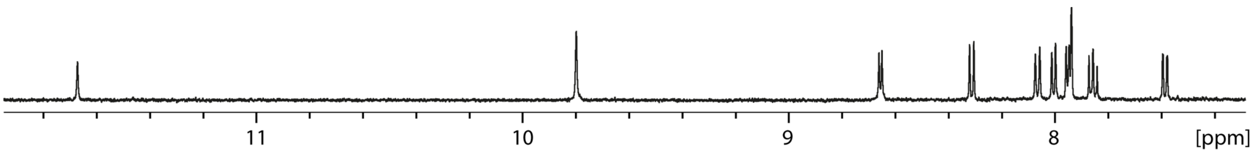

Figure S3: Partial ^{1}H NMR (500 MHz, 298 K, DMSO-d$_6$) of **L^1**.

^{13}C{^{1}H} NMR (151 MHz, 298 K, DMSO-d$_6$): δ = 162.32, 150.14, 143.86, 140.22, 135.45, 131.75, 130.39, 127.48, 127.13, 122.76, 121.21, 120.81, 120.43, 119.10, 114.56, 97.05, 85.36.

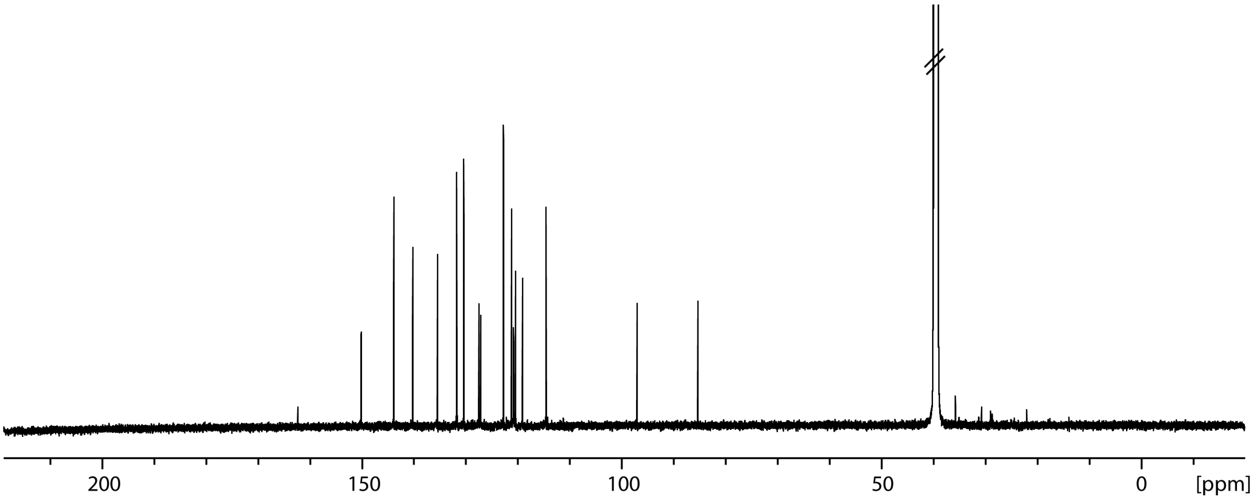

Figure S4: ^{13}C{^{1}H} NMR (151 MHz, 298 K, DMSO-d$_6$) of **L^1**.

HR-ESI-MS (positive mode): measured for: $[C_{34}H_{19}N_3+H]^+$: 411.1623

calculated: 411.1598

2.3 Synthesis of 2,7-dibromo-9-methyl-9*H*-carbazole 2

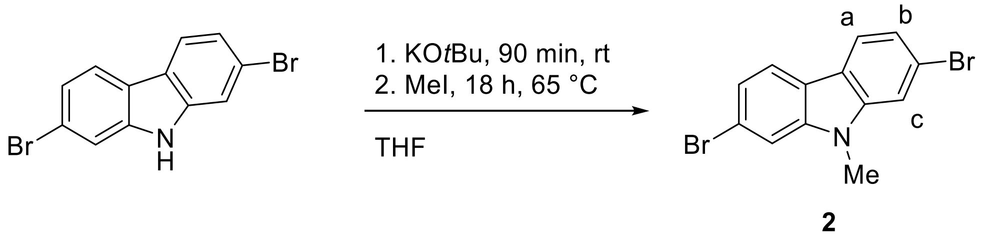

2

Scheme S3: Synthesis of 2,7-dibromo-9-methyl-9*H*-carbazole.

To a suspension of 2,7-dibromo-9*H*-carbazole (500.00 mg, 1.54 mmol, 1.0 eq)) in dry THF (10 mL) KO*t*Bu (345.27 mg, 3.08 mmol, 2.0 eq) was slowly added and the reaction mixture was stirred at rt for 90 min. Then a solution of MeI (240.20 mg, 1.69 mmol, 1.1 eq) in dry THF (3 mL) were added and the reaction mixture was stirred at 65 °C for 18 h. After cooling to rt, the reaction mixture was dissolved in DCM and washed with water and brine and dried over $MgSO_4$. The solvent was removed *in vacuo*. The crude product was purified via column chromatography (pentane/ethyl acetate 9:1) to obtain the desired product as a white solid (140 mg, 0.4 mmol, 27%).

^{1}H NMR (700 MHz, 298 K, $CDCl_3$): δ = 7.89 (d, ^{3}J = 8.2, 2H, a), 7.55 (d, ^{4}J = 1.6, 2H, c), 7.35 (dd, ^{3}J = 8.2, ^{4}J = 1.6, 2H, b), 3.80 (s, 3H, NCH_3).

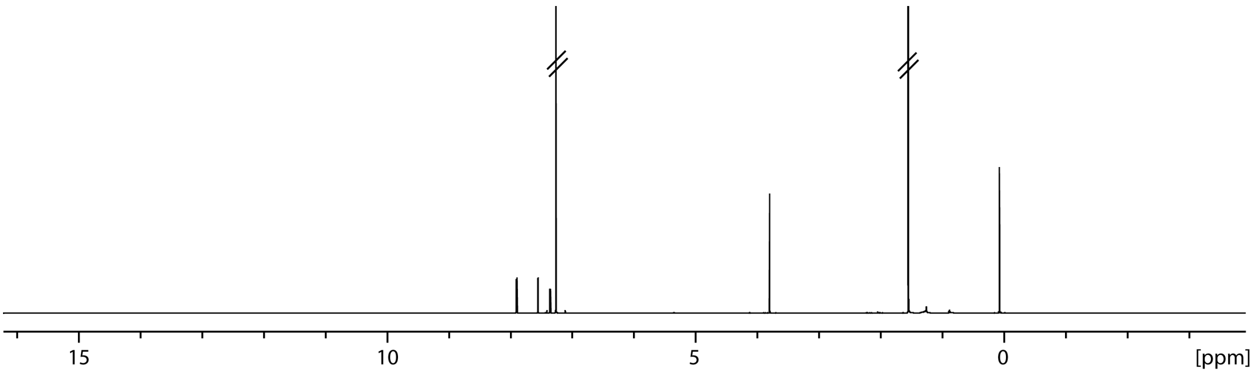

Figure S5: ^{1}H NMR (700 MHz, 298 K, $CDCl_3$) of **2**.

^{13}C NMR (176 MHz, 298 K, $CDCl_3$): δ = 140.92, 121.63, 120.42, 120.23, 118.75, 110.89, 28.30.

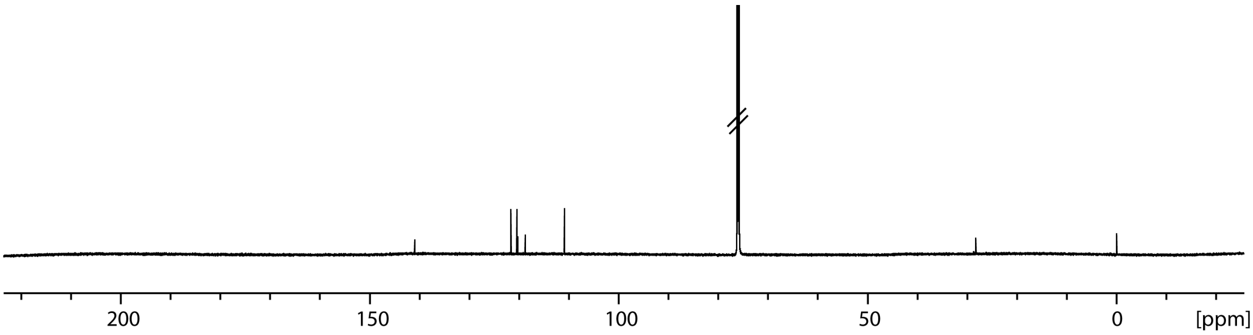

Figure S6: ^{13}C{^{1}H} NMR (176 MHz, 298 K, $CDCl_3$) of **2**.

GC-EI-MS: [80 °C (1 min) – 270 °C (5.5 min), heat rate 40 °C min^{-1}] (70 eV, retention time: 7.58 min): *m*/*z* (%) = 339 (100) [*M*]$^{+\cdot}$, 324 (3) [*M* – Me]$^{+}$, 258 (13) [*M* – Br]$^{+}$, 179 (44) [*M* – 2 Br]$^{+}$, 164 (47) [*M* – Me – 2 Br]$^{+}$.

2.4 Synthesis of 2,7-bis(isoquinolin-8-ylethinyl)-9-methyl-9*H*-carbazole L²

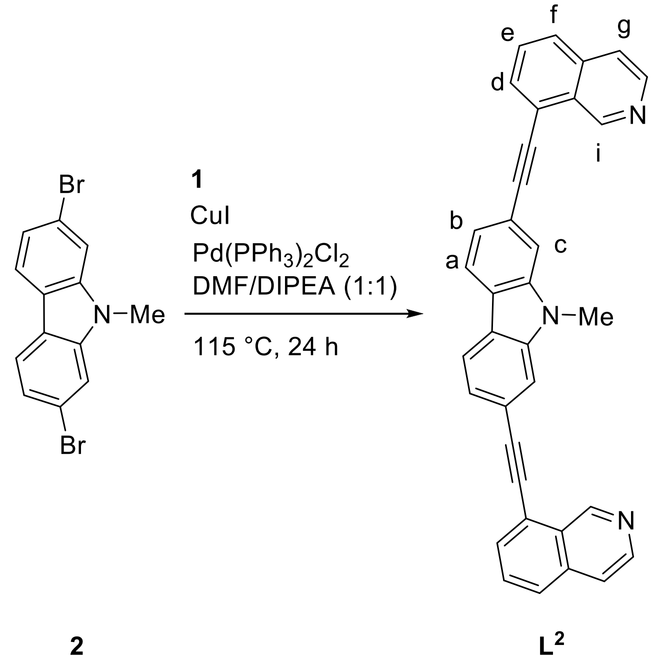

Scheme S4: Synthesis of **L²**.

To a degassed mixture of DMF and DIPEA (1:1, 15 mL) 2,7-dibromo-9-methyl-9*H*-carbazole **2** (100.00 mg, 0.29 mmol, 1.0 eq), 8-ethinylisoquinoline **1** (135.55 mg, 0.87 mmol, 3.0 eq), copper(I)-iodide (11.24 mg, 0.06 mmol, 0.2 eq) and dichloridobis(triphenylphosphine)palladium(II) (20.70 mg, 0.03 mmol, 0.1 eq) were added under argon in a pressure flask and the reaction was heated to 115 °C for 24 h. The reaction mixture was allowed to cool down to rt, dissolved in $CHCl_3$ and was washed with water and brine and dried over $MgSO_4$. The solvent was removed *in vacuo*. The crude product was dissolved in DMF and purified via GPC to obtain **L²** as a yellowish solid (55.00 mg, 0.11 mmol, 39%).

^{1}H NMR (600 MHz, 298 K, DMSO-d$_6$): δ = 9.48 (s, 2H, i), 8.66 (d, ^3J = 5.6, 2H, h), 8.34 (d, ^3J = 8.0, 2H, a), 8.15 (s (br), 2H, d), 8.07 (d, ^3J = 8.3, 2H, f), 8.00 (dd, ^3J = 7.1, ^5J = 0.9, 2H, g), 7.96 (d, ^3J = 5.6, 2H, c), 7.86 (dt, ^3J = 7.2, 2H, e), 7.62 (dd, ^3J = 8.0, ^4J = 1.3, 2H, b), 4.05 (s, 3H, NC*H*$_3$).

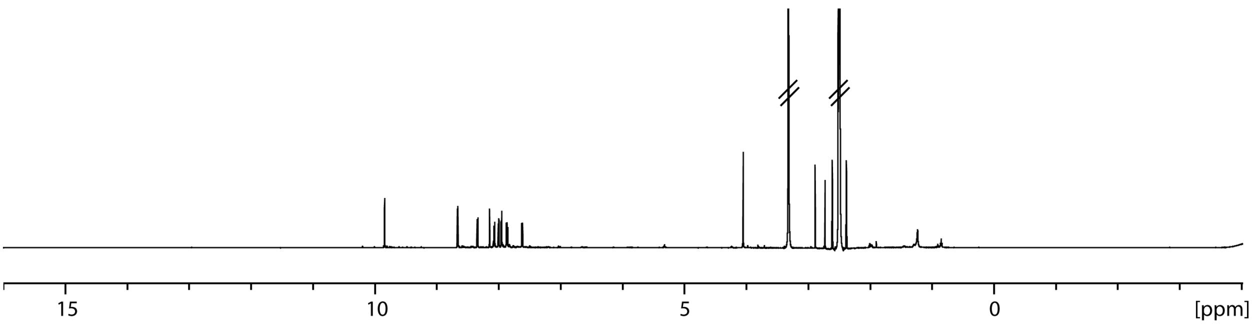

Figure S7: ^{1}H NMR (600 MHz, 298 K, DMSO-d$_6$) of **L²**.

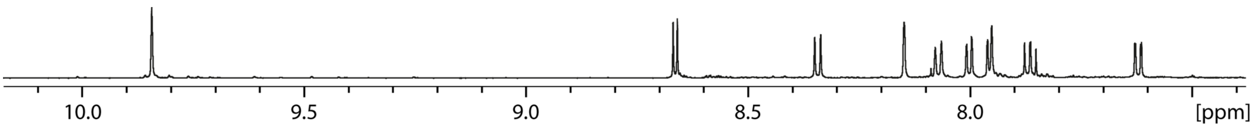

Figure S8: Partial ^{1}H NMR (600 MHz, 298 K, DMSO-d6) of **L²**.

$^{13}C\{^{1}H\}$ NMR (151 MHz, 298 K, DMSO-d6): δ = 150.22, 143.90, 141.20, 135.46, 131.73, 130.39, 127.52, 127.12, 122.85, 122.29, 121.18, 120.79, 120.41, 119.30, 113.08, 97.07, 85.51, 29.38.

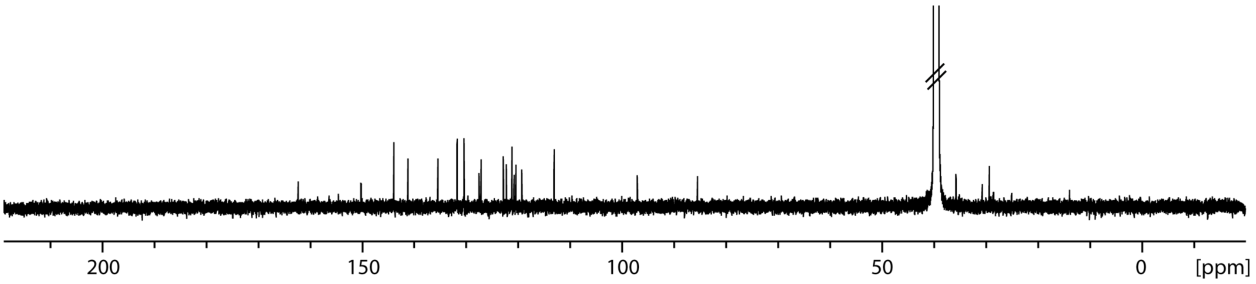

Figure S9: $^{13}C\{^{1}H\}$ NMR (151 MHz, 298 K, DMSO-d6) of **L²**.

HR-ESI-MS (positive mode): measured for: $[C_{35}H_{21}N_3+H]^+$: 484.1763

calculated: 484.1808

2.5 Synthesis of 2,7-dibromo-*N*-methyl-9*H*-carbazole-9-carboxamide 3

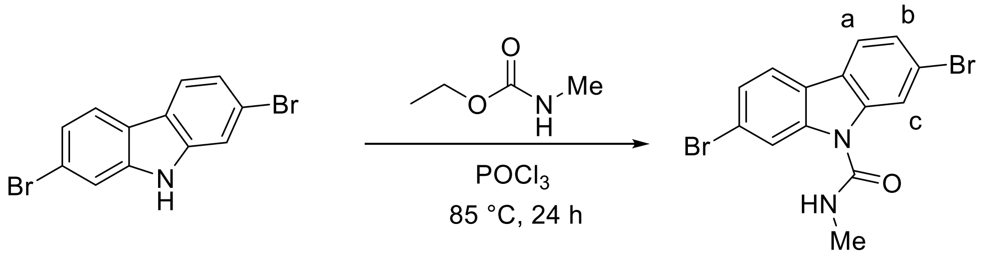

3

Scheme S5: Synthesis of 2,7-dibromo-*N*-methyl-9*H*-carbazole-9-carboxamide **3**.

2,7-dibromo-9*H*-carbazole (1.00 g, 3.08 mmol, 1.0 eq) and ethyl-*N*-methylcarbamate (1.27 g, 12.31 mmol, 4.0 eq) were dissolved in freshly distilled phosphoryl trichloride (20 mL) and heated to 85 °C for 24 h. The reaction mixture was cooled to rt and diluted with ice-cold water. Saturated $NaHCO_3$ solution was added until the mixture was pH-neutral. The reaction mixture was extracted with DCM and the combined organic layers were washed with water and brine and dried over $MgSO_4$. The solvent was removed *in vacuo* and the crude product was purified via column chromatography (pentane/ethyl acetate 9:1) to obtain **3** as a white solid (0.29 g, 0.75 mmol, 24%).

^{1}H NMR (600 MHz, 298 K, DMSO-*d*6): δ = 8.38 (q, ^{3}J = 4.2, 1H, N*H*), 8.18 (d, ^{3}J = 8.3, 2H, a), 8.08 (d, ^{4}J = 1.6, 2H, c), 7.53 (dd, ^{3}J = 8.3, ^{4}J = 1.7, 2H, b), 2.94 (d, ^{3}J = 4.2, 3H, C*H*3).

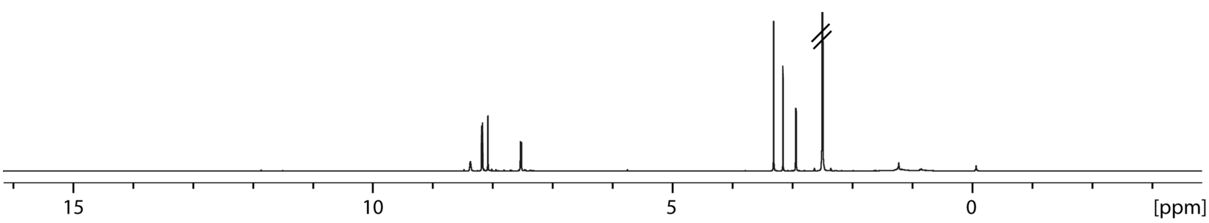

Figure S10: ^{1}H NMR (500 MHz, 298 K, DMSO-d$_6$) of **3**.

^{13}C{^{1}H} NMR (126 MHz, 298 K, DMSO-d_6): δ = 151.9, 138.7, 124.9, 122.3, 122.2, 119.7, 116.4, 28.9.

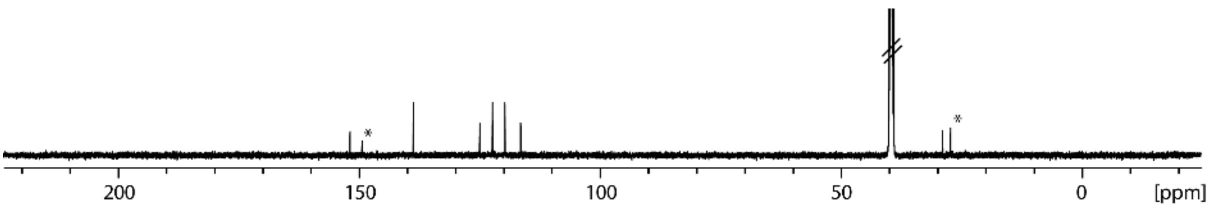

Figure S11: ^{13}C{^{1}H} NMR (126 MHz, 298 K, DMSO-d$_6$) of **3**. Reagent residues are marked with a star *.

HR-ESI-MS (positive mode): measured for: $[C_{14}H_{10}Br_2N_2O+Na]^+$: 404.9023

calculated: 404.9032

2.6 Synthesis of 2,7-bis(isoquinolin-8-ylethinyl)-*N*-methyl-9*H*-carbazole-9-carboxamide

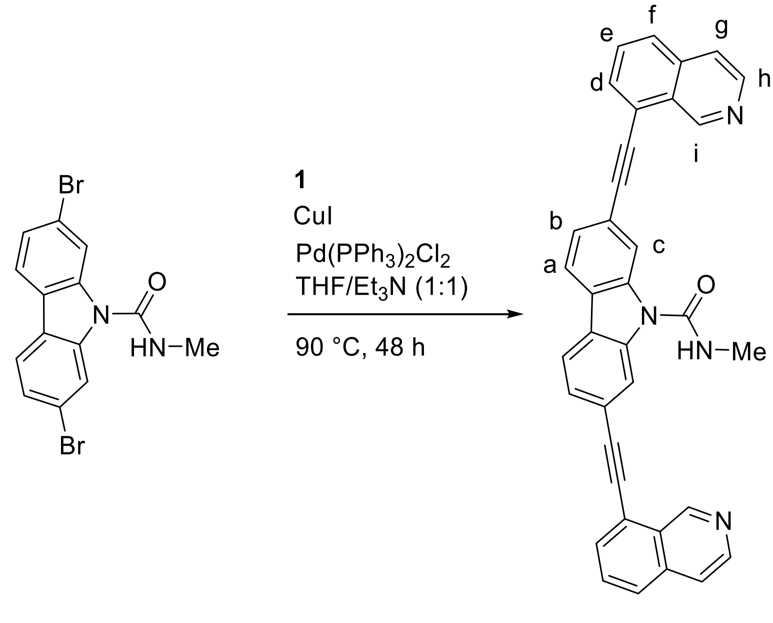

3 **L^3**

Scheme S6: Synthesis of 2,7-bis(isoquinolin-8-ylethinyl)-*N*-methyl-9*H*-carbazole-9-carboxamide **L^3**.

To a degassed mixture of dry THF and Et_3N (1:1, 20 mL) **3** (250.00 mg, 0.65 mmol, 1.0 eq), **1** (250.59 mg, 1.64 mmol, 2.5 eq), copper(I)-iodide (24.92 mg, 0.13 mmol, 0.2 eq) and dichloridobis(triphenylphosphine)palladium(II) (45.93 mg, 0.07 mmol, 0.1 eq) were added under argon

in a pressure flask and heated to 90 °C for 48 h. The reaction mixture was allowed to cool down to rt, dissolved in EtOAc and washed with water and brine and dried over $MgSO_4$. The solvent was removed *in vacuo*. The crude product was purified via column chromatography (EtOAc 100%) and GPC ($CHCl_3$) to obtain **L**^{3} as an orange solid (20 mg, 0.04 mmol, 6%).

^{1}**H NMR** (600 MHz, 298 K, DMSO-d$_6$): δ = 9.81 (s (br), 2H, i), 8.67 (s (br), 2H, h), 8.49 (q, ^3J = 4.4, 1H, N*H*), 8.39 (d, ^3J = 8.0, 2H, a), 8.30 (d, ^4J = 0.5, 2H, c), 8.08 (d, ^3J = 8.3, 2H, d), 8.03 (dd, ^3J = 7.1, ^4J = 0.9 Hz, 2H, f), 7.96 (d, ^3J = 5.3 Hz, 2H, g), 7.86 (m, 2H, e), 7.78 (dd, ^3J = 8.0 Hz, ^4J = 1.3 Hz, 2H, b), 3.04 (d, ^3J = 4.4 Hz, 3H, C*H*$_3$).

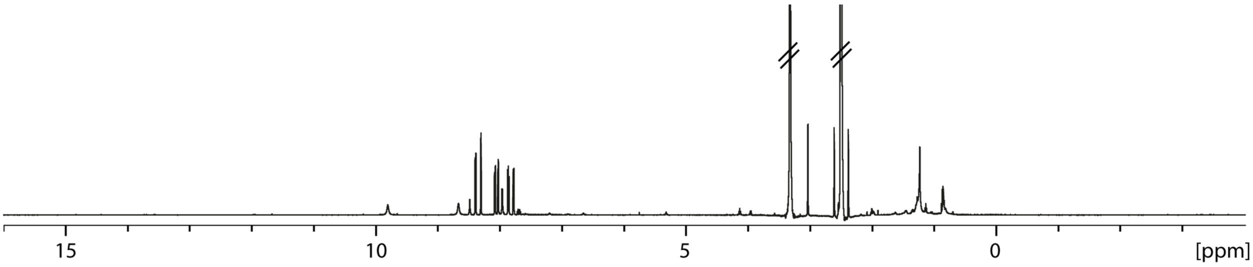

Figure S12: ^{1}H NMR (600 MHz, 298 K, DMSO-d$_6$) of **L**^{3}.

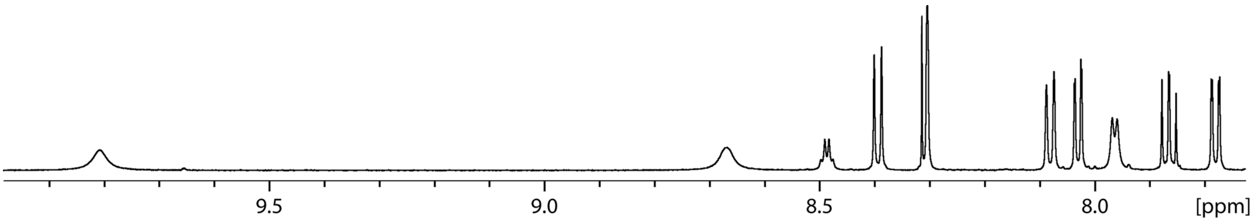

Figure S13: Partial ^{1}H NMR (600 MHz, 298 K, DMSO-d$_6$) of **L**^{3}.

^{13}**C{**^{1}**H} NMR** (151 MHz, 298 K, DMSO-d$_6$): δ = 152.62, 150.58, 144.38, 138.80, 135.90, 132.43, 130.85, 129.14, 128.19, 126.11, 124.49, 121.78, 120.71, 120.62, 117.37, 96.92, 86.48, 79.65, 27.90.

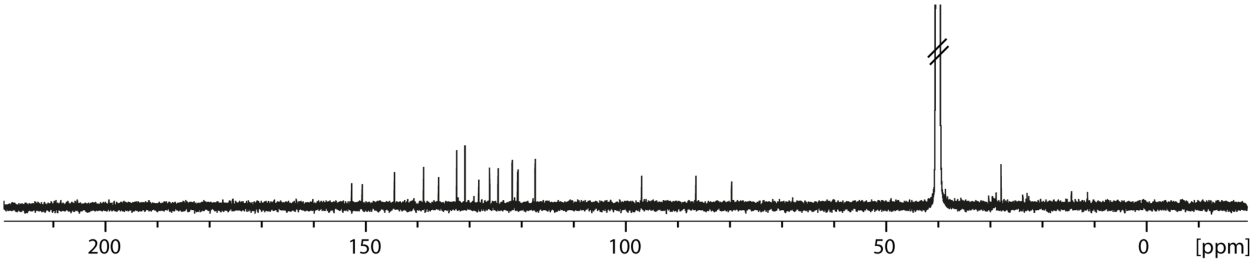

Figure S14: ^{13}C{^{1}H} NMR (151 MHz, 298 K, DMSO-d$_6$) of **L**^{3}.

HR-ESI-MS (positive mode): measured for: $[C_{36}H_{22}N_4O+H]^+$: 527.1859

calculated: 527.1866

2.7 Synthesis of 2,7-dibromo-*N*-ethyl-9*H*-carbazole-9-carboxamide 4

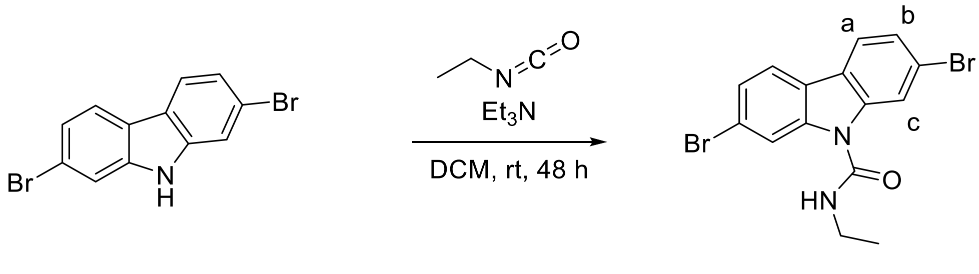

4

Scheme S7: Synthesis of 2,7-dibromo-*N*-ethyl-9*H*-carbazole-9-carboxamide **4**.

2,7-dibromo-9*H*-carbazole (250.00 mg, 0.77 mmol, 1.0 eq) was dissolved in dry DCM (20 mL). Ethyl isocyanate (82.01 mg, 1.15 mmol, 1.5 eq) and Et_3N (116.76 mg, 1.15 mmol, 1.5 eq) were added and the reaction mixture was stirred at rt for 48 h. The reaction mixture was diluted with water (30 mL) and extracted with DCM. The combined organic phases were washed with water and brine and dried over $MgSO_4$. The solvent was removed *in vacuo*. The crude product was purified via column chromatography (pentane/EtOAc, 9:1) and GPC ($CHCl_3$) to obtain **4** as a white solid (94.00 mg, 0.24 mmol, 31%).

^{1}H NMR (500 MHz, 298 K, DMSO-d_6): δ = 8.51 (t, ^3J = 5.3, 1H, N*H*), 8.18 (d, ^3J = 8.3, 2H, a), 8.06 (d, ^4J = 1.6, 2H, c), 7.53 (dd, ^3J = 8.3, ^4J = 1.6, 2H, b), 3.41 (m, 2H, CH_2), 1.25 (t, ^3J = 7.2, 3H, CH_3).

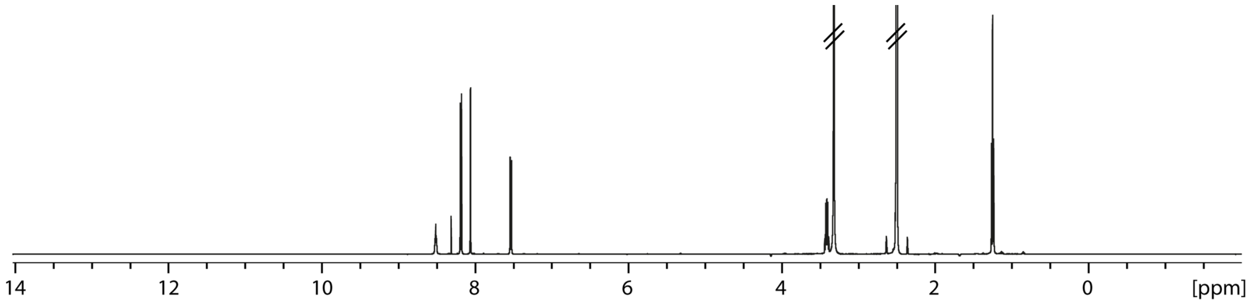

Figure S15: ^{1}H NMR (500 MHz, 298 K, DMSO-d$_6$) of **4**.

^{13}C{^{1}H} NMR (126 MHz, 298 K, DMSO-d_6): δ = 151.1, 138.8, 124.9, 122.3, 122.2, 119.7, 116.4, 35.4, 14.6.

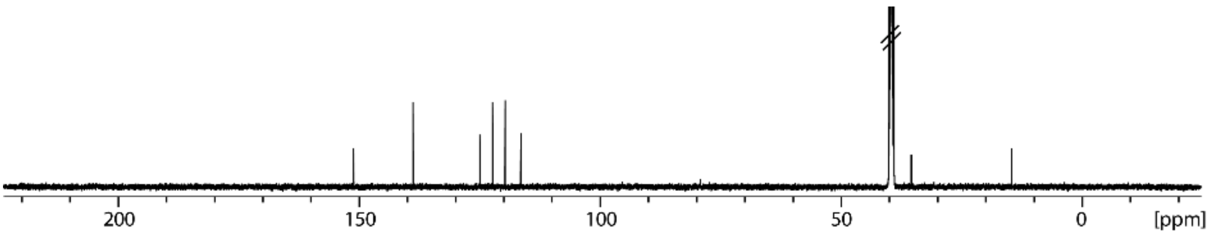

Figure S16: ^{13}C{^{1}H} NMR (126 MHz, 298 K, DMSO-d$_6$) of **4**.

HR-ESI-MS (positive mode): measured for: $[C_{15}H_{12}Br_2N_2O+Na]^+$: 418.9186

calculated: 418.9189

2.8 Synthesis of 2,7-bis(isoquinolin-8-ylethinyl)-*N*-ethyl-9*H*-carbazole-9-carboxamide L[4]

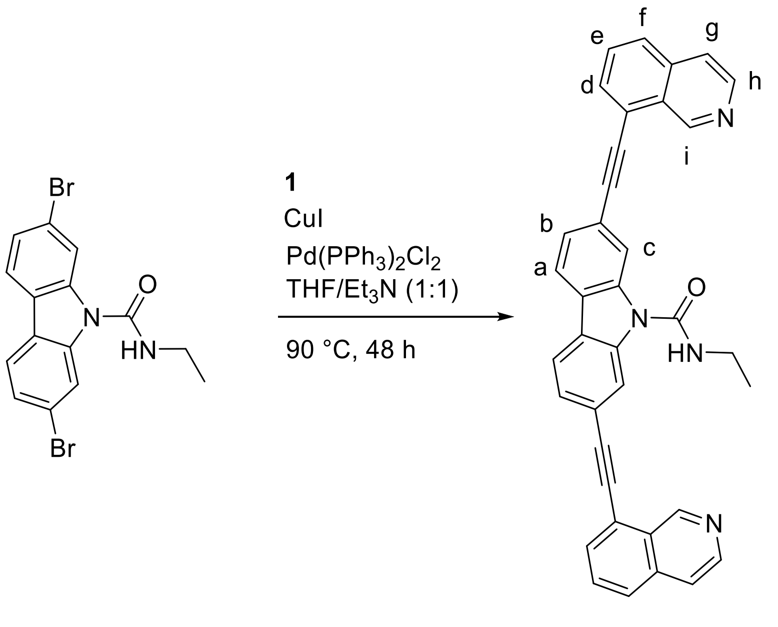

4 **L[3]**

Scheme S8: Synthesis of 2,7-bis(isoquinolin-8-ylethinyl)-*N*-ethyl-9*H*-carbazole-9-carboxamide **L[4]**.

To a degassed mixture of THF and Et_3N (1:1, 10 mL) **4** (94.00 mg, 0.24 mmol, 1.0 eq), **1** (90.89 mg, 0.59 mmol, 2.5 eq), copper(I)-iodide (13.6 mg, 0.07 mmol, 0.3 eq) and dichloridobis(triphenylphosphine)palladium(II) (25.03 mg, 0.04 mmol, 0.15 eq) were added under argon in a pressure flask and heated to 90 °C for 48 h. The reaction mixture was allowed to cool down to rt, dissolved in $CHCl_3$ and washed with water and brine and dried over $MgSO_4$. The solvent was removed *in vacuo*. The crude product was purified via column chromatography ($CHCl_3$/MeOH, 10:1) and GPC ($CHCl_3$) to obtain **L[3]** as an orange solid (15.00 mg, 0.03 mmol, 12%).

^{1}H NMR (600 MHz, 298 K, DMSO-d_6): δ = 9.80 (s, 2H, i), 8.66 (d, ^3J = 5.6, 2H, h), 8.63 (t, ^3J = 5.4, 1H, N*H*), 8.40 (d, ^3J = 8.0, 2H, a), 8.28 (d, ^4J = 0.5, 2H, c), 8.08 (d, ^3J = 8.3, 2H, f), 8.03 (dd, ^3J = 7.1, ^4J = 0.9, 2H, d), 7.96 (dd, ^3J = 5.6, ^4J = 0.5, 2H, g), 7.86 (m, 2H, e), 7.78 (dd, ^3J = 8.1, ^4J = 1.7, 2H, b), 3.51 (m, 2H, CH_2), 1.34 (t, ^3J = 7.2, 3H, CH_3).

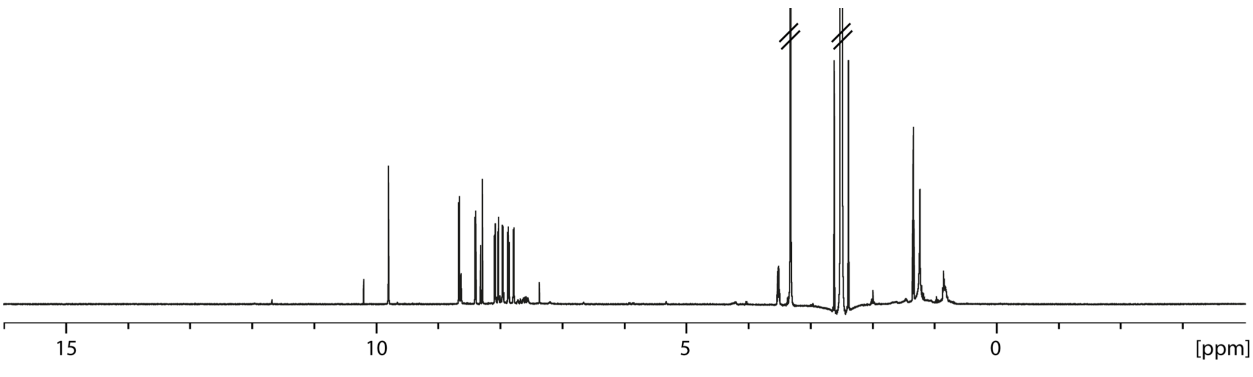

Figure S17: ^{1}H NMR (600 MHz, 298 K, DMSO-d$_6$) of **L[4]**.

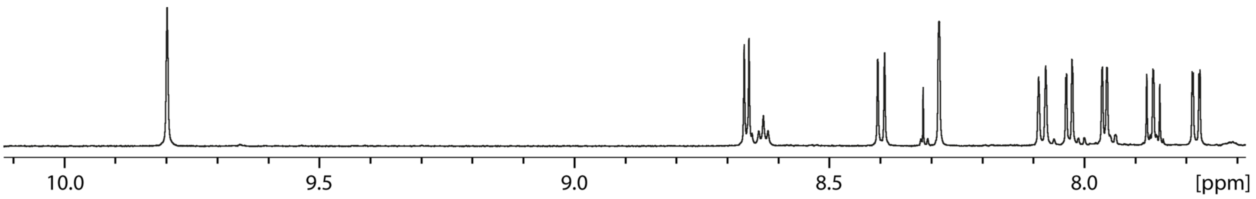

Figure S18: Partial ^{1}H NMR (600 MHz, 298 K, DMSO-d$_6$) of **L^4**.

^{13}C{^{1}H} NMR (151 MHz, 298 K, DMSO-d$_6$, lb = 2 Hz): δ = 151.4, 150.1, 143.9, 138.4, 135.4, 131.9, 130.4, 127.7, 127.1, 125.6, 124.0, 121.3, 120.8, 120.2, 120.1, 116.9, 96.5, 86.0, 35.5, 14.8.

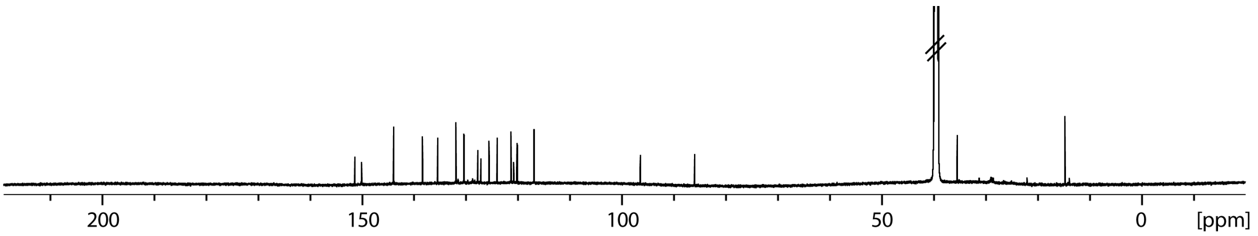

Figure S19: ^{13}C{^{1}H} NMR (151 MHz, 298 K, DMSO-d$_6$, lb = 1 Hz) of **L^4**.

HR-ESI-MS (positive mode): measured for: $[C_{37}H_{24}N_4O+H]^+$: 541.2018

calculated: 541.2023

2.9 Synthesis of 3,6-dibromo-9-hexyl-9*H*-carbazole 5

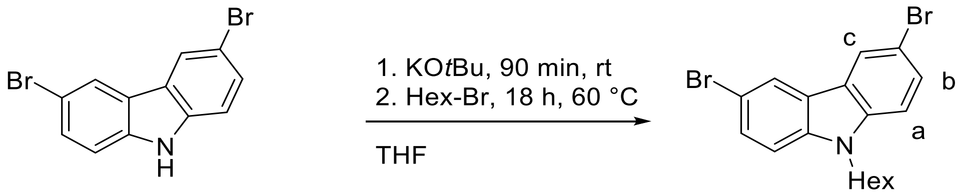

5

Scheme 9: Synthesis of 3,6-dibromo-hexyl-9*H*-carbazole **5**.

3,6-dibromo-9*H*-carbazole (1.00 g, 3.08 mmol, 1.0 eq) and KO*t*Bu (0.38 g, 3.38 mmol, 1.1 eq) were suspended in dry THF (50 mL) and stirred at rt for 90 min. After adding 1-bromo hexane (1.02 g, 6.15 mmol, 2.0 eq) dropwise to the reaction mixture, it was heated to 60 °C for 18 h. The reaction mixture was cooled to rt, filtered over celite and washed with EtOAc. The solvent was removed *in vacuo*. The crude product was purified via column chromatography (pentane/EtOAc, 20:1) to obtain **5** as a yellow solid (1.04 g, 2.53 mmol, 82%).

^{1}H NMR (500 MHz, 298 K, CDCl$_3$): δ = 8.13 (s, 2H, c), 7.54 (dd, ^3J = 8.8, ^4J = 1.7, 2H, b), 7.26 (d, ^3J = 8.8, 2H, a), 4.23 (t, ^3J = 7.2, 2H, NCH_2), 1.81 (quint, ^3J = 7.2, 2H, NCH$_2$CH_2), 1.30 (m, 6H), 0.85 (t, ^3J = 6.9, 3H, CH_3).

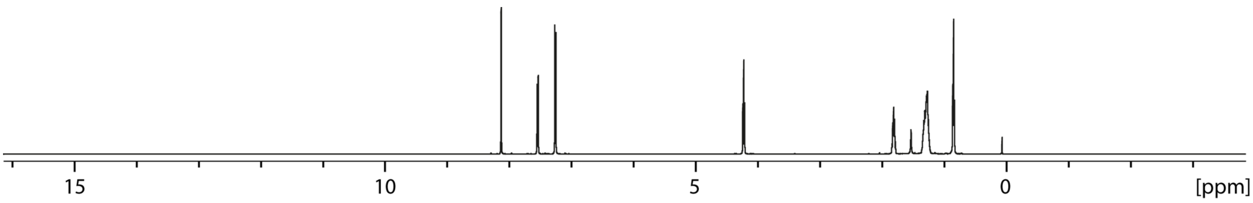

Figure S20: ^{1}H NMR (500 MHz, 298 K, $CDCl_3$) of **5**.

$^{13}C\{^{1}H\}$ NMR (126 MHz, 298 K, $CDCl_3$): δ = 139.5, 129.1, 123.6, 123.4, 112.1, 110.5, 43.5, 31.6, 29.0, 27.0, 22.6, 14.1.

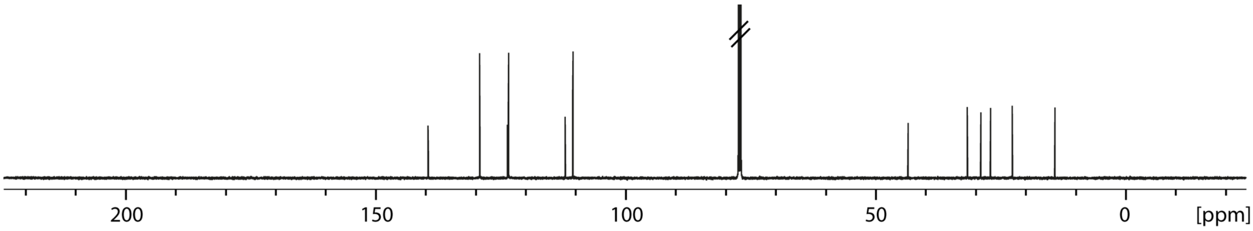

Figure S21: $^{13}C\{^{1}H\}$ NMR (126 MHz, 298 K, $CDCl_3$) of **5**.

HR-ESI-MS (positive mode): measured for: $[C_{18}H_{19}Br_2N_2+K]^+$: 447.9480

calculated: 447.9496

2.10 Synthesis of 9-hexyl-3,6-di(pyridine-4-yl)-9H-carbazole L^{C}

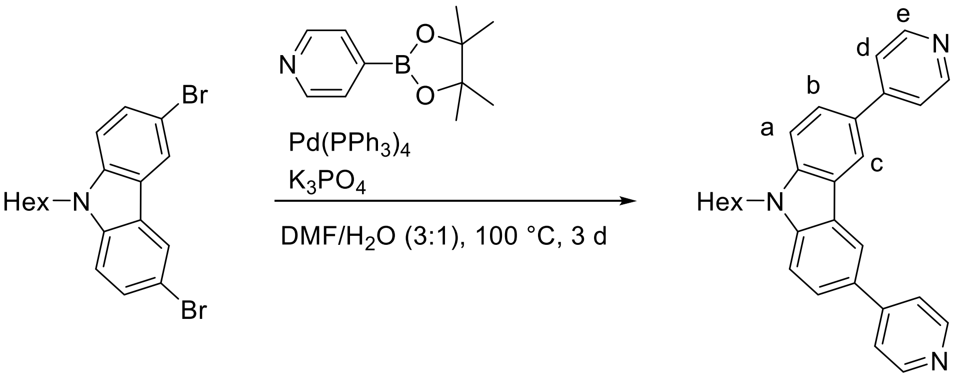

5 **L^{C}**

Scheme 10: Synthesis of **L^{C}**.

To a degassed mixture of DMF and water (3:1, 30 mL) **5** (100.00 mg, 0.24 mmol, 1.0 eq), 4-(4,4,5,5-tetramethyl-1,3,2-dioxaborolan-2-yl)pyridine (300.71 mg, 1.47 mmol, 6.0 eq), tetrakis(triphenylphosphine)palladium(0) (28.24 mg, 0.02 mmol, 0.1 eq) and potassium phosphate (155.63 mg, 0.73 mmol, 3.0 eq) were added under argon in a pressure flask and heated to 100 °C for 3 d. After the reaction mixture was cooled down to rt, $CHCl_3$ (90 mL) was added. The mixture was consecutively washed with water, brine and saturated ammonium chloride solution and dried over $MgSO_4$. The solvent was removed *in vacuo*. The crude product was purified via column chromatography (pentane/acetone, 1:1) and GPC ($CHCl_3$) to yield **L^{C}** as a yellow solid (60.00 mg, 0.15 mmol, 61%).

^{1}H NMR (500 MHz, 298 K, $CDCl_3$): δ = 8.75 (s (br), 4H, e), 8.46 (d, ^{4}J = 1.3, 2H, c), 7.81 (dd, ^{3}J = 8.5, ^{4}J = 1.6, 2H, b), 7.70 (s (br), 4H, d), 7.54 (d, ^{3}J = 8.5, 2H, a), 4.37 (t, ^{3}J = 7.2, 2H, NCH_2), 1.93 (quint., ^{3}J = 7.2, 2H, NCH_2CH_2), 1.41 (m, 2H, $NCH_2CH_2CH_2$), 1.32 (m, 4H), 0.88 (t, ^{3}J = 7.1, 3H, CH_3).

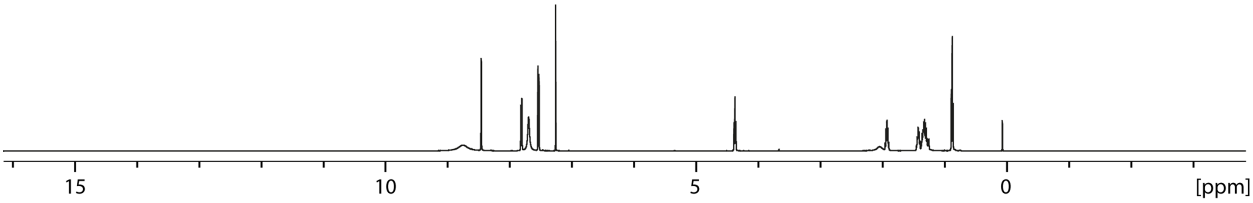

Figure 22: ^{1}H NMR (500 MHz, 298 K, $CDCl_3$) of **L^{C}**.

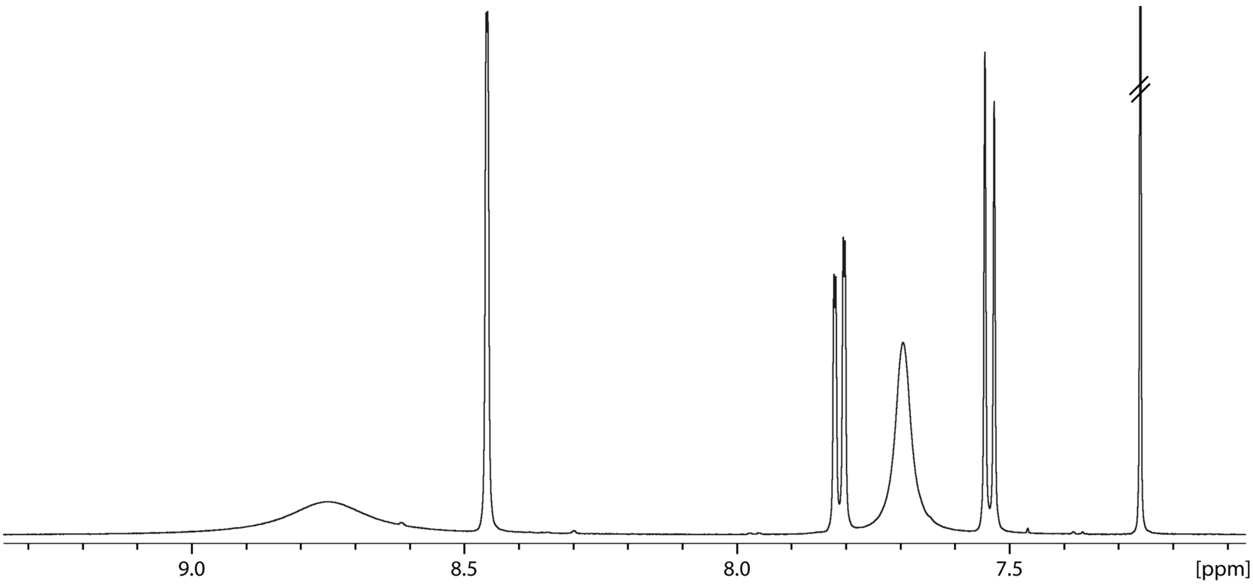

Figure 23: Partial ^{1}H NMR (500 MHz, 298 K, $CDCl_3$) of **L^{C}**.

^{13}C NMR (126 MHz, 298 K, $CDCl_3$): δ = 150.1, 149.2, 141.6, 129.5, 125.4, 123.7, 122.1, 119.3, 109.8, 43.7, 31.7, 29.1, 27.1, 22.7, 14.1.

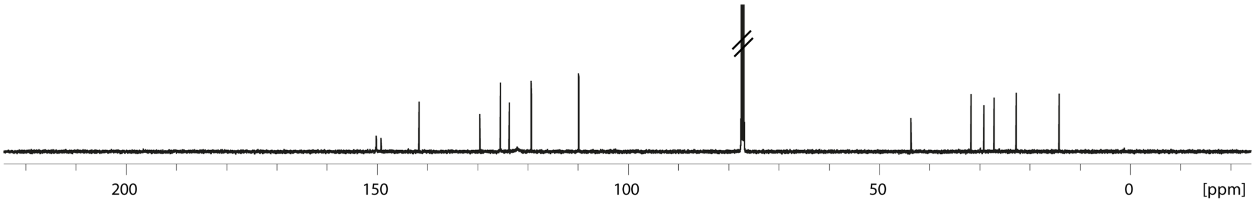

Figure S24: ^{13}C{^{1}H} NMR (126 MHz, 298 K, $CDCl_3$) of **L^{C}**.

HR-ESI-MS (positive mode): measured for: $[C_{28}H_{27}N_3+H]^+$: 406.2293

calculated: 406.2278

2.11 Synthesis of 3,6-di(pyridine-4-yl)-9*H*-fluoren-9-one L^D

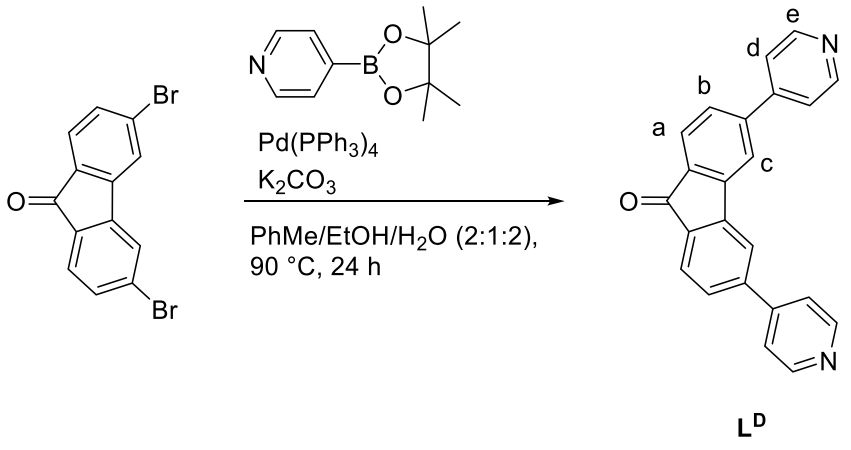

Scheme 11: Synthesis of **L^D**.

To a degassed mixture of toluene, ethanol and water (2:1:2, 25 mL), 2,6-dibromo-9H-fluoren-9-one (200.00 mg, 0.59 mmol, 1.0 eq), 4-(4,4,5,5-tetramethyl-1,3,2-dioxaborolan-2-yl)pyridine (485.36 mg, 2.37 mmol, 4.0 eq), tetrakis(triphenylphosphine)palladium(0) (68.38 mg, 0.06 mmol, 0.1 eq) and potassium carbonate (245.34 mg, 1.78 mmol, 3.0 eq) were added under argon in a pressure flask and heated to 90 °C for 24 h. The reaction mixture was allowed to cool down to rt and extracted with DCM. The combined organic layers were washed with water and brine and dried over $MgSO_4$. The solvent was removed *in vacuo*. The crude product was purified via column chromatography (DCM/MeOH, 10:1) to obtain **L^D** as a yellow solid (401.00 mg, 1.20 mmol, 81%).

^{1}H NMR (700 MHz, 298 K, DMSO-d$_6$): δ = 8.75 (d, ^3J = 4.54, 4H, e), 8.50 (s, 2H, c), 7.87 (m, 6H, b, d), 7.78 (d, ^3J = 7.60, 2H, a).

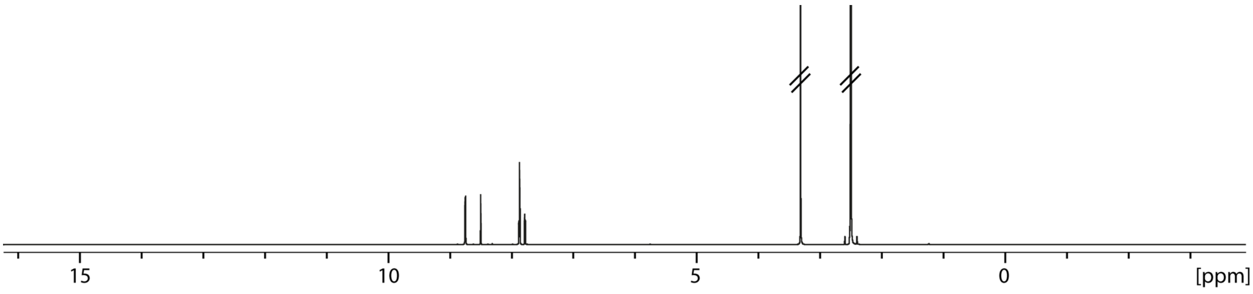

Figure S25: ^{1}H NMR (700 MHz, 298 K, DMSO-d$_6$) of **L^D**.

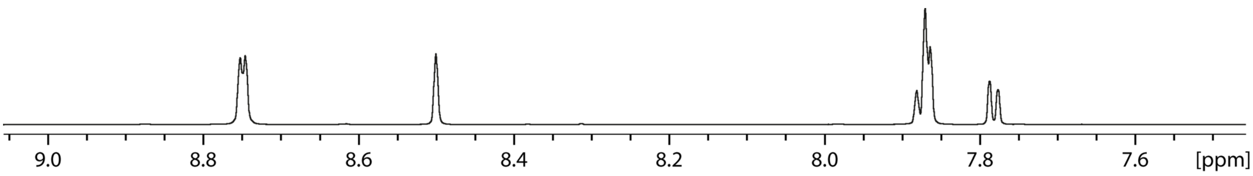

Figure S26: Partial ^{1}H NMR (700 MHz, 298 K, DMSO-d$_6$) of **L^D**.

^{13}C{^{1}H} NMR (176 MHz, 298 K, DMSO-d$_6$): δ = 192.0, 150.4, 145.8, 144.5, 143.9, 133.9, 128.3, 124.6, 121.4, 120.3.

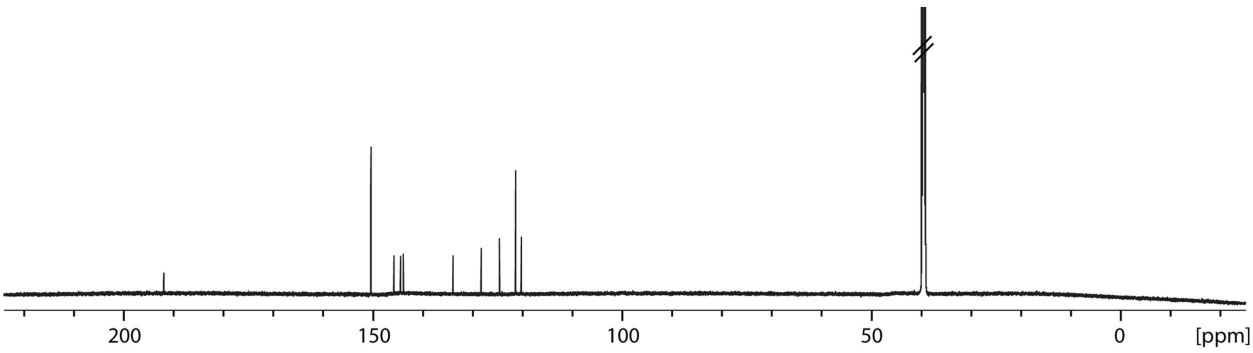

Figure S27: $^{13}C\{^1H\}$ NMR (176 MHz, 298 K, DMSO-d$_6$) of $\mathbf{L^D}$.

HR-ESI-MS (positive mode): measured for: $[C_{23}H_{14}N_2O+H]^+$: 335.1199

calculated: 335.1184

2.12 General synthesis of heteroleptic coordination cages

To a 1:1 mixture of the corresponding ligands (270 µL each, 2.8 mM, DMSO-d$_6$) a solution of $[Pd(MeCN)_4](BF_4)_2$ (60 µL, 15.0 mM, 0.55 eq, DMSO-d$_6$) was added in a NMR tube and shaken for 5 min at rt. The coordination cages were formed in a quantitative yield within minutes. Cage formation was also observed when using ligands and Pd-salt solution in DMF-d$_7$.

2.12.1 $Pd_2L^1_2L^A_2$

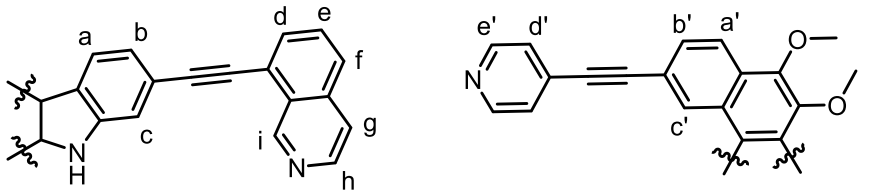

Scheme S12: Ligand Assignment for $Pd_2\mathbf{L}^1{}_2\mathbf{L}^A{}_2$.

^{1}H NMR (500 MHz, 298 K, DMSO-d$_6$): δ = 11.76 (s, 2H, N*H*), 9.90 (s, 4H, i), 9.54 (d, ^3J = 6.5, 4H, h), 9.43 (d, ^3J = 6.7, 8H, e'), 9.24 (s, 4H, c'), 8.47 (d, ^3J = 8.0, 4H, a), 8.41 (d, ^3J = 6.5, 4H, g), 8.23 (d, ^3J = 8.6, 4H, a'), 8.19 (d, ^3J = 8.3, 4H, d), 8.10 (m, 4H, f), 8.06 (m, 8H, d'), 8.02 (m, 4H, e), 7.99 (s, 4H, c), 7.85 (dd, ^3J = 8.5, ^5J = 1.0, 4H, b'), 7.47 (dd, ^3J = 7.9, ^4J = 1.2, 4H, b), 4.01 (s, 12H, OC*H*$_3$).

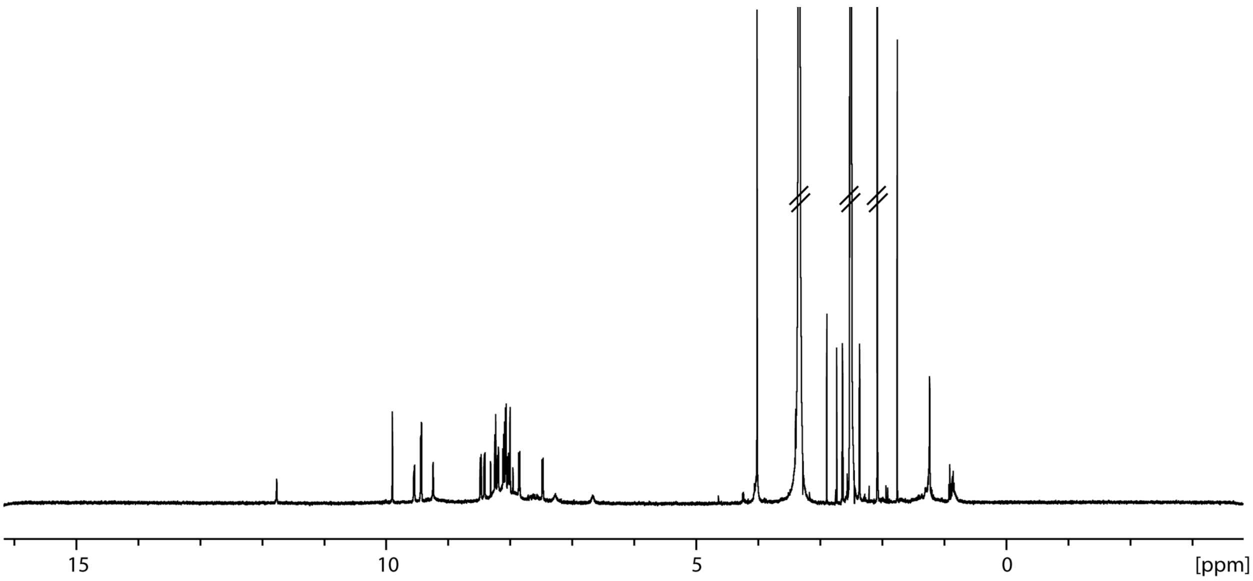

Figure S28: ^{1}H NMR (500 MHz, 298 K, DMSO-d$_6$) of Pd$_2$**L^1**$_2$**L^A**$_2$.

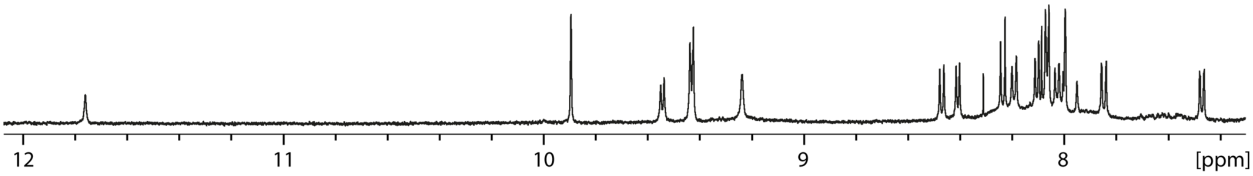

Figure S29: Partial ^{1}H NMR (500 MHz, 298 K, DMSO-d$_6$) of Pd$_2$**L^1**$_2$**L^A**$_2$.

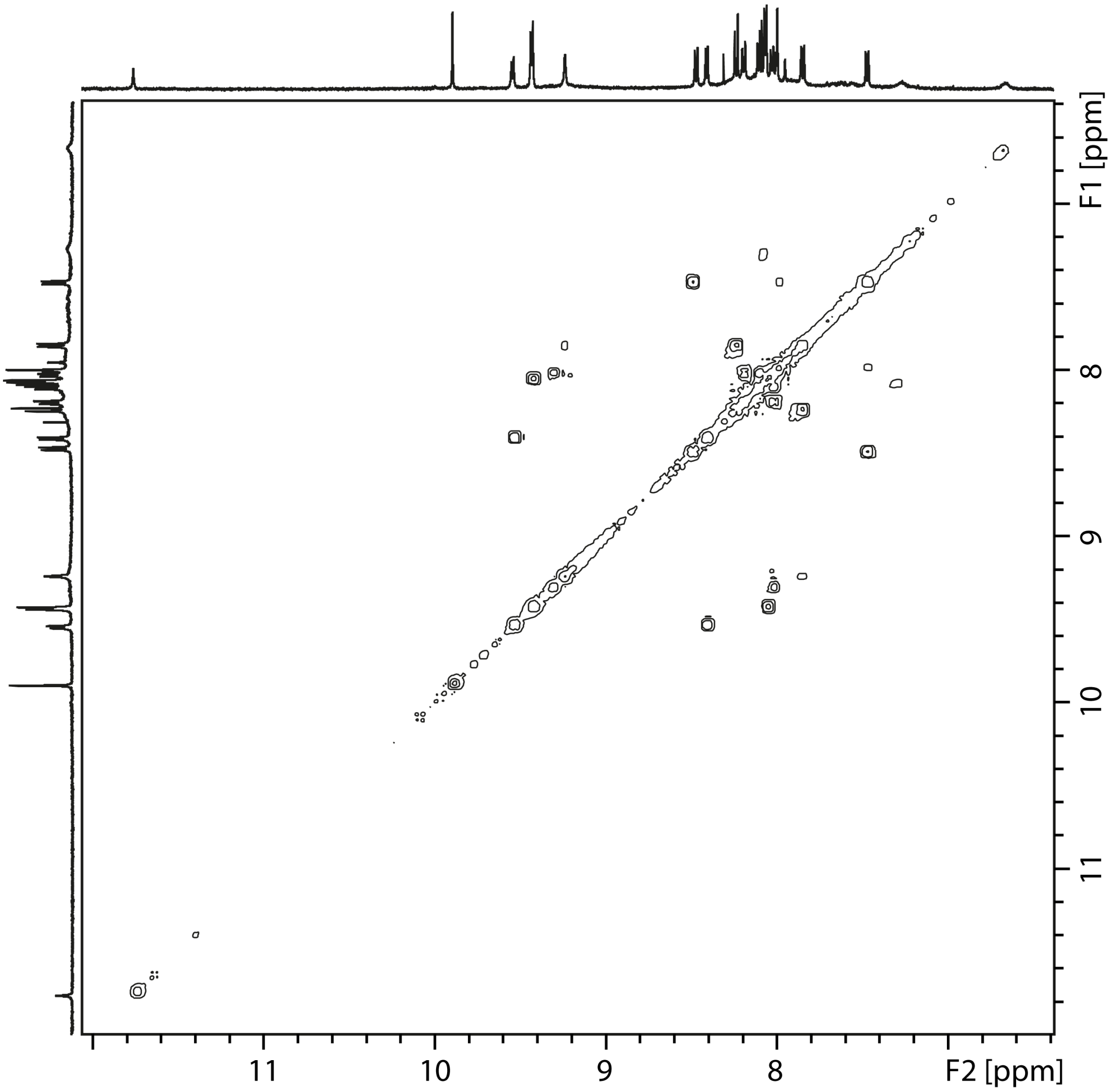

Figure S30: Partial $^{1}H-^{1}H$ COSY NMR (500 MHz, 298 K, DMSO-d_6) of $Pd_2\mathbf{L^1}_2\mathbf{L^A}_2$.

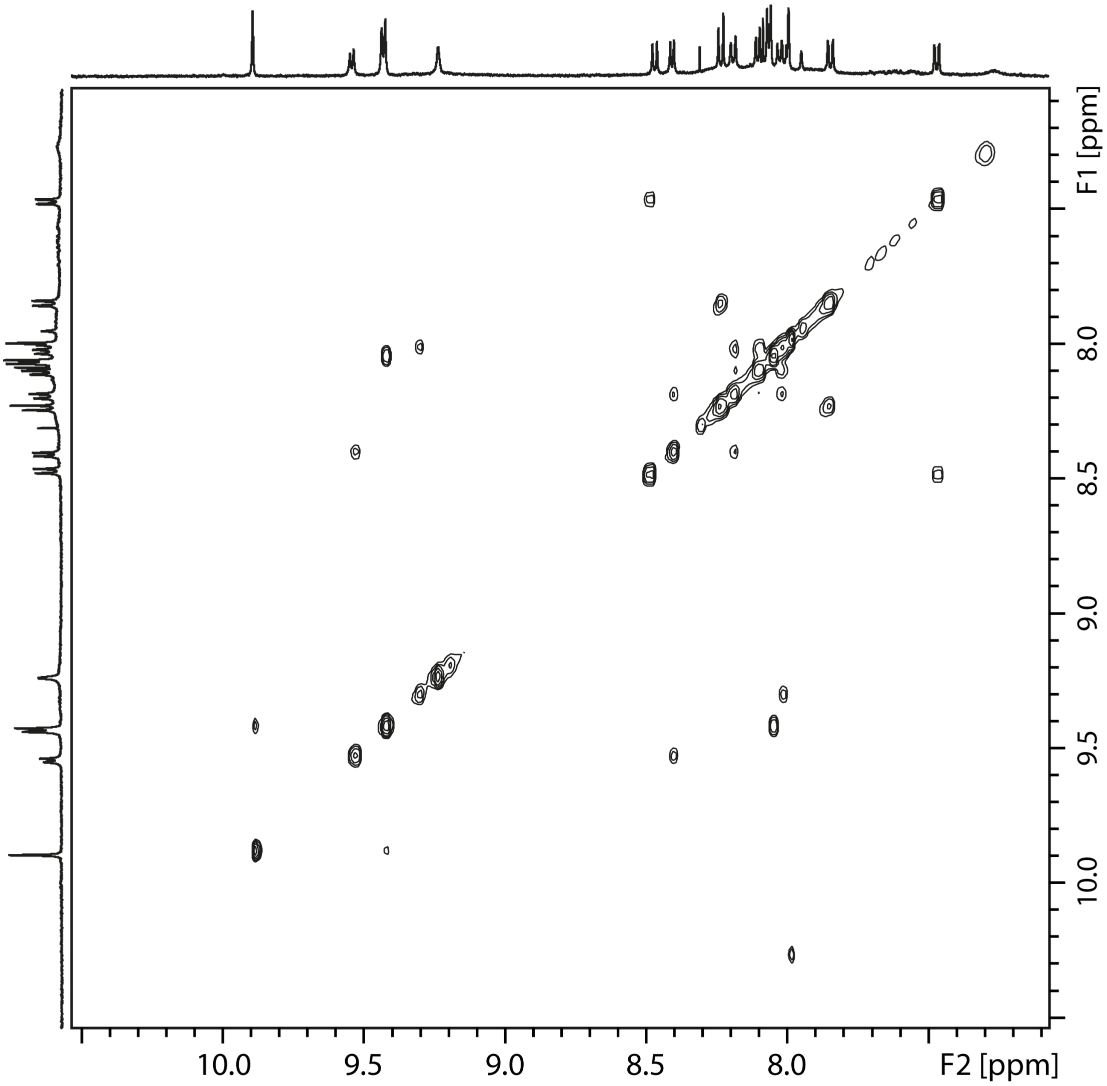

Figure S31: Partial ^{1}H−^{1}H NOESY NMR (500 MHz, 298 K, DMSO-d_6) of Pd_2**L**$^{\mathbf{1}}_2$**L**$^{\mathbf{A}}_2$.

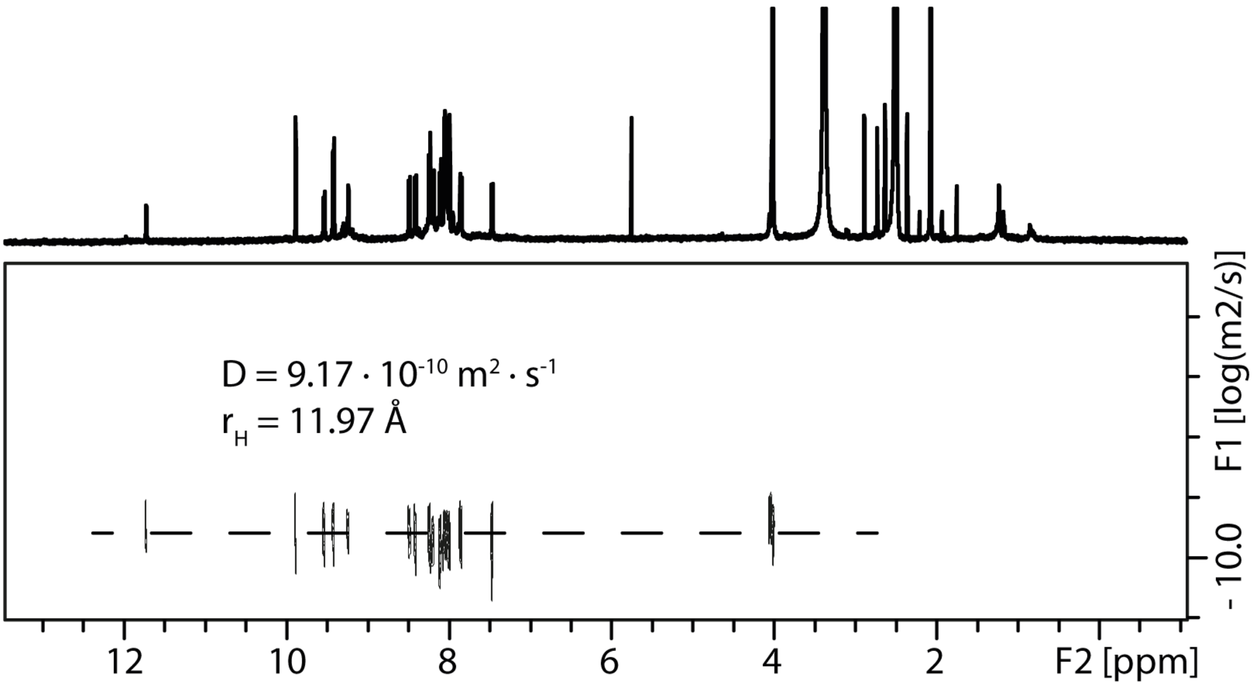

Figure S32: ^{1}H DOSY NMR (500 MHz, 298 K, DMSO-d6) of Pd_2**L**$^1{}_2$**L**$^A{}_2$.

^{13}C{^{1}H} NMR (151 MHz, 298 K, DMSO-d6): δ = 171.6, 162.4, 153.8, 151.0, 144.8, 142.4, 140.2, 136.2, 134.9, 133.9, 130.0, 129.0, 128.0, 127.2, 123.4, 123.0, 121.6, 121.1, 119.0, 118.1, 114.7, 99.4, 98.5, 86.3, 84.7, 79.2, 61.2.

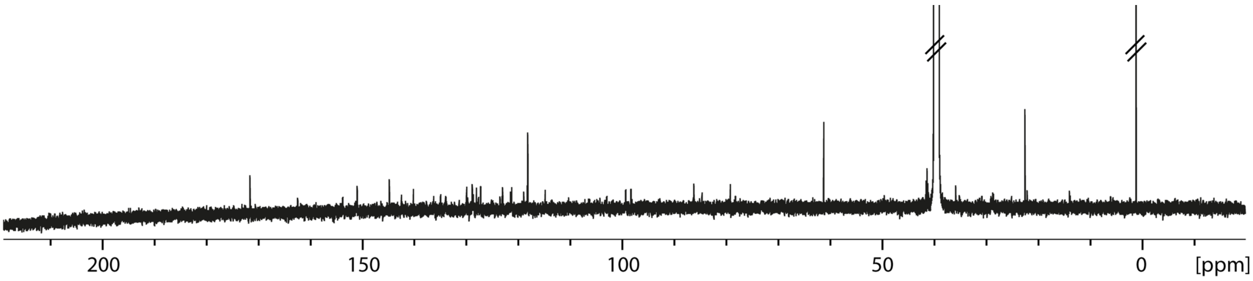

Figure S33: ^{13}C{^{1}H} NMR (151 MHz, 298 K, DMSO-d6) of Pd_2**L**$^1{}_2$**L**$^A{}_2$.

HR-ESI-MS (positive mode):

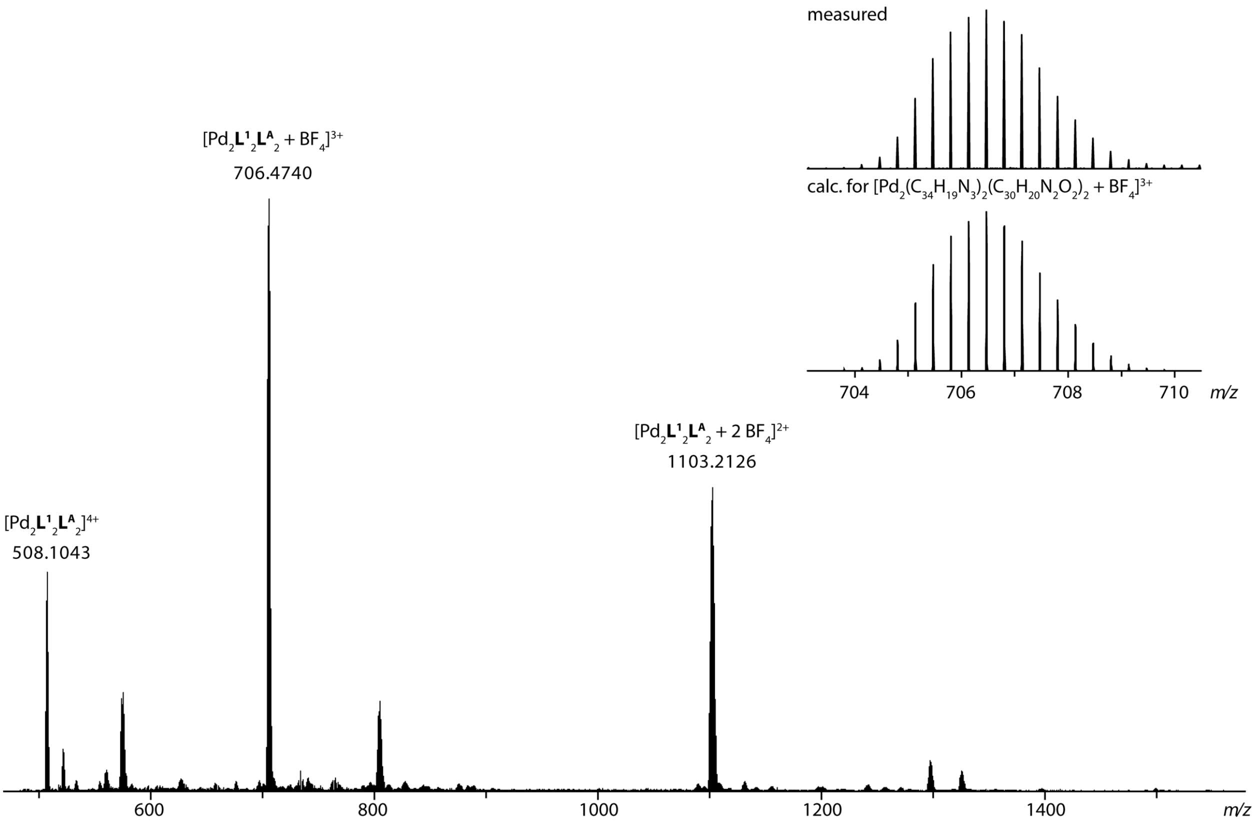

Figure S34: ESI-MS spectrum of [Pd$_2$**L**$^1_2$**L**$^A_2$ + n BF$_4$]$^{(4-n)+}$ and inset of comparison of measured and calculated mass.

2.12.2 Pd$_2$L$^1_2$L$^B_2$

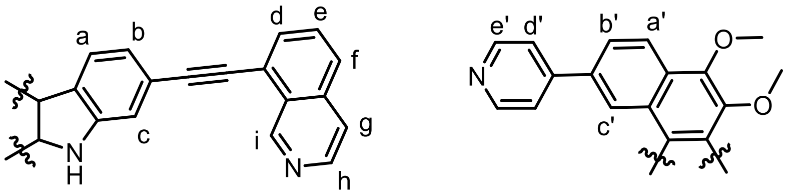

Scheme S13: Ligand Assignment for Pd$_2$**L**$^1_2$**L**$^B_2$.

^{1}**H NMR** (500 MHz, 298 K, DMSO-d$_6$): δ = 11.89 (s, 2H, N*H*), 10.09 (s, 4H, i), 9.73 (d, ^3J = 6.4, 8H, e'), 9.66 (d, ^3J = 6.6, 4H, h), 9.17 (s (br), 4H, c'), 8.45 (d, ^3J = 6.5, 4H, g), 8.32 (m, 12H, d',f), 8.20 (d, ^3J = 8.3, 4H, d), 8.15 (d, ^3J = 8.1, 4H, a), 8.08 (m, 8H, a', b'), 8.01 (m, 4H, e), 7.96 (s, 4H, c), 7.42 (dd, ^3J = 7.9, ^4J = 1.2 , 4H, b)

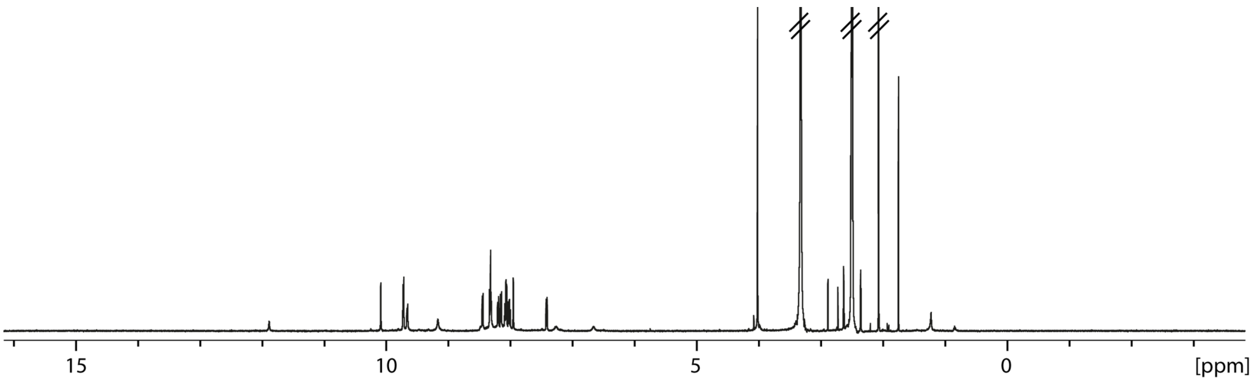

Figure S35: ^{1}H NMR (500 MHz, 298 K, DMSO-d$_6$) of $Pd_2\mathbf{L^1}_2\mathbf{L^B}_2$.

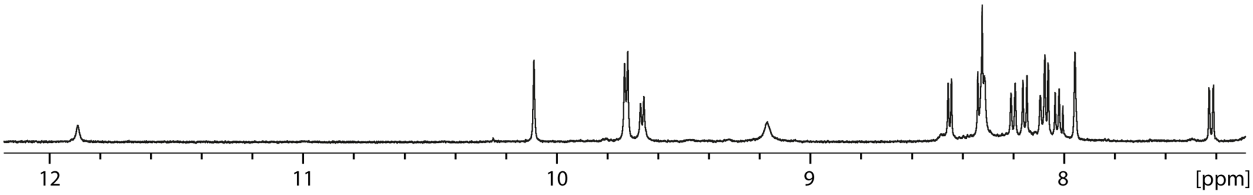

Figure S36: Partial ^{1}H NMR (500 MHz, 298 K, DMSO-d$_6$) of $Pd_2\mathbf{L^1}_2\mathbf{L^B}_2$.

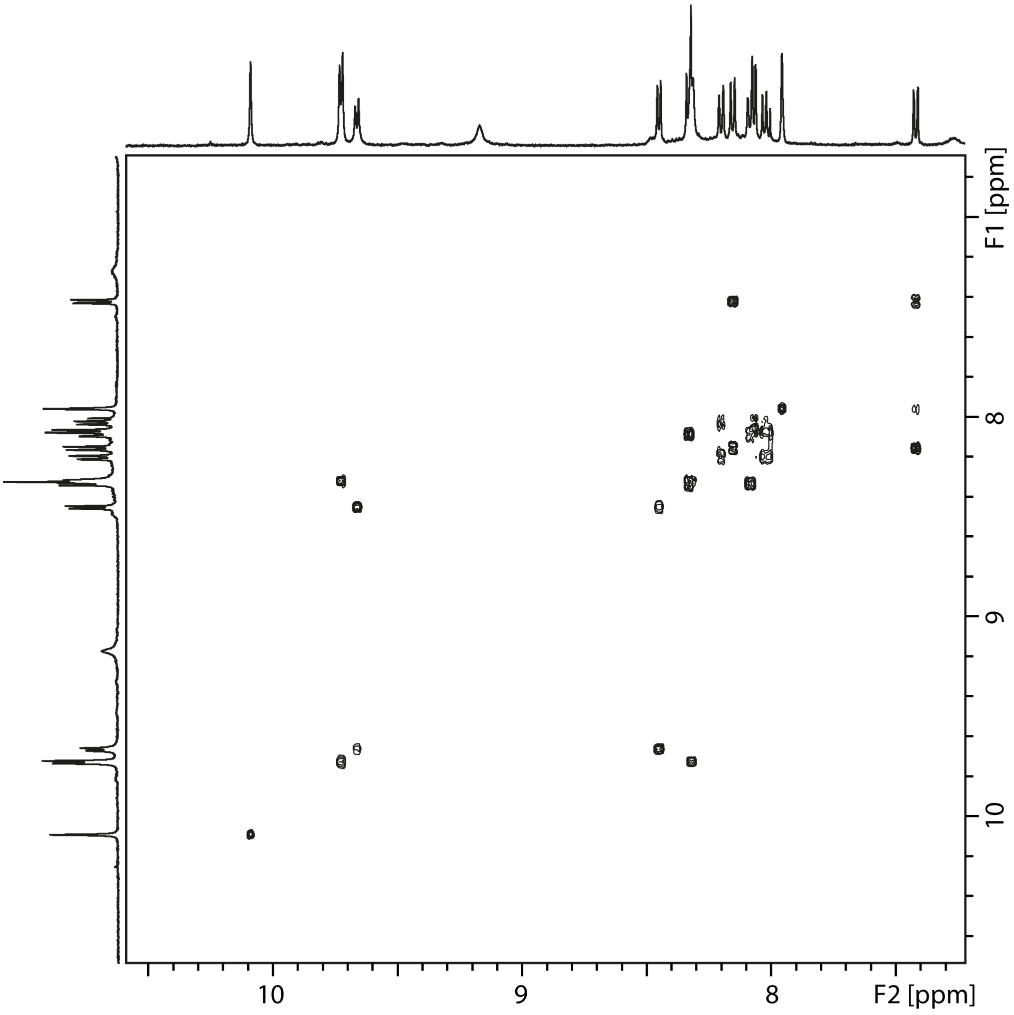

Figure S37: Partial ^{1}H−^{1}H COSY NMR (500 MHz, 298 K, DMSO-d$_6$) of $Pd_2\mathbf{L^1}_2\mathbf{L^B}_2$.

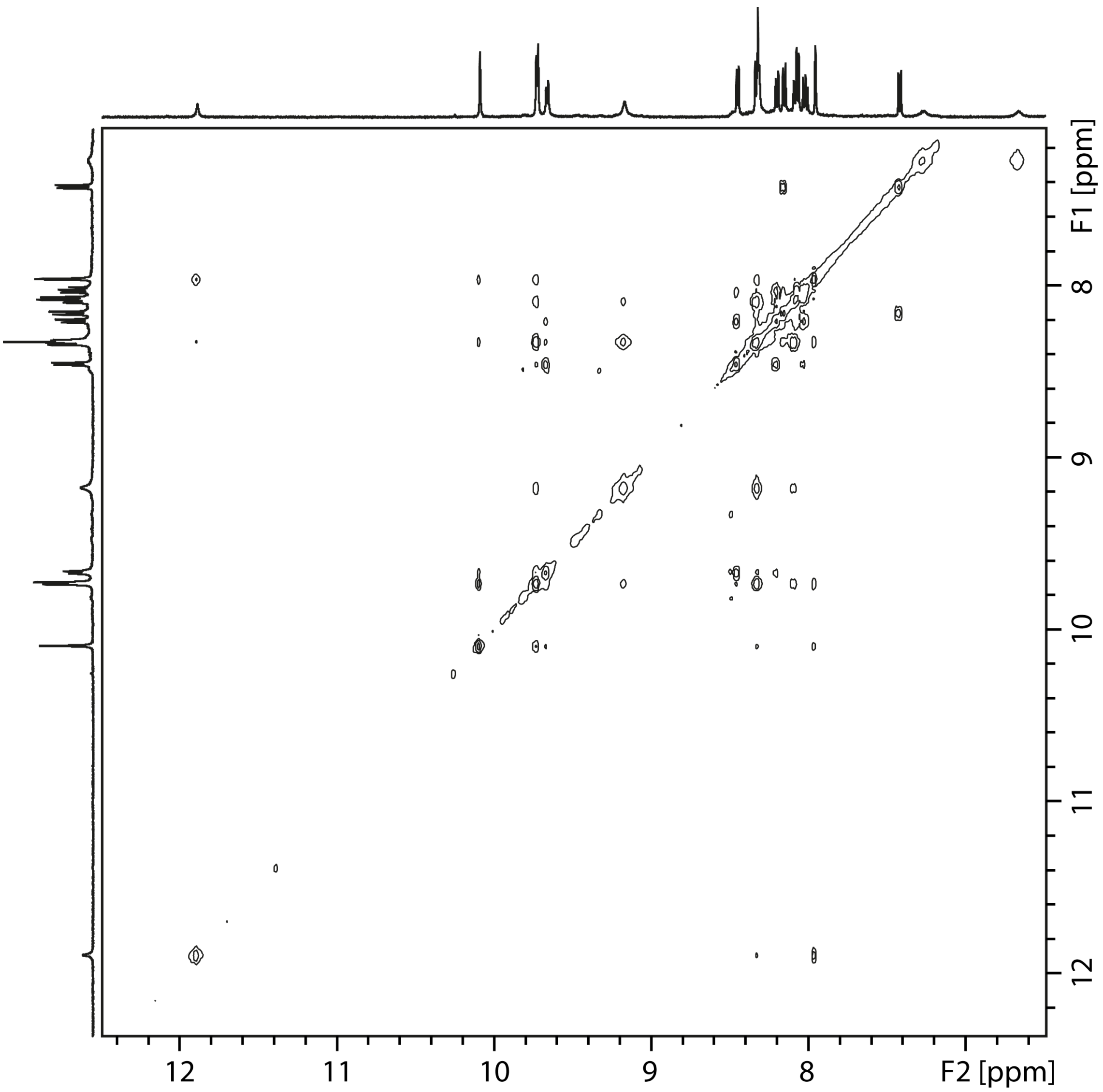

Figure S38: Partial ^{1}H–^{1}H NOESY NMR (500 MHz, 298 K, DMSO-d$_6$) of Pd$_2$**L^1**$_2$**L^B**$_2$.

$^{13}C\{^{1}H\}$ NMR (151 MHz, 298 K, DMSO-d$_6$): δ = 171.5, 162.3, 151.5, 151.1, 144.2, 142.4, 140.0, 136.2, 133.0, 130.2, 128.2, 125.4, 123.1, 121.5, 121.2, 119.0, 118.1, 115.3, 98.5, 84.5, 61.2.

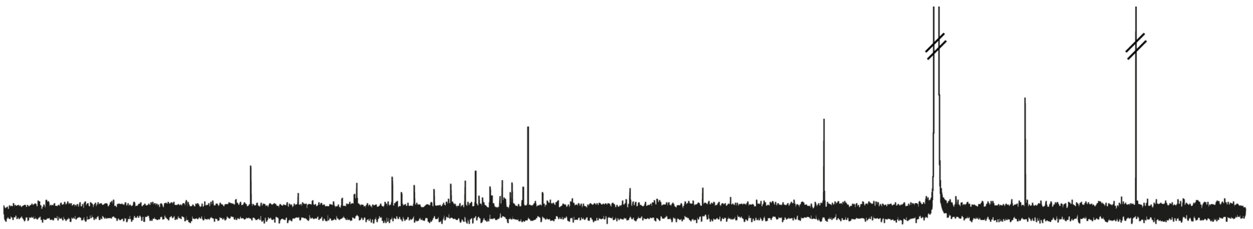

Figure S39: $^{13}C\{^{1}H\}$ NMR (151 MHz, 298 K, DMSO-d$_6$) of Pd$_2$**L^1**$_2$**L^B**$_2$.

HR-ESI-MS (positive mode):

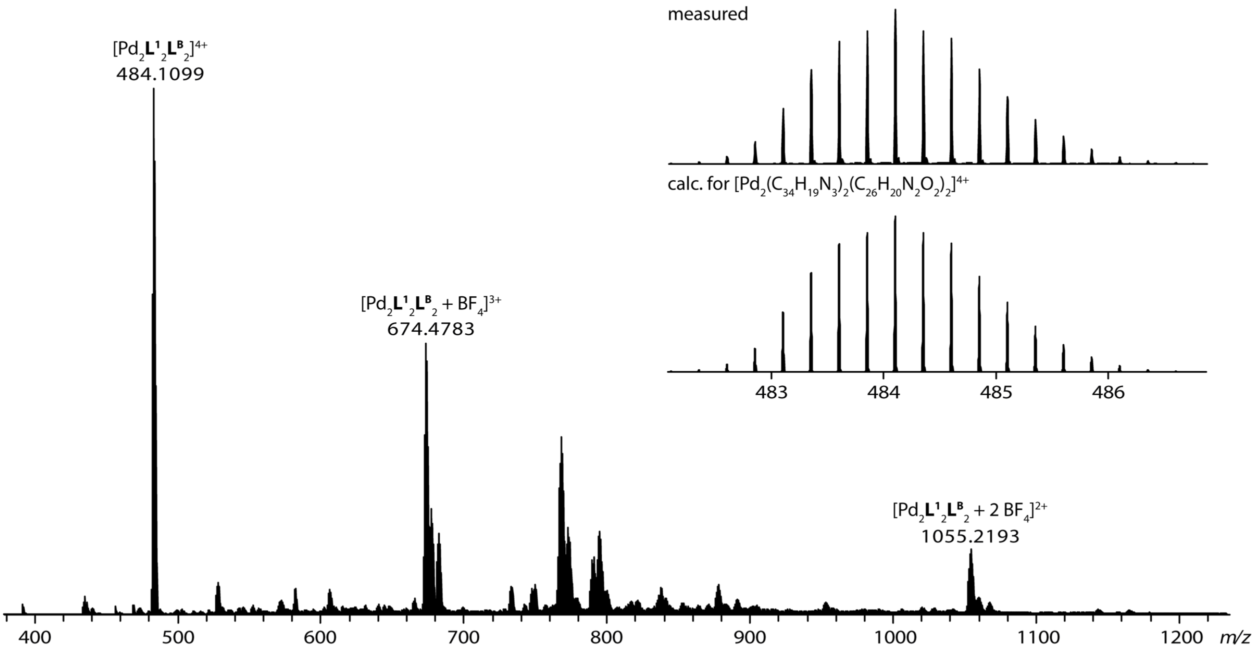

Figure S40: ESI-MS spectrum of [Pd_2**L**$^1{}_2$**L**$^B{}_2$ + n BF_4]$^{(4-n)+}$ and inset of comparison of measured and calculated mass.

2.12.3 $Pd_2L^1{}_2L^C{}_2$

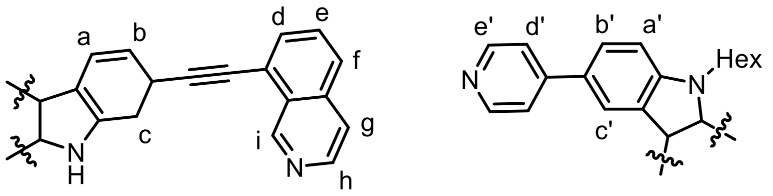

Scheme S14: Ligand Assignment for Pd_2**L**$^1{}_2$**L**$^C{}_2$.

^{1}H NMR (500 MHz, 298 K, DMSO-d$_6$): δ = 11.98 (s, 2H, N*H*), 10.14 (s, 4H, i), 9.45 (s (br), 8H, e'), 9.36 (d, ^3J = 6.6, 4H, h), 8.44 (m, 8H, g, c'), 8.26 (d, ^3J = 8.4, 4H, f), 8.10 (m, 4H, d), 8.05 (t, ^3J = 8.0, 4H, e), 7.93 (m, 16H, c, b', d'), 7.83 (d, ^3J = 8.7, 4H, a'), 7.77 (d, ^3J = 7.9, 4H, a), 7.23 (dd, ^3J = 7.9, ^4J = 1.0, 4H, b), 4.47 (s (br), 4H, NC*H*$_2$), 1.24−1.04 (m, 16H, C*H*$_2$), 0.65 (t, ^3J = 7.3, 6H, C*H*$_3$).

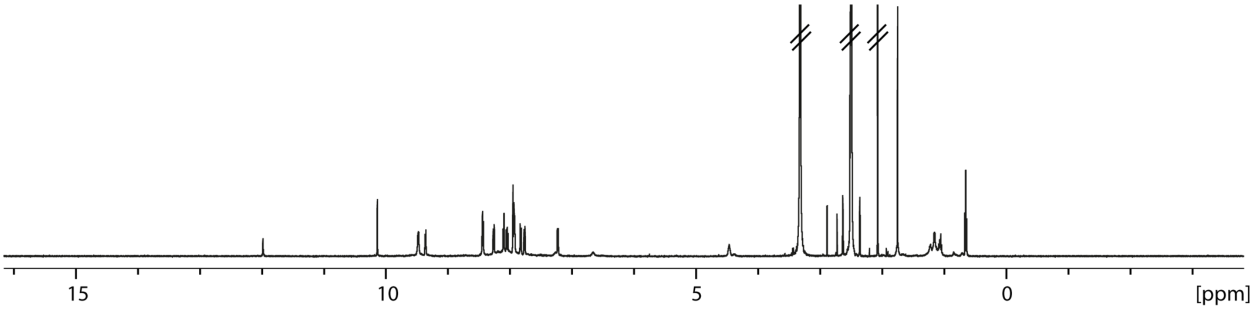

Figure S41: ^{1}H NMR (500 MHz, 298 K, DMSO-d$_6$) of Pd_2**L**$^1{}_2$**L**$^C{}_2$.

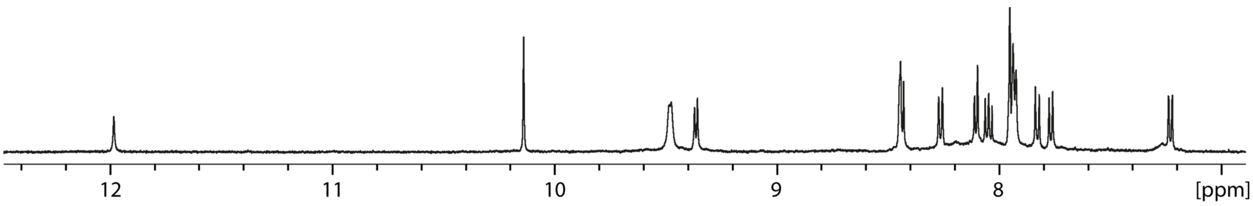

Figure S42: Partial ^{1}H NMR (500 MHz, 298 K, DMSO-d_6) of $Pd_2\mathbf{L^1}_2\mathbf{L^C}_2$.

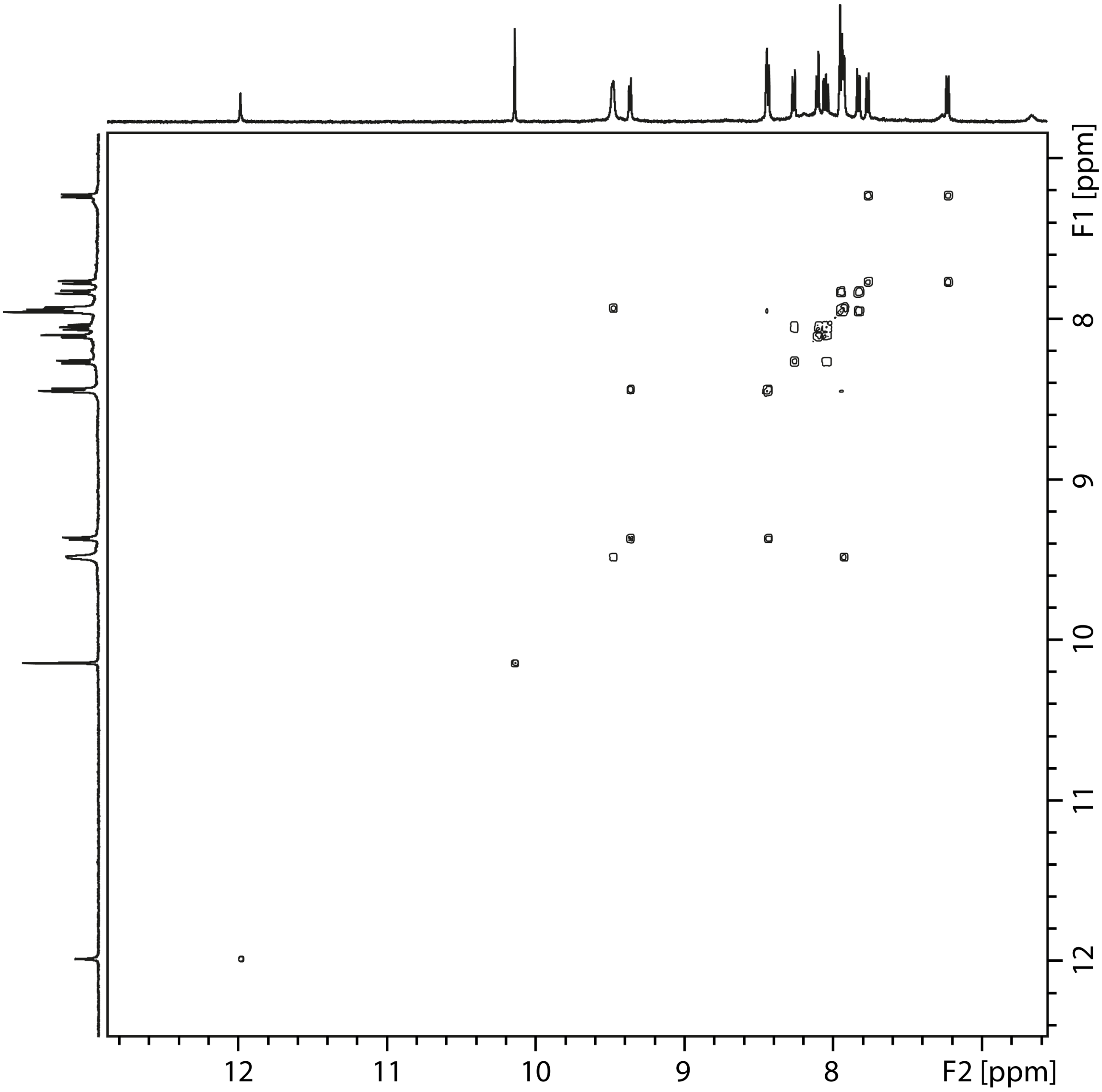

Figure S43: Partial ^{1}H–^{1}H COSY NMR (600 MHz, 298 K, DMSO-d_6) of $Pd_2\mathbf{L^1}_2\mathbf{L^C}_2$.

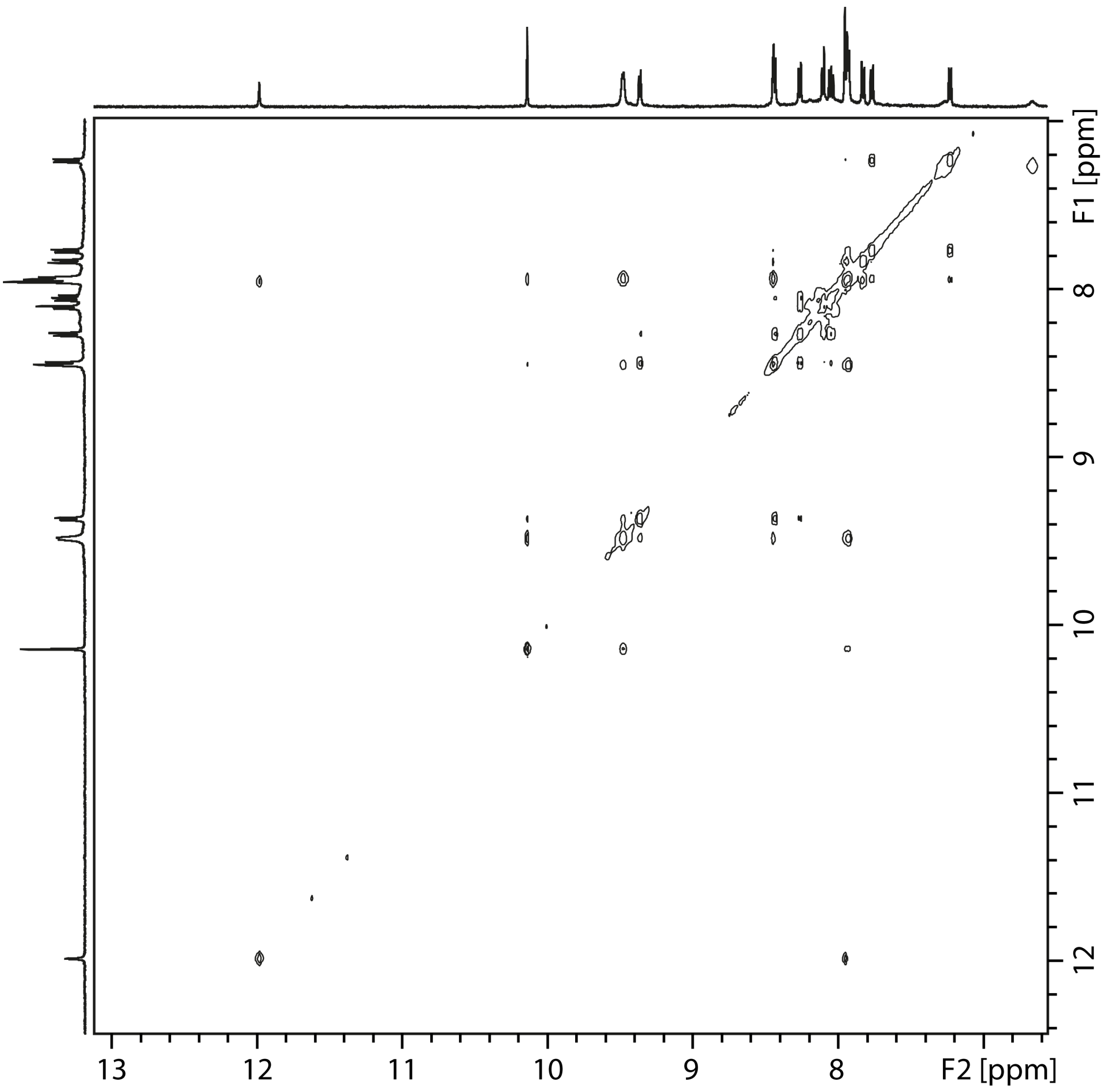

Figure S44: Partial ^{1}H–^{1}H NOESY NMR (600 MHz, 298 K, DMSO-d$_6$) of $Pd_2\mathbf{L}^1_2\mathbf{L}^C_2$.

^{13}C{^{1}H} NMR (151 MHz, 298 K, DMSO-d$_6$): δ = 171.4, 162.3, 153.4, 151.9, 150.4, 142.7, 142.1, 140.3, 136.3, 133.9, 127.7, 125.8, 125.0, 124.2, 123.3, 123.1, 122.8, 121.5, 121.1, 119.1, 118.1, 114.0, 111.2, 98.2, 84.1, 30.7, 28.5, 25.8, 22.5, 21.9, 13.6.

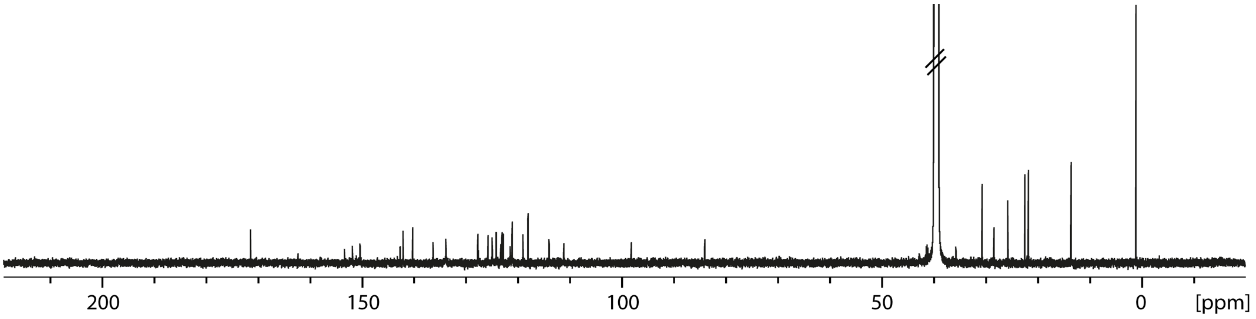

Figure S45: ^{13}C{^{1}H} NMR (151 MHz, 298 K, DMSO-d$_6$) of $Pd_2\mathbf{L}^1_2\mathbf{L}^C_2$.

HR-ESI-MS (positive mode):

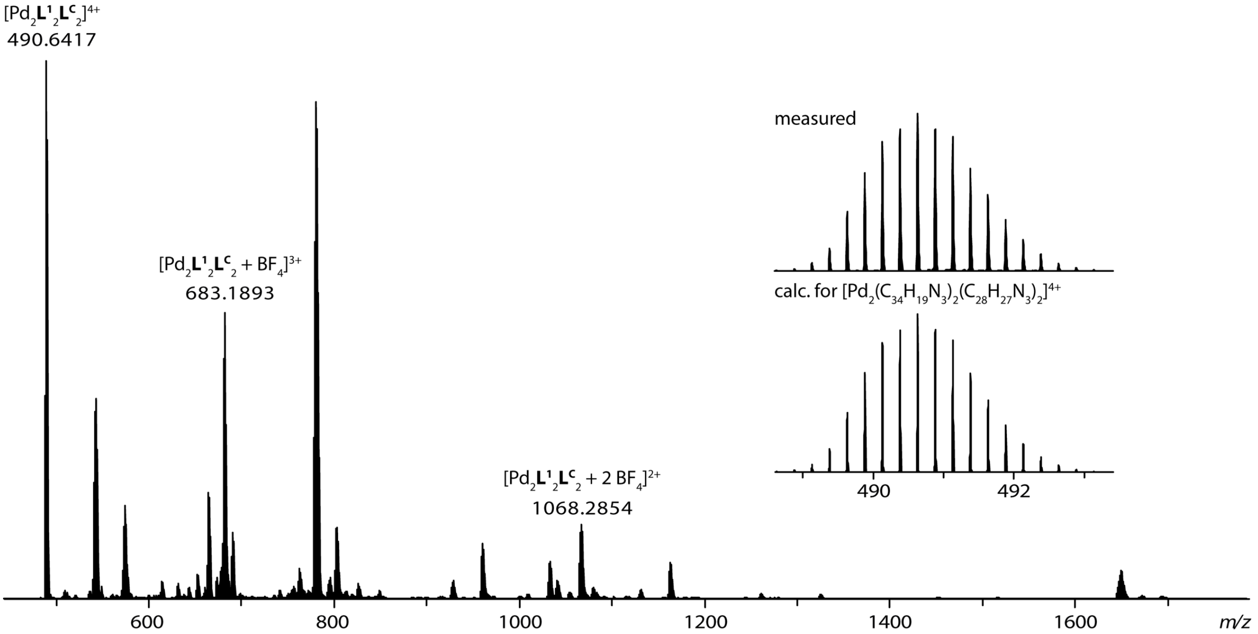

Figure S46: ESI-MS spectrum of $[Pd_2\mathbf{L^1}_2\mathbf{L^C}_2 + n\ BF_4]^{(4-n)+}$ and inset of comparison of measured and calculated mass.

2.12.4 $Pd_2L^1{}_2L^D{}_2$

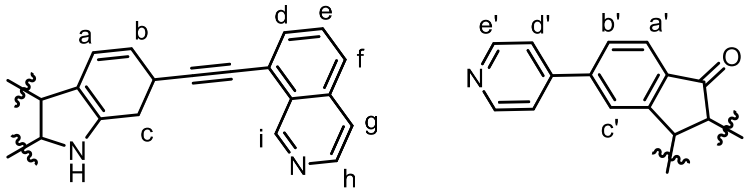

Scheme S15: Ligand Assignment for $Pd_2\mathbf{L^1}_2\mathbf{L^D}_2$.

^{1}H NMR (500 MHz, 298 K, DMSO-d$_6$): δ = 12.00 (s, 2H, N*H*), 10.04 (s, 4H, i), 9.58 (d, ^3J = 6.3, 8H, e'), 9.33 (d, ^3J = 6.5, 4H, h), 8.42 (d, ^3J = 6.4, 4H, g), 8.25 (d, ^3J = 8.3, 4H, f), 8.09 (dd, ^3J = 7.1, ^4J = 1.1, 4H, d), 8.05 (m, 4H, e), 7.98 (d, ^3J = 8.1, 4H, a'), 7.94 (s, 4H, c'), 7.91 (s, 4H, c), 7.88 (d, ^3J = 6.2, 8H, a, b'), 7.84 (s, 8H, d'), 7.23 (dd, ^3J = 7.9, ^4J = 1.1, 4H, b).

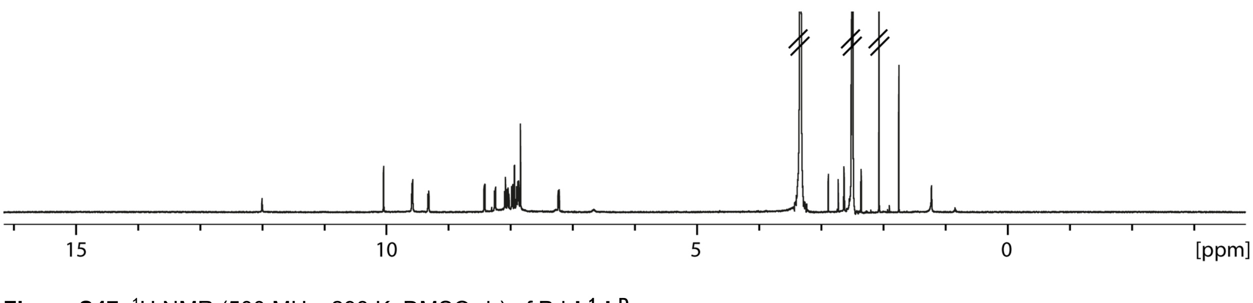

Figure S47: ^{1}H NMR (500 MHz, 298 K, DMSO-d$_6$) of $Pd_2\mathbf{L^1}_2\mathbf{L^D}_2$.

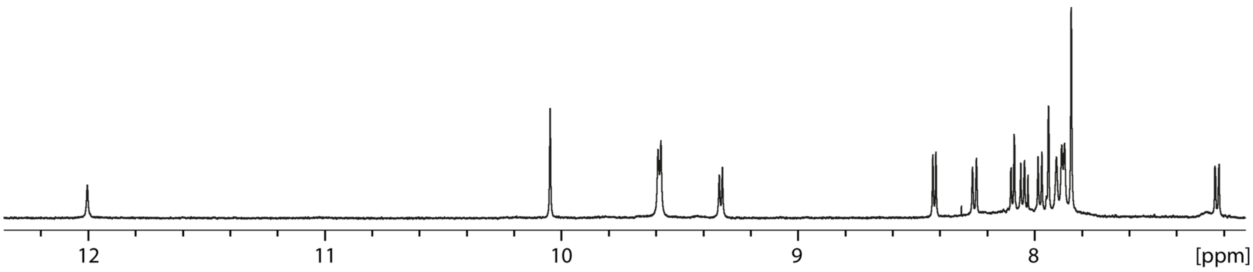

Figure S48: Partial ^{1}H NMR (500 MHz, 298 K, DMSO-d_6) of Pd_2**L**$^{1}{}_2$**L**$^{D}{}_2$.

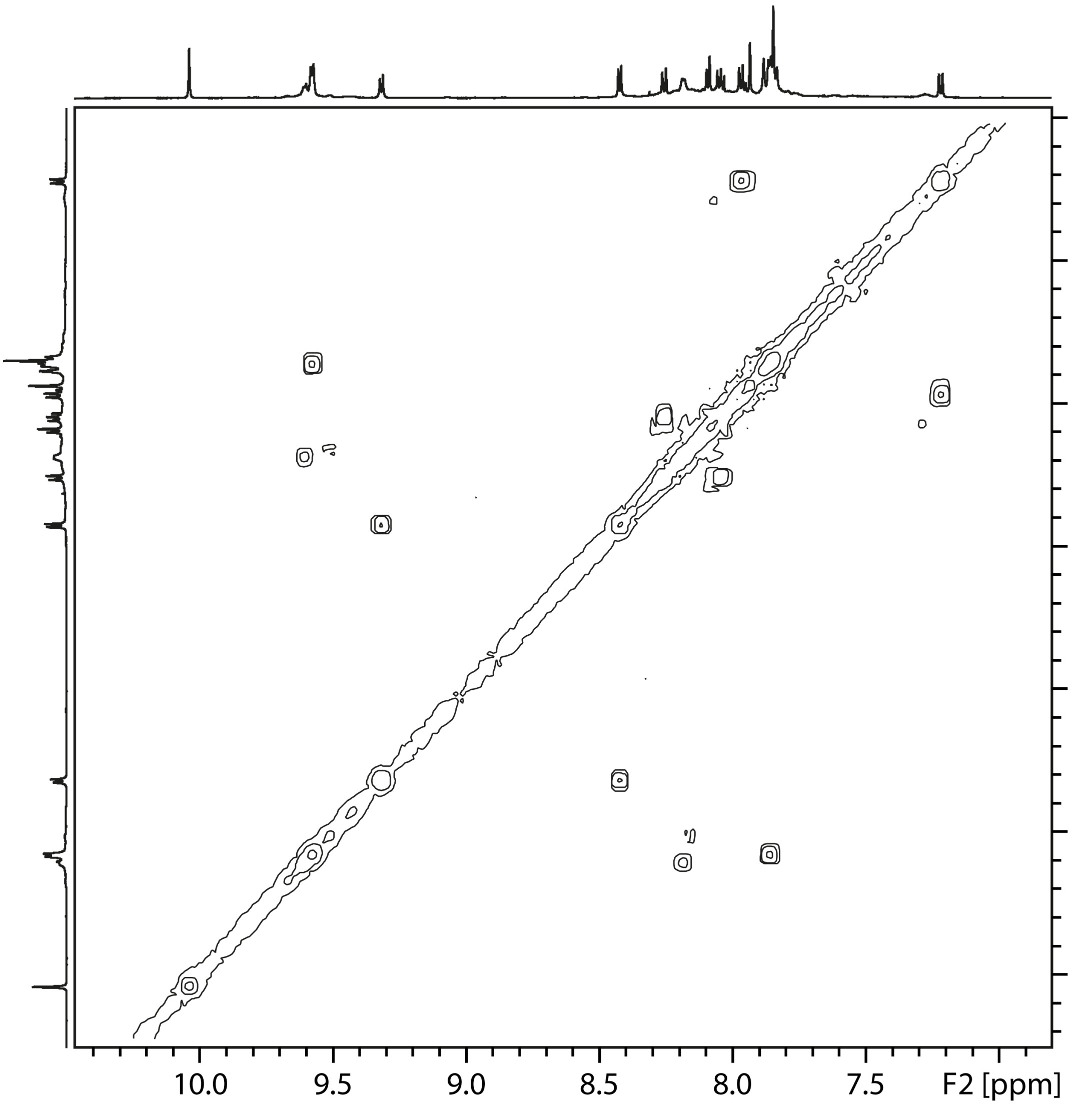

Figure S49: Partial ^{1}H–^{1}H COSY NMR (600 MHz, 298 K, DMSO-d_6) of Pd_2**L**$^{1}{}_2$**L**$^{D}{}_2$.

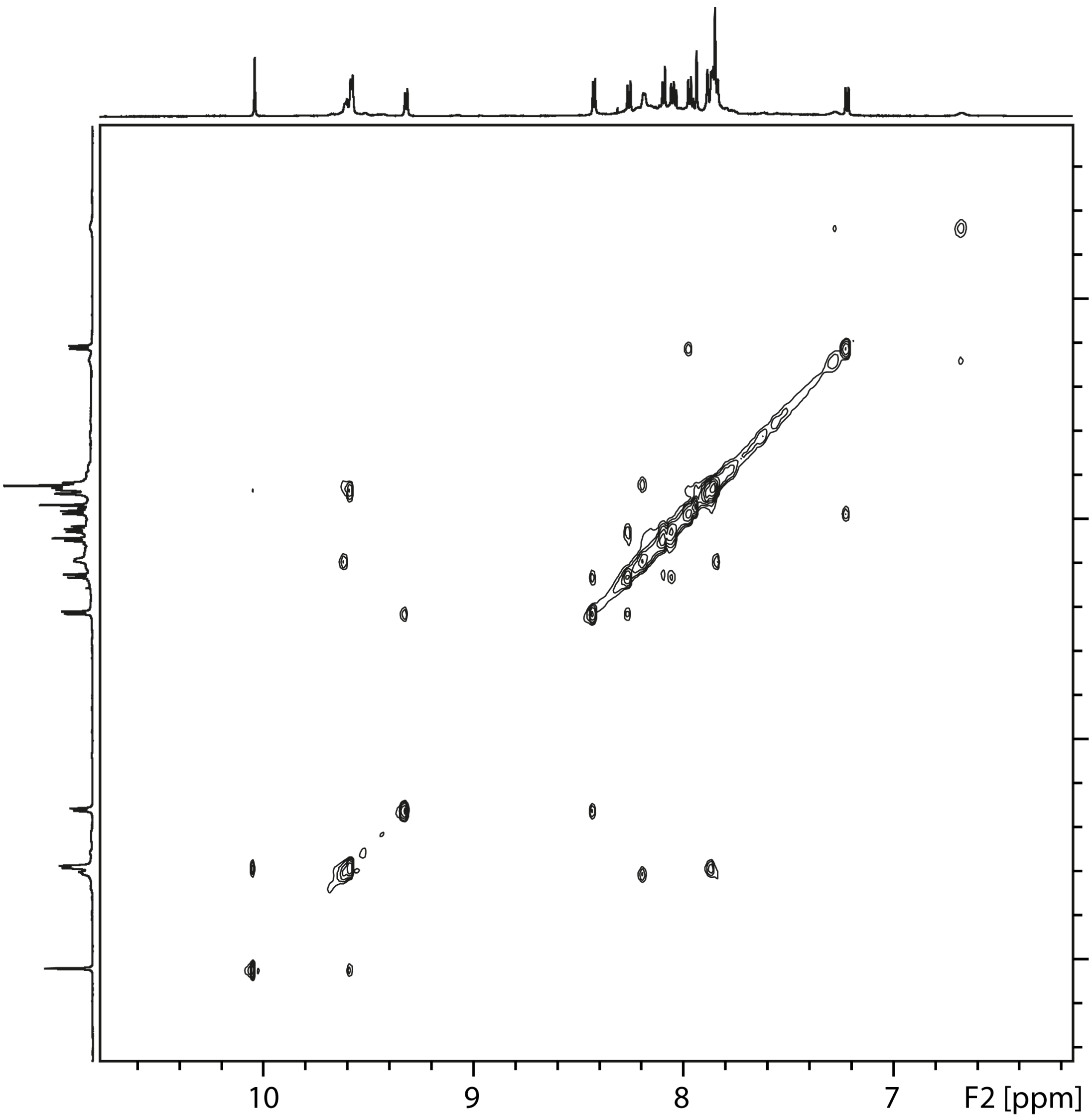

Figure S50: Partial ^1H–^1H NOESY NMR (600 MHz, 298 K, DMSO-d$_6$) of Pd$_2$**L**$^1{}_2$**L**$^D{}_2$.

^{13}**C{^{1}H} NMR** (151 MHz, 298 K, DMSO-d$_6$): δ = 191.6, 171.4, 162.3, 153.4, 150.9, 150.1, 143.8, 142.6, 141.1, 140.3, 136.3, 134.8, 134.0, 133.7, 128.8, 127.8, 127.7, 125.2, 123.2, 123.0, 121.4, 121.1, 119.0, 118.1, 114.1, 98.2, 83.8.

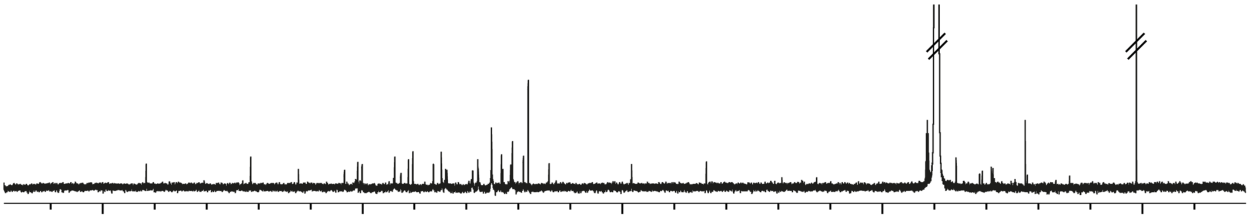

Figure S51: ^{13}C{^{1}H} NMR (151 MHz, 298 K, DMSO-d$_6$) of Pd$_2$**L**$^1{}_2$**L**$^D{}_2$.

HR-ESI-MS (positive mode):

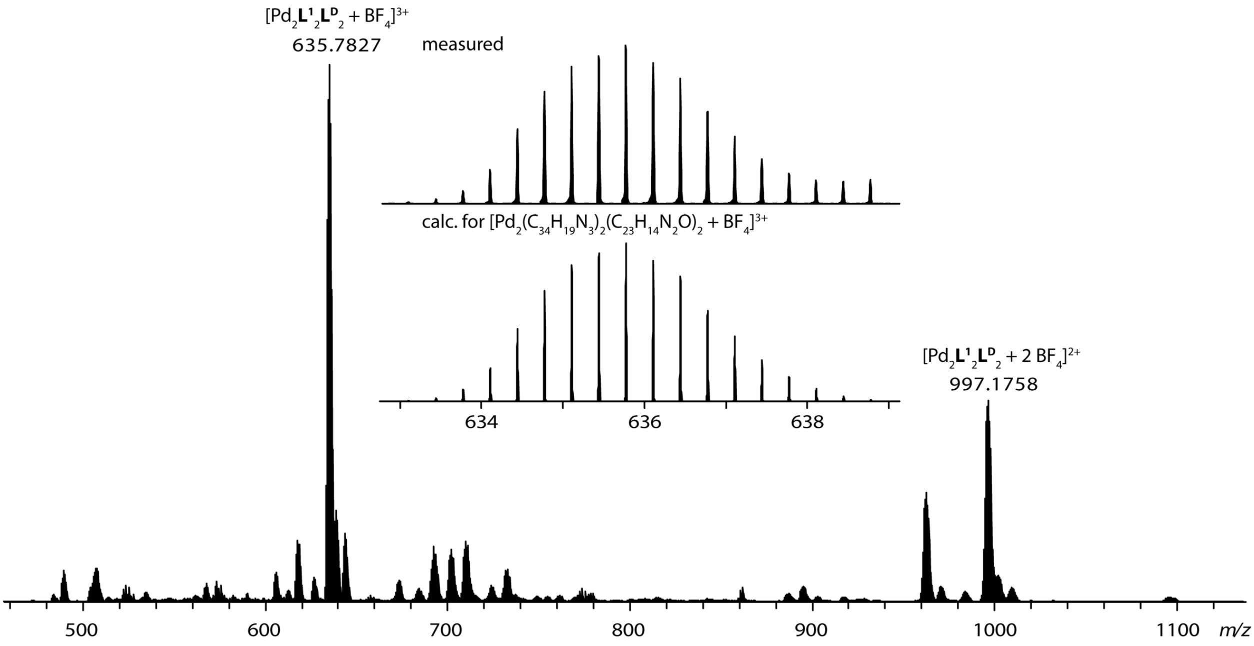

Figure S52: ESI-MS spectrum of [Pd_2**L**$^1{}_2$**L**$^D{}_2$ + n BF_4]$^{(4-n)+}$ and inset of comparison of measured and calculated mass.

2.12.5 $Pd_2L^2{}_2L^A{}_2$

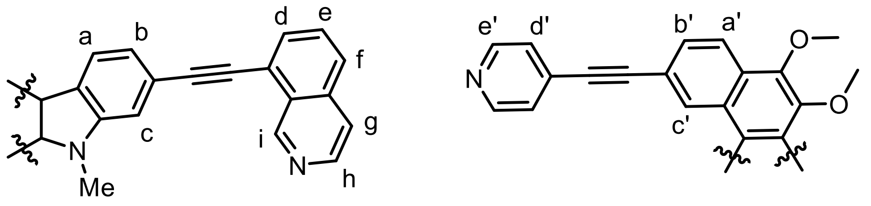

Scheme S16: Ligand Assignment for Pd_2**L**$^2{}_2$**L**$^A{}_2$.

^{1}H NMR (500 MHz, 298 K, DMSO-d$_6$): δ = 9.84 (s, 4H, i), 9.50 (d, ^3J = 6.7, 4H, h), 9.32 (d, ^3J = 6.8, 8H, e'), 9.27 (s, 4H, c'), 8.59 (d, ^3J = 8.0, 4H, a), 8.41 (d, ^3J = 6.7, 4H, g), 8.24 (d, ^3J = 8.6, 4H, a'), 8. 21 (d, ^3J = 8.4, 4H, f), 8.12 (m, 8H, c, d), 8.03 (m, 4H, e), 8.00 (d, ^3J = 6.8, 8H, d'), 7.87 (d, ^3J = 8.5, 4H, b'), 7.51 (dd, ^3J = 7.6, ^4J = 1.1, 4H, b), 4.05 (s, 6H, NCH_3), 4.01 (s, 12H, OCH_3).

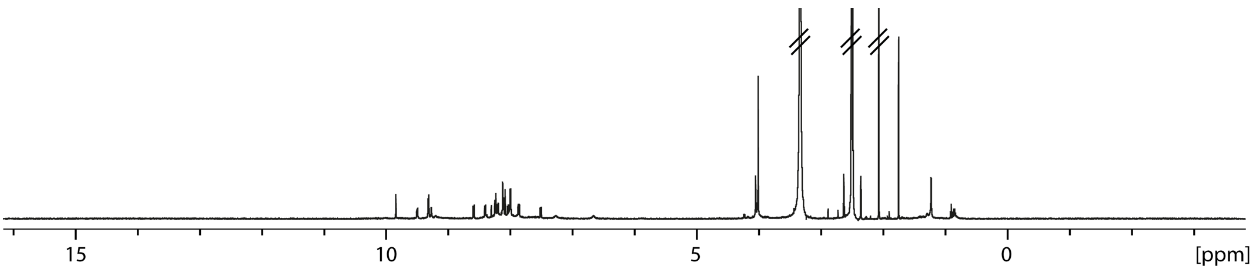

Figure S53: ^{1}H NMR (500 MHz, 298 K, DMSO-d$_6$) of Pd_2**L**$^2{}_2$**L**$^A{}_2$.

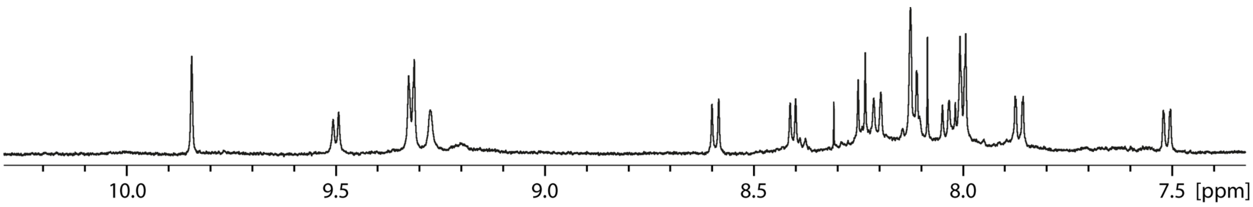

Figure S54: Partial ^{1}H NMR (500 MHz, 298 K, DMSO-d$_6$) of Pd$_2$**L**$^{\mathbf{2}}$$_2$**L**$^{\mathbf{A}}$$_2$.

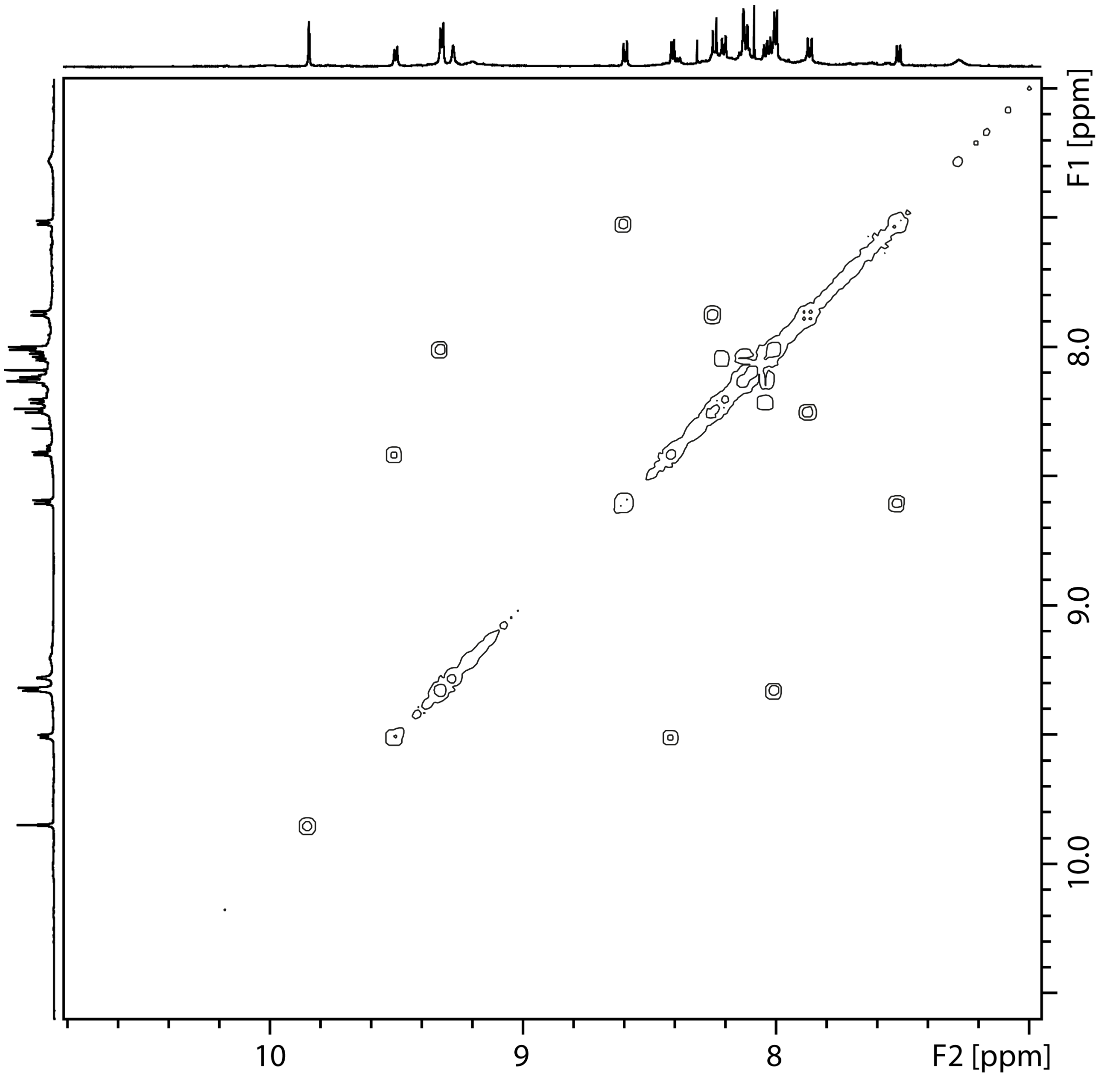

Figure S55: Partial ^{1}H−^{1}H COSY NMR (600 MHz, 298 K, DMSO-d$_6$) of Pd$_2$**L**$^{\mathbf{2}}$$_2$**L**$^{\mathbf{A}}$$_2$.

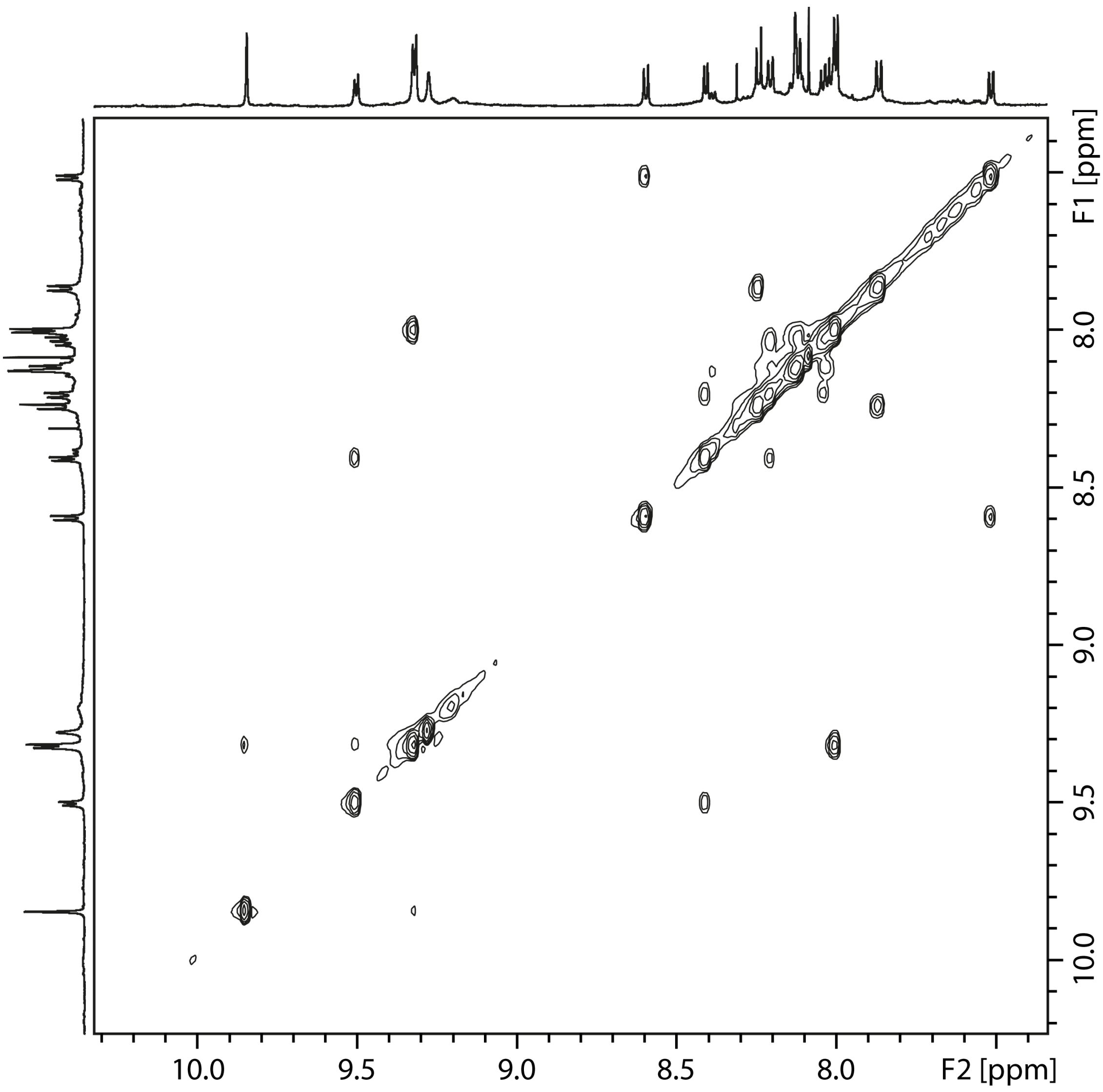

Figure S56: Partial ^{1}H–^{1}H NOESY NMR (600 MHz, 298 K, DMSO-d$_6$) of Pd$_2$**L**$^{2}{}_{2}$**L**$^{A}{}_{2}$.

$^{13}C\{^{1}H\}$ NMR (151 MHz, 298 K, DMSO-d$_6$): δ = 171.5, 151.1, 144.7, 141.1, 136.2, 134.8, 133.8, 129.9, 129.5, 128.8, 128.6, 128.1, 127.8, 127.2, 123.0, 122.5, 121.5, 121.1, 119.0, 118.2, 118.1, 113.4, 99.4, 98.1, 86.2, 84.6, 61.2, 22.5.

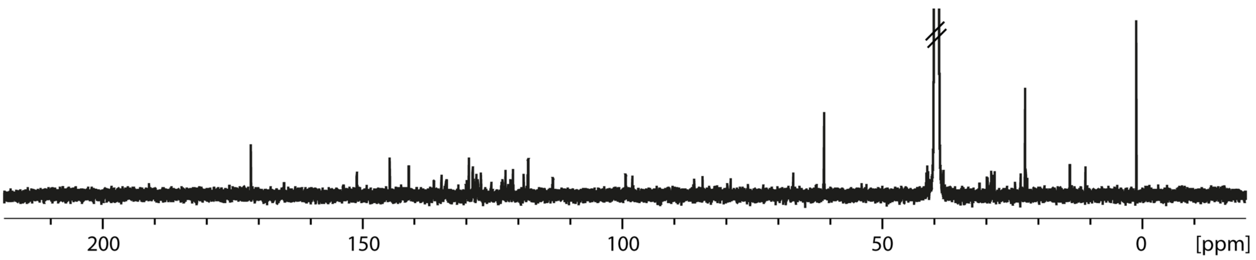

Figure S57: $^{13}C\{^{1}H\}$ NMR (151 MHz, 298 K, DMSO-d$_6$) of Pd$_2$**L**$^{2}{}_{2}$**L**$^{A}{}_{2}$.

HR-ESI-MS (positive mode):

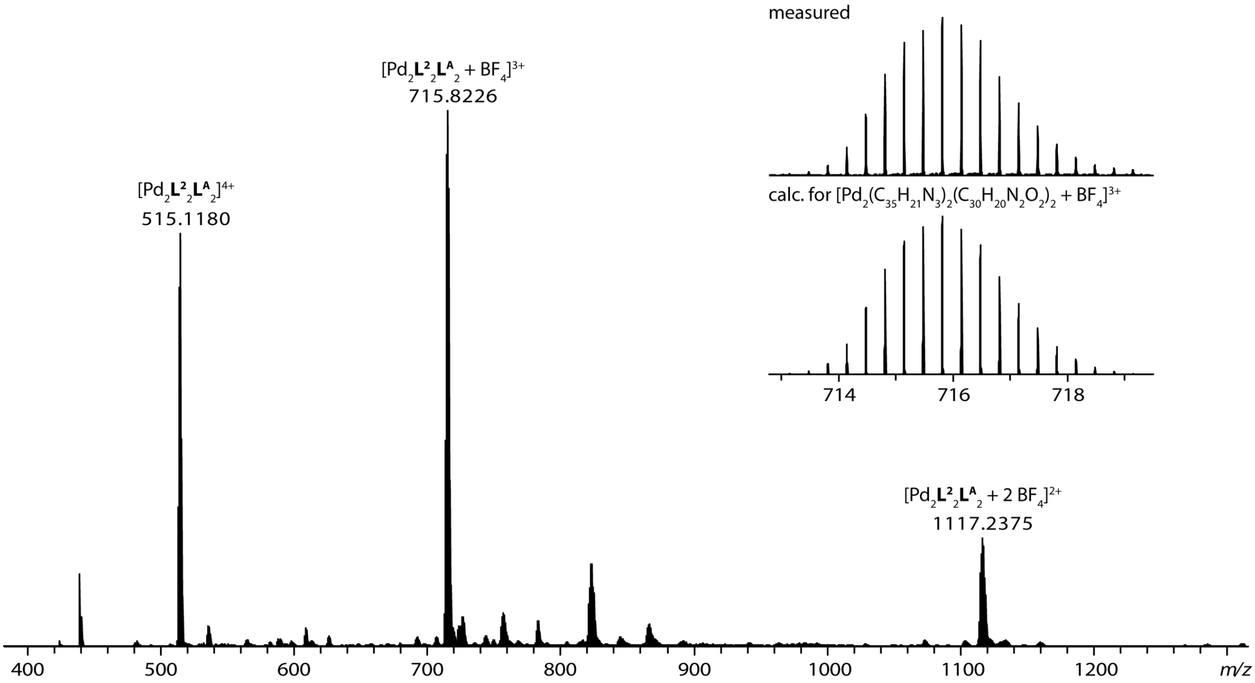

Figure S58: ESI-MS spectrum of [Pd_2**L**$^2{}_2$**L**$^A{}_2$ + n BF_4]$^{(4-n)+}$ and inset of comparison of measured and calculated mass.

2.12.6 $Pd_2L^2{}_2L^B{}_2$

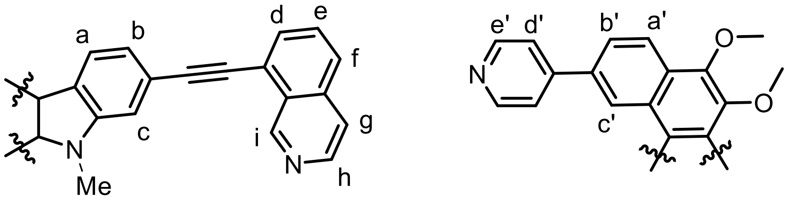

Scheme S17: Ligand Assignment for Pd_2**L**$^2{}_2$**L**$^B{}_2$.

^{1}H NMR (500 MHz, 298 K, DMSO-d6): δ = 10.21 (s, 4H, i), 9.79 (d, ^3J = 6.5, 4H, h), 9.75 (d, ^3J = 6.2, 8H, e'), 9.20 (s (br), 4H, c'), 8.46 (d, ^3J = 6.2, 12H, g, d'), 8.30 (m, 8H, a, a'), 8.17 (m, 8H, f, b'), 8.07 (d, ^3J = 6.5, 4H, d), 8.01 (m, 8H, c, e), 7.50 (d, ^3J = 8.7, 4H, b), 4.37 (s, 6H, NCH_3), 4.00 (s, 12H, OCH_3).

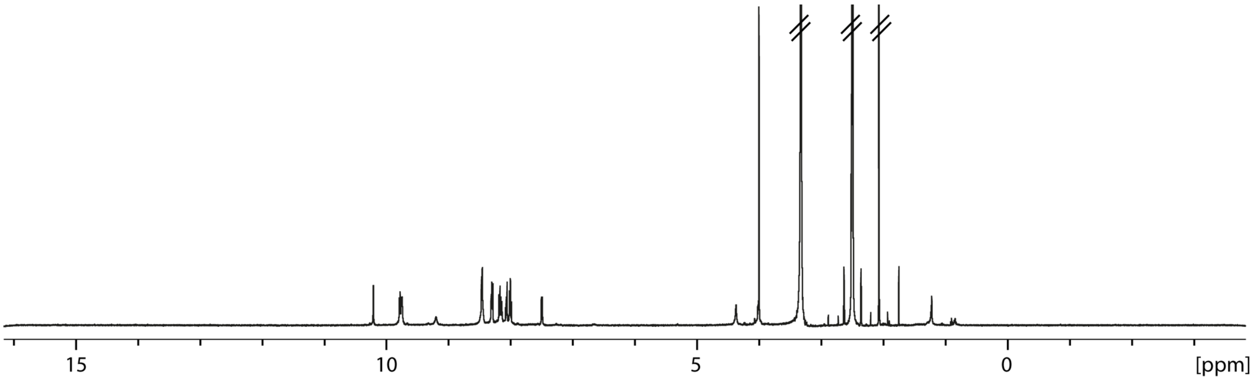

Figure S59: ^{1}H NMR (500 MHz, 298 K, DMSO-d6) of Pd_2**L**$^2{}_2$**L**$^B{}_2$.

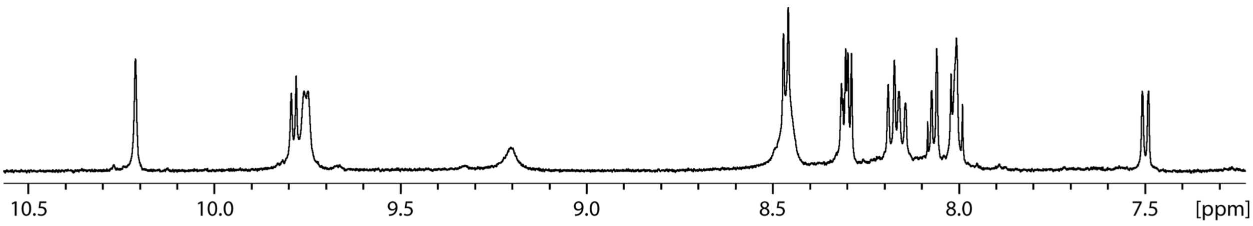

Figure S60: Partial ^{1}H NMR (500 MHz, 298 K, DMSO-d$_6$) of Pd$_2$**L**$^{2}_{2}$**L**$^{B}_{2}$.

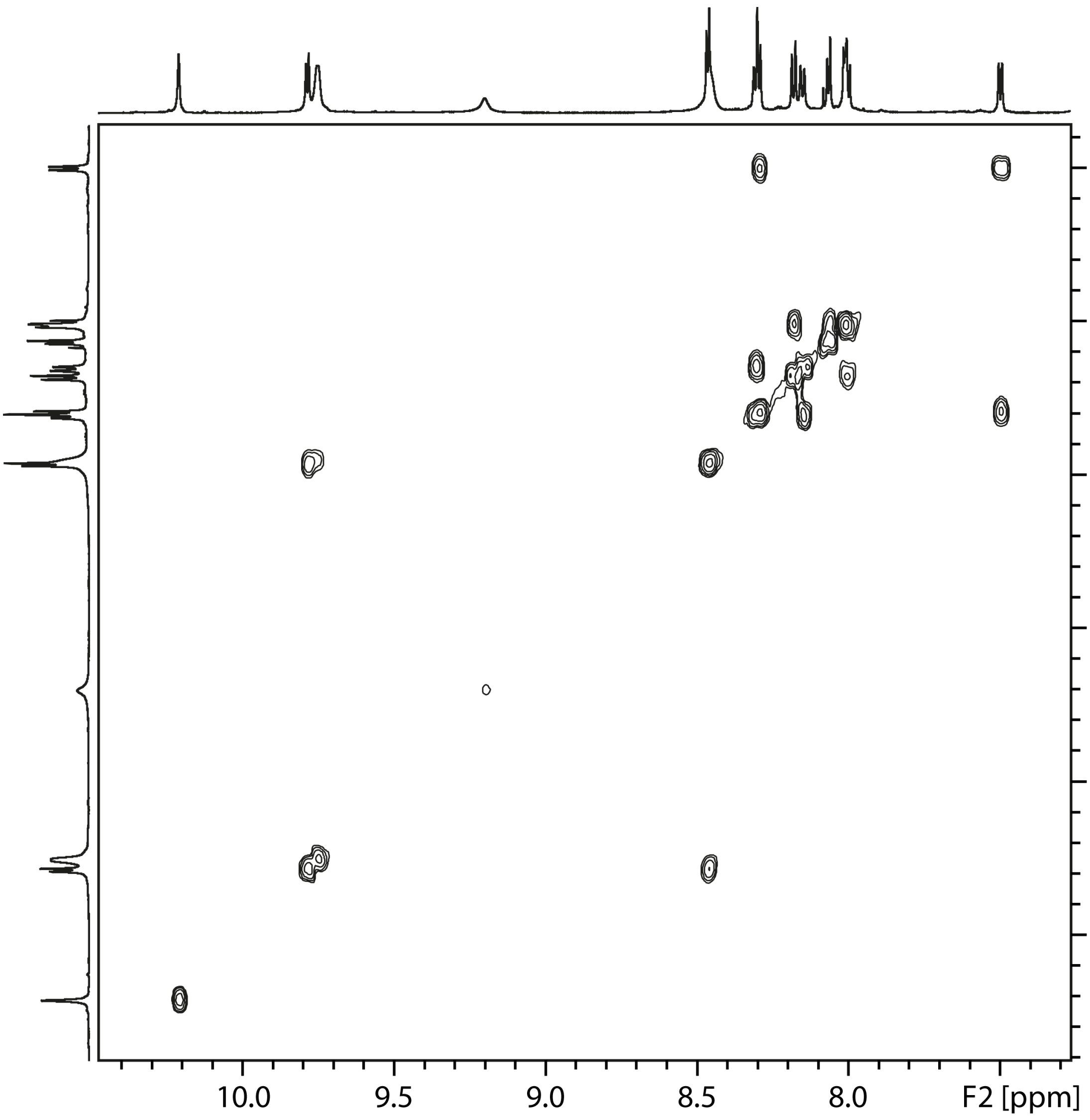

Figure S61: Partial ^{1}H–^{1}H COSY NMR (700 MHz, 298 K, DMSO-d$_6$) of Pd$_2$**L**$^{2}_{2}$**L**$^{B}_{2}$.

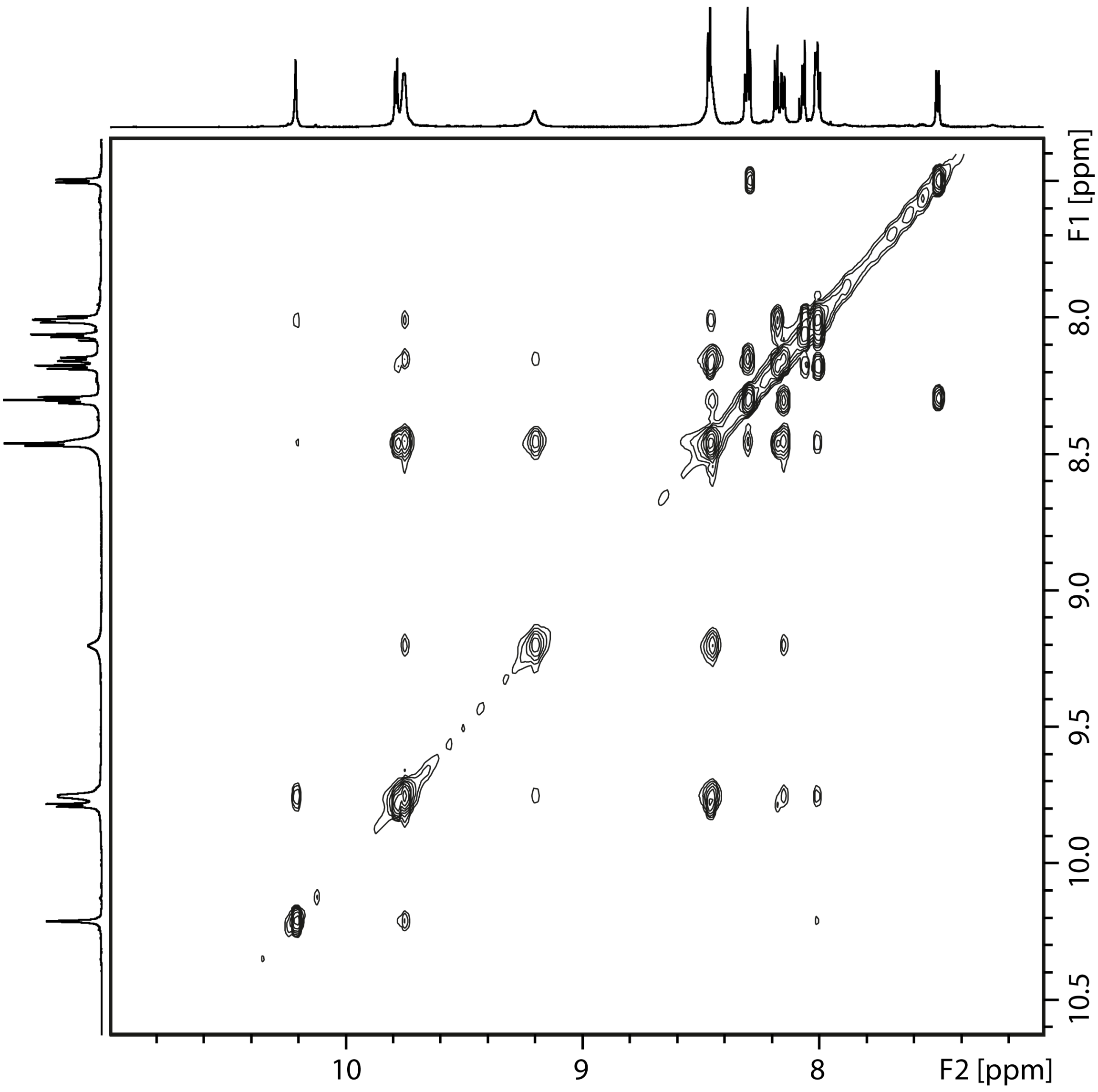

Figure S62: Partial $^{1}H-^{1}H$ NOESY NMR (700 MHz, 298 K, DMSO-d$_6$) of Pd$_2$**L**$^{\mathbf{2}}_2$**L**$^{\mathbf{B}}_2$.

^{13}C{^{1}H} NMR (176 MHz, 298 K, DMSO-d$_6$): δ = 154.4, 151.4, 150.3, 144.3, 142.3, 140.9, 136.1, 133.7, 133.4, 132.6, 130.3, 128.1, 127.5, 125.0, 124.6, 122.4, 121.5, 121.3, 118.9, 118.1, 114.2, 98.4, 84.8, 61.1, 22.5.

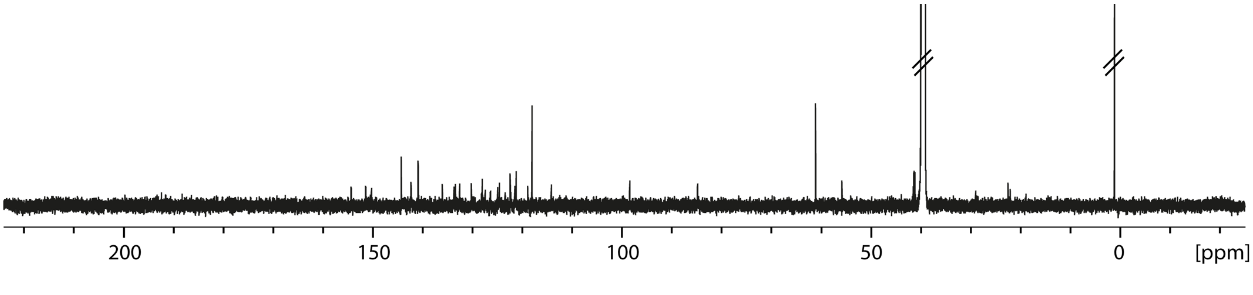

Figure S63: ^{13}C{^{1}H} NMR (176 MHz, 298 K, DMSO-d$_6$) of Pd$_2$**L**$^{\mathbf{2}}_2$**L**$^{\mathbf{B}}_2$.

HR-ESI-MS (positive mode):

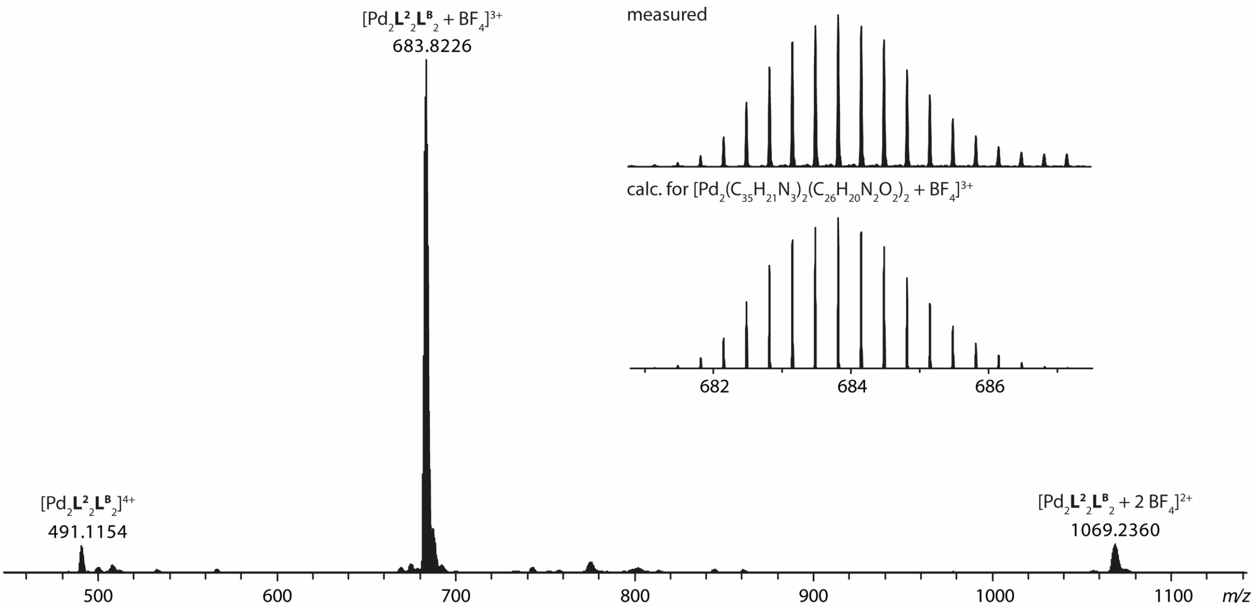

Figure S64: ESI-MS spectrum of $[Pd_2\mathbf{L}^2{}_2\mathbf{L}^B{}_2 + n\ BF_4]^{(4-n)+}$ and inset of comparison of measured and calculated mass.

2.12.7 $Pd_2L^3{}_2L^A{}_2$

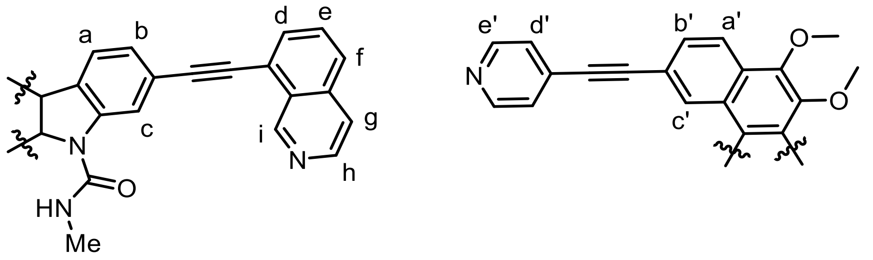

Scheme S18: Ligand Assignment for $Pd_2\mathbf{L}^3{}_2\mathbf{L}^A{}_2$.

^{1}H NMR (500 MHz, 298 K, DMSO-d$_6$): δ = 9.95 (s, 4H, i), 9.60 (m, 12H, h, e'), 9.01 (s, 4H, c'), 8.47 (m, 2H, N*H*), 8.43 (m, 8H, a, g), 8.37 (s, 4H, c), 8.24 (d, ^3J = 8.6, 4H, a') 8.21 (d, ^3J = 8.3, 4H, f), 8.15 (d, ^3J = 6.7, 8H, d'), 8.11 (d, ^3J = 6.4, 4H, d), 8.02 (t, ^3J = 8.0, 4H, e), 7.83 (d, ^3J = 9.7, 4H, b'), 7.69 (dd, ^3J = 7.8, ^4J = 1.0, 4H, b), 4.02 (s, 12H, OC*H*$_3$), 3.48 (d, ^3J = 4.4, 6H, C*H*$_3$).

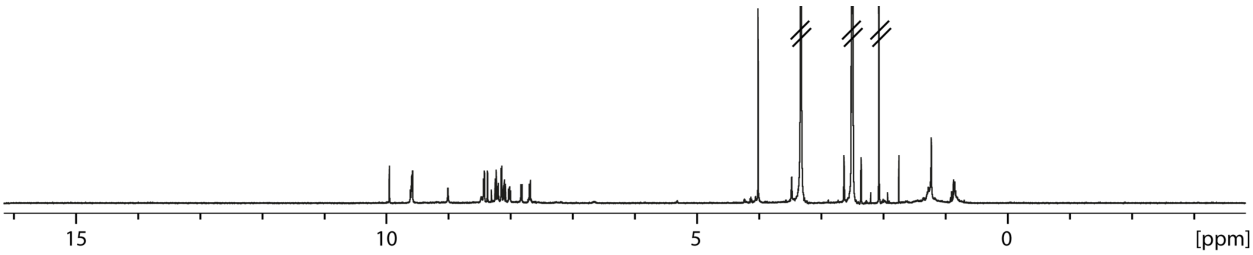

Figure S65: ^{1}H NMR (500 MHz, 298 K, DMSO-d$_6$) of $Pd_2\mathbf{L}^3{}_2\mathbf{L}^A{}_2$.

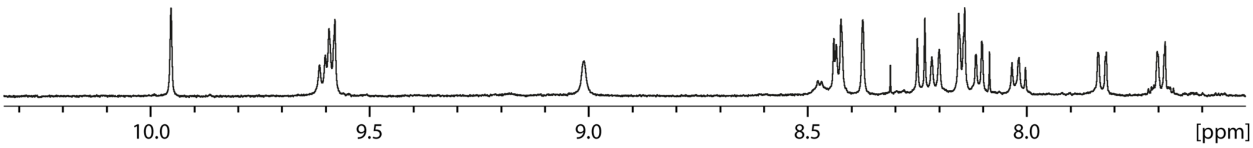

Figure S66: Partial ^{1}H NMR (500 MHz, 298 K, DMSO-d$_6$) of Pd$_2$**L**$^{3}_{2}$**L**$^{A}_{2}$.

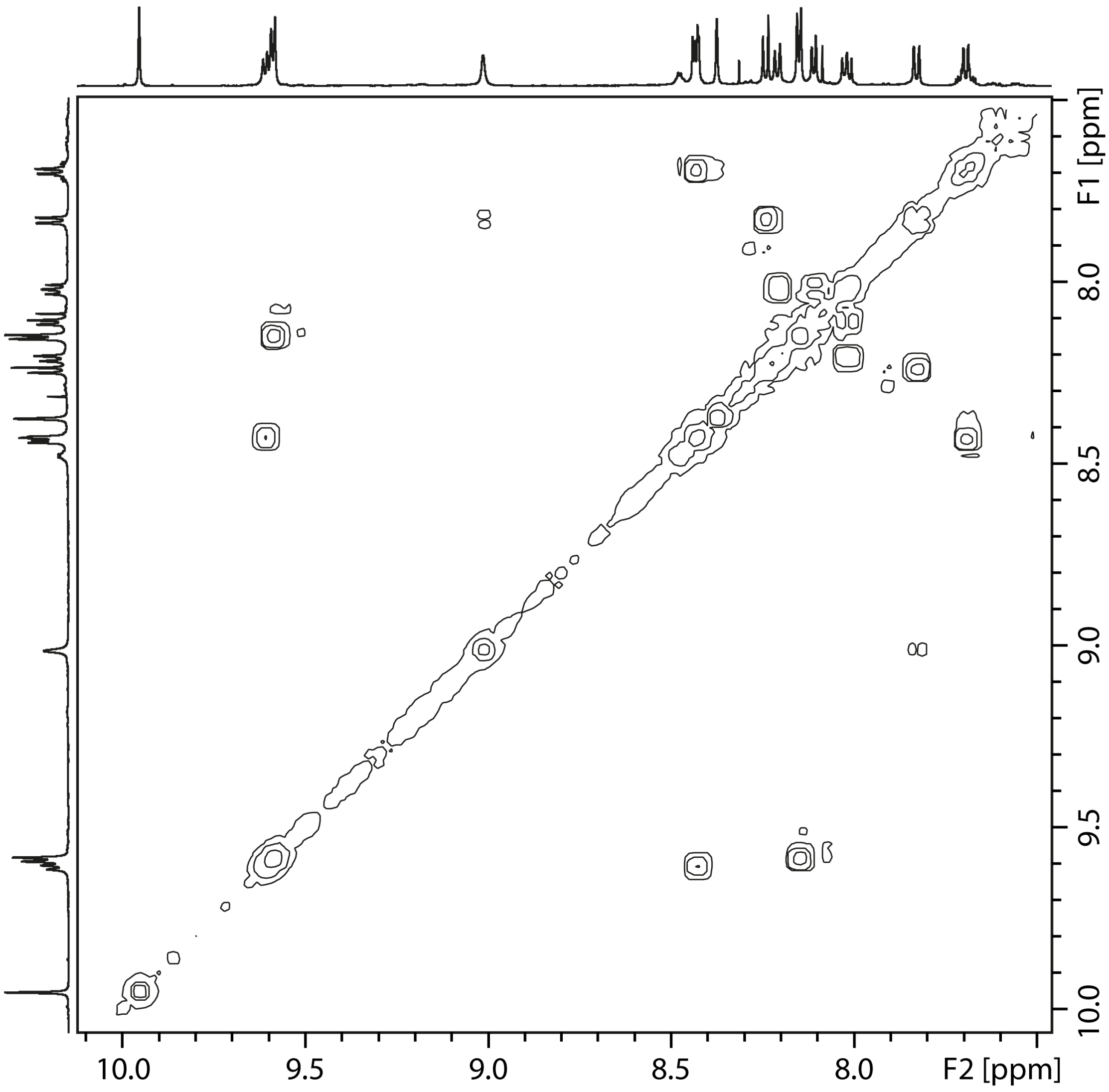

Figure S67: Partial $^{1}H-^{1}H$ COSY NMR (600 MHz, 298 K, DMSO-d$_6$) of Pd$_2$**L**$^{3}_{2}$**L**$^{A}_{2}$.

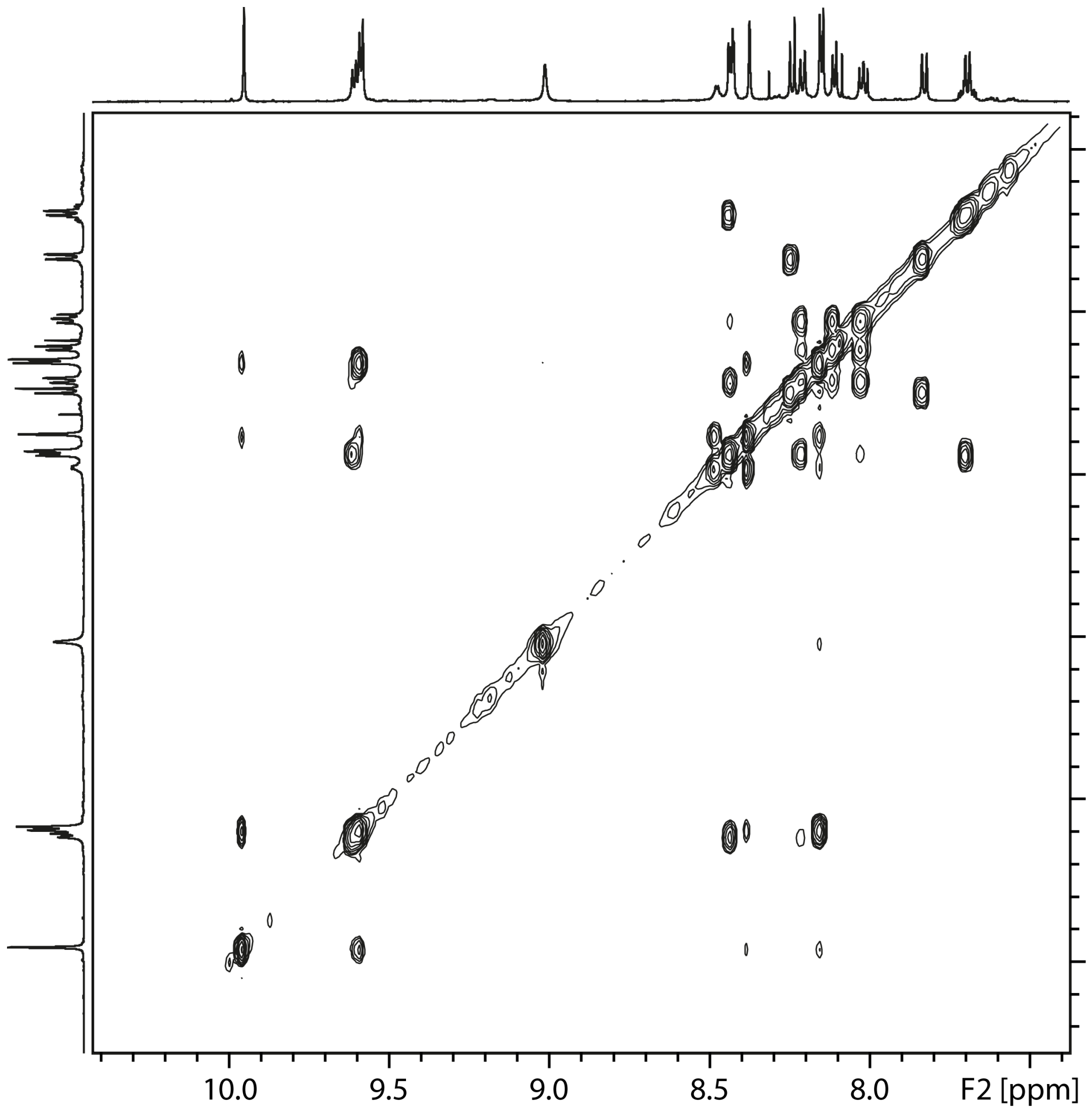

Figure S68: Partial ^{1}H–^{1}H NOESY NMR (600 MHz, 298 K, DMSO-d$_6$) of Pd$_2$**L**$^3_2$**L**$^{\mathbf{A}}_2$.

^{13}C{^{1}H} NMR (151 MHz, 298 K, DMSO-d$_6$): δ = 152.8, 151.0, 144.8, 142.3, 138.1, 136.1, 134.7, 134.2, 133.8, 131.7, 131.6, 129.8, 129.6, 129.5, 128.9, 128.7, 128.3, 127.7, 126.8, 126.2, 125.1, 124.3, 123.2, 121.6, 120.9, 120.1, 118.1, 117.4, 98.9, 97.6, 86.3, 85.1, 61.2, 13.9.

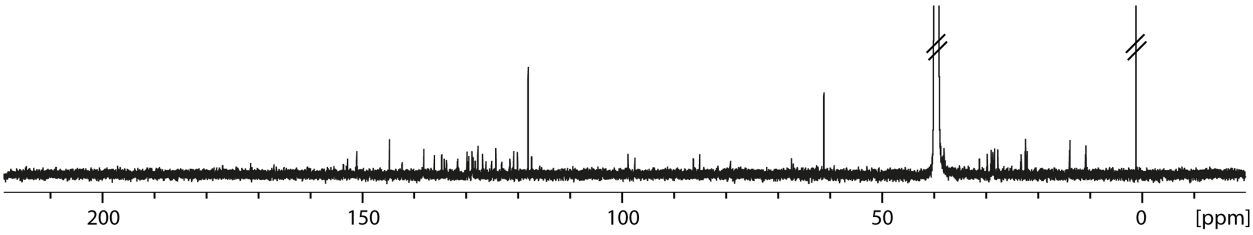

Figure S69: ^{13}C{^{1}H} NMR (151 MHz, 298 K, DMSO-d$_6$) of Pd$_2$**L**$^3_2$**L**$^{\mathbf{A}}_2$.

HR-ESI-MS (positive mode):

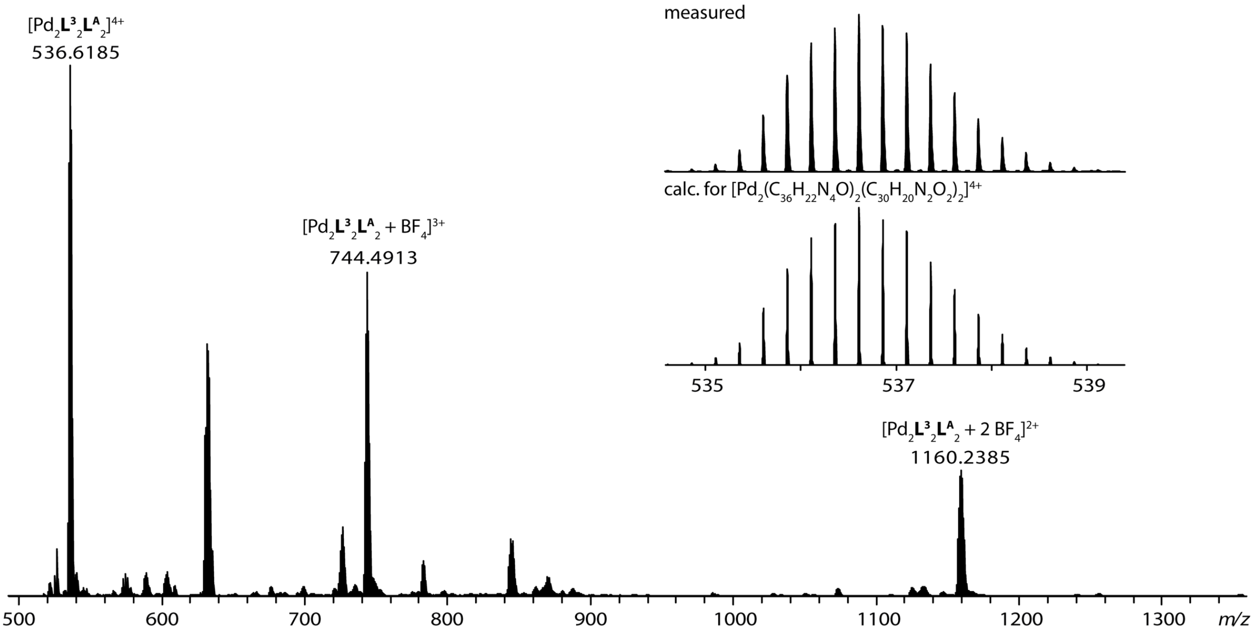

Figure S70: ESI-MS spectrum of $[Pd_2\mathbf{L}^3{}_2\mathbf{L}^A{}_2 + n\ BF_4]^{(4-n)+}$ and inset of comparison of measured and calculated mass.

2.12.8 $Pd_2L^4{}_2L^A{}_2$

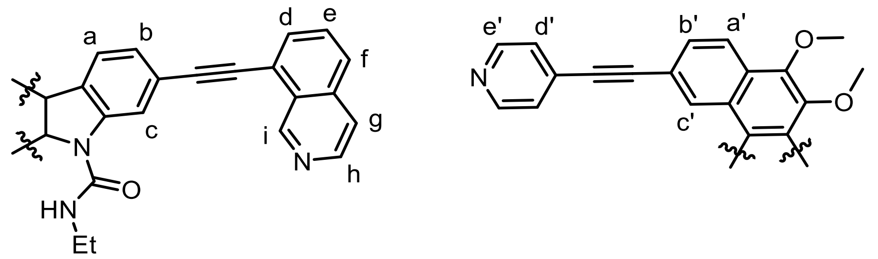

Scheme S19: Ligand Assignment for $Pd_2\mathbf{L}^1{}_2\mathbf{L}^D{}_2$.

^{1}H NMR (500 MHz, 298 K, DMSO-d6): δ = 9.97 (s, 4H, i), 9.62 (d, ^3J = 6.5, 4H, h), 9.57 (d, ^3J = 6.6, 8H, e'), 8.97 (s, 4H, c'), 8.70 (t, ^3J = 5.1, 2H, N*H*), 8.42 (m, 12H, a, c, g), 8.24 (d, ^3J = 8.6, 4H, a'), 8.20 (d, ^3J = 8.4, 4H, f), 8.12 (m, 12H, d, d'), 8.02 (t, ^3J = 8.0, 4H, e), 7.84 (d, ^3J = 8.5, 4H, b'), 7.70 (dd, ^3J = 7.9, ^4J = 1.2, 4H, b), 4.02 (s, 12H, OCH_3), 3.83 (m, 4H, NCH_2), 1.49 (t, ^3J = 7.1, 6H, NCH$_2$CH_3).

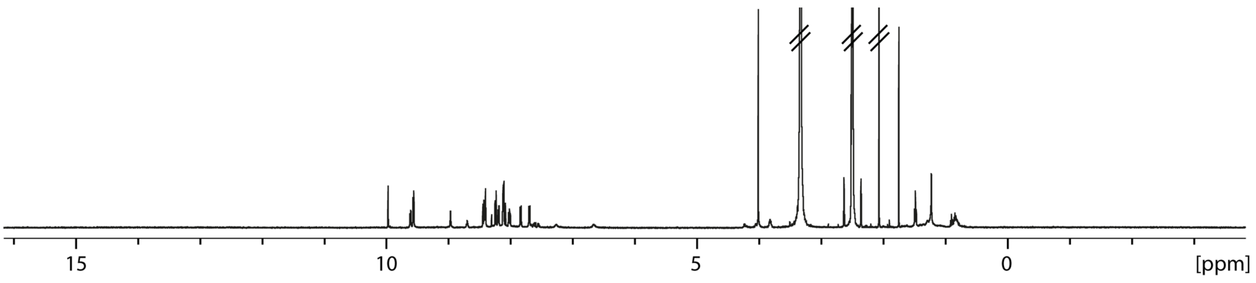

Figure S71: ^{1}H NMR (500 MHz, 298 K, DMSO-d6) of $Pd_2\mathbf{L}^4{}_2\mathbf{L}^A{}_2$.

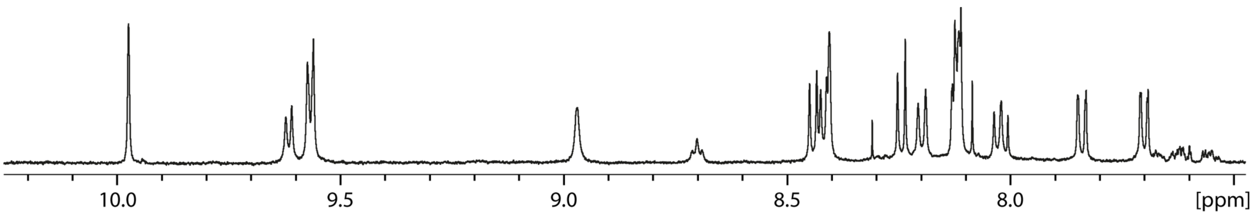

Figure S72: Partial ^{1}H NMR (500 MHz, 298 K, DMSO-d$_6$) of $Pd_2\mathbf{L^4}_2\mathbf{L^A}_2$.

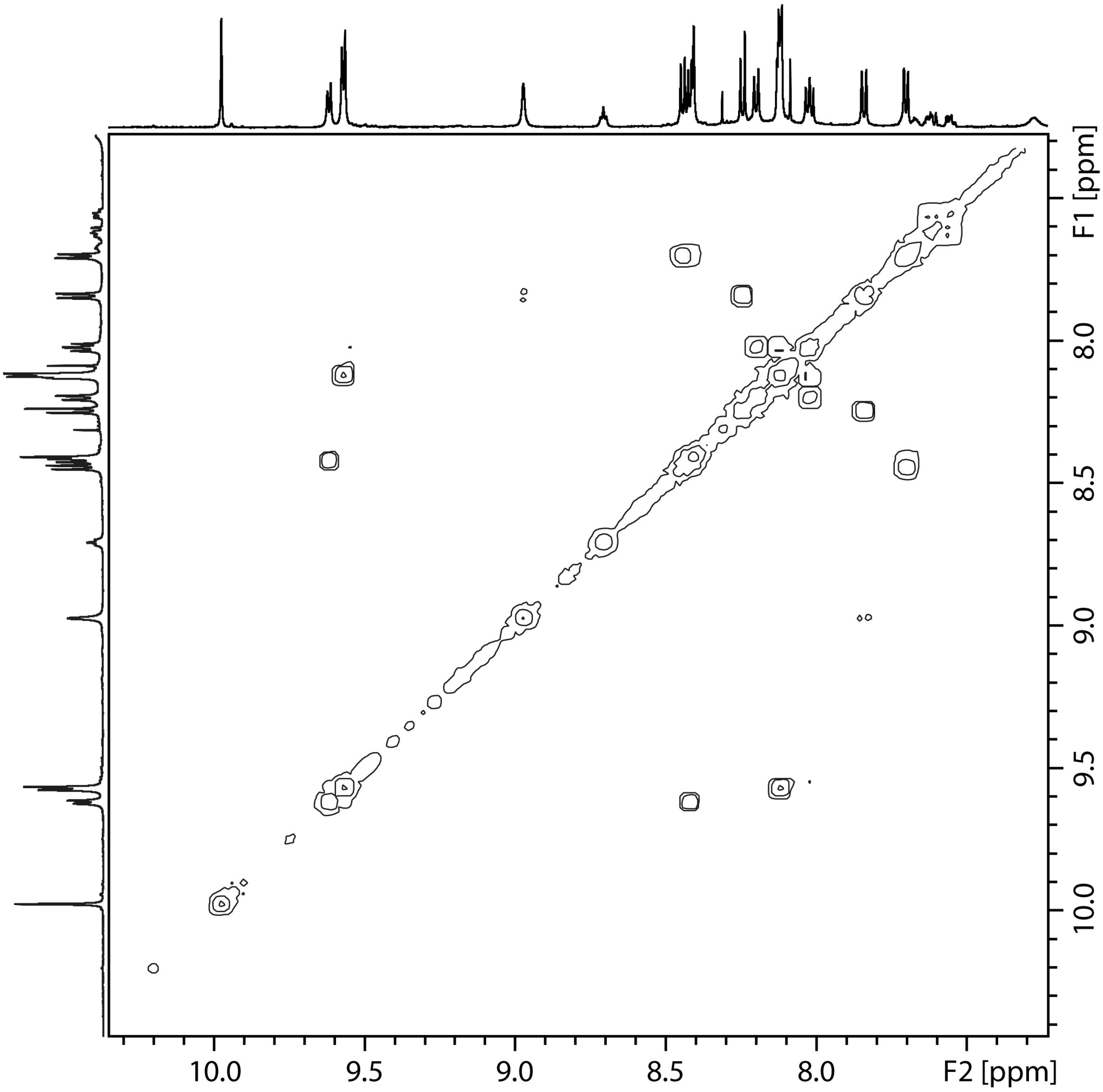

Figure S73: Partial $^{1}H-^{1}H$ COSY NMR (600 MHz, 298 K, DMSO-d$_6$) of $Pd_2\mathbf{L^4}_2\mathbf{L^A}_2$.

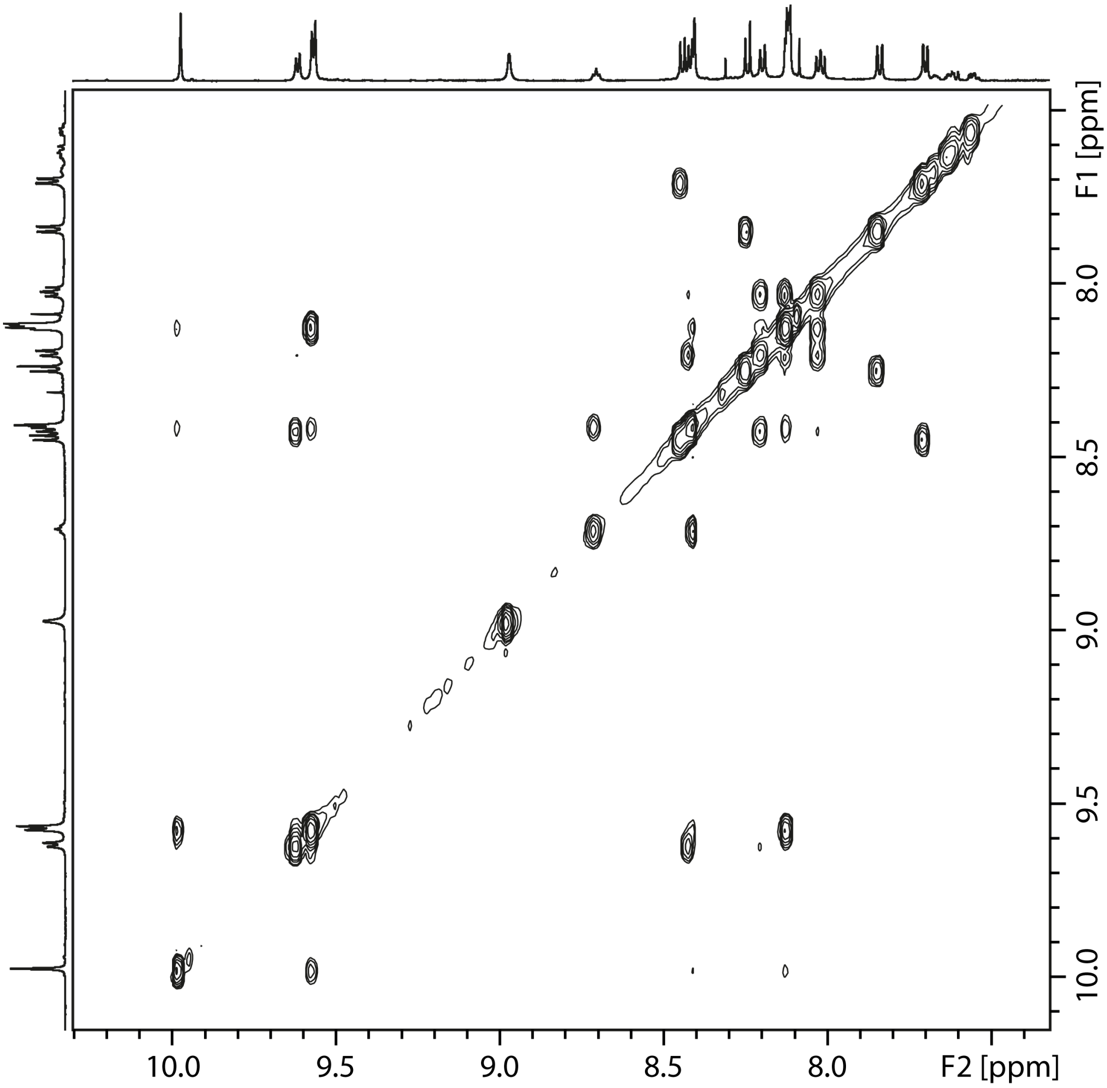

Figure S74: Partial $^{1}H-^{1}H$ NOESY NMR (600 MHz, 298 K, DMSO-d$_6$) of $Pd_2\mathbf{L}^{4}{}_2\mathbf{L}^{A}{}_2$.

$^{13}C\{^{1}H\}$ NMR (151 MHz, 298 K, DMSO-d$_6$): δ = 171.5, 153.7, 152.0, 151.0, 144.7, 142.4, 138.2, 136.1, 134.7, 134.4, 133.8, 132.1, 131.5, 131.4, 129.9, 129.7, 129.5, 128.9, 128.8, 128.7, 128.0, 127.7, 126.8, 126.2, 125.1, 124.3, 123.2, 121.7, 121.0, 120.1, 118.1, 117.5, 98.8, 97.6, 86.2, 85.3, 61.2, 22.5, 15.2.

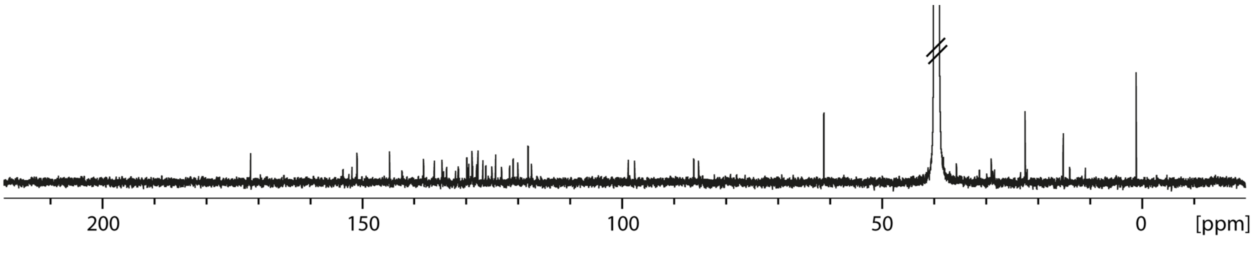

Figure S75: $^{13}C\{^{1}H\}$ NMR (151 MHz, 298 K, DMSO-d$_6$) of $Pd_2\mathbf{L}^{4}{}_2\mathbf{L}^{A}{}_2$.

HR-ESI-MS (positive mode):

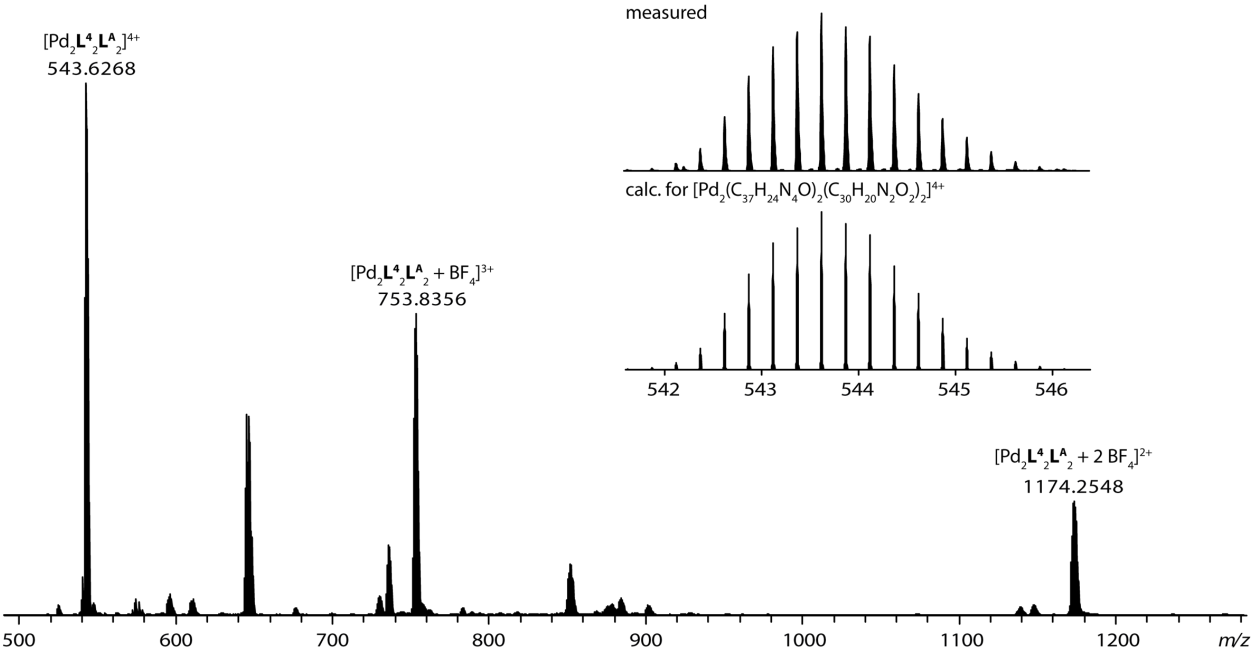

Figure S76: ESI-MS spectrum of [Pd_2**L**$^4_2$**L**$^A_2$ + n BF_4]$^{(4-n)+}$ and inset of comparison of measured and calculated mass.

2.13 General Synthesis of phosphate ester salts G^1-G^6

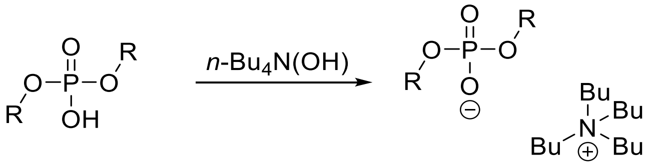

Scheme 20: General synthesis of phosphate ester salts.

100 mg of the commercially available organic phosphoric acids of the type P(O)$(OR)_2OH$ (R = Ph, Bn, Tol, Et, Bu, (*S*)-Binaphtyl) were dissolved in 50 mL HPLC-grade MeOH. A solution of tetra-*n*-butyl ammonium hydroxide (10% *ww* in HPLC-grade MeOH) was added dropwise until a neutral pH was reached. The solvent was removed *in vacuo* and the salts were dried at high vacuum for 24 h. The correct ratio of phosphate to TBA was checked via proton NMR. For titration experiments, stock solutions in DMSO-d_6 (15 mM) were used.

2.13.1 Dibutylphosphate G^1

^{1}H NMR (700 MHz, 298 K, DMSO-d$_6$):3.56 (q, ^3J = 7.42, 2H, d), 3.16 (m, 2H, NCH_2), 1.57 (m, 2H, NCH$_2$CH_2), 1.43 (m, 2H, c), 1.30 (m, 4H, b, NCH$_2$CH$_2$CH_2), 0.93 (t, ^3J = 7.31, 3H, CH_3), 0.87 (t, ^3J = 7.42, 3H, a).

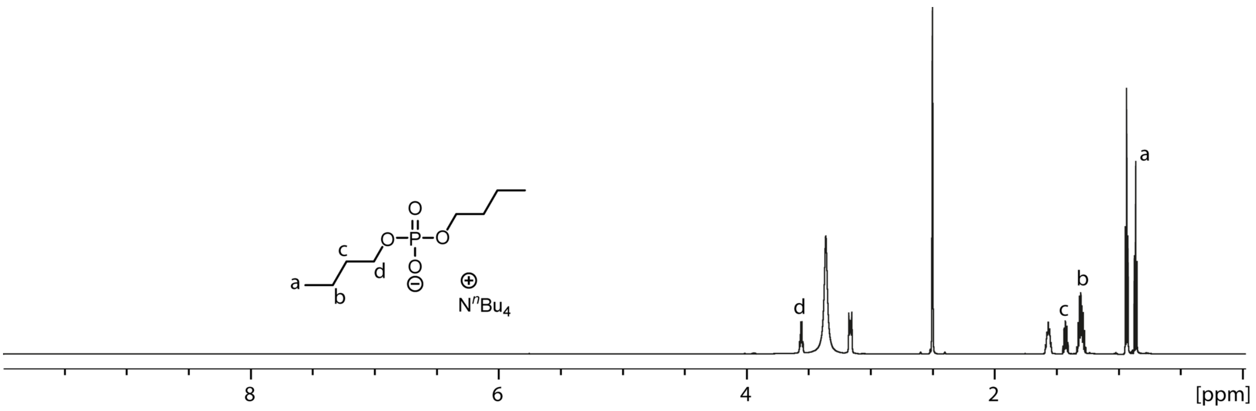

Figure S77: ^{1}H NMR spectrum (700 MHz, 298 K, DMSO-d$_6$) and structure assignment of **G**^{1}.

^{13}C{^{1}H} NMR (176 MHz, 298 K, DMSO-d$_6$): δ = 63.3, 57.5, 32.7, 23.1, 19.2, 18.7, 13.7, 13.5.

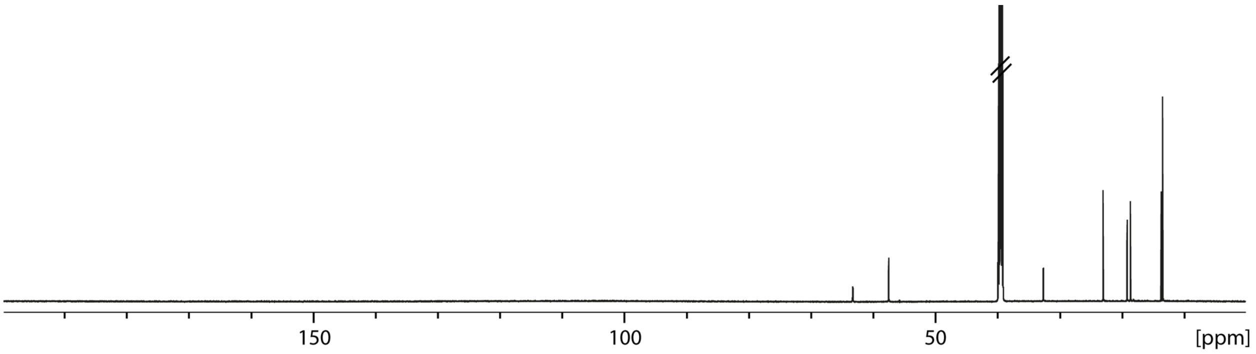

Figure S78: ^{13}C{^{1}H} NMR spectrum (176 MHz, 298 K, DMSO-d$_6$) of **G^1**.

^{31}P{^{1}H} NMR (283 MHz, 298 K, DMSO-d$_6$, lb = 5 Hz): δ = −1.0.

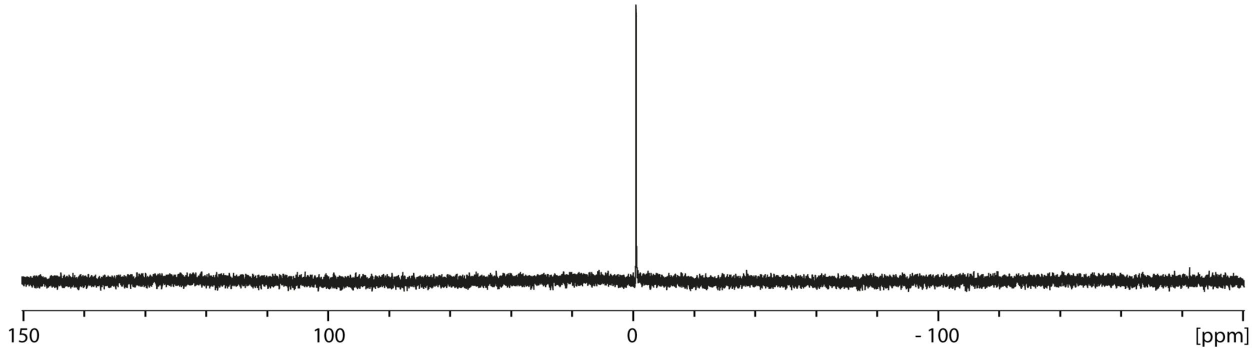

Figure S79: ^{31}P{^{1}H} NMR spectrum (283 MHz, 298 K, DMSO-d$_6$, lb = 5 Hz) of **G^1**.

2.13.2 Dibenzylphosphate G^2

^{1}H NMR (700 MHz, 298 K, DMSO-d$_6$): δ = 7.30 (m, 4H, b, c), 7.22 (m, 1H, a), 4.7 (d, 2H, ^2J = 6.46, d), 3.16 (m, 2H, NCH_2), 1.56 (m, 2H, NCH$_2$CH_2), 1.30 (m, 2H, NCH$_2$CH$_2$CH_2), 0.93 (t, ^3J = 7.30, 3H, CH_3).

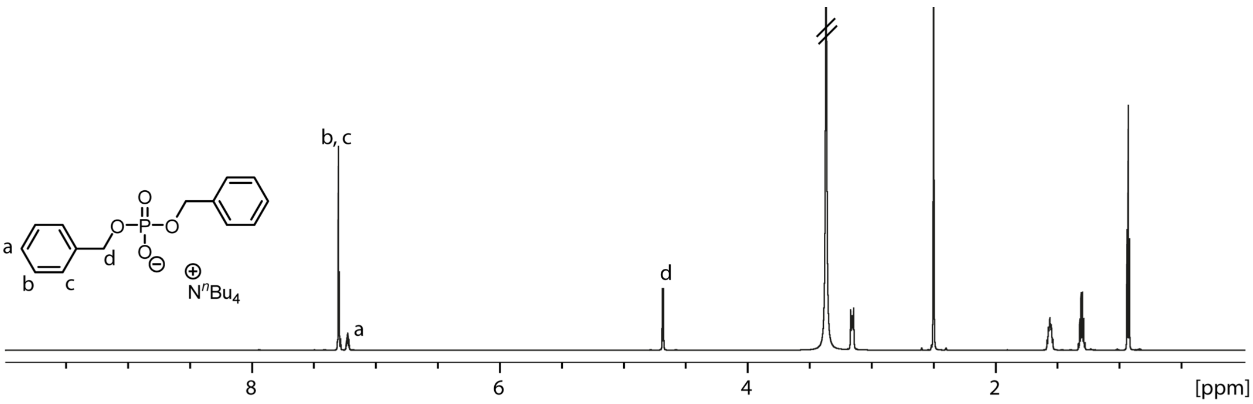

Figure S80: ^{1}H NMR spectrum (700 MHz, 298 K, DMSO-d$_6$) and structure assignment of **G^2**.

^{13}C{^{1}H} NMR (176 MHz, 298 K, DMSO-d$_6$): δ = 139.9, 128.0, 127.0, 126.8, 65.7, 57.5, 23.0, 19.2, 13.5.

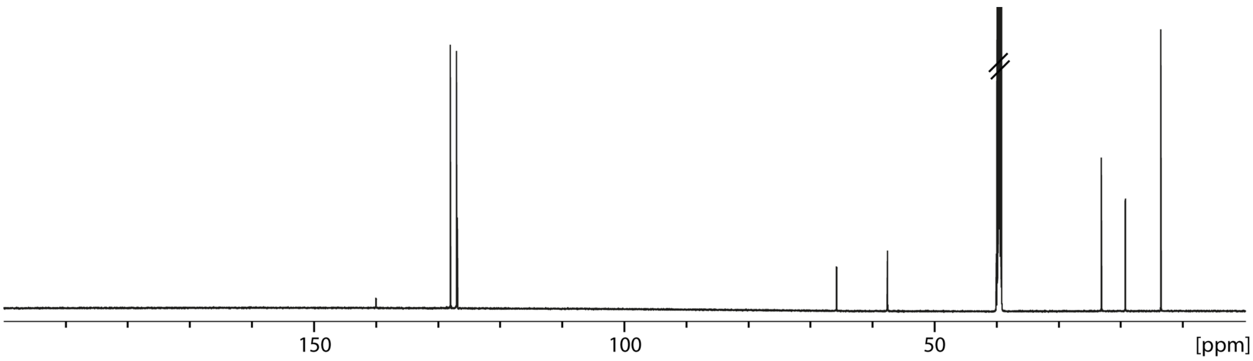

Figure S81: ^{13}C{^{1}H} NMR spectrum (176 MHz, 298 K, DMSO-d$_6$) of **G^2**.

^{31}P{^{1}H} NMR (283 MHz, 298 K, DMSO-d$_6$, lb = 5 Hz): δ = −0.8.

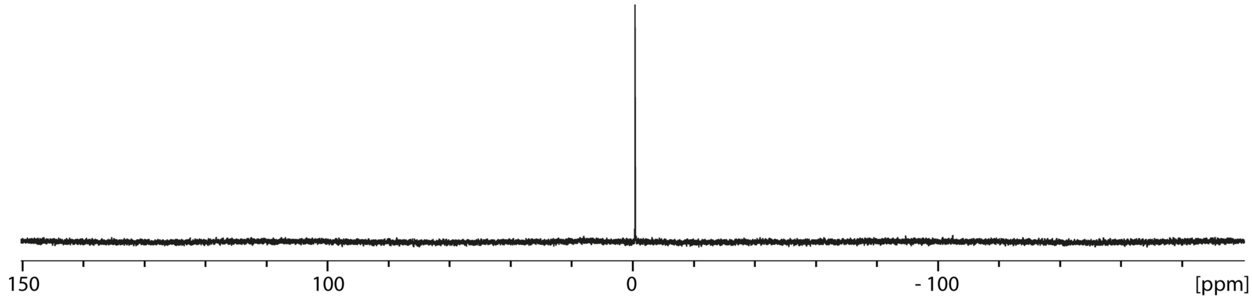

Figure S82: ^{31}P{^{1}H} NMR spectrum (283 MHz, 298 K, DMSO-d$_6$, lb = 5 Hz) of **G^2**.

2.13.3 Diphenylphosphate G^3

^{1}H NMR (700 MHz, 298 K, DMSO-d$_6$): δ = 7.20 (m, 2H, b), 7.11 (m, 2H, c), 6.93 (t, ^3J = 7.30, 1H, a), 3.16 (m, 2H, NCH_2), 1.56 (m, 2H, NCH$_2$CH_2), 1.31 (tq, ^3J = 7.31, 2H, NCH$_2$CH$_2$CH_2), 0.93 (t, ^3J = 7.31, 3H, CH_3).

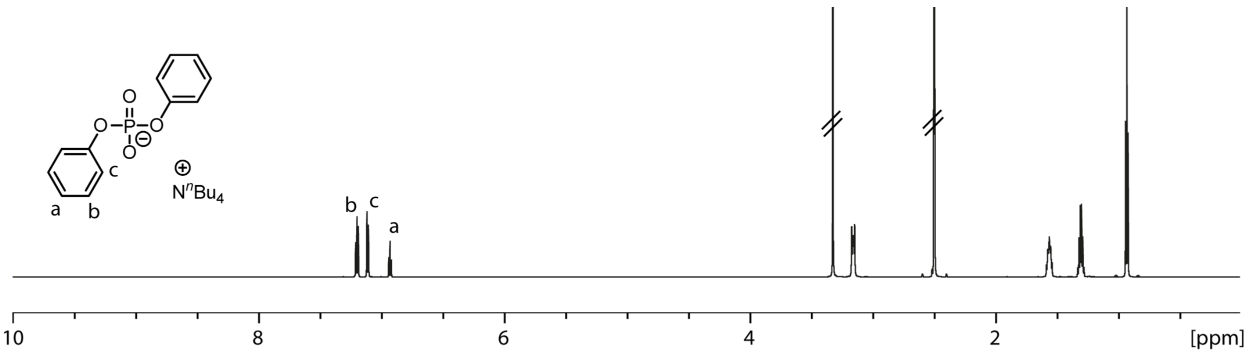

Figure S83: ^{1}H NMR spectrum (700 MHz, 298 K, DMSO-d$_6$) and structure assignment of **G**^{3}.

$^{13}C\{^{1}H\}$ **NMR** (176 MHz, 298 K, DMSO-d$_6$): δ = 154.1, 128.7, 121.6, 119.8, 57.5, 23.0, 19.2, 13.5.

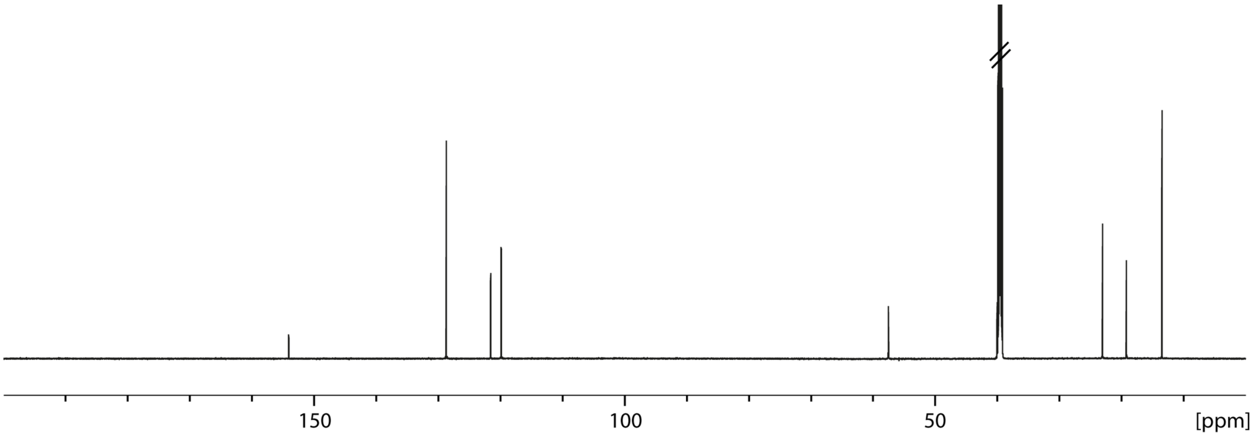

Figure S84: $^{13}C\{^{1}H\}$ NMR spectrum (176 MHz, 298 K, DMSO-d$_6$) of **G**^{3}.

$^{31}P\{^{1}H\}$ **NMR** (283 MHz, 298 K, DMSO-d$_6$, lb = 5 Hz): $\delta = -11.3$.

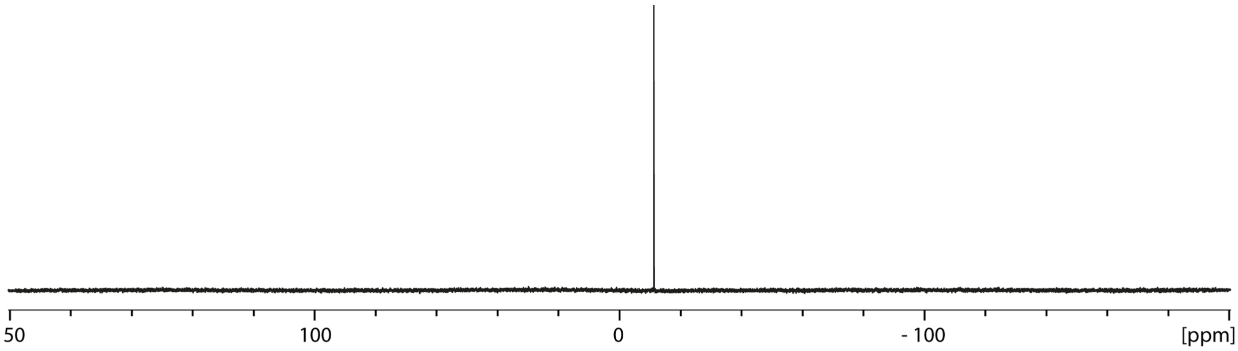

Figure S85: $^{31}P\{^{1}H\}$ NMR spectrum (283 MHz, 298 K, DMSO-d$_6$, lb = 5 Hz) of **G**^{3}.

2.13.4 Diethylphosphate G^{4}

^{1}**H NMR** (700 MHz, 298 K, DMSO-d$_6$): δ = 3.60 (m, 2H, b), 3.16 (m, 2H, NCH_2), 1.57 (m, 2H, NCH$_2$CH_2), 1.31 (m, 2H, NCH$_2$CH$_2$CH_2), 1.06 (t, ^{3}J = 7.05, 3H, a), 0.93 (t, ^{3}J = 7.31, 3H, CH_3).

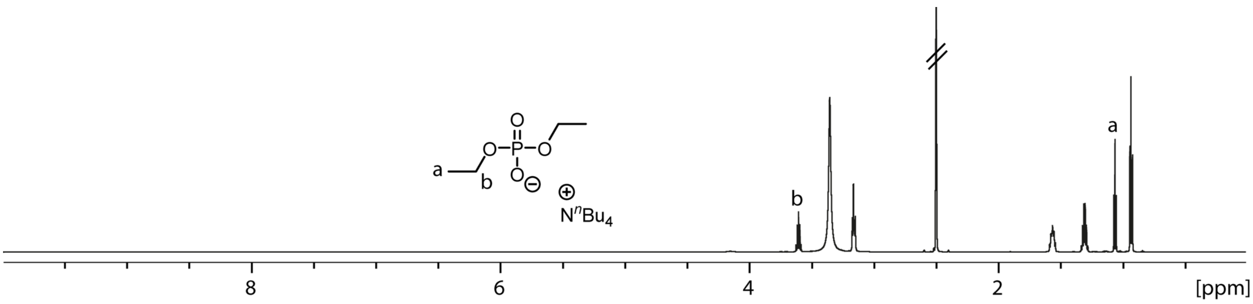

Figure S86: ^{1}H NMR spectrum (700 MHz, 298 K, DMSO-d$_6$) and structure assignment of **G^4**.

^{13}C{^{1}H} NMR (176 MHz, 298 K, DMSO-d$_6$): δ = 59.0, 57.5, 23.1, 19.2, 16.7, 13.5.

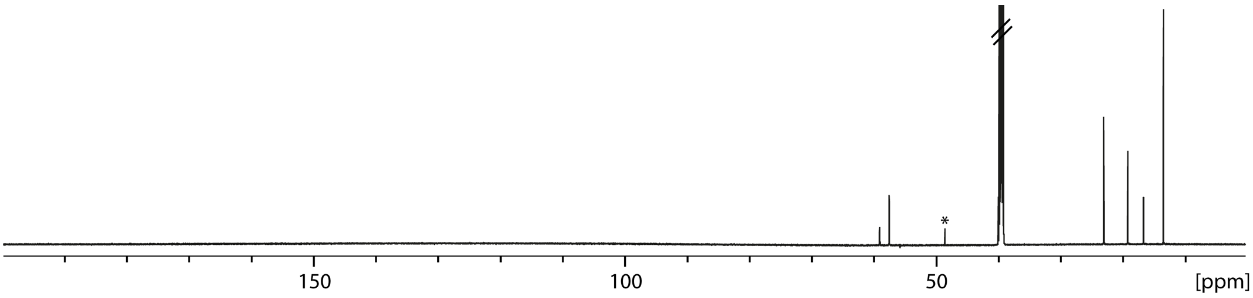

Figure S87: ^{13}C{^{1}H} NMR spectrum (176 MHz, 298 K, DMSO-d$_6$) of **G^4**, solvent residue signal (MeOH) marked with a star *.

^{31}P{^{1}H} NMR (283 MHz, 298 K, DMSO-d$_6$, lb = 5 Hz): δ = −1.0.

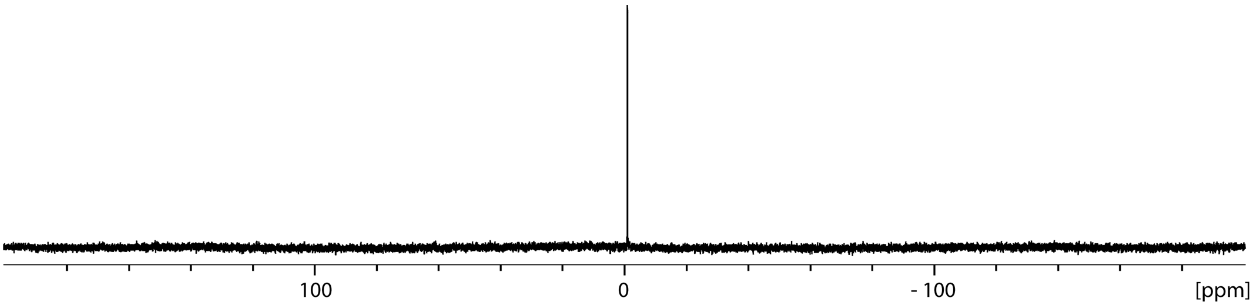

Figure S88: ^{31}P{^{1}H} NMR spectrum (283 MHz, 298 K, DMSO-d$_6$, lb = 5 Hz) of **G^4**.

2.13.5 Di-*p*-toluylphosphate G^5

^{1}H NMR (700 MHz, 298 K, DMSO-d$_6$): δ = 6.98 (s, 4H, b, c), 3.16 (m, 2H, NCH_2), 2.20 (s, 3H, a), 1.56 (m, 2H, NCH$_2$CH_2), 1.31 (m, 2H, NCH$_2$CH$_2$CH_2), 0.93 (t, ^3J = 7.31, 3H, CH_3).

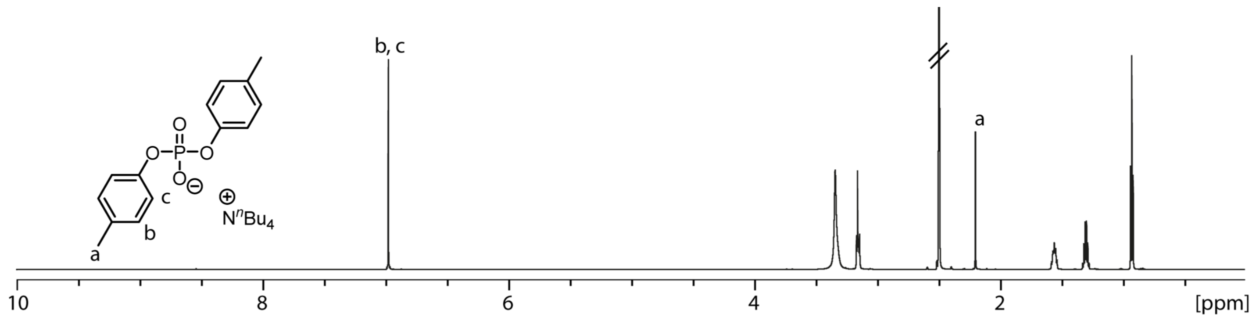

Figure S89: ^{1}H NMR spectrum (700 MHz, 298 K, DMSO-d$_6$) and structure assignment of **G^5**.

^{13}C{^{1}H} NMR (176 MHz, 298 K, DMSO-d$_6$): δ = 151.9, 130.1, 129.0, 119.8, 57.5, 23.1, 20.2, 19.2, 13.5.

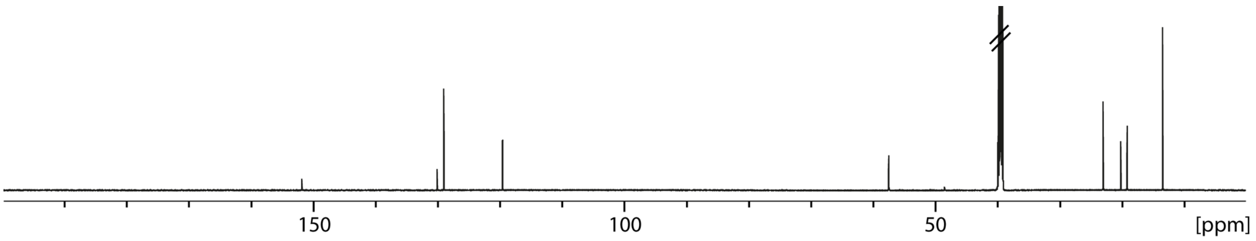

Figure 90: ^{13}C{^{1}H} NMR spectrum (176 MHz, 298 K, DMSO-d$_6$) of **G^5**.

^{31}P{^{1}H} NMR (283 MHz, 298 K, DMSO-d$_6$, lb = 5 Hz): δ = −11.1.

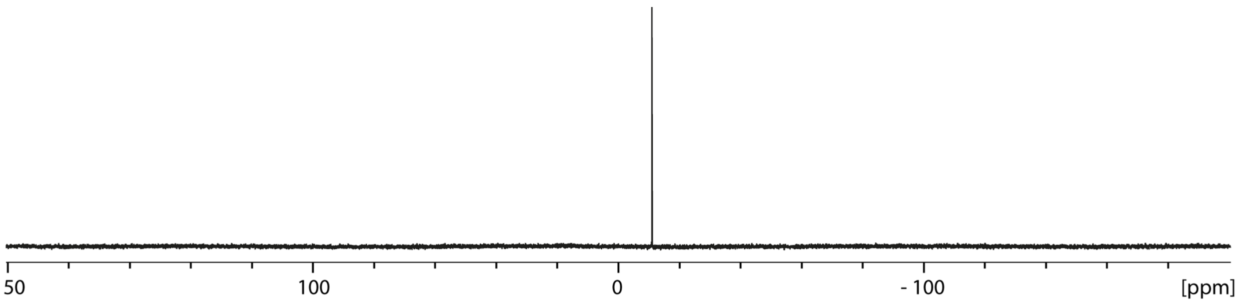

Figure S91: ^{31}P{^{1}H} NMR spectrum (283 MHz, 298 K, DMSO-d$_6$, lb = 5 Hz) of **G^5**.

2.13.6 (*S*)-Binaphtylphosphate G^6

^{1}H NMR (700 MHz, 298 K, DMSO-d$_6$): δ = 8.01 (m, 4H, b, c), 7.42 (m, 2H, e), 7.38 (d, ^{3}J = 8.69, 2H, a), 7.28 (m, 2H, d), 7.20 (d, ^{3}J = 8.50, 2H, f), 3.16 (m, 2H, NCH_2), 1.56 (m, 2H, NCH$_2$CH_2), 1.31 (m, 2H, NCH$_2$CH$_2$CH_2), 0.93 (t, ^{3}J = 7.31, 3H, CH_3).

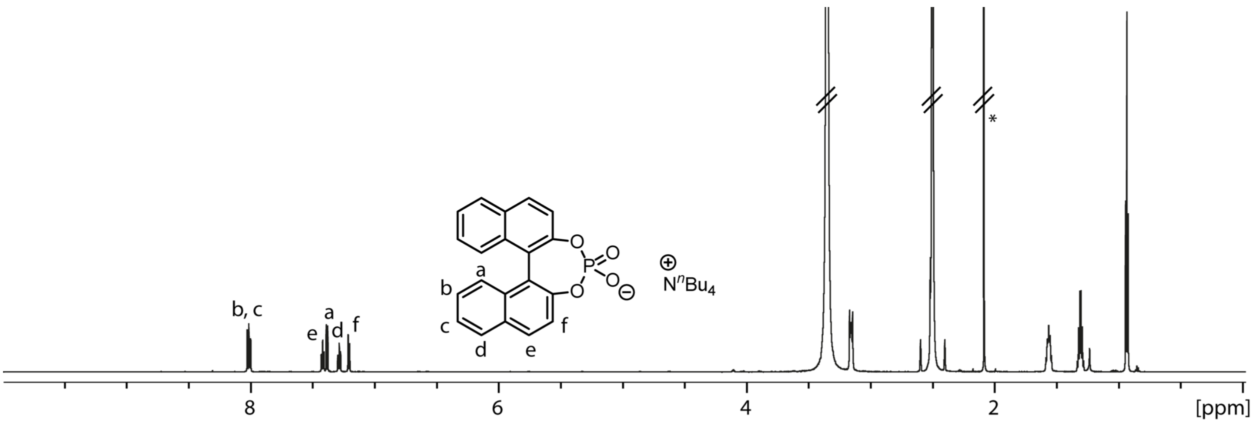

Figure S92: ^{1}H NMR spectrum (700 MHz, 298 K, DMSO-d$_6$) and structure assignment of **G^6**, solvent residue signal (acetone) marked with a star *.

^{13}C{^{1}H} NMR (176 MHz, 298 K, DMSO-d$_6$): δ = 150.3, 132.0, 130.2, 129.6, 128.4, 126.0, 125.9, 124.3, 122.8, 121.8, 57.5, 20.2, 19.2, 13.5.

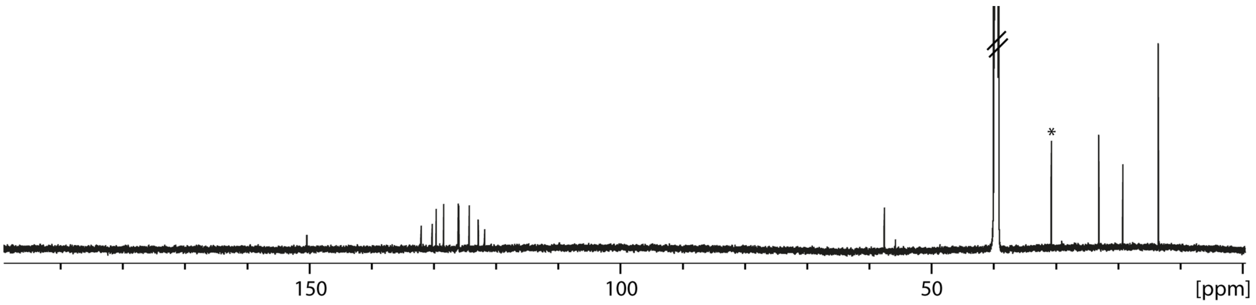

Figure S93: ^{13}C{^{1}H} NMR spectrum (176 MHz, 298 K, DMSO-d$_6$) of **G^6**, solvent residue signal (acetone) marked with a star *.

^{31}P{^{1}H} NMR (283 MHz, 298 K, DMSO-d$_6$, lb = 5 Hz): δ = 5.2.

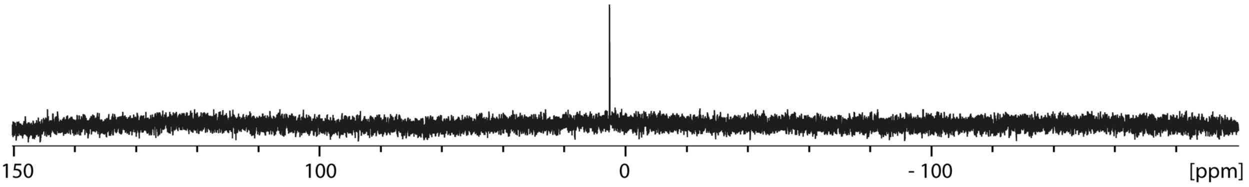

Figure S94: ^{31}P{^{1}H} NMR spectrum (283 MHz, 298 K, DMSO-d$_6$, lb = 5 Hz) of **G^6**.

3 Guest Titration Experiments

^{1}H NMR Titration experiments were carried out in the following way: The guest solution (15 mM in DMSO-d$_6$) was added to the host solution (0.7 mM in DMSO-d$_6$) in steps of 0.2 eq (until reaching 2 eq in total, then 2.5, 3.0, 3.5, 4.0 and 5.0 eq, if not mentioned otherwise). After adding, the sample was shaken briefly and measured directly. Owing to clarity, only every second spectrum is shown in the following figures.

3.1 Titration of G^1 to $Pd_2L^1{}_2L^A{}_2$

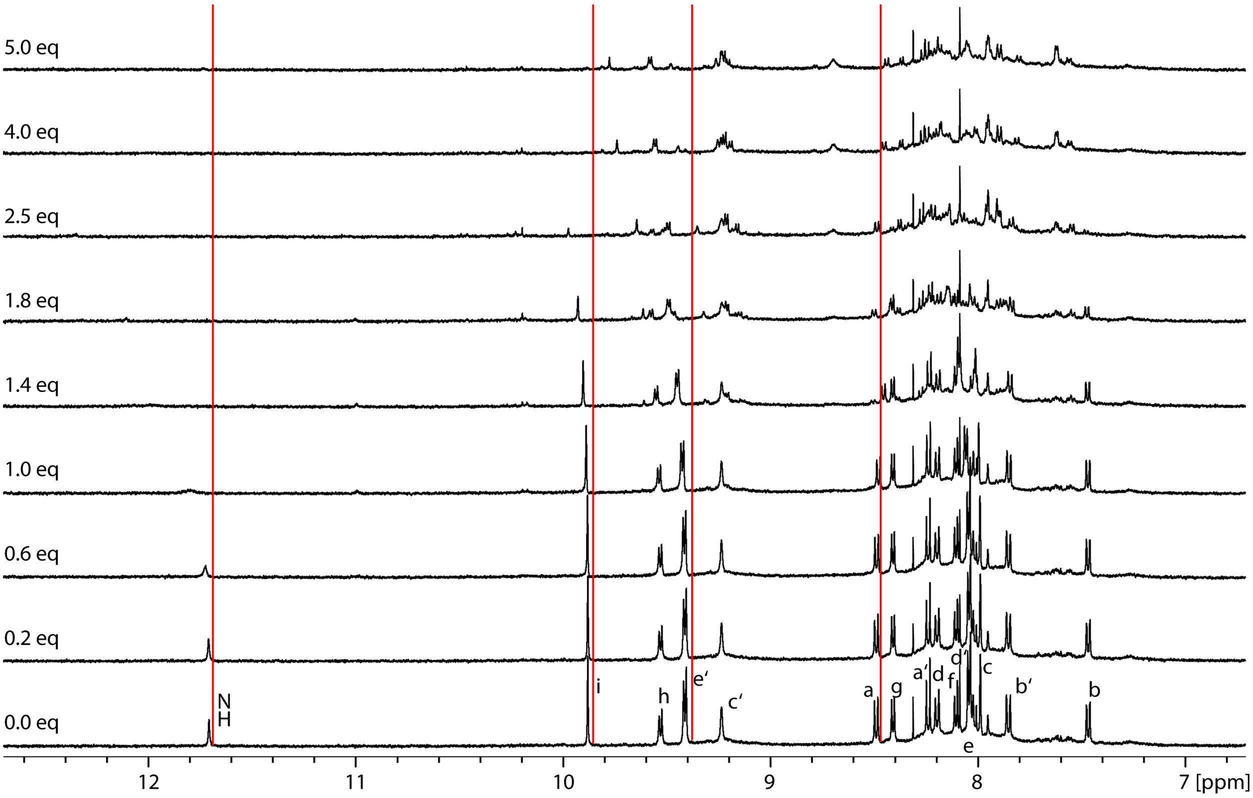

Figure S95: Stacked partial ^{1}H NMR spectra (500 MHz, 298 K, DMSO-d$_6$) of **G^1**@Pd_2**L^1**$_2$**L^A**$_2$. Beyond addition of the first guest equivalent, signals for new species appear in case of the aliphatic guests that may point to formation of a host:guest 1:2 complex or an assembly loosing a ligand under growing competition of the anionic guest at high concentrations.

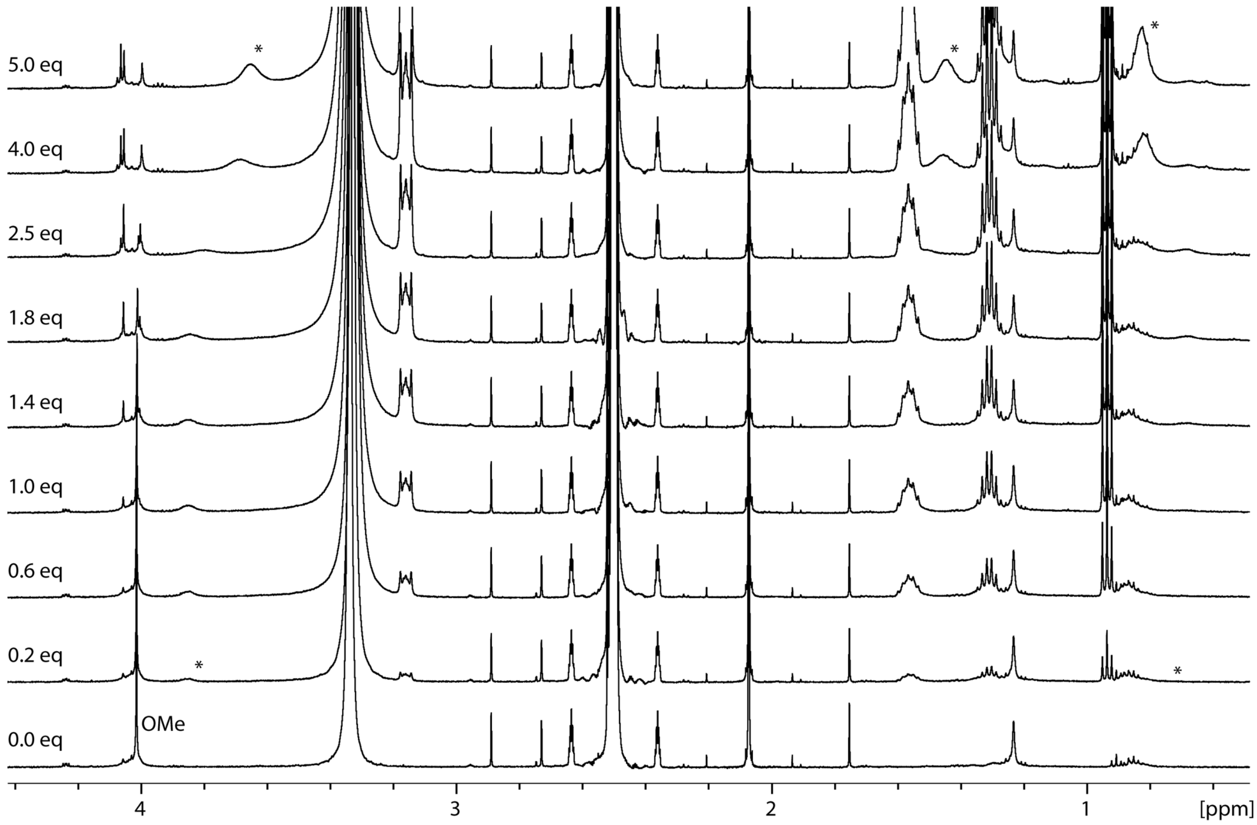

Figure S96: Stacked partial ^{1}H NMR spectra (500 MHz, 298 K, DMSO-d$_6$) of $\mathbf{G^1}$@Pd$_2$$\mathbf{L^1}_2$$\mathbf{L^A}_2$. Guest signals are indicated with a star *.

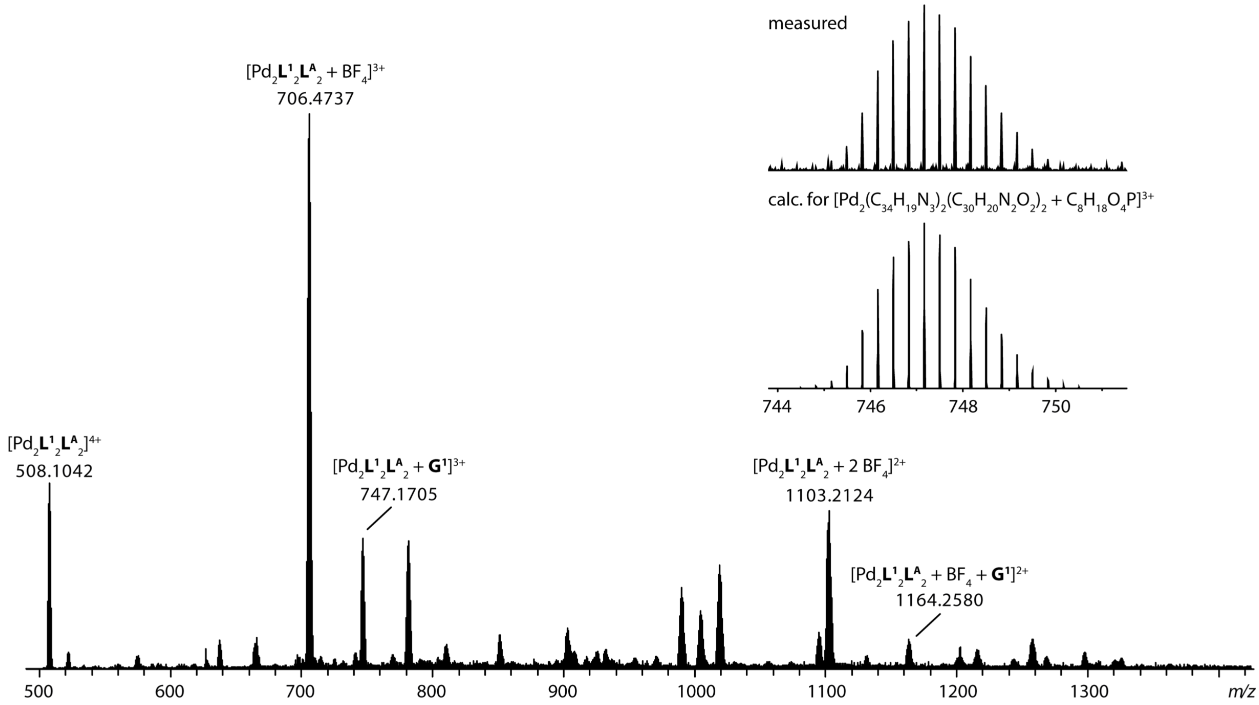

Figure S97: ESI-MS spectrum of [Pd$_2$$\mathbf{L^1}_2$$\mathbf{L^A}_2$ + m $\mathbf{G^1}$ + n BF$_4$]$^{(4-[m+n])+}$ and inset of comparison of measured and calculated mass.

3.2 Titration of G^2 to $Pd_2L^1{}_2L^A{}_2$

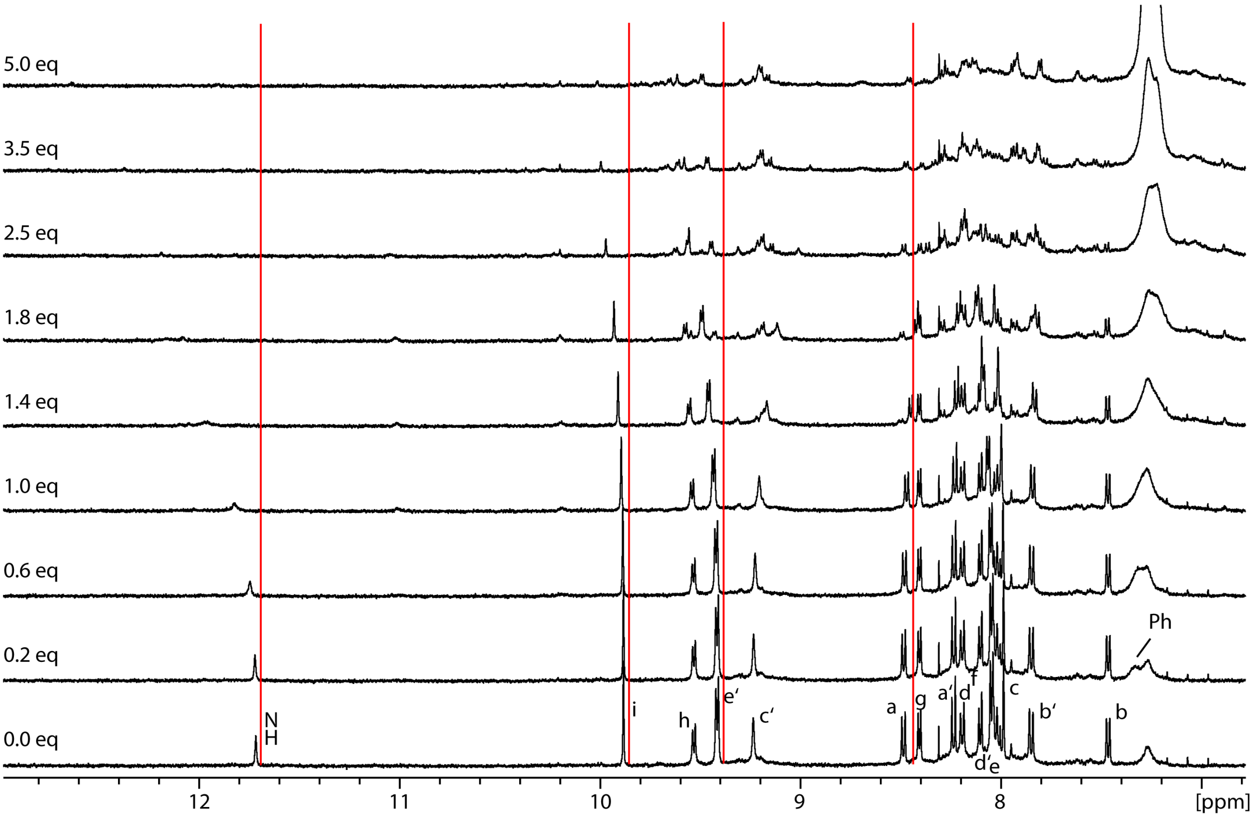

Figure S98: Stacked partial ^{1}H NMR spectra (500 MHz, 298 K, DMSO-d$_6$) of **G**^{2}@Pd$_2$**L**$^1{}_2$**L**$^A{}_2$.

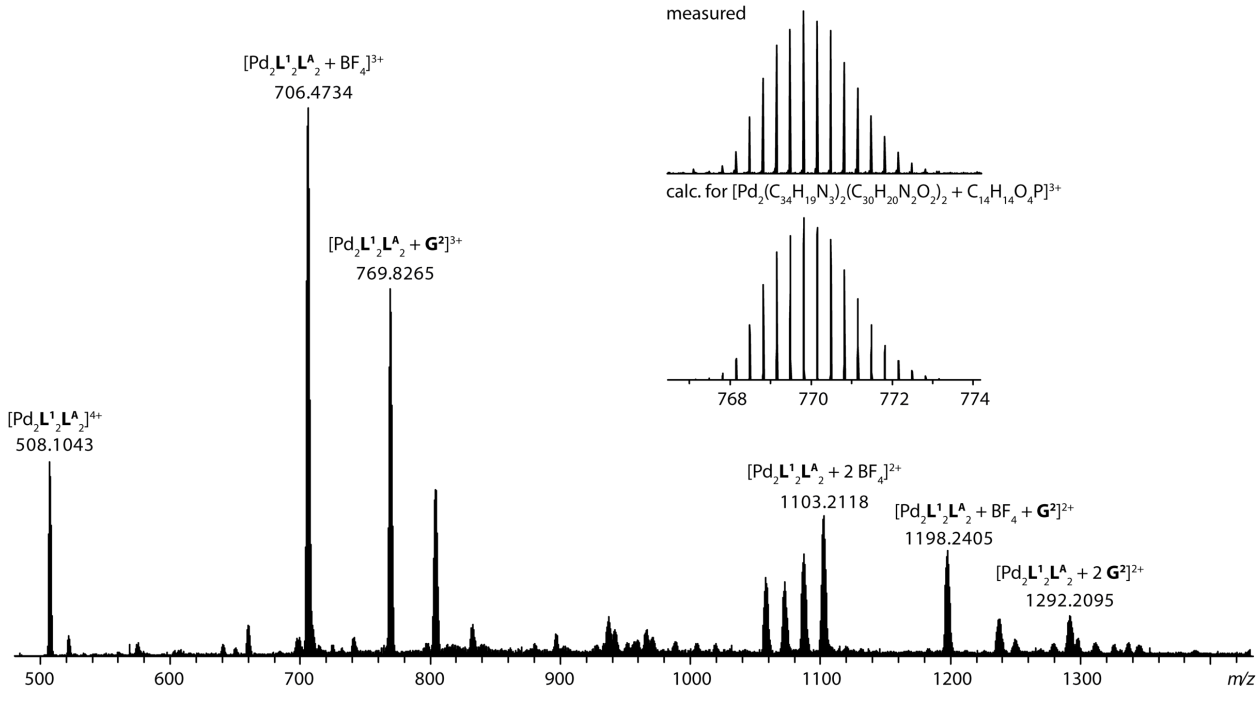

Figure S99: ESI-MS spectrum of [Pd$_2$**L**$^1{}_2$**L**$^A{}_2$ + m **G**^{2} + n BF$_4$]$^{(4-[m+n])+}$ and inset of comparison of measured and calculated mass.

3.3 Titration of G^3 to $Pd_2L^1{}_2L^A{}_2$

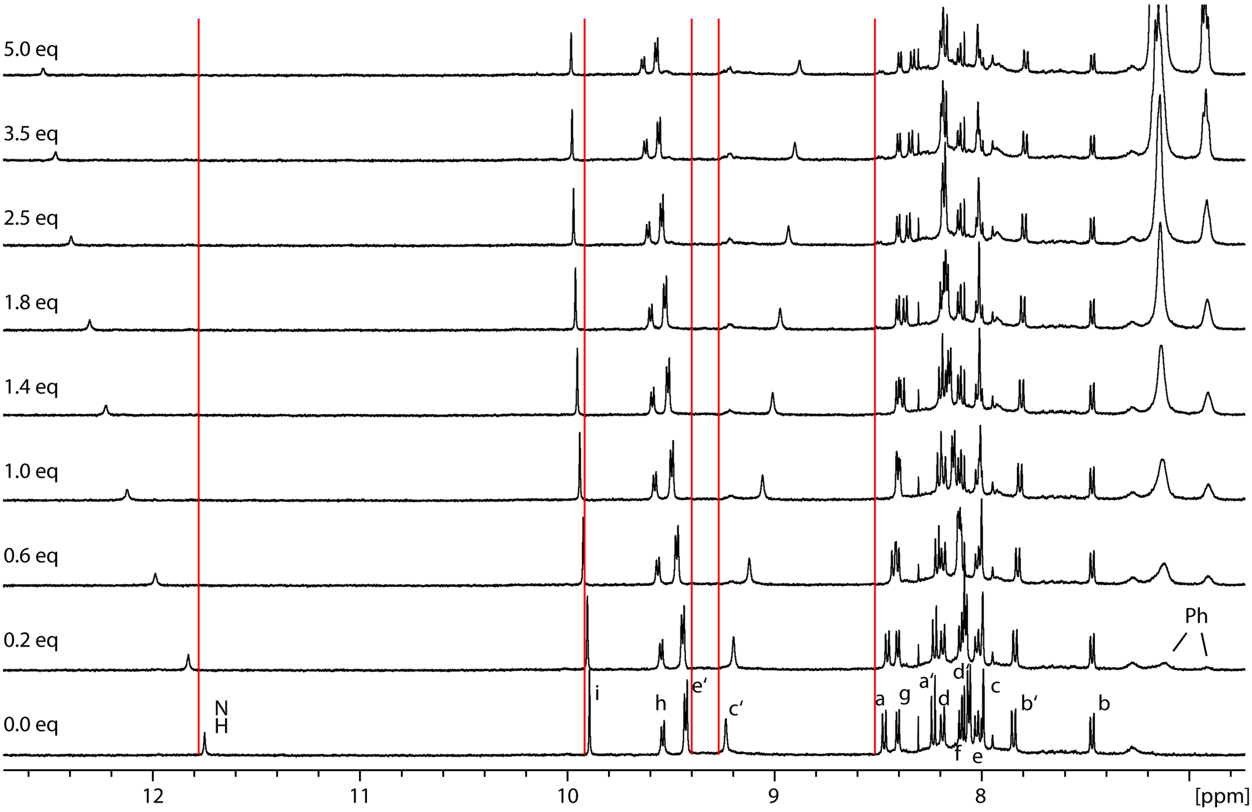

Figure S100: Stacked partial ^{1}H NMR spectra (500 MHz, 298 K, DMSO-d_6) of **G^3**@Pd_2**L^1**$_2$**L^A**$_2$.

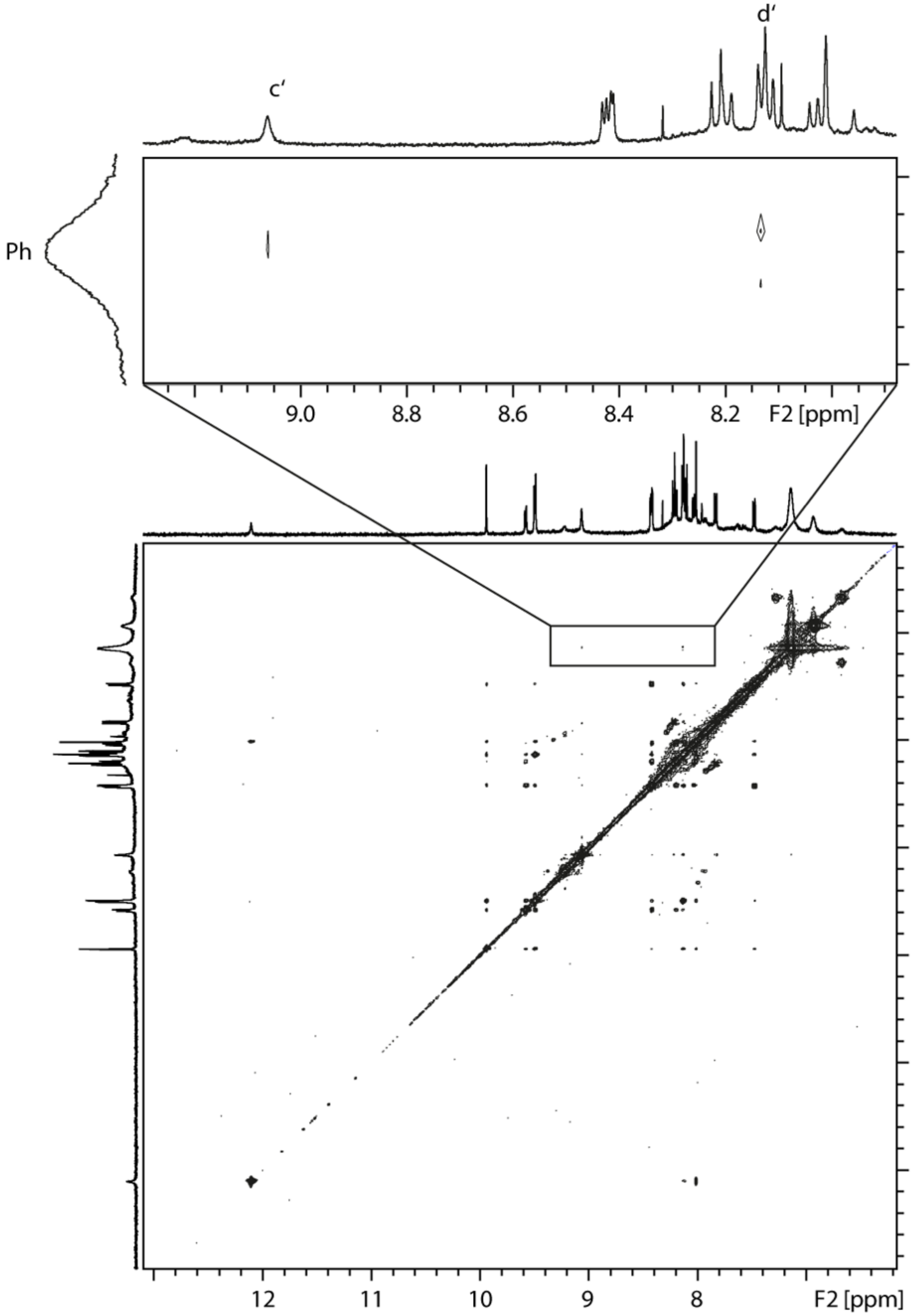

Figure S101: Partial $^1H-^1H$ NOESY NMR (500 MHz, 298 K, DMSO-d$_6$) of 1 eq $\mathbf{G^3}$@Pd$_2$$\mathbf{L^1}_2$$\mathbf{L^A}_2$ with highlight to visualize NOE crosspeaks between phenyl proton signals of $\mathbf{G^3}$ and inward pointing proton signals c' and d' of ligand $\mathbf{L^A}$ showing that the guest is encapsulated in solution and that its phenyl substituents are located close to ligand $\mathbf{L^A}$ in accordance with the MD simulation results.

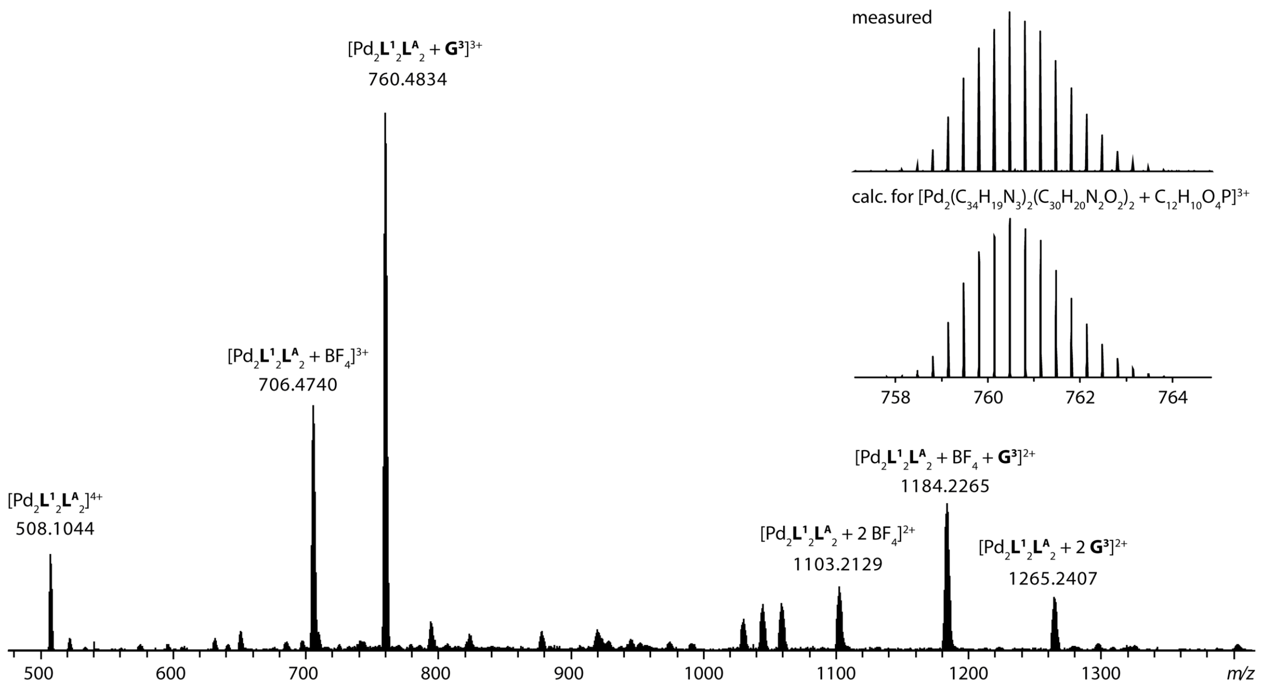

Figure S102: ESI-MS spectrum of $[Pd_2\mathbf{L}^1{}_2\mathbf{L}^A{}_2 + m\ \mathbf{G}^3 + n\ BF_4]^{(4-[m+n])+}$ and inset of comparison of measured and calculated mass.

3.4 Titration of G^4 to $Pd_2L^1{}_2L^A{}_2$

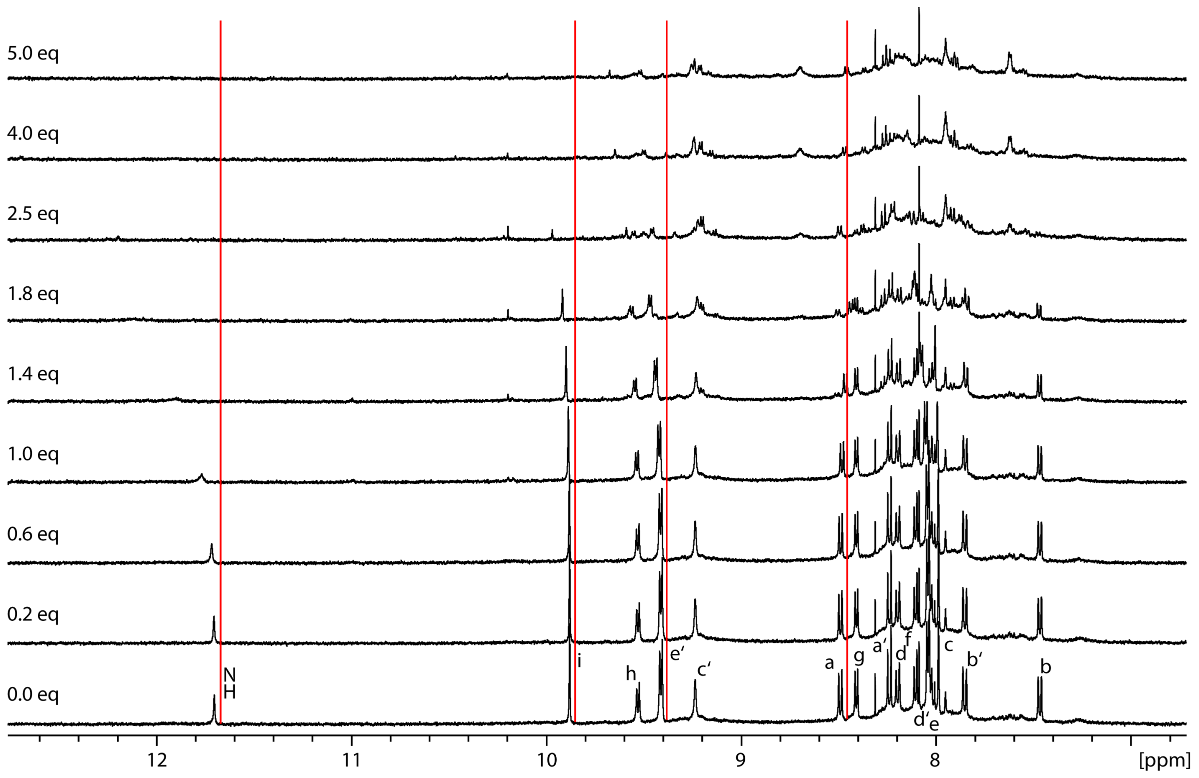

Figure S103: Stacked partial ^1H NMR spectra (500 MHz, 298 K, DMSO-d_6) of $\mathbf{G}^4@Pd_2\mathbf{L}^1{}_2\mathbf{L}^A{}_2$.

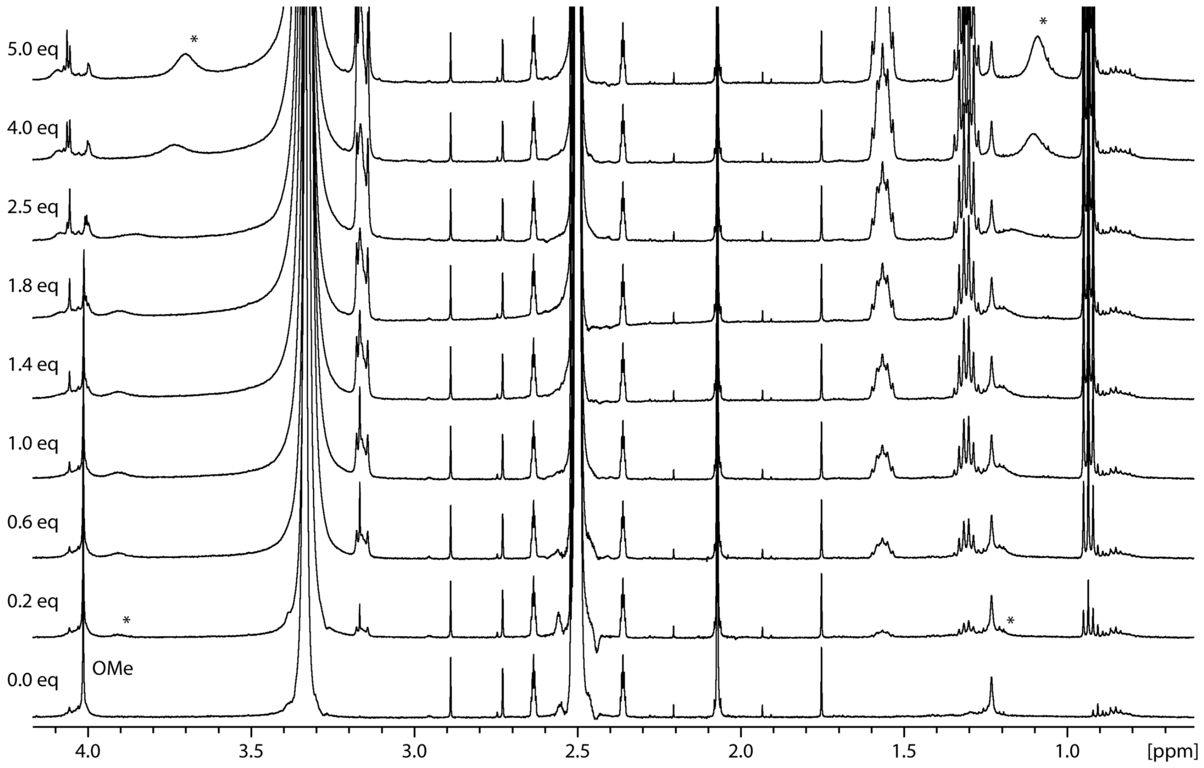

Figure S104: Stacked partial ^{1}H NMR spectra (500 MHz, 298 K, DMSO-d$_6$) of $\mathbf{G^4}$@Pd$_2$$\mathbf{L^1}_2$$\mathbf{L^A}_2$. Guest signals are indicated with a star *.

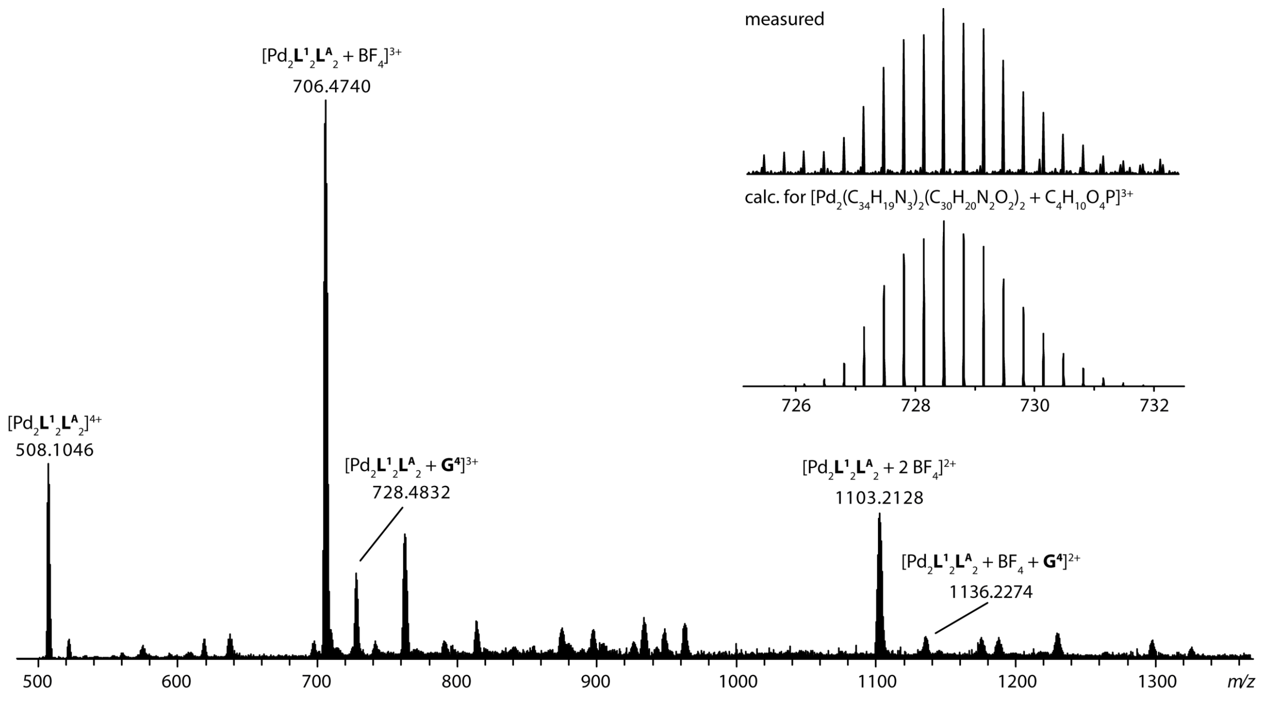

Figure S105: ESI-MS spectrum of [Pd$_2$$\mathbf{L^1}_2$$\mathbf{L^A}_2$ + m $\mathbf{G^4}$ + n BF$_4$]$^{(4-[m+n])+}$ and inset of comparison of measured and calculated mass.

3.5 Titration of G^5 to $Pd_2L^1{}_2L^A{}_2$

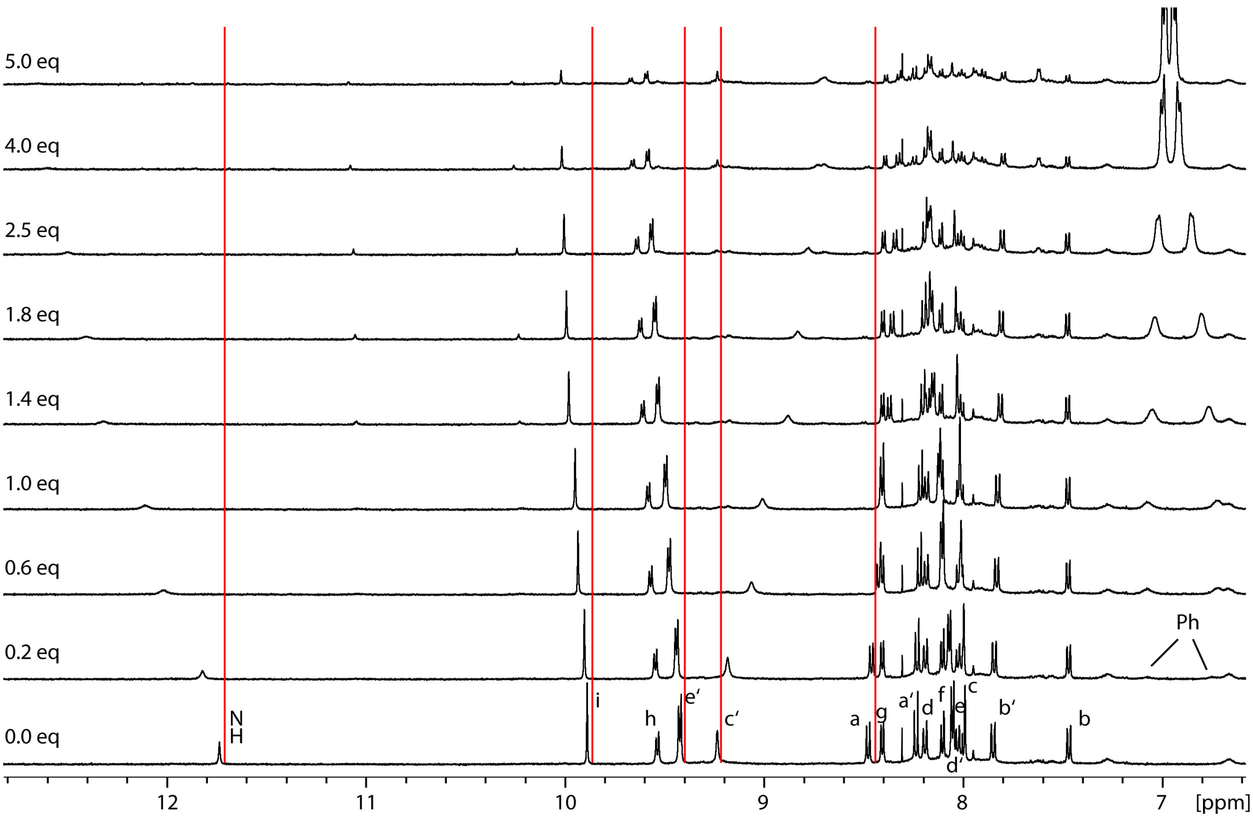

Figure S106: Stacked partial ^{1}H NMR spectra (500 MHz, 298 K, DMSO-d$_6$) of $\mathbf{G^5}$@Pd$_2\mathbf{L^1}_2\mathbf{L^A}_2$.

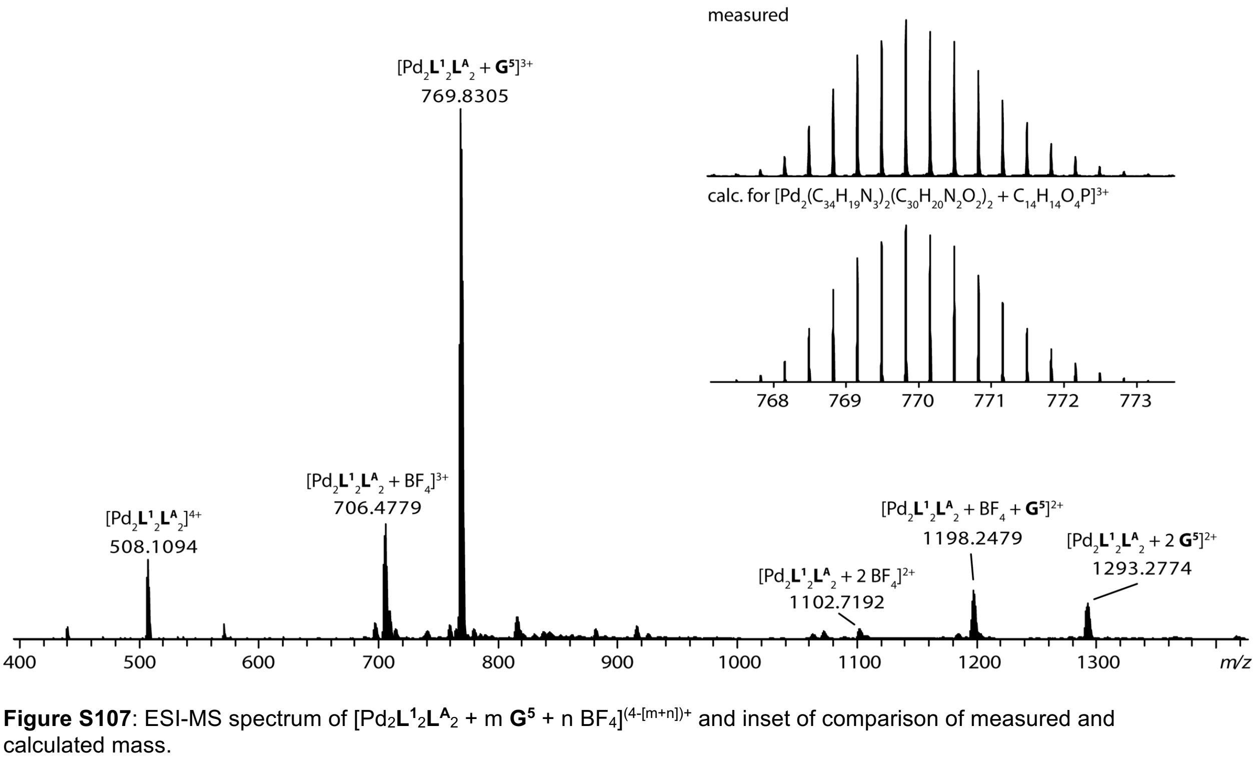

Figure S107: ESI-MS spectrum of [Pd$_2\mathbf{L^1}_2\mathbf{L^A}_2$ + m $\mathbf{G^5}$ + n BF$_4$]$^{(4-[m+n])+}$ and inset of comparison of measured and calculated mass.

3.6 Titration of G^6 to $Pd_2L^1{}_2L^A{}_2$

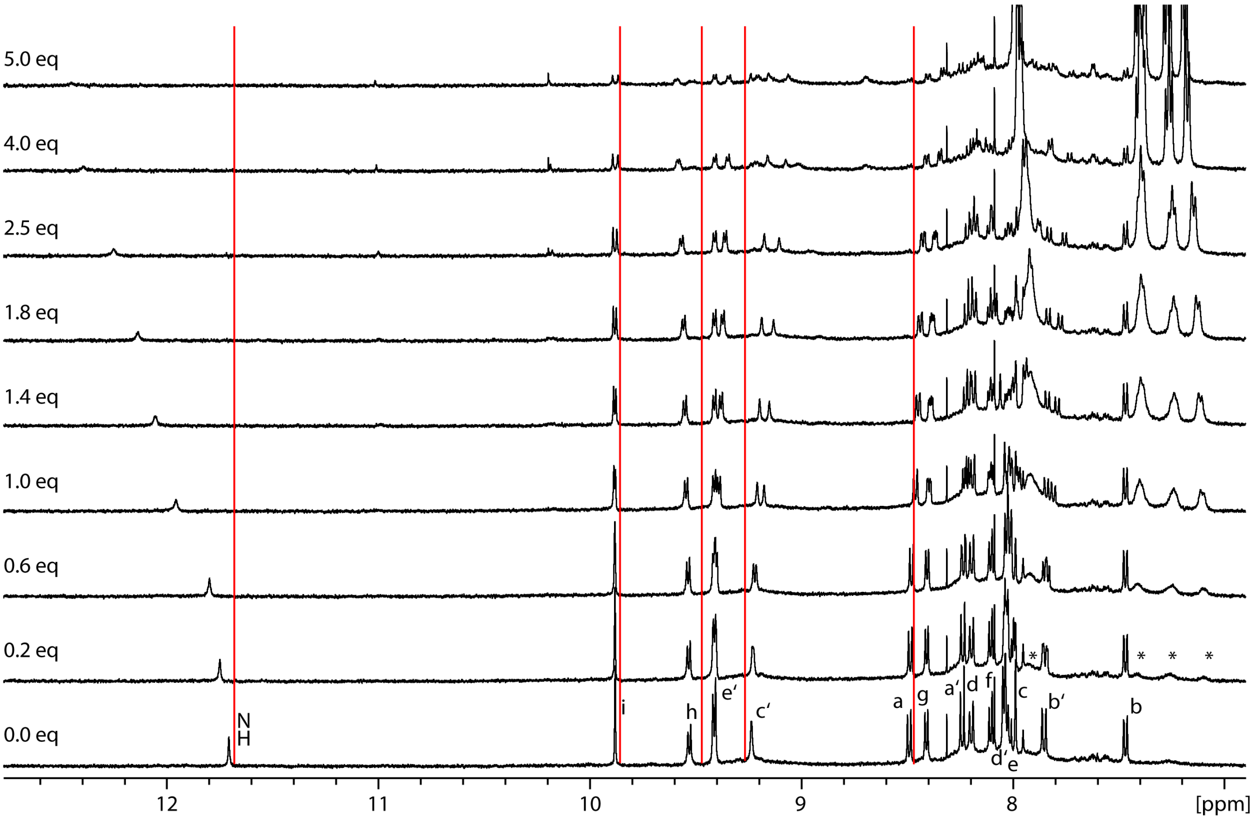

Figure S108: Stacked partial ^{1}H NMR spectra (500 MHz, 298 K, DMSO-d$_6$) of $\mathbf{G^6}$@$Pd_2\mathbf{L^1}_2\mathbf{L^A}_2$. Guest signals are marked with a star *.

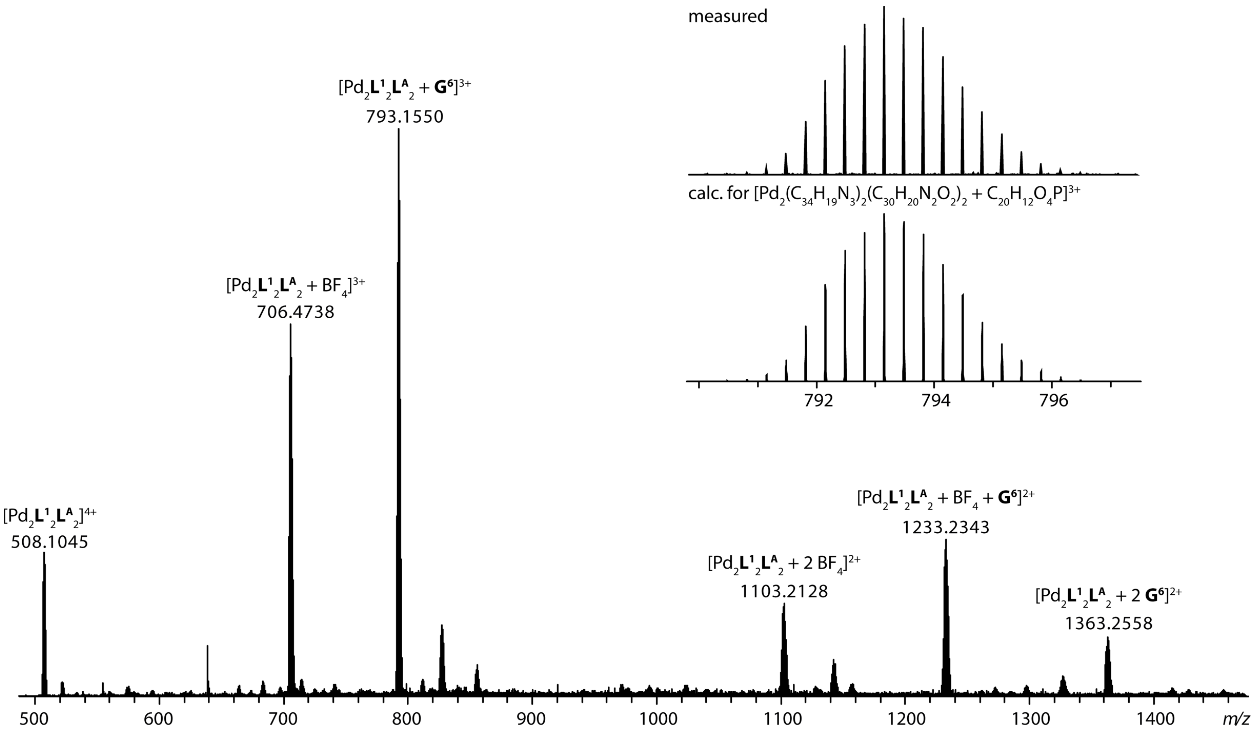

Figure S109: ESI-MS spectrum of $[Pd_2\mathbf{L^1}_2\mathbf{L^A}_2 + m\ \mathbf{G^6} + n\ BF_4]^{(4-[m+n])+}$ and inset of comparison of measured and calculated mass.

3.7 Titration of G^3 to $Pd_2L^1_2L^B_2$

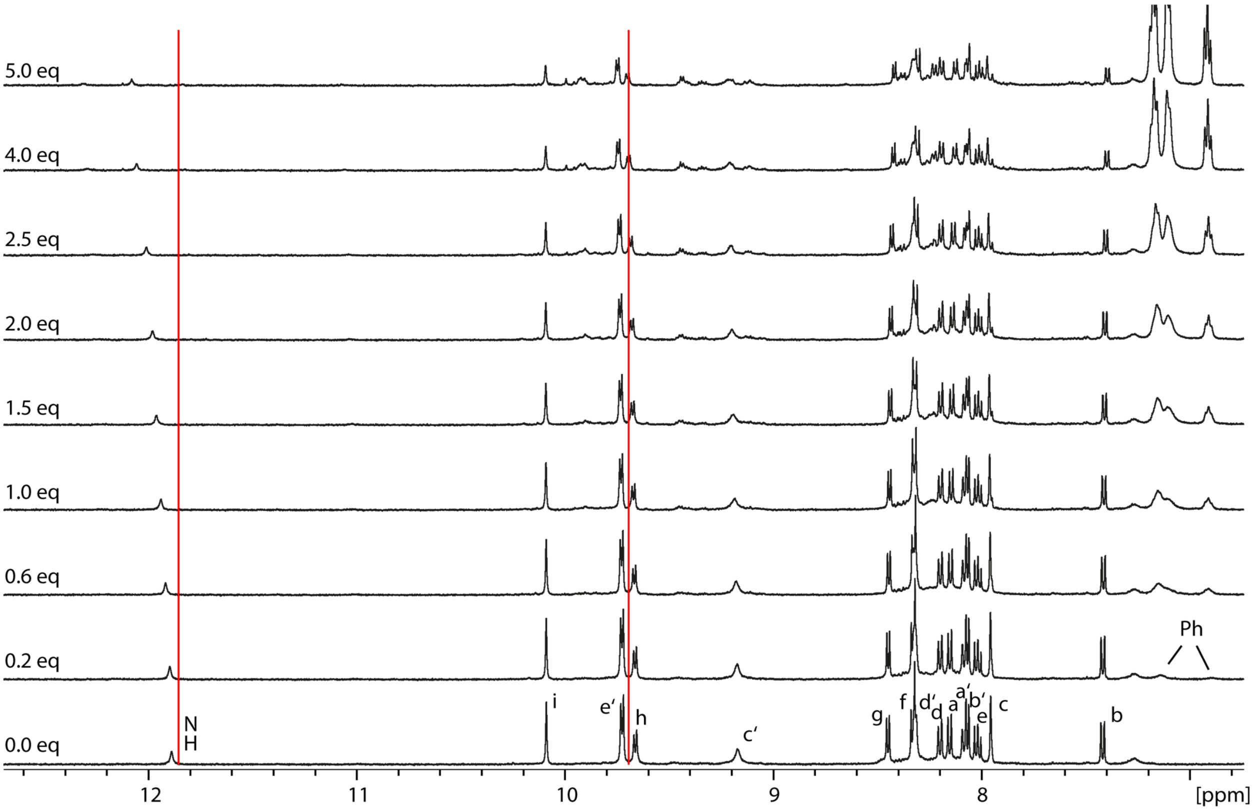

Figure S110: Stacked partial ^1H NMR spectra (500 MHz, 298 K, DMSO-d$_6$) of $\mathbf{G^6}$@$Pd_2\mathbf{L^1}_2\mathbf{L^B}_2$.

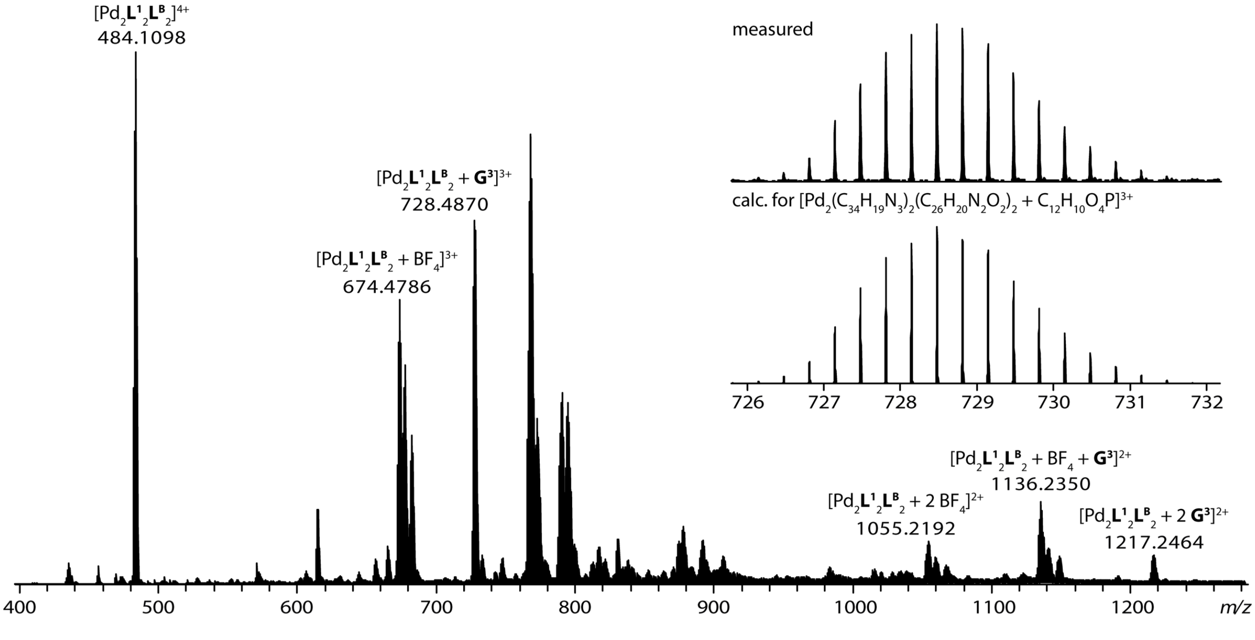

Figure S111: ESI-MS spectrum of $[Pd_2\mathbf{L^1}_2\mathbf{L^B}_2 + m\ \mathbf{G^3} + n\ BF_4]^{(4-[m+n])+}$ and inset of comparison of measured and calculated mass.

3.8 Titration of G^3 to $Pd_2L^1{}_2L^C{}_2$

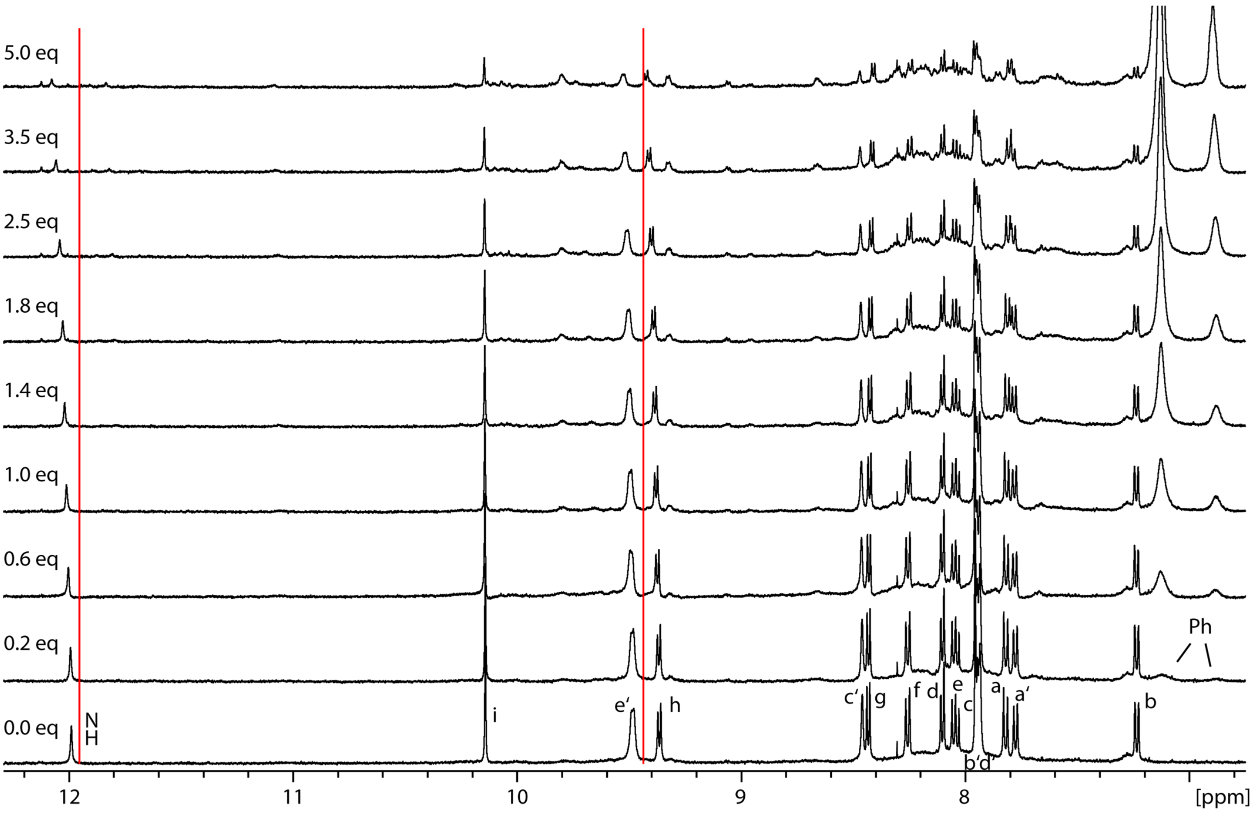

Figure S112: Stacked partial ^1H NMR spectra (500 MHz, 298 K, DMSO-d6) of $\mathbf{G^6}$@$Pd_2\mathbf{L^1}_2\mathbf{L^C}_2$.

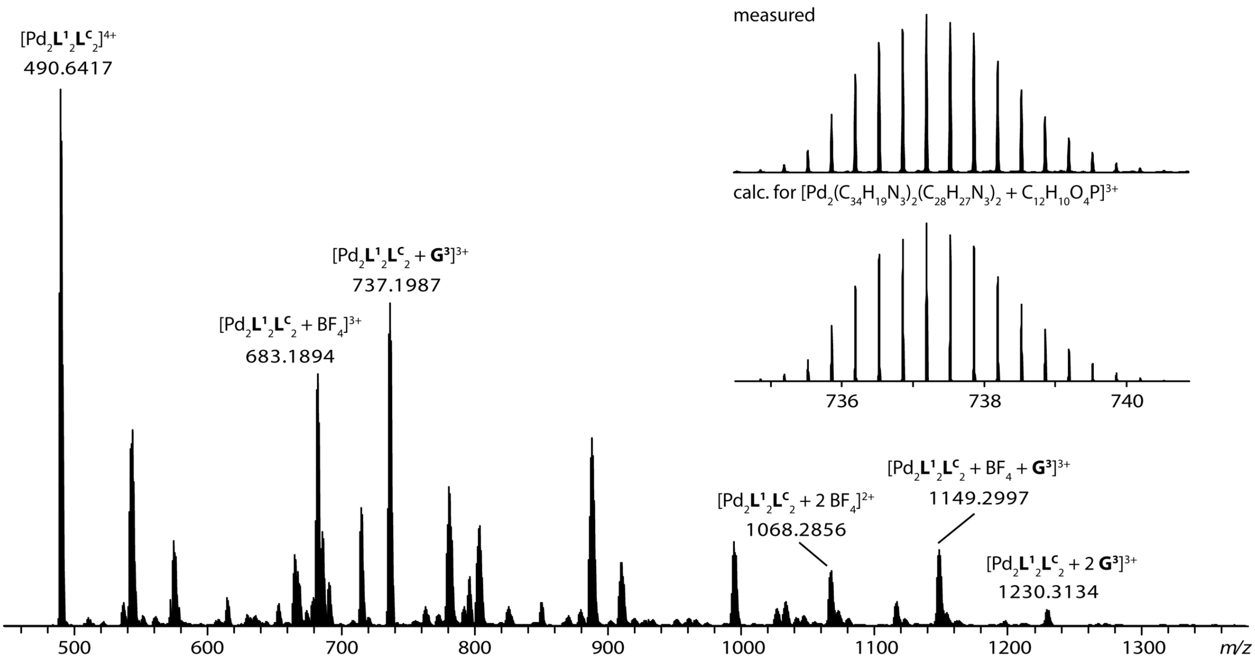

Figure S113: ESI-MS spectrum of $[Pd_2\mathbf{L^1}_2\mathbf{L^C}_2 + m\ \mathbf{G^3} + n\ BF_4]^{(4-[m+n])+}$ and inset of comparison of measured and calculated mass.

3.9 Titration of G^3 to $Pd_2L^1_2L^D_2$

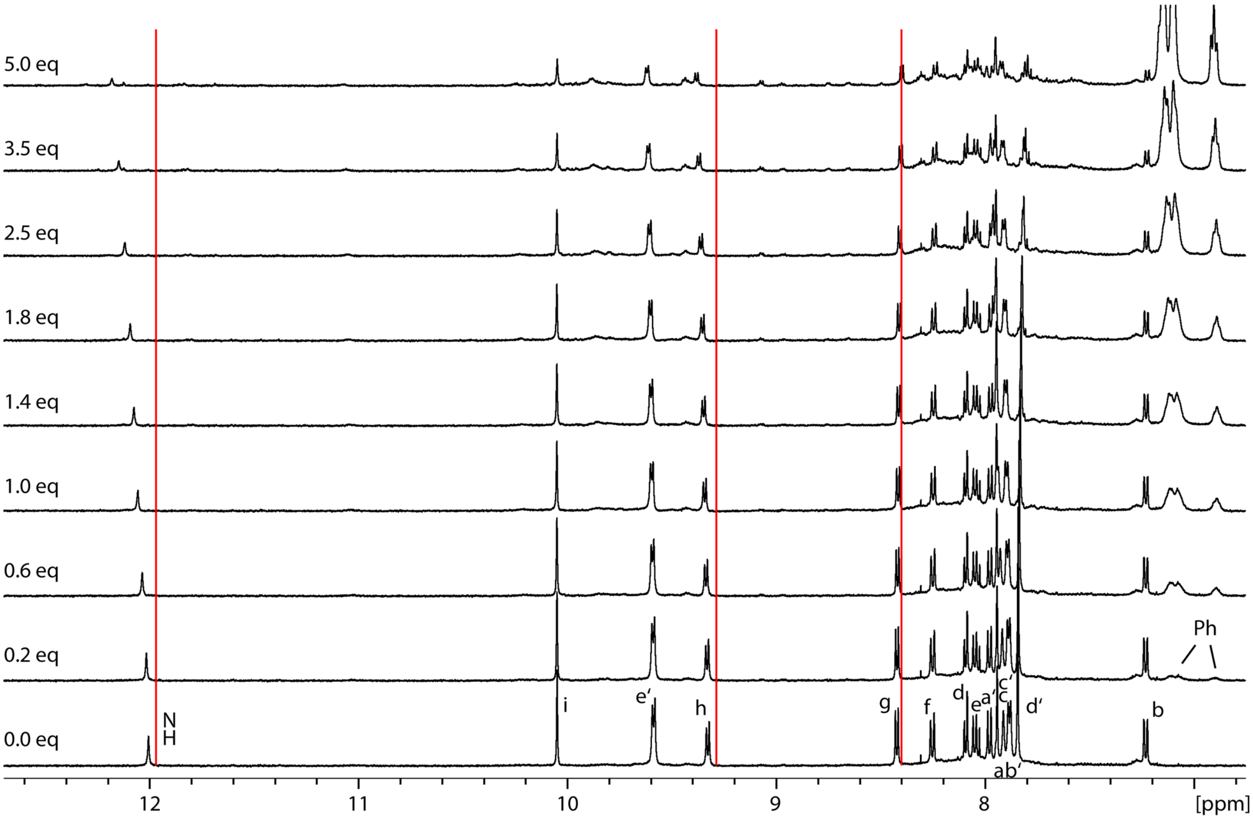

Figure 114: Stacked partial ^{1}H NMR spectra (500 MHz, 298 K, DMSO-d$_6$) of $\mathbf{G^6}$@$Pd_2\mathbf{L^1}_2\mathbf{L^D}_2$.

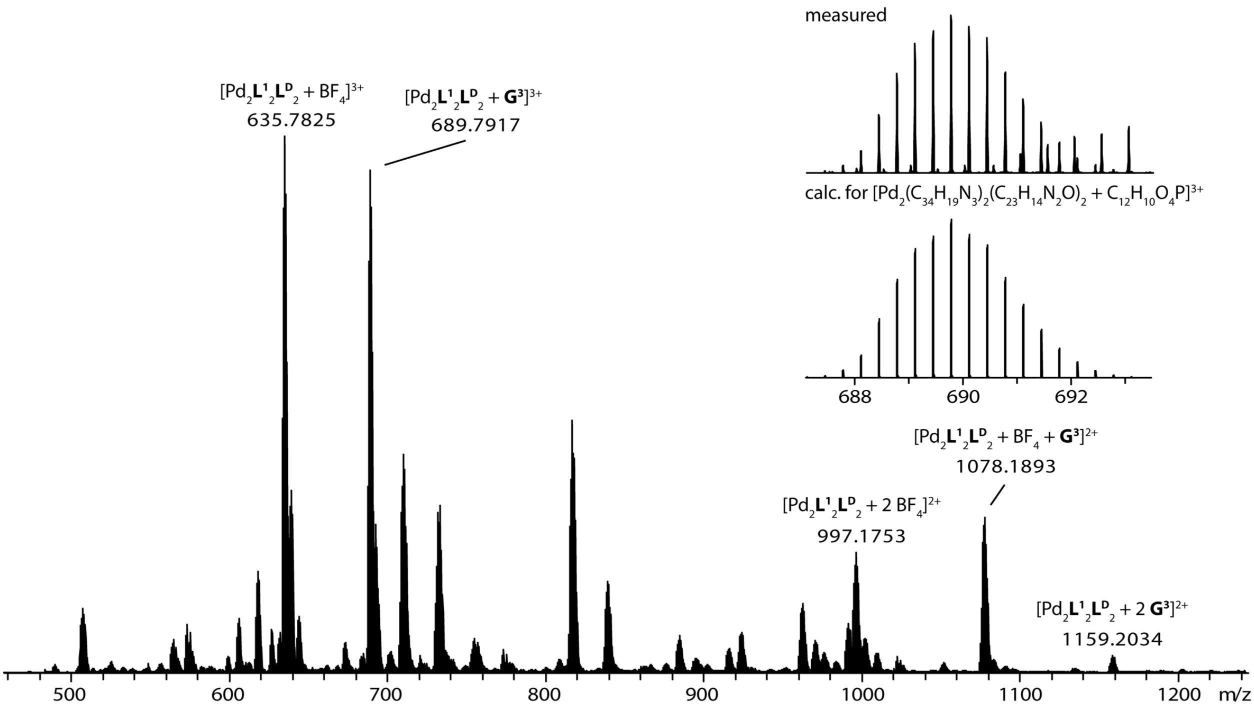

Figure S115: ESI-MS spectrum of $[Pd_2\mathbf{L^1}_2\mathbf{L^D}_2 + m\ \mathbf{G^3} + n\ BF_4]^{(4-[m+n])+}$ and inset of comparison of measured and calculated mass.

3.10 Titration of G^3 to $Pd_2L^2{}_2L^{A}{}_2$

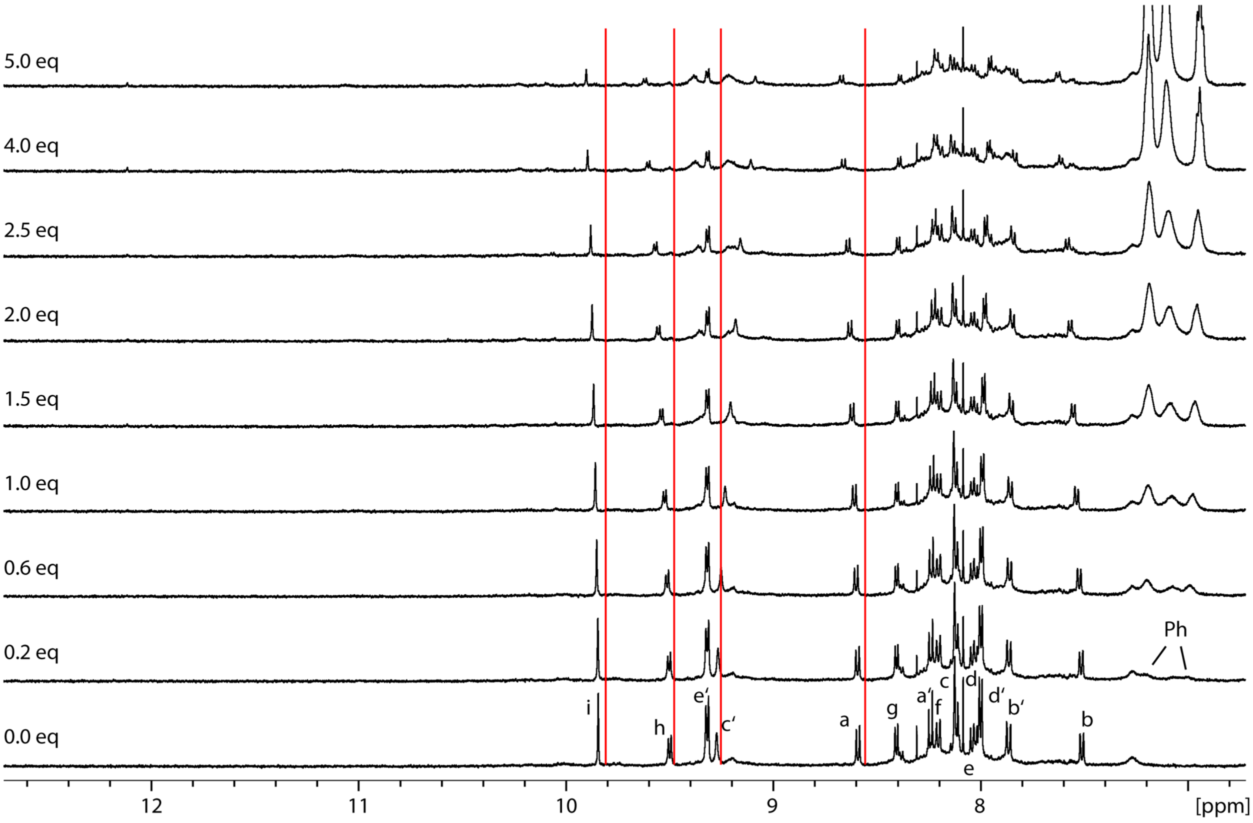

Figure S116: Stacked partial ^1H NMR spectra (500 MHz, 298 K, DMSO-d_6) of $\mathbf{G}^3$@$Pd_2\mathbf{L}^2{}_2\mathbf{L}^{A}{}_2$.

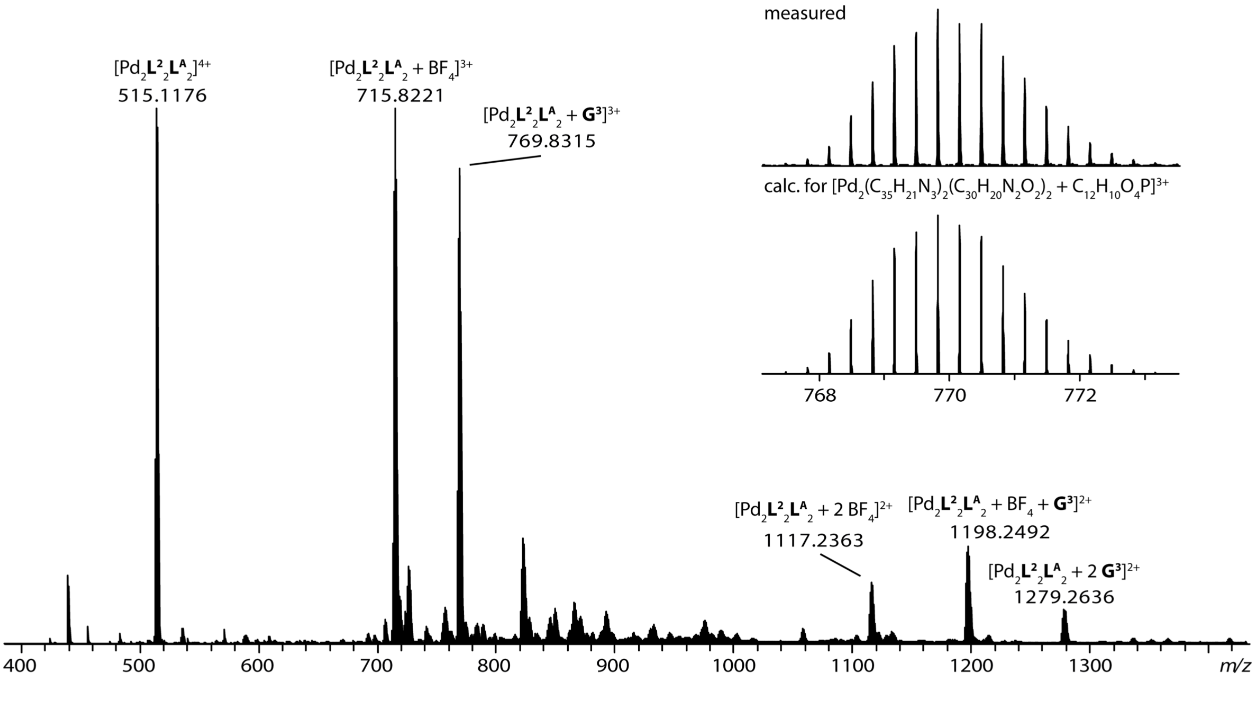

Figure S117: ESI-MS spectrum of $[Pd_2\mathbf{L}^2{}_2\mathbf{L}^{A}{}_2 + m\ \mathbf{G}^3 + n\ BF_4]^{(4-[m+n])+}$ and inset of comparison of measured and calculated mass.

3.11 Titration of G^3 to $Pd_2L^2_2L^B_2$

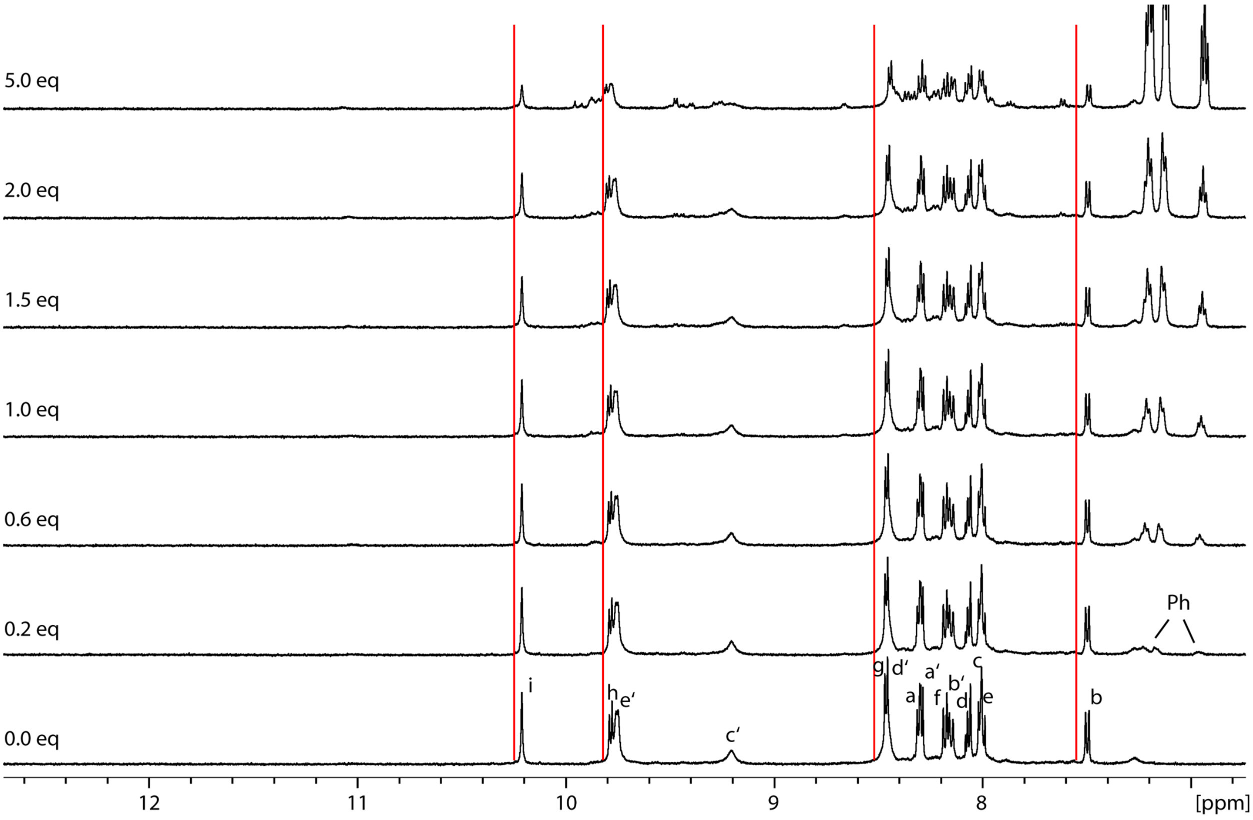

Figure S118: Stacked partial ^{1}H NMR spectra (500 MHz, 298 K, DMSO-d$_6$) of $\mathbf{G^3}$@Pd$_2\mathbf{L^2}_2\mathbf{L^B}_2$.

3.12 Titration of G^3 to $Pd_2L^3{}_2L^A{}_2$

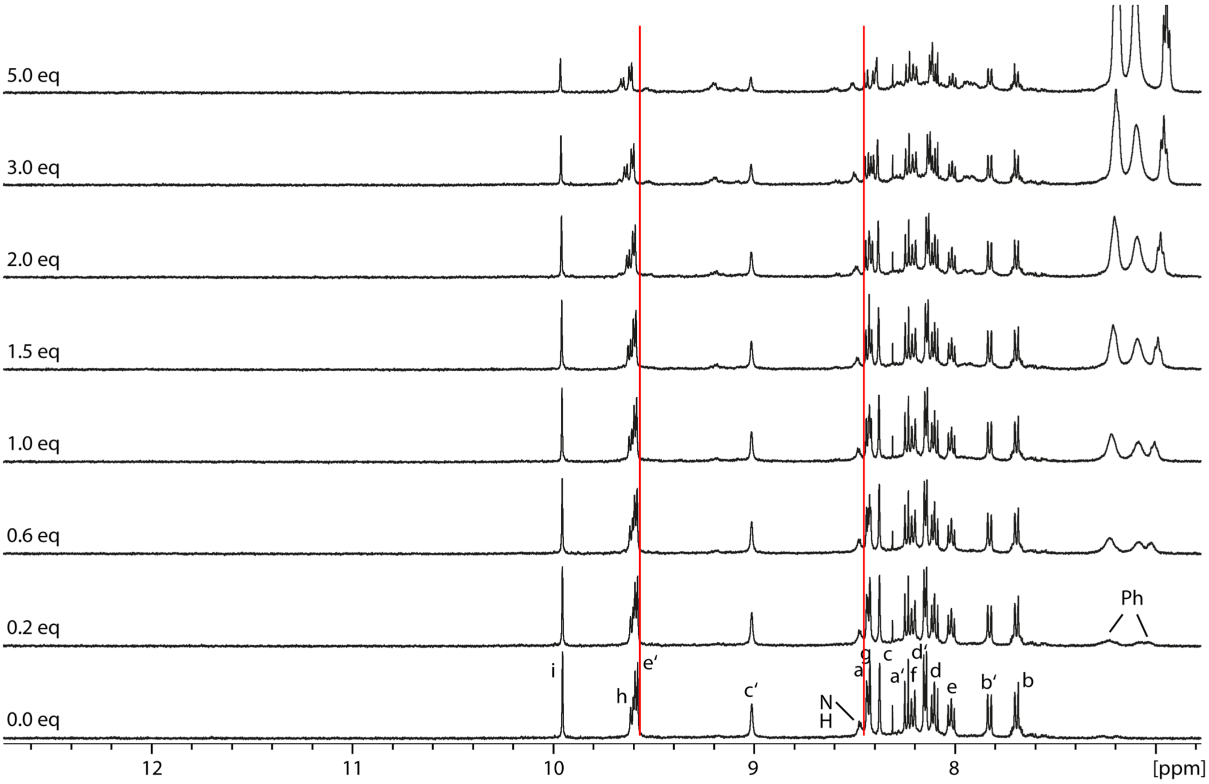

Figure S119: Stacked partial ^1H NMR spectra (500 MHz, 298 K, DMSO-d$_6$) of $\mathbf{G^3}$@$Pd_2\mathbf{L^3}_2\mathbf{L^A}_2$.

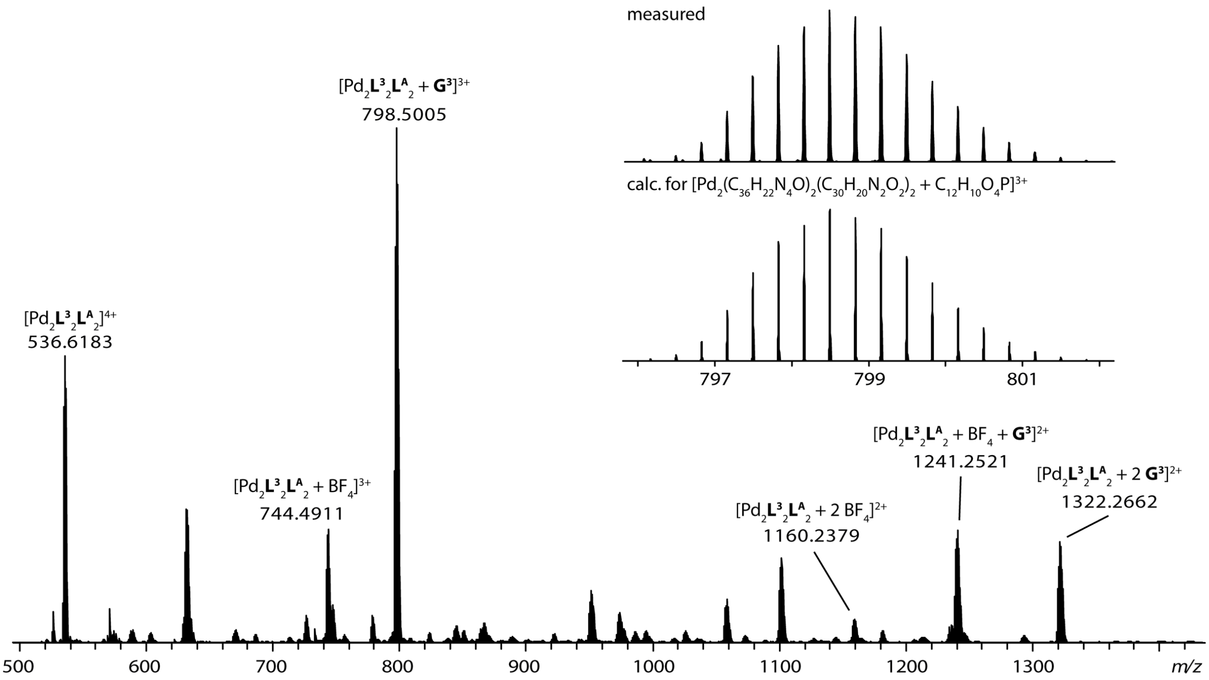

Figure S120: ESI-MS spectrum of $[Pd_2\mathbf{L^3}_2\mathbf{L^A}_2 + m\ \mathbf{G^3} + n\ BF_4]^{(4-[m+n])+}$ and inset of comparison of measured and calculated mass.

3.13 Titration of G^3 to $Pd_2L^4{}_2L^A{}_2$

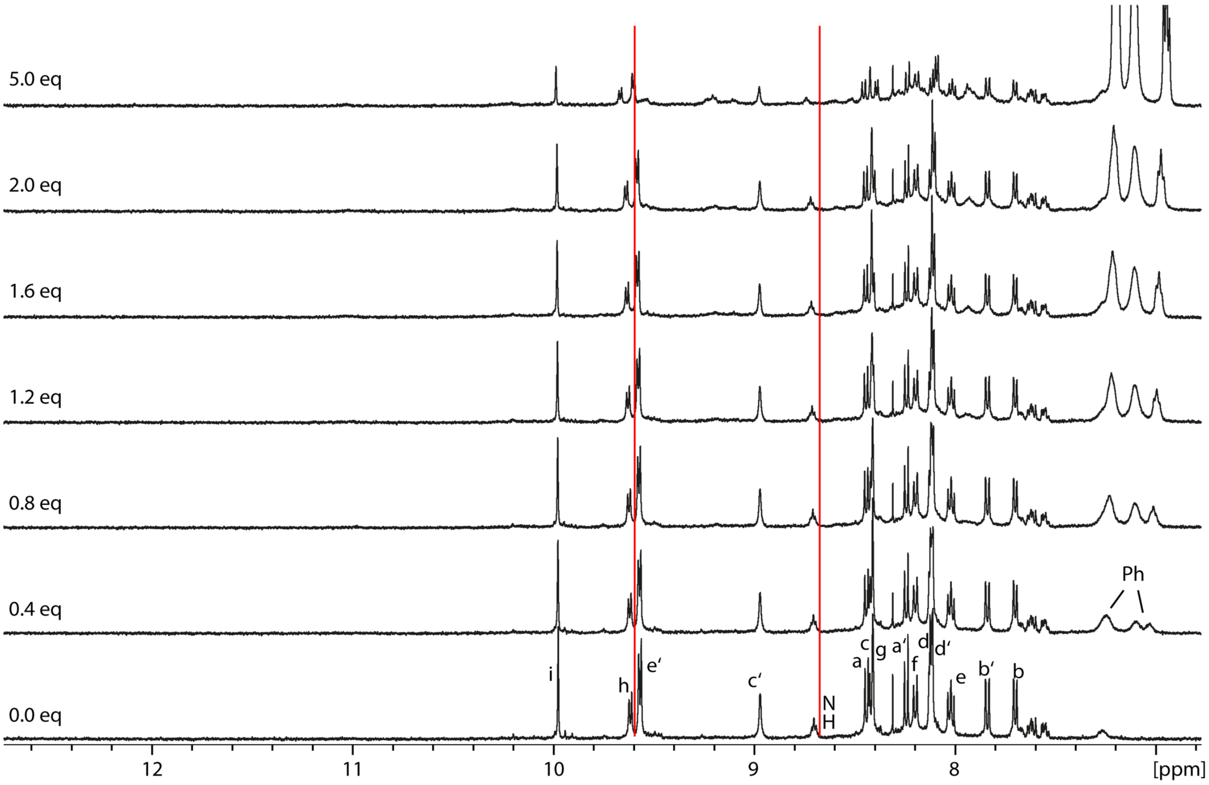

Figure S121: Stacked partial ^1H NMR spectra (500 MHz, 298 K, DMSO-d$_6$) of **G**^{3}@Pd_2**L**$^4{}_2$**L**$^A{}_2$.

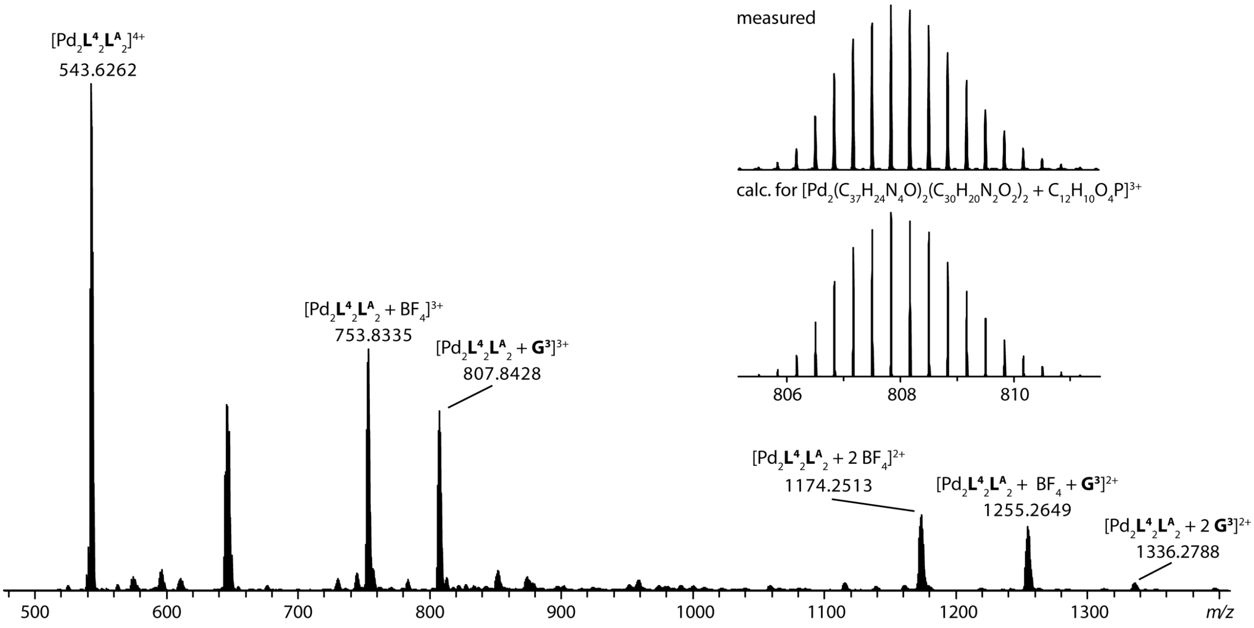

Figure S122: ESI-MS spectrum of $[Pd_2$**L**$^4{}_2$**L**$^A{}_2$ + m **G**^{3} + n $BF_4]^{(4-[m+n])+}$ and inset of comparison of measured and calculated mass.

3.14 Titration of G^3 to $Pd_2L^1_2L^A_2$ in DMF-d₇

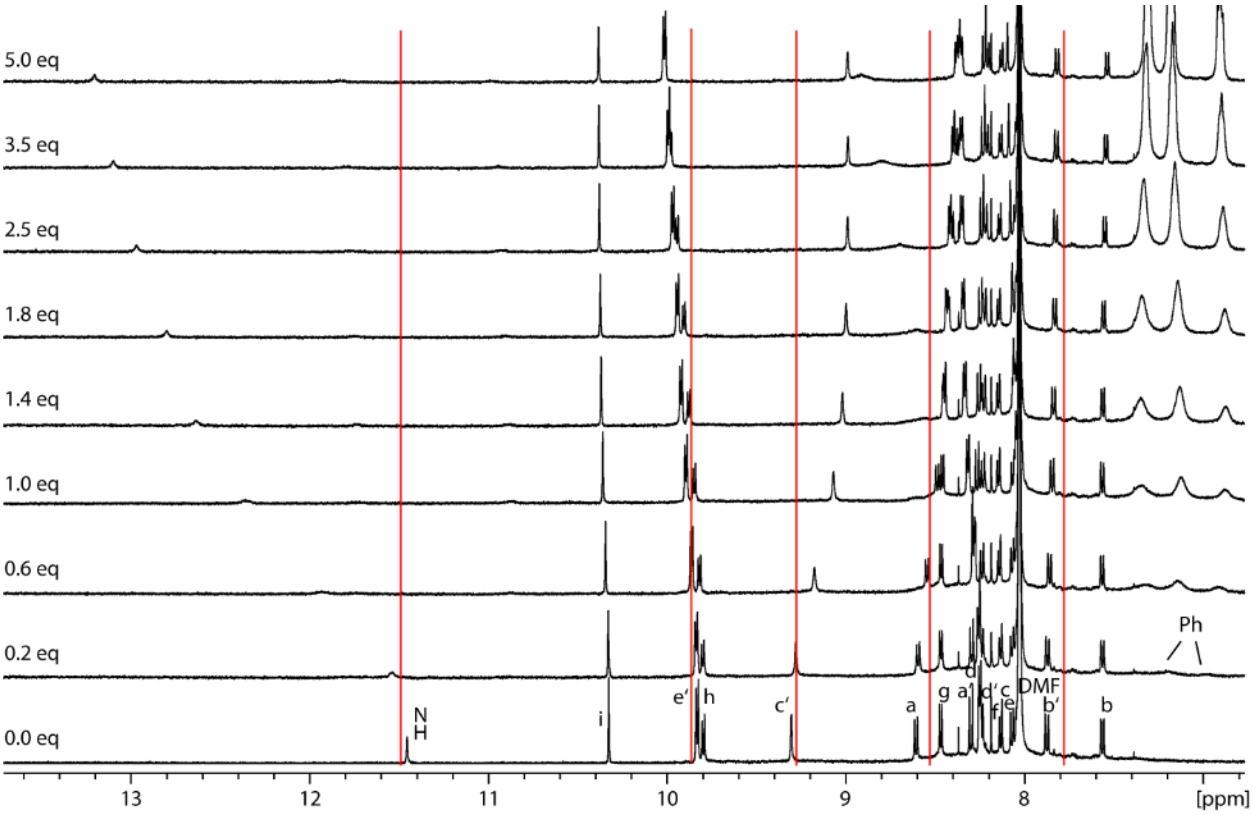

Figure S123: Stacked partial ^1H NMR spectra (500 MHz, 298 K, DMF-d_7) of $\mathbf{G^3}$@$Pd_2\mathbf{L^1}_2\mathbf{L^A}_2$.

3.15 Titration of G^3 to $Pd_2L^2{}_2L^A{}_2$ in DMF-d_7

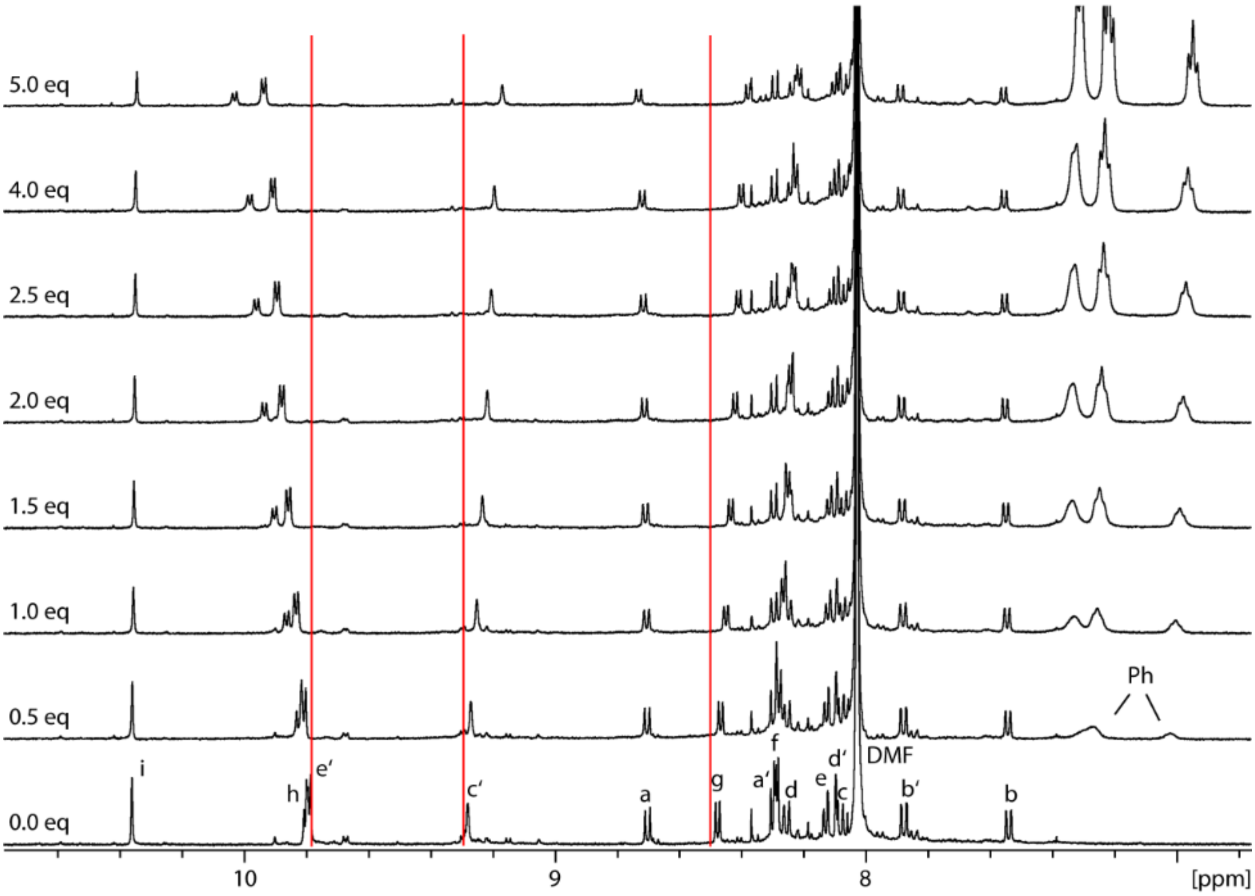

Figure S124: Stacked partial ^{1}H NMR spectra (500 MHz, 298 K, DMF-d_7) of **G**^{3}@Pd_2**L**$^2{}_2$**L**$^A{}_2$.

Table S1: Overview of obtained binding constants K [M^{-1}].

DMSO-d_6	**G**^{3}	**G**^{5}	**G**^{6}
Pd_2**L**$^1{}_2$**L**$^A{}_2$	2062 ± 31	1432 ± 117	513 ± 17
Pd_2**L**$^1{}_2$**L**$^B{}_2$	98 ± 1		
Pd_2**L**$^2{}_2$**L**$^A{}_2$	77 ± 3		
Pd_2**L**$^2{}_2$**L**$^B{}_2$	no binding		
Pd_2**L**$^3{}_2$**L**$^A{}_2$	128 ± 3		
Pd_2**L**$^4{}_2$**L**$^A{}_2$	46 ± 2		
Pd_2**L**$^1{}_2$**L**$^C{}_2$	33 ± 1		
Pd_2**L**$^1{}_2$**L**$^D{}_2$	149 ± 1		
DMF-d_7	**G**^{3}		
Pd_2**L**$^1{}_2$**L**$^A{}_2$	1734 ± 97		
Pd_2**L**$^2{}_2$**L**$^A{}_2$	199 ± 6		

4 Guest competition experiments

Since it was not possible to determine binding constants for the aliphatic guest molecules (see discussion in main text and above), we performed guest competition experiments to test for the relative binding affinity of aliphatic **G**^{4} compared to aromatic **G**^{3}, qualitatively. Therefore, first 1 eq of **G**^{4} was added to a solution of Pd_2**L**$^1{}_2$**L**$^A{}_2$, followed by 1 eq of **G**^{3}. NMR spectra were recorded immediately and 24 and 48 h after addition of **G**^{3}. As a result, compared to the spectrum of pure **G**^{3}@Pd_2**L**$^1{}_2$**L**$^A{}_2$, the aliphatic guest was found to be fully replaced by the aromatic one, indicating that **G**^{3} has a higher affinity to the host than **G**^{4} (compare a similar trend in the MD simulation results for **G**^{3}@Pd_2**L**$^2{}_2$**L**$^A{}_2$ vs. **G**^{4}@Pd_2**L**$^2{}_2$**L**$^A{}_2$).

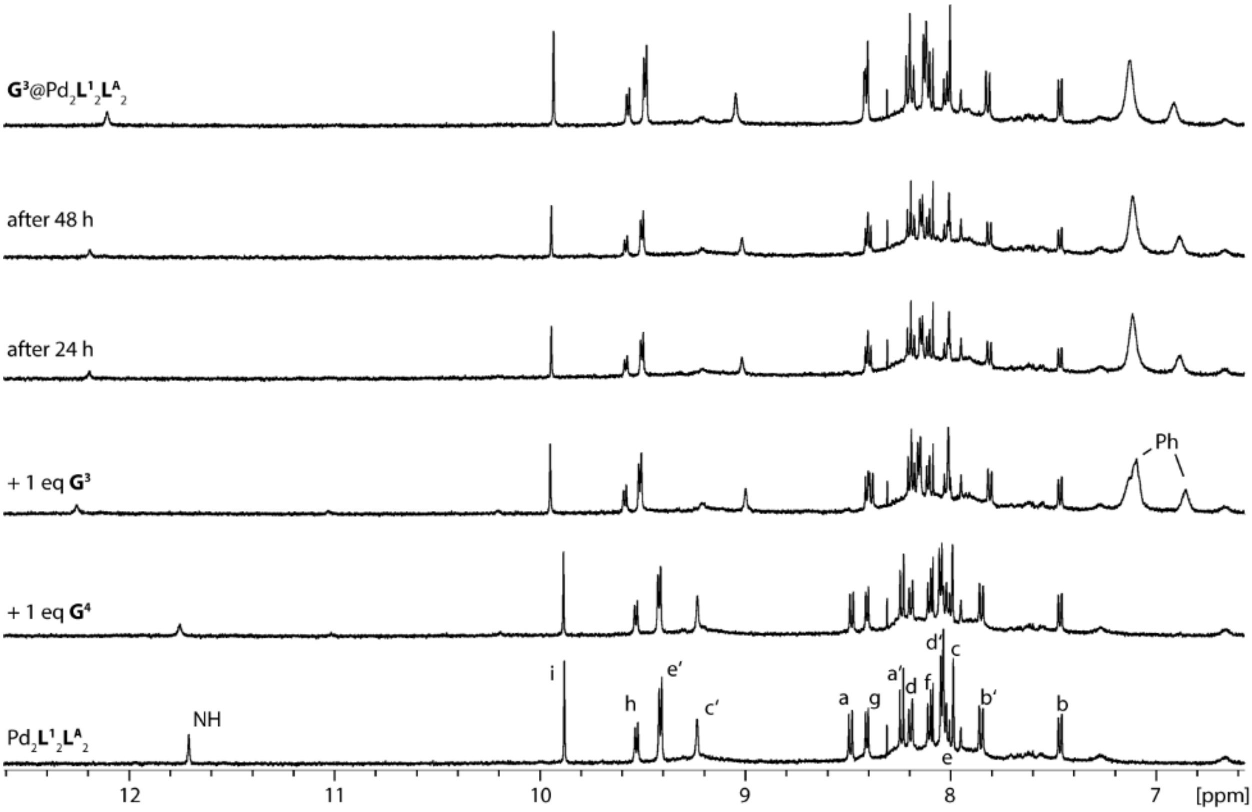

Figure S125: Stacked partial ^{1}H NMR spectra (500 MHz, 298 K, DMSO-d$_6$) of (from bottom to top) Pd_2**L**$^1{}_2$**L**$^A{}_2$, 1 eq **G**^{4} to Pd_2**L**$^1{}_2$**L**$^A{}_2$, 1 eq **G**^{3} to **G**^{4}@Pd_2**L**$^1{}_2$**L**$^A{}_2$, the same after 24 h, after 48 h and pure **G**^{3}@Pd_2**L**$^1{}_2$**L**$^A{}_2$ for comparison.

Next, the order of guest addition was exchanged. First, 1 eq of **G**^{3} was added, yielding the host-guest complex **G**^{3}@Pd_2**L**$^1{}_2$**L**$^A{}_2$, followed by addition of 1 eq of **G**^{4}, leading to only a slight further downfield shift of the inward pointing NH proton, expected for a situation where **G**^{3} is the stronger binding guest (the additional downfield shift is explainable by the doubling of the phosphate guest concentration in this experiment, regardless of substituent). Again, this experiment confirms that **G**^{3} is the stronger binding guest.

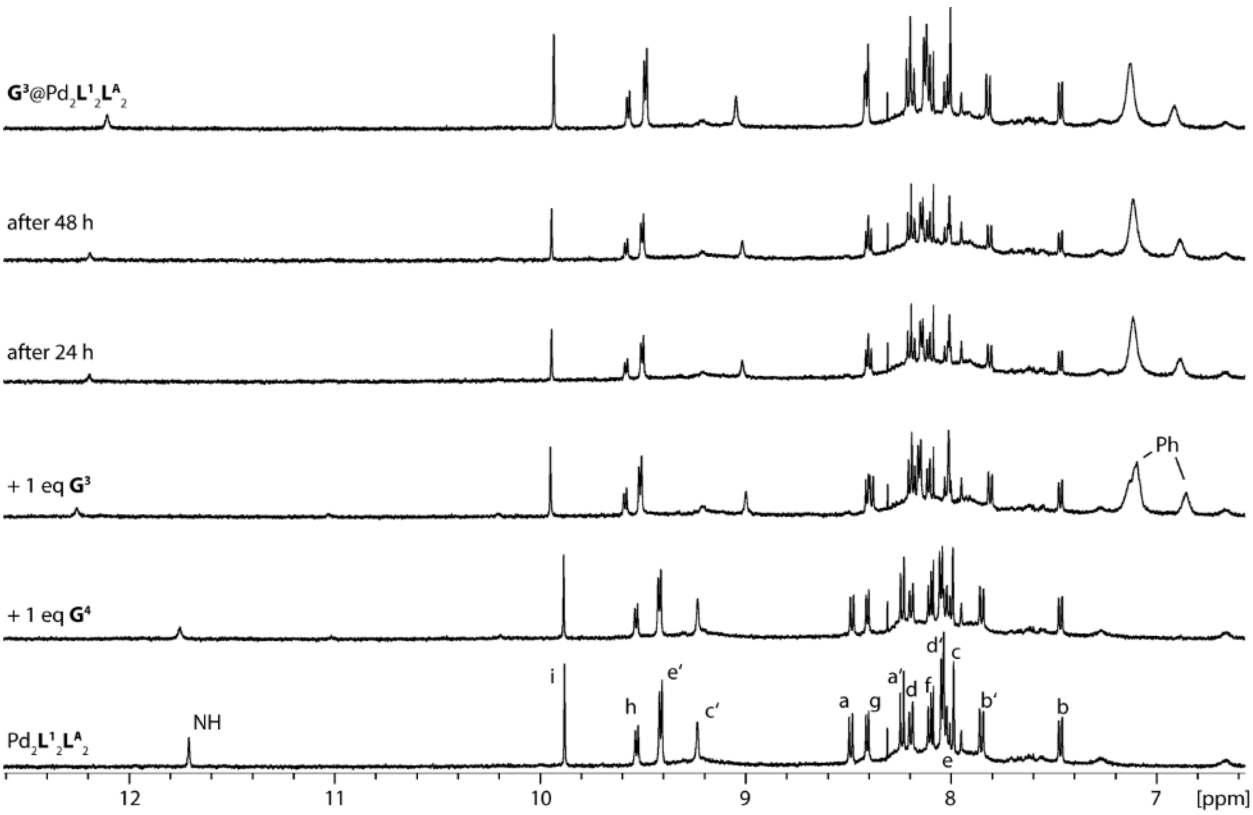

Figure S126: Stacked partial ^1H NMR spectra (500 MHz, 298 K, DMSO-d6) of (from bottom to top) $Pd_2\mathbf{L}^1_2\mathbf{L}^A_2$, 1 eq $\mathbf{G}^3$ to $Pd_2\mathbf{L}^1_2\mathbf{L}^A_2$, 1 eq $\mathbf{G}^4$ to $\mathbf{G}^3$@$Pd_2\mathbf{L}^1_2\mathbf{L}^A_2$, the same after 24 h, after 48 h and pure $\mathbf{G}^3$@$Pd_2\mathbf{L}^1_2\mathbf{L}^A_2$ for comparison (for spectrum of pure $\mathbf{G}^4$@$Pd_2\mathbf{L}^1_2\mathbf{L}^A_2$ see 2nd spectrum from bottom in Fig. S126).

5 Single-crystal X-ray structure analysis

The structures of complexes [$Pd_2\mathbf{L}^1_2\mathbf{L}^A_2$], [$\mathbf{G}^3$@$Pd_2\mathbf{L}^1_2\mathbf{L}^A_2$] and [$\mathbf{G}^5$@$Pd_2\mathbf{L}^1_2\mathbf{L}^A_2$] were studied using single-crystal X-ray crystallography. Due to very thin plate-shaped crystals, the analysis was hampered by the limited scattering power of the samples not allowing to reach the desired (sub-)atomic resolution using our modern microfocussed X-ray in-house CuKα source. Gaining detailed structural insight required cryogenic crystal handling and highly brilliant synchrotron radiation. Hence, diffraction data of all three supramolecular assemblies was collected during two beamtime shifts at macromolecular synchrotron beamline P11, PETRA III, DESY.[8] Counterion and solvent flexibility required carefully adapted macromolecular refinement protocols employing geometrical restraint dictionaries, similarity restraints and restraints for anisotropic displacement parameters (ADPs).

Table S2: Crystal data and structure refinement for cage assemblies [$Pd_2L^1{}_2L^A{}_2$], [G^3@$Pd_2L^1{}_2L^A{}_2$] and [G^5@$Pd_2L^1{}_2L^A{}_2$]

**Compound**	[$Pd_2\mathbf{L^1}_2\mathbf{L^A}_2$]	[$\mathbf{G^3}$@$Pd_2\mathbf{L^1}_2\mathbf{L^A}_2$]	[$\mathbf{G^5}$@$Pd_2\mathbf{L^1}_2\mathbf{L^A}_2$]
**CIF ID**	**ap136d**	**ap214**	**ap217_sq**
**CCDC number**	**2180522**	**2180523**	**2180524**
Empirical formula	$C_{176}H_{140}B_4F_{16}N_{12}O_6Pd_2$	$C_{170}H_{142}B_2F_8N_{12}O_{17}P_2Pd_2$	$C_{324}H_{240}B_4F_{16}N_{24}O_{28}P_4Pd_4$
Formula weight	3079.03	3073.31	5814.11
Temperature [K]	100(2)	100(2)	100(2)
Crystal system	triclinic	monoclinic	monoclinic
Space group (number)	$P\bar{1}$ (2)	$C2/c$ (15)	$C2/c$ (15)
a [Å]	17.287(4)	44.284(9)	44.281(9)
b [Å]	20.596(4)	17.110(3)	16.740(3)
c [Å]	22.355(5)	21.142(4)	21.175(4)
α [Å]	78.53(3)	90	90
β [Å]	76.99(3)	96.51(3)	96.36(3)
γ [Å]	85.78(3)	90	90
Volume [$Å^3$]	7597(3)	15916(6)	15600(5)
Z	2	4	2
ρ_{calc} [g/cm^3]	1.346	1.283	1.238
μ [mm^{-1}]	0.291	0.293	0.399
F(000)	3168	6344	5968
Crystal size [mm^3]	0.060×0.020×0.010	0.300×0.300×0.001	0.100×0.100×0.005
Crystal colour	colourless	colourless	colourless
Crystal shape	plate	plate	plate
Radiation	synchrotron (λ=0.6888 Å)	synchrotron (λ=0.6888 Å)	synchrotron (λ=0.77491 Å)
2Θ range [°]	1.84 to 55.38 (0.74 Å)	1.79 to 55.66 (0.74 Å)	2.02 to 55.88 (0.83 Å)
Index ranges	$-20 \le h \le 20$<br>$-26 \le k \le 26$<br>$-29 \le l \le 29$	$-53 \le h \le 53$<br>$-20 \le k \le 19$<br>$-26 \le l \le 26$	$-45 \le h \le 45$<br>$-19 \le k \le 20$<br>$-25 \le l \le 24$
Reflections collected	93015	95335	66738
Independent reflections	25824<br>R_{int} = 0.0260<br>R_{sigma} = 0.0236	16159<br>R_{int} = 0.0285<br>R_{sigma} = 0.0189	10988<br>R_{int} = 0.0211<br>R_{sigma} = 0.0138
Completeness	84.5 %	96.2 %	77.8 %
Data/Restraints/Parameters	25824/4462/2336	16159/270/1023	10988/1384/912
Goodness-of-fit on F^2	1.008	1.051	1.140
Final R indexes [$I \ge 2\sigma(I)$]	R_1=0.0794, wR_2=0.2675	R_1=0.06, wR_2=0.2012	R_1=0.0720, wR_2=0.2351
Final R indexes [all data]	R_1=0.0844, wR_2=0.2733	R_1=0.071, wR_2=0.2096	R_1=0.078, wR_2=0.2475
Largest peak/hole [$eÅ^{-3}$]	4.13/-0.98	1.56/-1.09	0.76/-1.06

5.1 Data collection and refinement details of [Pd_2**L**$^1{}_2$**L**$^A{}_2$], ap136d

Single crystals were grown by slow diffusion of toluene into a solution of [Pd_2**L**$^1{}_2$**L**$^A{}_2$] in dimethylformamide (DMF). A single crystal of [Pd_2**L**$^1{}_2$**L**$^A{}_2$] in mother liquor was pipetted onto a glass slide containing NVH oil. To avoid cracking of the crystal, the crystal was quickly mounted onto a 0.06 mm nylon loop and immediately flash cooled in liquid nitrogen. Crystals were stored at cryogenic temperature in dry shippers, in which they were safely transported to macromolecular beamline P11 at Petra III,[8] DESY, Germany. A wavelength of λ = 0.6888 Å was chosen using a liquid N_2 cooled double crystal monochromator. Single crystal X-ray diffraction data was collected at 100(2) K on a single axis goniometer, equipped with an Oxford Cryostream 800 open flow cooling device and an Eiger 2 12M detector.

3600 diffraction images were collected in a 360° φ sweep at a detector distance of 154 mm, 100% filter transmission, 0.1° step width and 50 ms exposure time per image. Data integration and reduction were undertaken using XDS.[9] The structure was solved by intrinsic phasing/direct methods using SHELXT[10] and refined with SHELXL[11] using 22 cpu cores for full-matrix least-squares routines on F^2 and ShelXle[12] as a graphical user interface and the DSR program plugin was employed for modeling.[13,14]

The asymmetric unit contains a full cage. Two of the four co-crystallised tetrafluoroborate was modelled with two discrete positions refining their occupancy factor to 78:22 using a free variable. Four of the five co-crystallised toluene molecules as well as one of the two co-crystallised DMF molecules were modelled disordered refining their occupancy factor with a free variable for to 66%:34% for Toluene in residue 21-22 and 78%:22% for DMF in residue 11-12. An additional free variable was refined to occupancy of 67%:53% for toluene in residues 19-20, 24-25, 28-29 as well as tetrafluoroborate in residues 9-10, all of which are in closed proximity to each other.

Despite reaching 0.74 Å resolution, disorder and poor crystal quality required stereochemical restraints to be employed for ensuring a sensible geometry of the organic part of the structure.

Stereochemical restraints for the ligands **L**^{1} (residue class ICZ) and **L**^{A} (residue class MPR) and co-crystallised toluene (residue class TOL) and dimethylformamide (residue class DMF) solvent molecules were generated by the GRADE program using the GRADE Web Server (http://grade.globalphasing.org) and applied in the refinement. A GRADE dictionary for SHELXL contains target values and standard deviations for 1,2-distances (DFIX) and 1,3-distances (DANG), as well as restraints for planar groups (FLAT). All displacements for non-hydrogen atoms were refined anisotropically. The refinement of ADP's for carbon, nitrogen, oxygen, boron and fluorine atoms was enabled by a combination of similarity restraints (SIMU) with lowered standard deviation of [0.02 0.04] and rigid bond restraints (RIGU).[15] For disordered toluene molecules in residues 24-25 additional ISOR restraints were applied.

5.2 Data collection and refinement details of [G^3@$Pd_2L^1{}_2L^A{}_2$], ap214

Single crystals were grown by slow diffusion of diethylether into a solution of [**G**^{3}@Pd$_2$**L**$^1{}_2$**L**$^A{}_2$] in dimethylformamide (DMF). A single crystal of [**G**^{3}@Pd$_2$**L**$^1{}_2$**L**$^A{}_2$] in mother liquor was pipetted onto a glass slide containing NVH oil. To avoid cracking of the crystal, the crystal was quickly mounted onto a 0.3 mm nylon loop and immediately flash cooled in liquid nitrogen. Crystals were stored at cryogenic temperature in dry shippers, in which they were safely transported to macromolecular beamline P11 at Petra III[8], DESY, Germany. A wavelength of λ = 0.6888 Å was chosen using a liquid N_2 cooled double crystal monochromator. Single-crystal X-ray diffraction data was collected at 100(2) K on a single axis goniometer, equipped with an Oxford Cryostream 800 open flow cooling device and an Eiger 2 12M detector.

3600 diffraction images were collected in a 360° φ sweep at a detector distance of 154 mm, 100% filter transmission, 0.1° step width and 10 ms exposure time per image. Data integration and reduction were undertaken using XDS.[9] The structure was solved by intrinsic phasing/direct methods using SHELXT[10] and refined with SHELXL[11] using 22 cpu cores for full-matrix least-squares routines on F^2 and ShelXle[12] as a graphical user interface and the DSR program plugin was employed for modeling.[13,14]

The asymmetric unit contains half a cage, one diphenylphosphate (residue class POB), one tetrafluoroborate counter ion (residue class BF4), three diethylether as well as one dimethylformamide solvent molecules. One of the three co-crystallised diethylether solvent molecules is close to a special position (2-fold axis) and was therefore modelled with negative PART and a fixed occupancy factor of 50%.

Despite reaching 0.74 Å resolution, disorder and poor crystal quality required stereochemical restraints to be employed for ensuring a sensible geometry of the solvent part of the structure.

Stereochemical restraints for the co-crystallised diethyether (residue class ETO) and dimethylformamide (residue class DMF) solvent molecules were generated by the GRADE program using the GRADE Web Server (http://grade.globalphasing.org) and applied in the refinement. A GRADE dictionary for SHELXL contains target values and standard deviations for 1,2-distances (DFIX) and 1,3-distances (DANG), as well as restraints for planar groups (FLAT). All displacements for non-hydrogen atoms were refined anisotropically. The refinement of ADP's for carbon, nitrogen, oxygen, boron and fluorine atoms of dimethylformamide and diethylether in residues classes DMF and ETO was enabled by a combination of similarity restraints (SIMU) and rigid bond restraints (RIGU).[15] Rigid bond restraints (RIGU) were also employed for the phosphate guest (**G**^{3}) in residue class POB.

5.3 Data collection and refinement details of [G^5@$Pd_2L^1{}_2L^A{}_2$], ap217_sq

Single crystals were grown by slow diffusion of diethylether into a solution of [$\mathbf{G}^5$@$Pd_2\mathbf{L}^1{}_2\mathbf{L}^A{}_2$] in dimethylformamide (DMF). A single crystal of [$\mathbf{G}^5$@$Pd_2\mathbf{L}^1{}_2\mathbf{L}^A{}_2$] in mother liquor was pipetted onto a glass slide containing NVH oil. To avoid cracking of the crystal, the crystal was quickly mounted onto a 0.1 mm nylon loop and immediately flash cooled in liquid nitrogen. Crystals were stored at cryogenic temperature in dry shippers, in which they were safely transported to macromolecular beamline P11 at Petra III,[8] DESY, Germany. A wavelength of λ = 0.77491 Å was chosen using a liquid N_2 cooled double crystal monochromator. Single crystal X-ray diffraction data was collected at 100(2) K on a single axis goniometer, equipped with an Oxford Cryostream 800 open flow cooling device and an Eiger 2 12M detector.

3600 diffraction images were collected in a 360° φ sweep at a detector distance of 154 mm, 100% filter transmission, 0.1° step width and 10 ms exposure time per image. Data integration and reduction were undertaken using XDS.[9] The structure was solved by intrinsic phasing/direct methods using SHELXT[10] and refined with SHELXL[11] using 22 cpu cores for full-matrix least-squares routines on F^2 and ShelXle[12] as a graphical user interface and the DSR program plugin was employed for modeling.[13,14]

The asymmetric unit contains half a cage, one ditoloylphosphate (residue class POT), one tetrafluoroborate counter ion (residue class BF4) and a co-crystallised dimethylformamide solvent molecule. Despite reaching 0.83 Å resolution, poor crystal quality required stereochemical restraints to be employed for ensuring a sensible geometry of the solvent part of the structure.

Stereochemical restraints for the co-crystallised dimethylformamide (residue class DMF) solvent molecule were generated by the GRADE program using the GRADE Web Server (http://grade.globalphasing.org) and applied in the refinement. A GRADE dictionary for SHELXL contains target values and standard deviations for 1,2-distances (DFIX) and 1,3-distances (DANG), as well as restraints for planar groups (FLAT). All displacements for non-hydrogen atoms were refined anisotropically. The refinement of ADP's for carbon, nitrogen, oxygen, boron and fluorine atoms was enabled by a combination of similarity restraints (SIMU) and rigid bond restraints (RIGU).[15]. The contribution of the electron density from disordered solvent molecules, which could not be modeled with discrete atomic positions were handled using the SQUEEZE[16] routine in PLATON.[17] The solvent mask file (.fab) computed by PLATON were included in the SHELXL refinement via the ABIN instruction leaving the measured intensities untouched.

6 Modelling of coordination cage structures

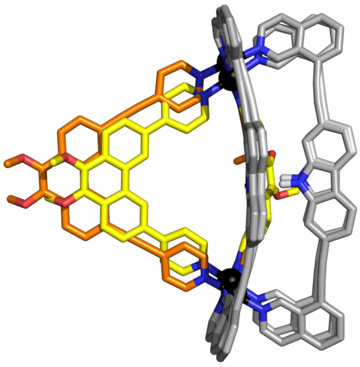

Figure S127: Overlay of PM6-optimized structures of $Pd_2\mathbf{L^1}_2\mathbf{L^A}_2$ with $Pd_2\mathbf{L^1}_2\mathbf{L^B}_2$.

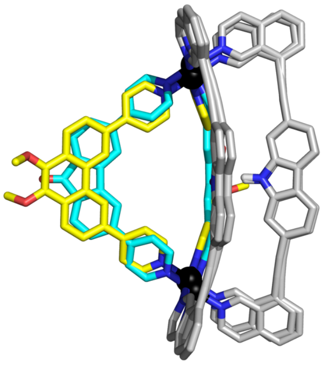

Figure 128: Overlay of PM6-optimized structures of $Pd_2\mathbf{L^1}_2\mathbf{L^B}_2$ with $Pd_2\mathbf{L^1}_2\mathbf{L^D}_2$.

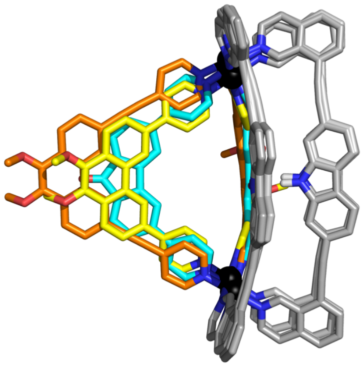

Figure S129: Overlay of PM6-optimized structures of $Pd_2\mathbf{L}^1{}_2\mathbf{L}^A{}_2$ with $Pd_2\mathbf{L}^1{}_2\mathbf{L}^B{}_2$ and $Pd_2\mathbf{L}^1{}_2\mathbf{L}^D{}_2$.

7 Ion Mobility Measurements

Ion mobility measurements were performed on a Bruker timsTOF instrument combining a trapped ion mobility (TIMS) with a time-of-flight (TOF) mass spectrometer in one instrument.

In contrast to the conventional drift tube method to determine mobility data, where ions are carried by an electric field through a stationary drift gas, the TIMS method is based on an electric field ramp to hold ions in place against a carrier gas pushing them in the direction of the analyzer. Consequently, larger sized ions that experience more carrier gas impacts leave the TIMS units first and smaller ions elute later. This method offers a much higher mobility resolution despite a smaller device size.

Measurement: After the generation of ions by electrospray ionisation (ESI, analyte concentration: 0.07 mM, solvent: acetonitrile, capillary voltage: 3600V, end plate offset voltage: 500V, nebulizer gas pressure: 0.3 bar, dry gas flow rate: 3.0 L/min, dry temperature: 75 °C) the desired ions were orthogonally deflected into the TIMS cell consisting of an entrance funnel, the TIMS analyser (carrier gas: N_2, temperature: 305 K, entrance pressure: 2.55 mbar, exit pressure: 0.89 mbar, IMS imeX ramp end: 1.92 $1/K_0$, IMS imeX ramp start: 0.54 $1/K_0$) and an exit funnel. As a result, the ions are stationary trapped. After accumulation (accumulation time: 10 ms), a stepwise reduction of the electric field strength leads to a release of ion packages separated by their mobility. After a subsequent focussing, the separated ions are transferred to the TOF-analyser.

The ion mobility K was directly calculated from the trapping electric field strength E and the velocity of the carrier gas stream v_g *via*

$$K = \frac{v_g}{E} = \frac{A}{U_{release} - U_{out}} \qquad (1)$$

where A is a calibration constant (based on calibration standards), $U_{release}$ is the voltage at which the ions are released from the analyser and U_{out} is the voltage applied to the exit of the tube. The ion mobility is corrected to standard gas density *via*

$$K_0 = K \frac{P}{1013\ \mathrm{hPa}} \frac{237\ \mathrm{K}}{T} \qquad (2)$$

to obtain the reduced mobility K_0, where P is the pressure and T is the temperature. By using the MASON-SCHAMP equation, the collisional cross-section Ω can be calculated:

$$\Omega = \frac{(18\pi)^{\frac{1}{2}}}{16} \frac{ze}{(k_B T)^{\frac{1}{2}}} \left[\frac{1}{\mu}\right]^{\frac{1}{2}} \frac{1}{K_0} \frac{1}{N_0} \qquad (3)$$

where ze is the ion charge, k_B is the BOLTZMANN constant, μ is the reduced mass of analyte and carrier gas and N_0 is the number density of the neutral gas.[18–20]

For calibration of both the TIMS and TOF analysers, commercially available Agilent ESI tuning mix was used. The instrument was calibrated before each measurement, including each change in the ion mobility resolution mode ("imeX" settings: survey, detect or ultra).

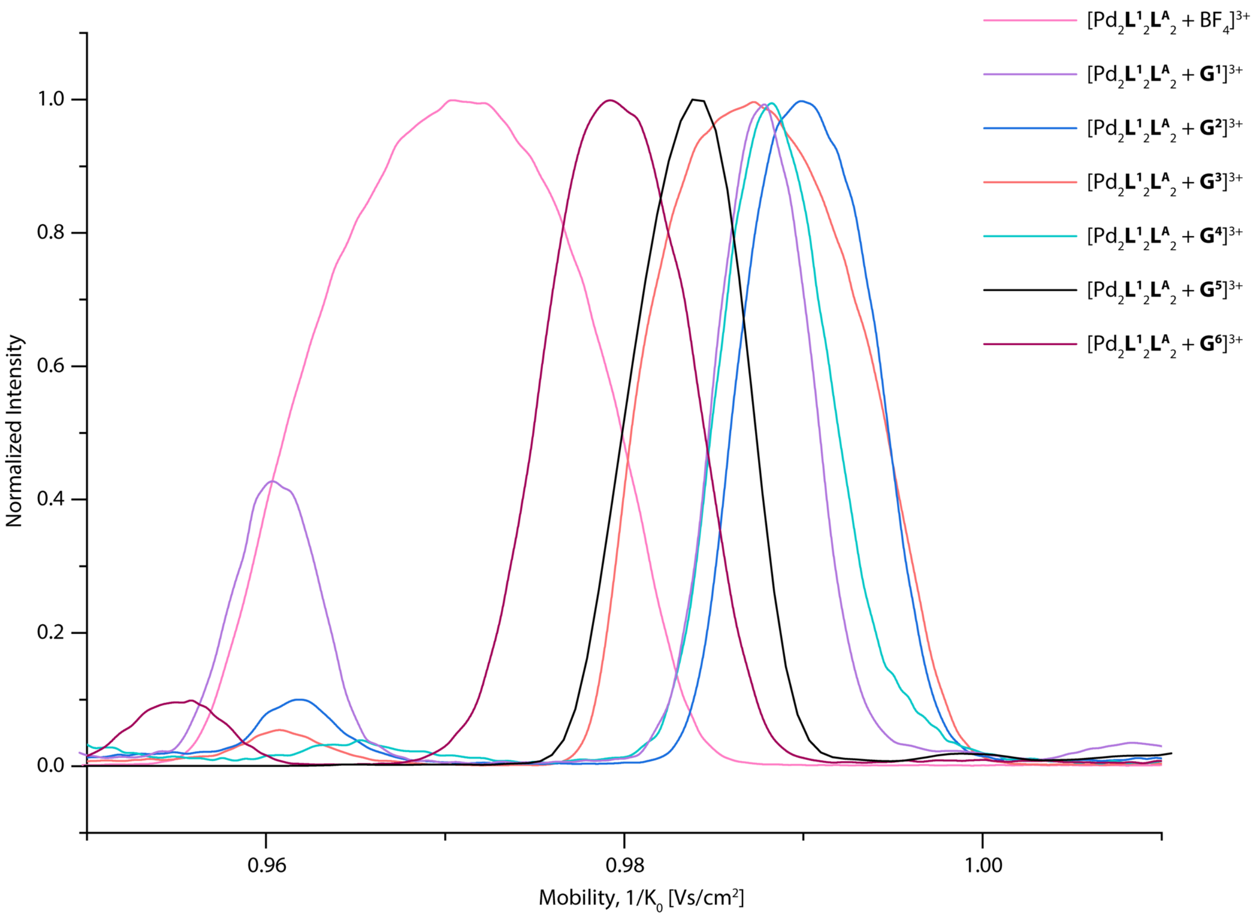

Figure S130: Ion-mobility spectra of $[Pd_2\mathbf{L^1}_2\mathbf{L^A}_2 + BF_4]^{3+}$ and $[Pd_2\mathbf{L^1}_2\mathbf{L^A}_2 + \mathbf{G^{1\text{-}6}}]^{3+}$.

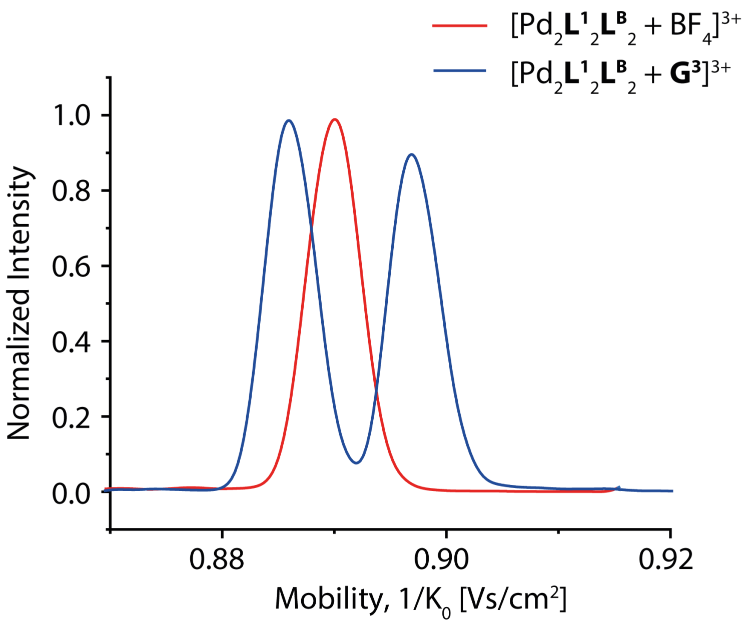

Figure S131: Ion-mobility mass spectra of [Pd_2**L**$^1{}_2$**L**$^B{}_2$ + BF_4]$^{3+}$ and [Pd_2**L**$^1{}_2$**L**$^B{}_2$ + **G**^{3}]$^{3+}$.

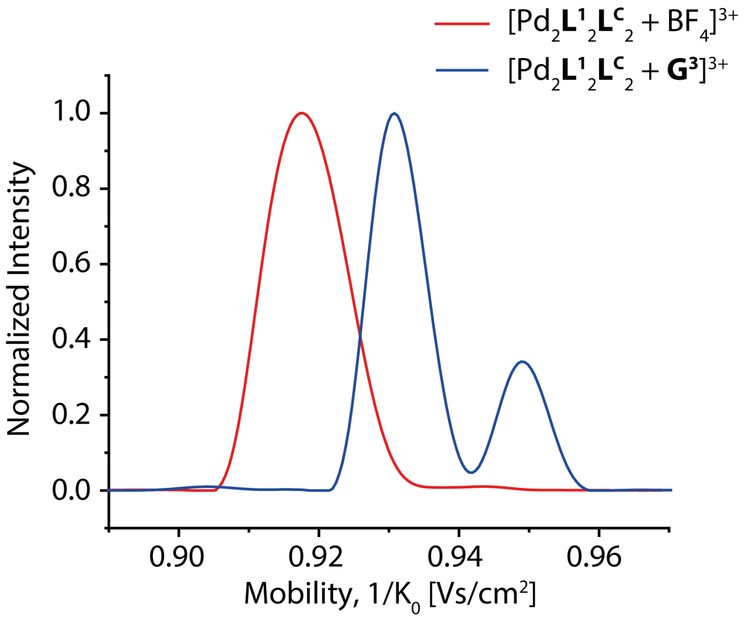

Figure S132: Ion-mobility mass spectra of [Pd_2**L**$^1{}_2$**L**$^C{}_2$ + BF_4]$^{3+}$ and [Pd_2**L**$^1{}_2$**L**$^C{}_2$ + **G**^{3}]$^{3+}$.

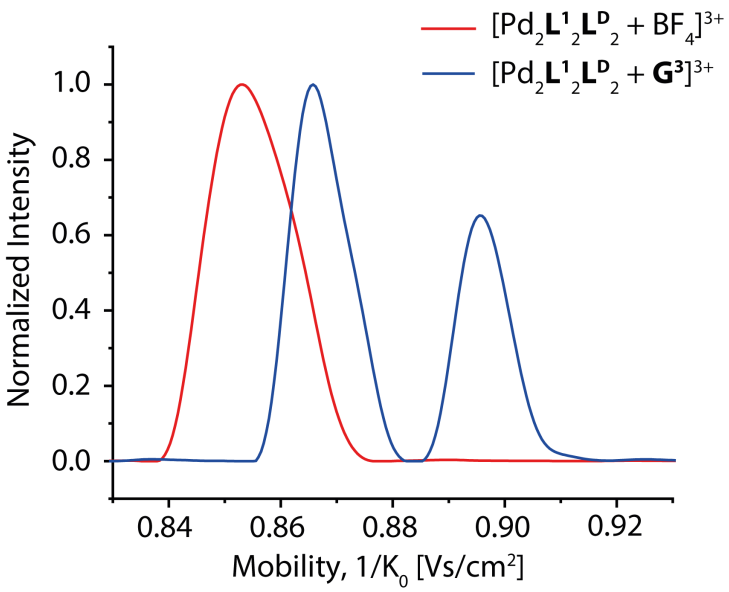

Figure S133: Ion-mobility mass spectra of $[Pd_2\mathbf{L^1}_2\mathbf{L^D}_2 + BF_4]^{3+}$ and $[Pd_2\mathbf{L^1}_2\mathbf{L^D}_2 + \mathbf{G^3}]^{3+}$.

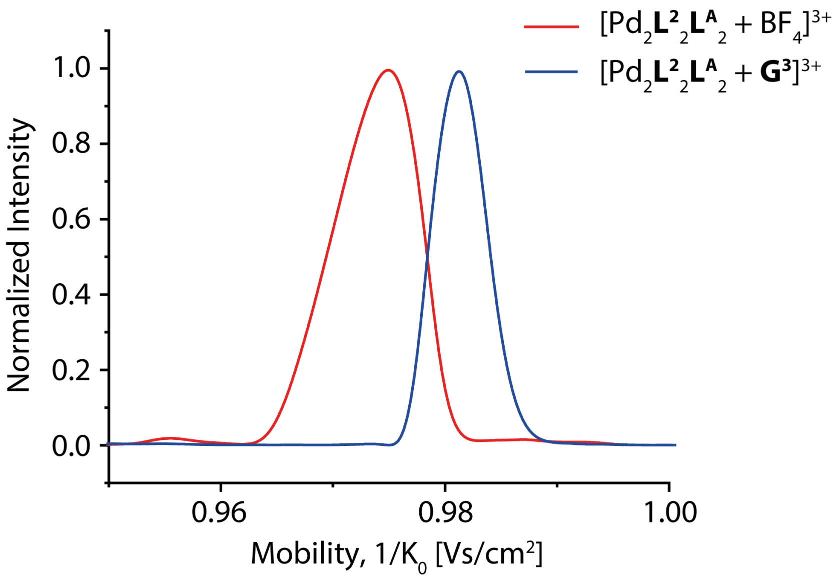

Figure 134: Ion-mobility mass spectra of $[Pd_2\mathbf{L^2}_2\mathbf{L^A}_2 + BF_4]^{3+}$ and $[Pd_2\mathbf{L^2}_2\mathbf{L^A}_2 + \mathbf{G^3}]^{3+}$.

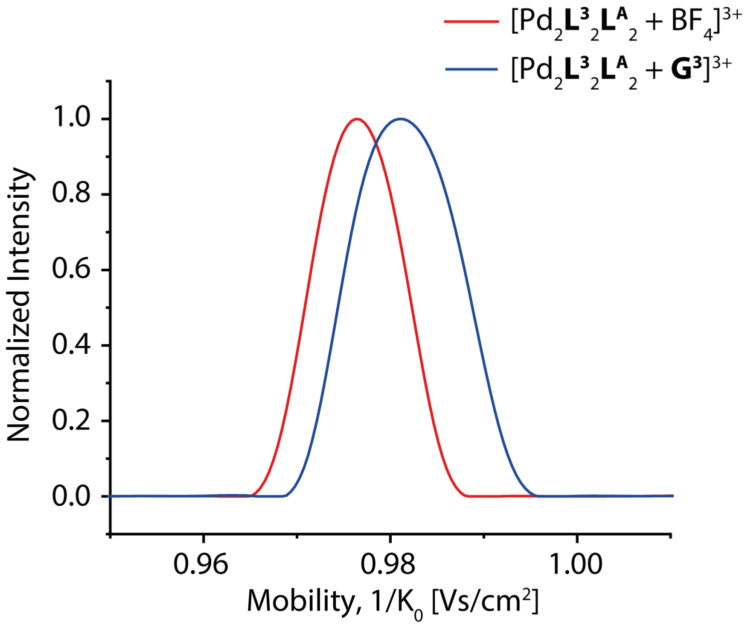

Figure S135: Ion-mobility mass spectra of $[Pd_2\mathbf{L^3}_2\mathbf{L^A}_2 + BF_4]^{3+}$ and $[Pd_2\mathbf{L^3}_2\mathbf{L^A}_2 + \mathbf{G^3}]^{3+}$.

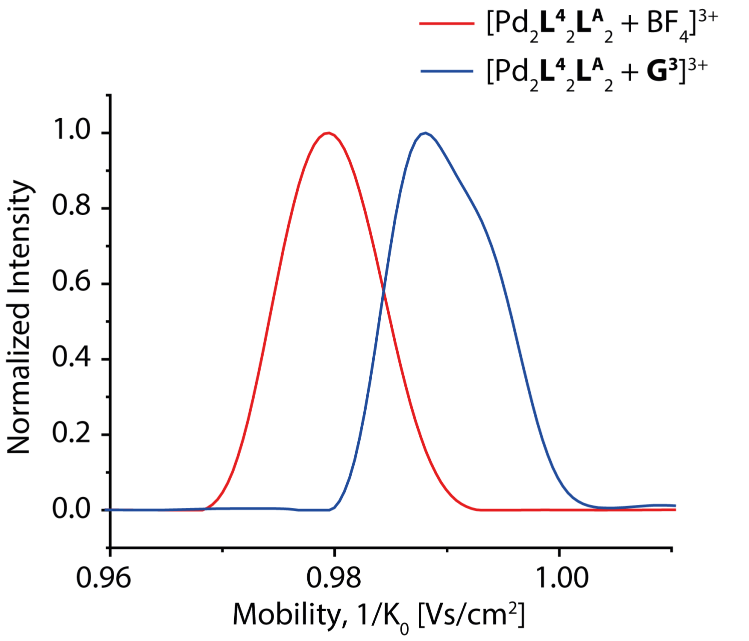

Figure S136: Ion-mobility mass spectra of $[Pd_2\mathbf{L^4}_2\mathbf{L^A}_2 + BF_4]^{3+}$ and $[Pd_2\mathbf{L^4}_2\mathbf{L^A}_2 + \mathbf{G^3}]^{3+}$.

Taking a close look at the measured ion mobility spectra, we observe that the signals are mostly gaussian-shaped or have slight shoulders. For some of the species with bound guest we observe two prominent mobility signals, indicating different co-conformers of the host-guest complexes in the gas phase, probably due to different positions of the guest inside the anisotropic cage. However, we were not able to differentiate between different conformers by means of theoretical CCS calculations (compare below). In all cases, the measured mobilities of the host-guest complexes are close to the mobilities of the hosts containing only a BF_4^- anion, indicating encapsulation inside the cavity (with – depending on the guest – slight protrusion of guest features, i.e. the phosphate's alkyl or aryl substituents outside the cage's apertures).

7.1 Modeling and Theoretical Collisional Cross Sections calculations (CCS)

The host-guest systems were optimized using the quantum chemistry software package ORCA (version 5.0.2),[21] by DFT methods on a PBE/def2-SVP level of theory. The theoretical collisional cross sections (${}^{Theo}CCS_{N2}$) of the optimized models were then calculated with Collidoscope (version 1.4).[22] The number of energy states was set to 16 and the temperature to 303 K. The used CM5 point charges[23] were calculated using the `xtb` software (version 6.4.1) and the semiempirical model GFN1-xTB.[24]

Table S3: Measured and calculated collisional cross sections (CCS) of Host-Guest-Complexes.

Complex	Measured CCS [Å^2]	Calculated CCS [Å^2]	Δ%
$[Pd_2\mathbf{L^1}_2\mathbf{L^A}_2 + BF_4]^{3+}$	595.1	637.273	+7.1%
$[Pd_2\mathbf{L^1}_2\mathbf{L^A}_2]^{3+} + BF_4$ **outside**	-	640.175	+7.6%
$[Pd_2\mathbf{L^1}_2\mathbf{L^A}_2 + \mathbf{G^1}]^{3+}$	604.2	649.624	+7.5%
$[Pd_2\mathbf{L^1}_2\mathbf{L^A}_2 + \mathbf{G^2}]^{3+}$	605.4	652.593	+7.8%
$[Pd_2\mathbf{L^1}_2\mathbf{L^A}_2 + \mathbf{G^3}]^{3+}$	603.7	652.774	+8.1%
$[Pd_2\mathbf{L^1}_2\mathbf{L^A}_2]^{3+} + \mathbf{G^3}$ **outside**	-	688.833	+14.1%
$[Pd_2\mathbf{L^1}_2\mathbf{L^A}_2 + \mathbf{G^4}]^{3+}$	601.1	647.946	+7.8%
$[Pd_2\mathbf{L^1}_2\mathbf{L^A}_2 + \mathbf{G^5}]^{3+}$	601.7	655.126	+8.9%
$[Pd_2\mathbf{L^1}_2\mathbf{L^A}_2 + \mathbf{G^6}]^{3+}$	599.0	652.191	+8.9%
$[Pd_2\mathbf{L^1}_2\mathbf{L^B}_2 + BF_4]^{3+}$	545.6	585.424	+7.3%
$[Pd_2\mathbf{L^1}_2\mathbf{L^B}_2 + \mathbf{G^3}]^{3+}$	549.2	594.947	+8.3%
$[Pd_2\mathbf{L^1}_2\mathbf{L^C}_2 + BF_4]^{3+}$	555.1	620.360	+11.8%
$[Pd_2\mathbf{L^1}_2\mathbf{L^C}_2 + \mathbf{G^3}]^{3+}$	562.7	626.520	+11.3%
$[Pd_2\mathbf{L^1}_2\mathbf{L^D}_2 + BF_4]^{3+}$	516.6	573.185	+11.0%
$[Pd_2\mathbf{L^1}_2\mathbf{L^D}_2 + \mathbf{G^3}]^{3+}$	523.9	575.587	+9.9%
$[Pd_2\mathbf{L^2}_2\mathbf{L^A}_2 + BF_4]^{3+}$	597.0	-	
$[Pd_2\mathbf{L^2}_2\mathbf{L^A}_2 + \mathbf{G^3}]^{3+}$	600.3	-	
$[Pd_2\mathbf{L^3}_2\mathbf{L^A}_2 + BF_4]^{3+}$	590.5	639.362	+8.3%
$[Pd_2\mathbf{L^3}_2\mathbf{L^A}_2 + \mathbf{G^3}]^{3+}$	592.7	643.551	+8.6%
$[Pd_2\mathbf{L^4}_2\mathbf{L^A}_2 + BF_4]^{3+}$	592.3	644.968	+8.9%
$[Pd_2\mathbf{L^4}_2\mathbf{L^A}_2 + \mathbf{G^3}]^{3+}$	597.0	642.655	+7.6%

For this system we observe a relatively large average deviation, around +7.5%, of the theoretical value compared to the experimental data. This could be due to neglecting dynamic cage motions in the CCS calculation technique and also due to the unique, funnel-like shape of the cage that may be problematic in the used trajectory method. The error is especially large for the $[Pd_2\mathbf{L^1}_2\mathbf{L^C}_2 + X]^{3+}$ species, which may be attributed to the two hexyl side chains, considered straight in the DFT models but probably adopting more folded conformations in the gas phase. The slight increase of the CCS value by replacing a BF_4^- anion with a guest, as observed experimentally, could be reproduced in the computations. As discussed in the main text, considering a guest binding mode outside of the cage would lead to very high deviations between measured and calculated CCS values (see 6th entry in Table S2), which is why we assume the guest to be always inside the cage during the TIMS measurement.

8 UV/Vis and CD spectroscopy

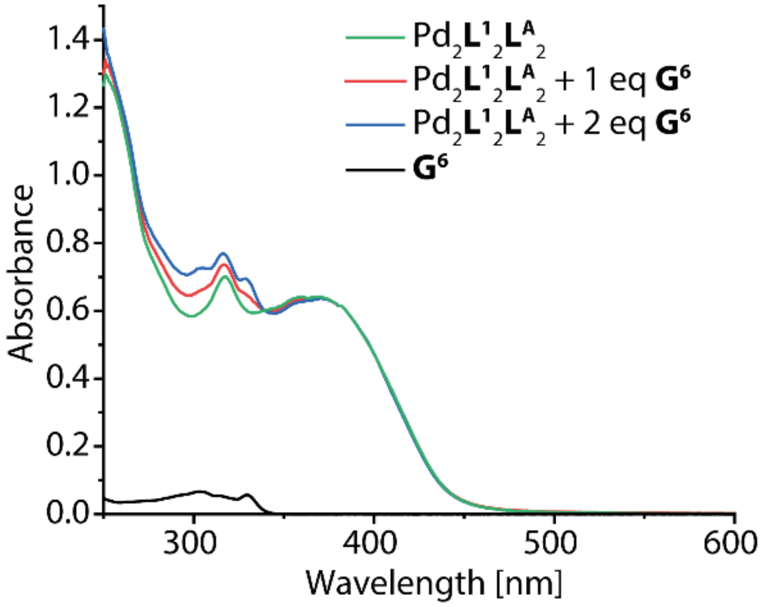

Figure S137: UV/vis spectra of Pd_2**L^1**$_2$**L^A**$_2$ (green, c = 0.07 mM), **G^6**@Pd_2**L^1**$_2$**L^A**$_2$ (1 eq red, 2 eq blue, c = 0.07 mM) and **G^6** (black, c = 0.07 mM).

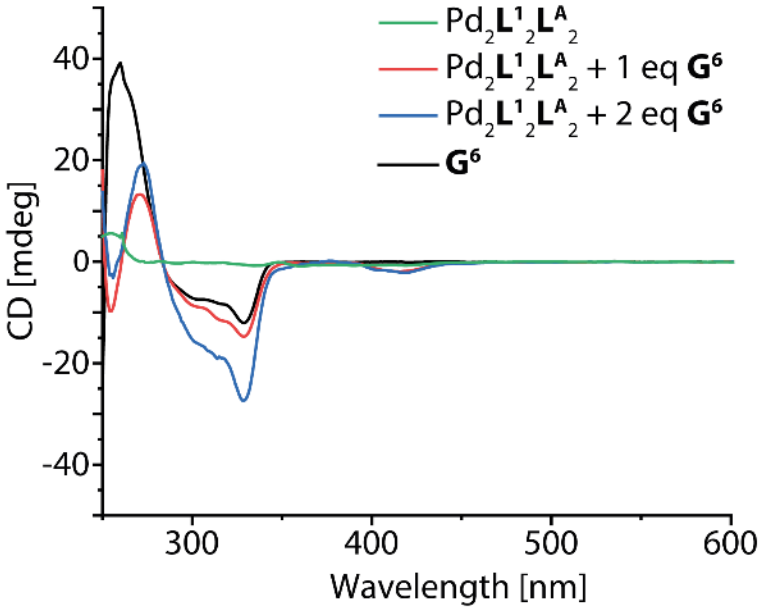

Figure S138: CD spectra of Pd_2**L^1**$_2$**L^A**$_2$ (green, c = 0.07 mM), **G^6**@Pd_2**L^1**$_2$**L^A**$_2$ (1 eq red, 2 eq blue, c = 0.07 mM) and **G^6** (black, c = 0.07 mM).

A small CD signal in the absorption range of the host-guest complex > 400 nm was observed upon addition of the chiral guest to the achiral host, indicating a certain degree of chirality transfer from the guest to the host.

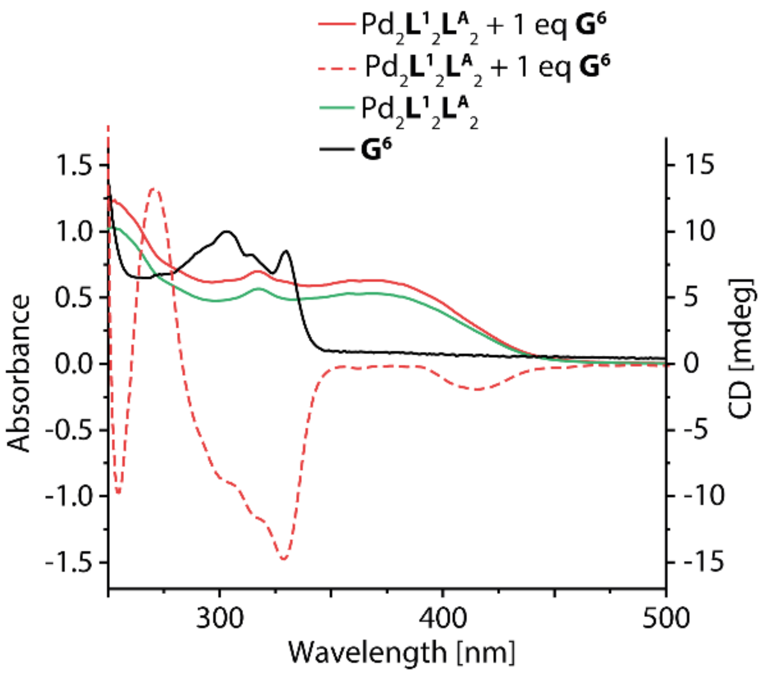

Figure S139: Overlay of UV/Vis (solid lines) and CD (dashed line) spectra of Pd_2**L**$^1{}_2$**L**$^A{}_2$ (green, c = 0.07 mM), **G**^{6}@Pd_2**L**$^1{}_2$**L**$^A{}_2$ (1 eq red, c = 0.07 mM) and **G**^{6} (black, c = 0.7 mM), showing that the emerging CD band > 400 nm belongs exclusively to a host absorption.

9 Molecular Dynamics Simulations

Initial general AMBER force field (GAFF) parameters[25] and topologies for the heteroleptic cages $[Pd_2\mathbf{L}^1{}_2\mathbf{L}^A{}_2]^{4+}$ and $[Pd_2\mathbf{L}^2{}_2\mathbf{L}^A{}_2]^{4+}$ as well as for the diphenylphosphate guest **G**^{3} were generated with the CHIMERA software.[26] Parameters for the bonds and angles involving Pd were taken from our previous work,[27] and Lennard-Jones (6,12) parameters for the palladium were taken from Yoneya et al.[28] Atomic partial charges for host and guest were calculated with the ESP method using the DFT functional B3LYP[29] with the 6-31G* all-electron basis set for all atoms except Pd, for which the Stuttgart-Dresden (SDD) pseudopotentials[30] were used.

After force field parametrisation, the cage and one guest molecule were placed randomly in a periodic simulation box with a volume of ca. 67 nm^3 and solvated in DMSO, for which parameters were taken from the work of van der Spoel et al.[31] Three tetrafluoroborate anions were added in the solvent to keep the overall charge of the simulation box zero. The system was energy minimized using steepest descent and then equilibrated at 298 K in a 500 ps NVT simulation, followed by 500 ps NPT simulation. The final production runs were carried out in the NPT ensemble. The LINCS algorithm[32] was used to constrain bond lengths involving H-atoms, allowing to integrate the equations of motion with 2 fs time steps using the leapfrog integrator. Temperature was kept constant at 298 K with the velocity-rescale thermostat of Bussi and coworkers[33] with a coupling time constant of 0.1 ps. To maintain constant 1 bar pressure, the Berendsen barostat was used with a coupling time constant of 2 ps. Short-range Lennard-Jones (6,12) and Coulomb interactions were treated with a buffered Verlet pair list with a cut-off of 1.0 nm. Long-range Coulomb interactions were treated with the

PME algorithm with 0.12 nm grid spacing. Analytical corrections to energy and pressure were applied to compensate for the truncation of the Lennard-Jones interactions. In the production runs, unbiased MD simulations of the host-guest system were performed. For each of the two cages investigated, ten independent simulations, each 5 μs long, were carried out, yielding a total sampling time of 100 μs. These extended simulation times enabled the observation of multiple spontaneous binding and unbinding events. The total number of binding/unbinding events observed during the simulations was 32 and 70 for the cages $[Pd_2\mathbf{L}^1{}_2\mathbf{L}^A{}_2]^{4+}$ and $[Pd_2\mathbf{L}^2{}_2\mathbf{L}^A{}_2]^{4+}$, respectively. These statistics allow one to estimate the free energy of binding directly through counting.[34] The free energy of binding was obtained according to equation 1.

$$(1) \qquad \Delta G = -RT * \ln\left(\frac{p_b}{p_u}\right) - RT * ln\left(\frac{V}{V^{Ref}}\right)$$

where the probabilities to find the system in the bound and unbound states, p_b and p_u, respectively, are given by the fractions of the total simulation time that the guest is found inside and outside of the cage, respectively, R is the gas constant, T is the temperature, V is the volume of the simulation box, and V^{Ref} is a reference volume. Using a reference volume that corresponds to the chemical standard state concentration of 1 mol/l (V^{Ref} = 1.66 nm^3) yields the standard-state binding free energy. The bound state was defined based on the distance between the center-of-mass of the cage to the P-atom of the diphenylphosphate guest. A histogram of the distance distribution obtained from the total 50 μs of simulation time for the cage $[Pd_2\mathbf{L}^1{}_2\mathbf{L}^A{}_2]^{4+}$ is shown in Figure S141. The bound state was defined up to a distance of 1.4 nm, where the distribution has a minimum. The statistical uncertainties were estimated from the standard deviation of the ΔG's computed from the 10 individual 5 μs simulations.

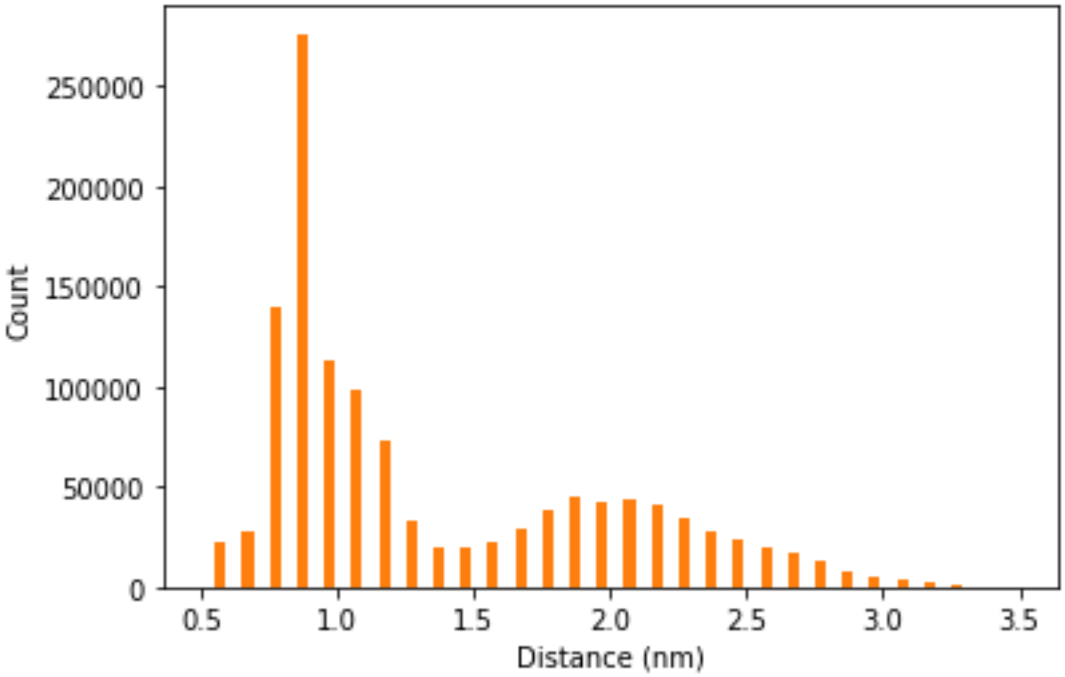

Figure S140: Distribution of the distance between the center-of-mass of the cage and the P-atom of the guest.

In addition to the above simulations with guest **G³**, we carried out MD simulations of the diethyl phosphate guest **G⁴** with the methylated cage $[Pd_2\mathbf{L}^2{}_2\mathbf{L}^A{}_2]^{4+}$ (note that no data is given for **G⁴** with $[Pd_2\mathbf{L}^1{}_2\mathbf{L}^A{}_2]^{4+}$, since a large kinetic barrier for unbinding of the diethylphosphate guest from the cage in the MD simulations prohibited the extraction of statistically reliable results). Ten individual simulations of length 1.2 μs were carried out (all other simulation parameters as well as the simulation setup were analogous to the previous simulations described above). In total, 31 binding events were observed during the accumulated simulation time of 12 μs, again allowing one to estimate the binding free energy with statistical precision (from equation 1). For the smaller **G⁴** guest, the distance criterion to separate the bound and unbound states (see above) was 0.6 nm.

To more closely analyse the binding mode of the guests **G³** and **G⁴** to the cage, only the chunks of the MD trajectories in which the guest was bound inside the cage cavity were analysed. The likelihood to find particular interactions between the different chemical moieties of the cage and the guest, as given by the percentages in Figure 5 in the main text, are the fraction of the time where the indicated interactions were found to be present. An H-bond between cage and guest was defined if the distance between donor (cage NH group) and acceptor (guest phosphate group) was smaller than 0.35 nm and the hydrogen - donor - acceptor angle was below 30 degrees. The cage can also interact with the guest through nonpolar interactions, for example between the π-surfaces. These interactions are possible also with the methylated cage. To quantify the nonpolar contacts in our MD simulations, contacts between the cage and the two phenyl rings of the guest were counted in the bound state. A contact was defined if the distance between the center-of-mass of 3 atoms forming one ring of the cage (i.e., the ligands) and the center-of-mass of 3 atoms in the phenyl rings of the guest was closer than 0.6 nm. For all multi-ring ligands of the cage, the nonpolar contacts were counted according to the previously mentioned conditions for each ring separately and then summed over all rings that constitute the full ligand. Several simultaneous contacts of neighboring rings with the guest were counted as only one contact. Note that the contact percentages (given in Figure 5 in the main text) do not necessarily sum up to 100%, first because the different contacts are not mutually exclusive and second because the guest can be inside the cage (i.e., bound) but transiently not form any H-bond or nonpolar contact.

In addition to the contacts between the structural components of the host and the guest, the interaction energies of each of these components with the guests **G³** and **G⁴** were calculated to take a closer look at the strength of these interactions. For that, energy groups were defined in Gromacs for each structural element of the cage, and the short-range Coulomb and Lennard-Jones interactions with the guest were calculated for each time step and then summed up and averaged over the simulation chunks were the guest was bound. The results are shown in Tables S4 and S5 for **G³** and **G⁴**, respectively.

Table S4: Average interaction energies (Lennard-Jones and Coulomb interactions summed up) of the different structural elements of the cages $[Pd_2\mathbf{L}^1{}_2\mathbf{L}^A{}_2]$ (featuring the endohedral NH group) and $[Pd_2\mathbf{L}^2{}_2\mathbf{L}^A{}_2]$ (with endohedral NMe) with the guest **G³**. Interactions with the solvent and the BF_4^- counter ions were not considered for this calculation. LIC refers to the orange colored structural element in Figure 5 (excluding the NH/N-methyl group, whose interactions are analyzed separately), LPL refers to the red part, Py to the green part and Q to the blue part. NH / N-methyl refers to the NH group (respectively the methyl group) in the LIC element.

Energy (kJ/mol)	$[Pd_2\mathbf{L}^1{}_2\mathbf{L}^A{}_2]$	$[Pd_2\mathbf{L}^2{}_2\mathbf{L}^A{}_2]$	$\Delta([Pd_2\mathbf{L}^1{}_2\mathbf{L}^A{}_2] - [Pd_2\mathbf{L}^2{}_2\mathbf{L}^A{}_2])$
NH / NMe	-30.5 ± 2.1	-3.9 ± 0.5	-26.6 ± 2.6
LIC	-36.4 ± 1.8	-9.7 ± 6.3	-26.7 ± 8.1
LPL	-16.4 ± 1.1	-22.6 ± 0.1	6.2 ± 1.2
Py	-29.4 ± 1.7	-40.5 ± 2.6	11.1 ± 4.3
Q	-3.2 ± 0.4	-1.3 ± 0.3	-1.9 ± 0.7

The interaction with the LIC part of the cage, which contains the NH function, is the strongest interaction, since the guest interacts through an H-bond with the NH group and additionally trough nonpolar contacts with the aromatic rings. The same interaction in $[Pd_2\mathbf{L}^2{}_2\mathbf{L}^A{}_2]^{4+}$, where the endohedral NH is replaced by NMe is significantly weaker, showing that the H-bond leads to interactions that strengthen the binding. Interestingly, the interaction with the non-H-bonding ligand LPL is stronger in the $[Pd_2\mathbf{L}^2{}_2\mathbf{L}^A{}_2]^{4+}$ cage, which shows that the ligand partly compensates the lack of the H-bond through stronger nonpolar contacts, which it can establish if it is not in an H-bonded configuration. The pyridine rings (Py, green) also interact strongly with the guest through non-polar contacts, showing that they also contribute to the guest binding. This interaction also becomes stronger in the absence of the H-bond. Taken together, this interaction energy analysis shows that the H-bond indeed strengthens the guest binding in this heteroleptic cage, and that the other, non-H-bonding ligand, also contributes to the binding and partly compensates the absence of the H-bond in the methylated form of the cage.

Table S5: Average interaction energies (Lennard-Jones and Coulomb interactions summed up) of the different structural elements of cage $[Pd_2\mathbf{L}^2{}_2\mathbf{L}^A{}_2]$ (with endohedral NMe) with the guests $\mathbf{G}^3$ and $\mathbf{G}^4$. Interactions with the solvent and the BF_4^- counter ions were not considered for this calculation. LIC refers to the orange-colored structural element in Figure 5 (excluding the NH/N-methyl group, whose interactions are analyzed separately), LPL refers to the red part, Py to the green part and Q to the blue part. NH / N-methyl refers to the NH group (respectively the methyl group) in the LIC element.

Energy (kJ/mol)	$\mathbf{G}^3$@$[Pd_2\mathbf{L}^2{}_2\mathbf{L}^A{}_2]$	$\mathbf{G}^4$@$[Pd_2\mathbf{L}^2{}_2\mathbf{L}^A{}_2]$	$\Delta([\mathbf{G}^3\text{-}\mathbf{G}^4])$
NMe	-3.9 ± 0.5	-2.7 ± 0.3	-1.3 ± 0.8
LIC	-9.7 ± 6.3	-5.1 ± 3.2	-4.6 ± 9.5
LPL	-22.6 ± 0.1	-15.2 ± 1.3	-7.3 ± 1.4
Py	-40.5 ± 2.6	-45.1 ± 1.0	4.6 ± 3.6
Q	-1.3 ± 0.3	-0.8 ± 0.2	-0.5 ± 0.5

10 Literature

[1] A. Jerschow, N. Müller, *J. Magn. Reson.* **1996**, *123*, 222.

[2] A. Jerschow, N. Müller, *J. Magn. Reson.* **1997**, *125*, 372.

[3] E. O. Stejskal, J. E. Tanner, *J. Chem. Phys.* **1965**, *42*, 288.

[4] J. E. Tanner, E. O. Stejskal, *J. Chem. Phys.* **1968**, *49*, 1768.

[5] A. Einstein, *Ann. d. Phys.* **1905**, *4*, 549.

[6] K. E. Ebbert, L. Schneider, A. Platzek, C. Drechsler, B. Chen, R. Rudolf, G. H. Clever, *Dalton Trans.* **2019**, *48*, 11070.

[7] W. M. Bloch, Y. Abe, J. J. Holstein, C. M. Wandtke, B. Dittrich, G. H. Clever, *J. Am. Chem. Soc.* **2016**, *138*, 13750.

[8] A. Burkhardt, T. Pakendorf, B. Reime, J. Meyer, P. Fischer, N. Stübe, S. Panneerselvam, O. Lorbeer, K. Stachnik, M. Warmer, P. Rödig, D. Göries, A. Meents, *European Phys. J. Plus* **2016**, *131*, 56.

[9] W. Kabsch, *Acta Crystallogr. D.* **2010**, *66*, 125.

[10] G. M. Sheldrick, *Acta Crystallogr. A* **2015**, *71*, 3.

[11] G. M. Sheldrick, *Acta Crystallogr. C* **2015**, *71*, 3.

[12] C. B. Hübschle, G. M. Sheldrick, B. Dittrich, *J. Appl. Crystallogr.* **2011**, *44*, 1281.

[13] D. Kratzert, I. Krossing, *J. Appl. Crystallogr.* **2018**, *51*, 928.

[14] D. Kratzert, J. J. Holstein, I. Krossing, *J. Appl. Crystallogr.* **2015**, *48*, 933.

[15] A. Thorn, B. Dittrich, G. M. Sheldrick, *Acta Crystallogr. A* **2012**, *68*, 448.

[16] A. L. Spek, *Acta Crystallogr. C* **2015**, *71*, 9.

[17] A. L. Spek, *Acta Crystallogr. D* **2009**, *65*, 148.

[18] F. A. Fernandez-Lima, D. A. Kaplan, M. A. Park, *Rev. Sci. Instrum.* **2011**, *82*, 126106.

[19] J.-F. Greisch, J. Chmela, M. E. Harding, D. Wunderlich, B. Schäfer, M. Ruben, W. Klopper, D. Schooss, M. M. Kappes, *Phys. Chem. Chem. Phys.* **2017**, *19*, 6105.

[20] D. R. Hernandez, J. D. DeBord, M. E. Ridgeway, D. A. Kaplan, M. A. Park, F. Fernandez-Lima, *Analyst* **2014**, *139*, 1913.

[21] F. Neese, *Wiley Interdiscip Rev Comput Mol Sci* **2018**, *8*, DOI 10.1002/wcms.1327.

[22] S. A. Ewing, M. T. Donor, J. W. Wilson, J. S. Prell, *J. Am. Soc. Mass Spectr.* **2017**, *28*, 587.

[23] A. V. Marenich, S. V. Jerome, C. J. Cramer, D. G. Truhlar, *J. Chem. Theory Comput.* **2012**, *8*, 527.

[24] S. Grimme, C. Bannwarth, P. Shushkov, *J. Chem. Theory Comput.* **2017**, *13*, 1989.

[25] J. Wang, R. M. Wolf, J. W. Caldwell, P. A. Kollman, D. A. Case, *J. Comput. Chem.* **2004**, *25*, 1157.

[26] E. F. Pettersen, T. D. Goddard, C. C. Huang, G. S. Couch, D. M. Greenblatt, E. C. Meng, T. E. Ferrin, *J. Comput. Chem.* **2004**, *25*, 1605.

[27] S. Juber, S. Wingbermühle, P. Nuernberger, G. H. Clever, L. V. Schäfer, *Phys. Chem. Chem. Phys.* **2021**, *23*, 7321.

[28] M. Yoneya, S. Tsuzuki, T. Yamaguchi, S. Sato, M. Fujita, *Acs Nano* **2014**, *8*, 1290.

[29] A. D. Becke, *J. Chem. Phys.* **1993**, *98*, 5648.

[30] A. Bergner, M. Dolg, W. Küchle, H. Stoll, H. Preuß, *Mol. Phys.* **1993**, *80*, 1431.

[31] C. Caleman, P. J. van Maaren, M. Hong, J. S. Hub, L. T. Costa, D. van der Spoel, *J. Chem. Theory Comput.* **2012**, *8*, 61.

[32] B. Hess, *J. Chem. Theory Comput.* **2008**, *4*, 116.

[33] G. Bussi, D. Donadio, M. Parrinello, *J. Chem. Phys.* **2007**, *126*, 014101.

[34] D. H. D. Jong, L. V. Schäfer, A. H. D. Vries, S. J. Marrink, H. J. C. Berendsen, H. Grubmüller, *J. Comput. Chem.* **2011**, *32*, 1919.
